# Case Report: Immune checkpoint inhibitor exhibits dual benefits for a refractory lymphoma patient with disseminated mucormycosis

**DOI:** 10.3389/fmed.2025.1608828

**Published:** 2025-07-02

**Authors:** Qibei Teng, Qianqian Yang, Zhizhi Tao, Jinwen Huang, Cheng Zhang, Fangfei Shao, Wenjue Pan, Xiujie Zhao, Haowen Xiao

**Affiliations:** ^1^Department of Hematology and Cell Therapy, Sir Run Run Shaw Hospital, School of Medicine, Zhejiang University, Hangzhou, Zhejiang, China; ^2^Department of Radiology, Sir Run Run Shaw Hospital, School of Medicine, Zhejiang University, Hangzhou, Zhejiang, China; ^3^Department of Hematology, Sir Run Run Shaw Hospital, School of Medicine, Zhejiang University, Hangzhou, Zhejiang, China

**Keywords:** lymphoma, invasive mucormycosis, *Rhizomucor pusillus*, immune checkpoint inhibitor, Bruton tyrosine kinase inhibitor, immunomodulator

## Abstract

Invasive mucormycosis contributes to a high mortality rate in patients with hematological malignancies. When it disseminates to multiple organs, especially to the brain, the prognosis is extremely poor. The traditional antifungal strategies including surgery have less efficacy in patients with hematological malignancies. Here, we describe a case report of a 62-year-old man with refractory lymphoma who developed mucormycosis caused by *Rhizomucor pusillus* after chemotherapy, which disseminated extensively to the lungs, diaphragm, brain and spleen. He was successfully treated with antifungal agents combined with PD-1 inhibitor, which may simultaneously exhibit efficacy in treatment of lymphoma and mucormycosis. The patient’s lymphoma and mucormycosis were well controlled; ultimately, autologous hematopoietic stem cell transplantation was successfully performed, and the patient achieved long-term survival.

## 1 Introduction

Invasive mucormycosis (IM) is an invasive fungal disease (IFD) caused by Mucorales fungi. It predominantly affects immunosuppressed individuals with hematological malignancies (HMs) after chemotherapy or hematopoietic stem cell transplantation (HSCT) and those with neutropenia, uncontrolled diabetes and COVID-19 (1). The morbidity rate of IM in patients with HMs ranges from 0.07% to 4.29% and increases annually, making it the third most common type of IFD ranged after invasive aspergillosis and invasive candidiasis (1). Disseminated mucormycosis (DM) is defined as mucormycosis involving two or more nonadjacent organs (2). The lung is the most common site of DM, followed by the central nervous system (CNS), sinuses, liver, and kidneys (1). IM has a very high mortality rate, especially in patients with HMs, and those with DM or CNS involvement. A study published in 2005 including 929 IM patients had reported the overall mortality rate of 46%, 66% for those with HMs, 96% for those with DM, and 98% for those with dissemination involving the CNS (2). With the advancement of early diagnostic techniques such as metagenomic next-generation sequencing (mNGS), the development of novel triazoles and the application of minimally invasive surgery, the mortality rate of IM has seemingly decreased over the past two decades (3, 4). However, for patients with HMs who are severely immunosuppressed, the prognosis of IM remains discouraging. A meta-analysis (5) of 811 patients with HMs complicated by IM from 2000 to 2022 reported the overall mortality rate of 61%, particularly the mortality rate of 83% in lymphoma patients. New therapeutic strategies are urgently needed. How to balance the immunosuppression caused by anti-lymphoma treatment and immune function required by anti-IFD remains an unsolved question. Here, we reported a case report of the successful combination of PD-1 inhibitor and antifungal agents in a patient with refractory lymphoma, with mucormycosis involving the lungs, diaphragm, CNS and spleen.

## 2 Case presentation

**Figure 1:**
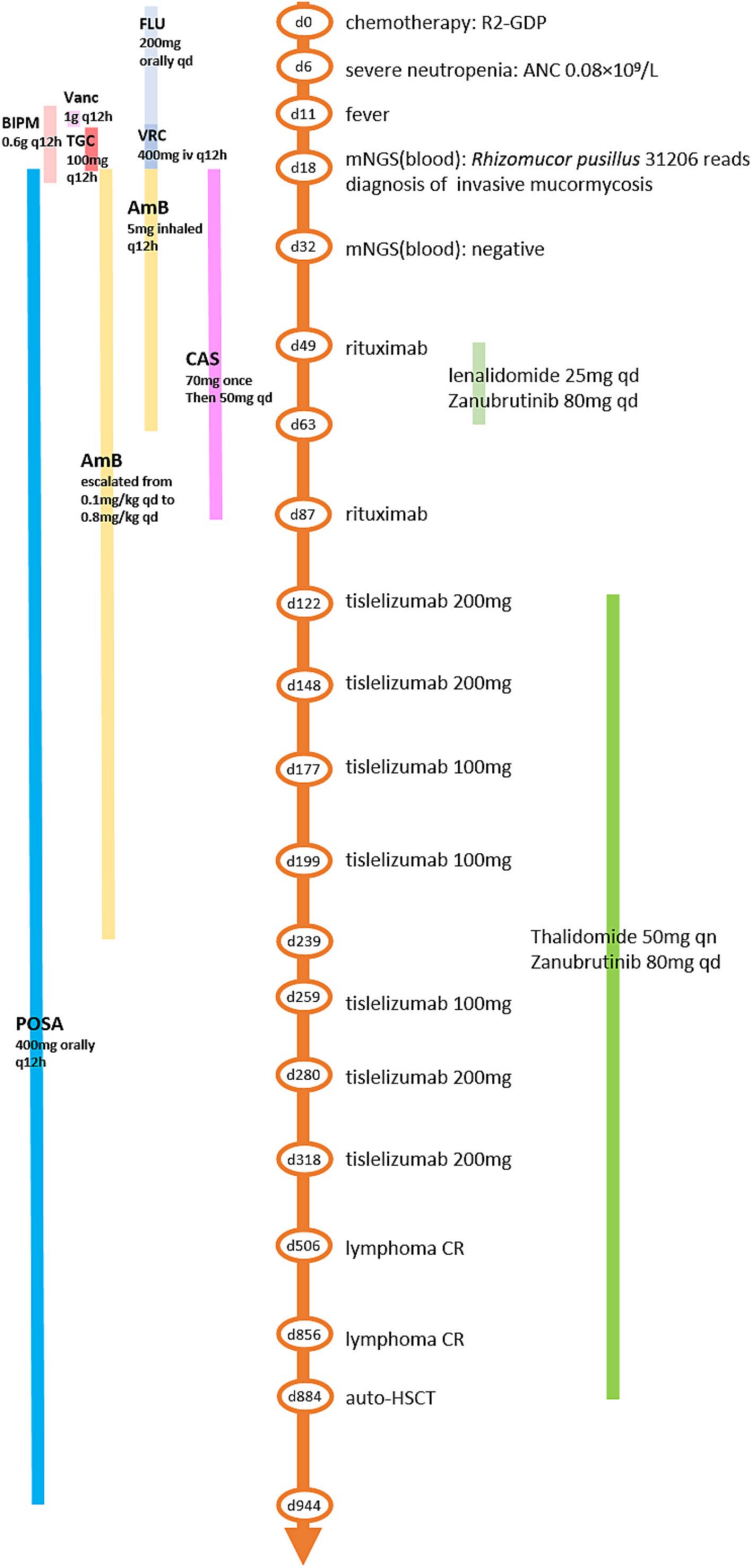
The timeline of the clinical course. Chemotherapy R2-GDP, rituximab, lenalidomide, gemcitabine, cisplatin, and dexamethasone; ANC, absolute neutrophil count; mNGS, metagenomics next-generation sequencing; CR, complete remission; auto-HSCT, autologous hematopoietic stem cell transplantation; FLU, fluconazole; VRC, voriconazole; AmB, amphotericin B; POSA, posaconazole; BIPM, biapenem; Vanc, vancomycin; TGC, tigecycline; qd, once a day; qn, once a night; q12h, every 12 h.

A 62-year-old man with type 2 diabetes was diagnosed with high-risk diffuse large B-cell lymphoma (DLBCL, subtype GCB, stage IV, group B, with an IPI score of 5) involving the lumbar vertebra, pancreas, pleura and lymph nodes. After 7 cycles of standard chemotherapy (4 cycles of R-CHOP and 3 cycles of R-EPOCH), the patient experienced new enlarged lymph nodes in the abdominal cavity, indicating refractory DLBCL. After 2 cycles of salvage therapy (1 cycle of hyper CVAD-A regimen including cyclophosphamide, vindesine, doxorubicin, and dexamethasone; 1 cycle of R2-GDP regimen including rituximab, lenalidomide, gemcitabine, cisplatin, and dexamethasone), the patient achieved partial remission of lymphoma, and his peripheral hematopoietic stem cells were successfully collected. On November 10, 2020 (indicated as day 0 here), the patient was treated with a standard chemotherapy regimen of R2-GDP (Figure 1). On day 6, the patient presented with severe neutropenia (absolute neutrophil count (ANC) 0.08 × 10^9^/L). He received the treatment of granulocyte colony-stimulating factor. Consequently, Fluconazole (200 mg, orally, daily, days 6–14) and sulfamethoxazole (960 mg, orally, daily, constantly) were empirically given as broad-spectrum infection prophylaxis.

**Figure 2:**
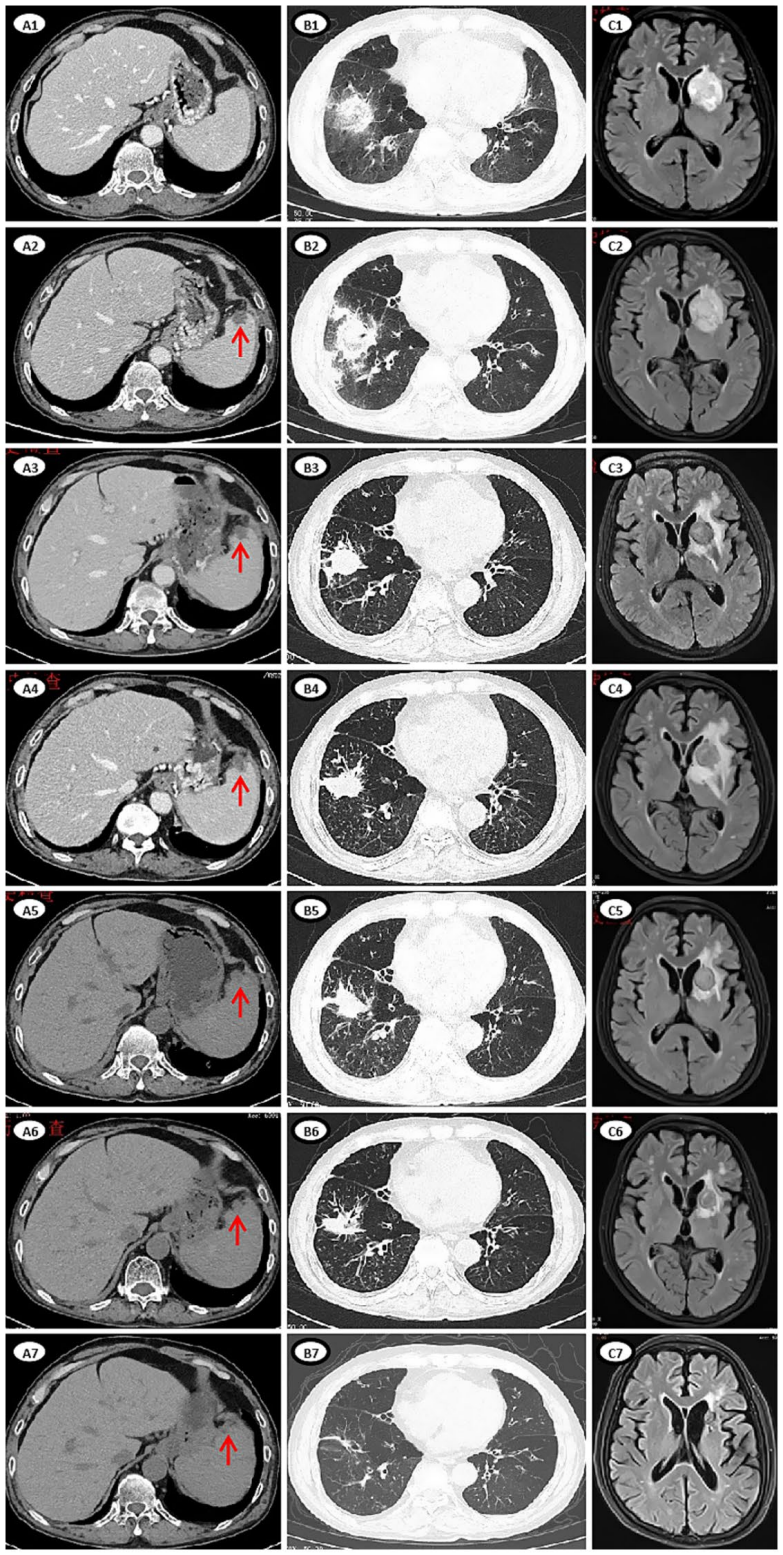
Imaging manifestations of lesions of mucormycosis during treatment. **(A1–A7)** CT images of abdomen. **(A1)** Day 10, no new lesion in the spleen. **(A2)** Day 24, multiple round low-density lesions in the spleen (red arrow), which appeared newly after the last chemotherapy. **(A3)** Day 74, some lesions were slightly reduced. **(A4)** Day 106, the lesions were slightly larger. **(A5)** Day 135, the lesions were smaller. **(A6)** Day 169, the lesions were smaller. **(A7)** Day 377, the lesions were no longer apparent. **(B1–B7)** CT images of chest. **(B1)** Day 17, multiple new nodules and consolidation in both lungs, extensive exudation in the right lower lung with a reversed halo sign, involving the right diaphragm. **(B2)** Day 27, the lesion in the right lower lung progressed. **(B3)** Day 75, a cavity formed and the extent of exudation was reduced. **(B4)** Day 119, the lesion was slightly progressed. **(B5)** Day 141. **(B6)** Day 187. **(B7)** Day 476. **(C1–C7)** MRI images of brain. **(C1)** Day 24, a patchy shadow (39 × 28.9 mm) in the left basal ganglia. **(C2)** Day 29, the lesion (41.9 × 30.3 mm) was enlarged. **(C3)** Day 79, size of the lesion (19.1 × 19.1 mm) reduced. **(C4)** Day 107, the lesion (21.3 × 21.2 mm) was slightly enlarged, the area of edema was enlarged. **(C5)** Day 138, size of the lesion (20.2 × 18.8 mm) and edema reduced. **(C6)** Day 192, size of the lesion (16.2 × 15.4 mm) and edema reduced. **(C7)** Day 644, size of the lesion (9.8 × 9.6 mm) decrease significantly.

On day 10, the patient developed abdominal pain in the right lower quadrant. A contrast-enhanced computed tomography (CT) scan of the abdomen revealed no abnormalities (Figure 2A1). On day 11, the patient developed a fever to 39.5°C without cough or chest tightness. Laboratory tests revealed a C-reactive protein (CRP) level of 76.3 mg/L and a procalcitonin level of 0.1 ng/mL. Empirical intravenous treatment with biapenem (0.6 g q12h) and vancomycin (1 g q12h) was initiated. The patient remained persisted fever at 39.5°C, causing weak enough to be bedridden. On day 14, empirical treatment of intravenous antibiotics was changed to biapenem combined with voriconazole (400 mg q12h) and tigecycline (100 mg q12h). However, the patient’s fever showed no remission. On day 16, we sent his peripheral blood sample for mNGS analysis. On day 17, the ANC increased to 1.24 × 10^9^/L, and the abdominal pain subsided as well as the temperature recovered. However, the patient developed persistent hiccups, along with cough, chest tightness, and sputum with blood. A chest CT scan was performed, which revealed multiple new nodules and consolidation in the lungs, as well as extensive exudation in the right lower lung with a reversed halo sign, potentially involving the right diaphragm (Figure 2B1), suggesting fungal infection of the lungs. On day 18, the results of mNGS analysis of his peripheral blood sample revealed 31,206 reads of *Rhizomucor pusillus,* and no other pathogen was detected. Multiple pathogen cultures of blood, sputum and stool samples obtained at the onset of fever and during persist fever, had no positive result to support any pathogen infection. The procalcitonin, (1,3)-*β*-D-glucan, and galactomannan test of the patient’s samples was negative. Esophageal echocardiography was performed without any valvular vegetation. He was diagnosed with IM involving the lungs and diaphragm. The anti-fungal regimen was immediately changed to a combination of amphotericin B (AmB, escalated from 0.1 mg/kg to 0.8 mg/kg intravenously daily; meanwhile, 5 mg was inhaled q12h), posaconazole (POSA, 400 mg orally q12h), and caspofungin (CAS, 70 mg on the first day, followed by 50 mg daily). The patient’s dyspnea and abdominal pain gradually decreased.

**Figure 3:**
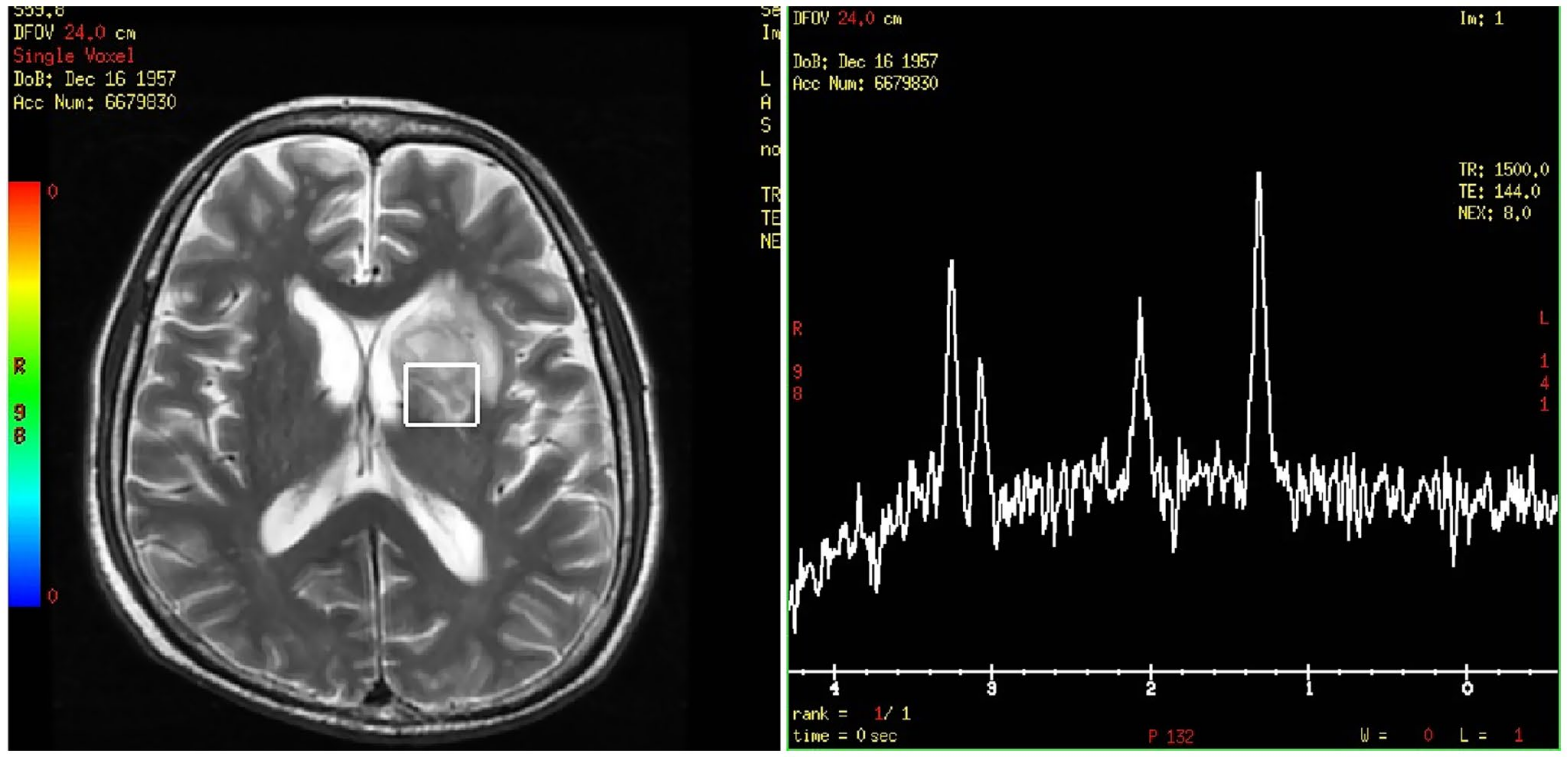
Brain magnetic resonance spectroscopy of the left basal ganglia.

On day 24, the patient presented with transient amnesia of recent events and one episode of fecal incontinence. Physical examination revealed a decline in functions of temporal and spatial orientation. The Glasgow Coma Scale score was 14 (E4V4M6). A brain MRI (Figure 2C1) was performed, which revealed a 39 × 28.9 mm mass in the left basal ganglia and new infarction foci in the left cerebral peduncle and the right cerebellar hemisphere. Moreover, another abdominal CT (Figure 2A2) scan revealed multiple new, rounded, low-density lesions in the spleen compared with the results of previous abdominal CT scan before chemotherapy, which suggested new splenic infarctions. However, no significant evidence of lymphoma progression was observed. On day 27, the patient experienced in continence of urine and feces again and concurrently answered incorrectly about his age. A subsequent chest CT scan revealed progression of the right lower lung lesion (Figure 2B2). A second brain MRI scan revealed an increased size of the mass in the left basal ganglia (Figure 2C2). Lumbar puncture was performed and normal cerebrospinal fluid (CSF) pressure was observed. Results of bacterial culture and mNGS test of CSF were negative. At the same time, we reexamined mNGS analysis of his peripheral blood sample, and no reads of *Rhizomucor* species were detected. On day 37, brain magnetic resonance spectroscopy (Figure 3) was conducted, indicating that the lesion in the left basal ganglia was nonneoplastic. After a discussion among experts in multiple disciplines, they agreed that the newly developed lesions in the lungs, CNS, and spleen were consistent with organ artery invasion by Mucor fungi and/or fungal emboli caused by *Rhizomucor* infection.

The patient was diagnosed with DM involving the lungs, diaphragm, CNS, and spleen. After continuous triple-drug antifungal treatment with AmB, POSA and CAS for 2 months, his symptoms of cough, chest tightness, hiccups, memory loss, fecal and urinary incontinence and abdominal pain were gradually relieved. After triple-drug antifungal therapy for about 3 months, follow-up imaging revealed a reduction in the size of the lesions in the spleen, lungs, and CNS, with no new lesions observed (Figures 2A3,B3,C3). Therefore, CAS treatment was stopped, and AmB (intravenous) treatment was continued for 8 months and POSA treatment was continued for 2.5 years (Figure 1).

Taking into account of a 6-week interruption of lymphoma treatment for the patient, we conducted chemotherapy as well as antifungal therapy to prevent lymphoma progression. On day 49, the patient received a ZR2 regimen, which included Zanubrutinib (ZAN, a Bruton’s tyrosine kinase inhibitor), 80 mg daily for 14 days; lenalidomide, 25 mg daily for 14 days; and rituximab, one 375 mg/m^2^ dose. On day 53, the patient developed a fever which was relieved by empirical treatment of sulbactam-cefoperazone. In addition, cytopenia occurred again after anti-lymphoma treatment. Although the patient received rituximab (375 mg/m^2^) monotherapy on day 87, he developed fever with cough and expectoration again on day 117. The CRP was 55.5 mg/L, and culture of sputum sample identified infection of *Acinetobacter lwoffii*. Piperacillin-tazobactam was initiated, resulting in symptoms relief. Even worse, after two cycles of chemotherapy, radiological imaging revealed that his DM-related lesions had progressed (Figures 2A4,B4,C4). Considering the patient’s weakness and persistent immunosuppressive state (lymphocyte count 0.53 × 10^9^/L, globulin level 15.9 g/L), new strategies for lymphoma were urgently needed.

With the continued administration of AmB and POSA for anti-fungal treatment, we administered the first dose of PD-1 inhibitor (tislelizumab (TIS), 200 mg) on day 122 combined with long-term treatment with thalidomide (THD, 50 mg orally once nightly for 2 years) and ZAN (80 mg daily for 2 years) to control the lymphoma. Follow-up imaging revealed a reduction in lesion size (Figures 2A5,B5,C5). On days 148 and 177, we administered the second (200 mg) and third (100 mg, reduced by half owing to low blood cell counts) doses of TIS. Follow-up imaging indicated a reduction in lesion size in the lungs and CNS, and a slight reduction in size of the spleen lesion (Figures 2A6,B6,C6). On day 199, the fourth dose of TIS was administered at 100 mg. On day 240, AmB was discontinued because the cumulative dose exceeded 3 g, and POSA was continued as long-term antifungal therapy. On days 259, 280, and 318, the fifth, sixth, and seventh doses of TIS were administered at doses of 100 mg, 200 mg, and 200 mg, respectively. Follow-up imaging assessments revealed continuous reduction of all the fungal lesions, while the size of the lymphoma lesions was stable.

During the period of TIS administration every 3–8 weeks in combination with ZAN and THD, there were occasional episodes of slightly low blood counts, but no severe infections occurred. His fungal lesions in the spleen, lungs and CNS stopped shrinking at 12, 15, and 20 months, respectively, after the initiation of antifungal therapy (Figures 2A7,B7,C7). PET/CT revealed complete remission of the lymphoma 15 months and 27 months after the diagnosis of mucormycosis. At this time, the patient’s physical condition had significantly improved. To achieve better long-term survival, autologous HSCT was performed at 28 months after the diagnosis of mucormycosis. THD and ZAN were withdrawn at autologous HSCT for a total of 2 years treatment, and POSA continued. The process of autologous HSCT was successful, and no infections occurred. POSA was continued for half a year after HSCT. The patient was subsequently regularly followed up in the outpatient clinic for 2 years, with normal organ function, good physical status, and all lesions either caused by lymphoma or by mucormycosis almost disappeared.

## 3 Discussion

DM mostly occurs in patients with organ transplants or HMs who have severe neutropenia, especially those with uncontrolled HMs (6). In our case, the patient had refractory lymphoma, severe neutropenia induced by chemotherapy, a history of corticosteroid therapy, and a medical history of diabetes, placing him in a very high-risk group for DM (2). The mucormycosis disseminated to the lungs, diaphragm, CNS and spleen, resulting in an extremely high risk of death. Although the ECMM and ECIL-6 guidelines all strongly recommend liposomal AmB (L-AmB) treatment combined with early and thorough surgery as the first-line treatment for IM (7, 8). For patients with severe conditions, such as HMs, who are not optimal candidates for surgery, a combination treatment of L-AmB with POSA or isavuconazole (ISA) is recommended (7, 8). There are a few studies and case series reported involving successful treatment of DM with a three-drug combination therapy [AmB plus POSA and CAS (9–12), or AmB plus ISA and CAS (13)] in patients with HMs or with immunocompromised status. Owing to the high risk of death, we initially adopted a combination regimen of AmB, POSA, and CAS, which yielded significant efficacy. Although current guidelines do not recommend combination therapy as a first-line strategy due to the lack of definitive data, we think that in patients with HMs, triple-drug combination regimens may represent a viable therapeutic option for DM.

Meanwhile, the patient needed continuous chemotherapy for his refractory lymphoma. In 2019, Andre Goy et al. reported the IR2 regimen (ibrutinib, lenalidomide and rituximab) in patients with relapsed/refractory DLBCL (14). Later, more and more studies on IR2/ZR2 in treatment of DLBCL were reported (15, 16). However, the patient experienced recurring cytopenia and bacterial infection after treatment with ZR2 regimen or rituximab monotherapy, contributing to progression of his DM-related lesions despite the use of antifungal agents. It was an enormous challenge for us to simultaneous management of lymphoma and DM. We choose PD-1 inhibitor combined with BTKi and THD for treatment of lymphoma. ICIs blocking the PD-1/PD-L1 pathway have been successfully used to treat multiple kinds of malignancies, such as melanoma, lung cancer, Hodgkin lymphoma, and some B-cell lymphoma (17). Furthermore, emerging clinical evidence has demonstrated the therapeutic potential of the TIS + ZAN combination in relapsed/refractory DLBCL (18, 19). Although anti-PD-1 therapy has shown limited efficacy in DLBCL compared with other B-cell lymphomas, with an ORR of only 6.12% in monotherapy for relapsed/refractory DLBCL, the combination of anti-PD-1 and BTKi has shown significant improvement, with an ORR of 32.69% (20). Preclinical studies have also demonstrated synergistic antitumor activity between BTKis and PD-1 inhibitors (21). Immunomodulators such as THD and lenalidomide are commonly used to treat lymphoma, and their efficacy is well established (22). Several studies have shown that THD can inhibit the expression of PD-1 (23), increase the antitumor efficacy of PD-1 inhibitors, with relatively mild adverse effects (24, 25).

On the other hand, a growing body of evidence suggests that the immune environment of IFD and cancer shares a common feature of T-cell exhaustion (26). Studies have shown that immune checkpoint molecules such as PD-1 and CTLA-4 play crucial roles in T-cell exhaustion and immune escape (27). The binding of PD-1 to its ligands inhibits T-cell proliferation and cytokine production, thereby impairing host immunity (27). The inhibition of PD-1 has been demonstrated to reverse this dysfunctional state (27). ICIs have become standard treatments for certain types of cancer. The potential therapeutic efficacy of ICIs is being increasingly explored in the field of infectious diseases (28–30). Research has shown that the expression of PD-1 on T cells is strongly correlated with the mortality rate in patients with invasive candidiasis (31). Blockade of the PD-1/PD-L1 pathway leads to modest increases in the levels of proinflammatory cytokines and chemokines associated with fungal clearance (e.g., GM-CSF and TNF-*α*) (32). ICIs can enhance host immunity by increasing the recruitment of innate immune cells, restoring antigen-presenting cell (APC)/T-cell interactions, and promoting the maturation and antifungal activity of APCs, thus facilitating the clearance of opportunistic fungi (33). In animal models of several IFDs, such as mucormycosis, aspergillosis and cryptococcosis, anti-PD-1 therapy significantly reduces the fungal burden in the brain, lungs and kidneys and increases fungal clearance rates, thereby increasing survival rates in animals with lethal infections (32, 34–36).

In addition to animal studies, five published clinical cases suggested that ICIs may be beneficial as salvage therapy for intractable mucormycosis (37–41) (Table 1). The patients in the cases include previously healthy individuals, as well as immunosuppressed individuals with COVID-19 or cancer. Some patients were infected by one genus of Mucorales, whereas others were infected by multiple genera of Mucorales or coinfected with Aspergillus. What they had in common was that mucormycosis spread widely and could not be controlled by surgery or multidrug therapy. High expression of PD-1 on T cells was detected in all of these patients. All patients were treated with nivolumab combined with IFN-*γ* in combination with antifungal agents and achieved an initial clinical response or even full recovery, regardless of their immune status and number of fungal species they were infected with. Among patients with normal immunity, nivolumab was administered as a single dose, whereas immunocompromised patients generally received repeated doses. One patient with CNS involvement also responded to this therapy (40). Our case has many commonalities; in particular, CNS mucormycosis was well controlled, suggesting that ICIs may increase the sensitivity of patients to antifungal agents by regulating the immune environment in the CNS. Furthermore, our case is the first case to report the therapeutic effect of ICIs against mucormycosis in patients with lymphoma, which added valuable evidence for ICIs as salvage therapy for intractable mucormycosis.

**Table 1:** Published case reports applying ICIs as salvage therapy for mucormycosis.

Ref.	Characteristics of patients	Site of infection	Species of fungi	Initial treatment methods	Expression on CD4+ and CD8+ T lymphocytes	Timing (diagnosis of mucormycosis is DAY 0)	Frequency and dose	Outcome
David Grimaldi et al. (37), 2017	A previously healthy 30-year-old woman	Stomach, spleen, peritoneal vascular structures	Mucorales fungi, the specific species is unknown	Gastrectomy and splenectomy; L-AmB and POSA	Increased expression of PD-1	Nivolumab from DAY 12; IFN-γ from DAY 10	A single 250 mg dose of nivolumab; IFN-γ 100 μg thrice weekly for 5 doses	Recovered several weeks later, no residual infection
Jan Christoph Banck et al. (38), 2020	A 51-year-old woman with relapsed AML after allo-HSCT	Paranasal sinus, spread to orbits	*Lichtheimia ramosa* and *Aspergillus fumigatus*	Surgery; L-AmB and ISA	Increased expression of PD-1, CTLA-4, CD39, LAG-3 and PDL1	DAY 12	Nivolumab 240 mg every 2 weeks for 4 doses; IFN-γ 100 μg thrice weekly for 10 doses	Olfaction and laboratory markers were restored, partial response detected by CT scan. However, died from progression of AML 2 month later
Anne-Claire Lukaszewicz et al. (39), 2022	A previously healthy 38-year-old woman	Wide range of left chest wall, spread to pleura	*Lichtheimia ramosa*, *Rhizopus arrhizus*, *Candida albicans*, *Aspergillus fumigatus*, *Aspergillus terreus*	Repeated surgeries; L-AmB and POSA replaced by ISA; HBOT	High expression of PD-1	Nivolumab from DAY 27 approximately; IFN-γ from DAY 11	A single 280 mg dose of nivolumab; IFN-γ 100 μg daily for 14 doses	Improved rapidly, recovered
Alexandra Serris et al. (40), 2022	A 56-year-old man with diabetes and severe COVID-19	Brain, ethmoidal sinus, skull bones	*Rhizopus*	Surgery; L-AmB and ISA	high PD-1 expression	DAY 15	Nivolumab 240 mg every 4 weeks for 2 doses; IFN-γ 100 μg thrice weekly for 12 doses	Cerebral abscesses reduced, Mucorales PCR turned negative, but died from septic shock following pneumonia
Rania Mhenni et al. (41), 2024	A 68-year-old man with diabetes and adenocarcinoma, treated with chemotherapy and colectomy	Anastomotic stoma of small bowel, peritoneum, skin	*Rhizopus microsporus*	Repeated surgery; L-AmB, ISA and CAS	High expression of PD-1	Nivolumab from DAY 19; IFN-γ from DAY 14	A single 240 mg dose of nivolumab; IFN-γ 100 μg thrice weekly for 5 doses	Alleviation of mucormycosis, successful treatment

L-AmB, liposomal amphotericin-B; POSA, posaconazole; ISA, isavuconazole; CAS, caspofungin; IFN-γ, interferon-γ; CTLA-4, cytotoxic T-lymphocyte-associated protein 4; LAG-3, lymphocyte-activation gene 3; HBOT, hyperbaric oxygen therapy.

The success of these cases and our case relied on the combination of ICIs and aggressive antifungal agents with (other cases) or without (our case) extensive surgery. Antifungal agents directly target and kill the pathogen, whereas ICIs work by reversing T-cell exhaustion and enhancing host immunity, thereby improving the ability of T-cells to clear the fungi. Research suggests that the three main classes of modern antifungal agents—L-AmB, azoles, and echinocandins—have potential synergistic effects with ICIs (42–44). Therefore, ICIs may have promising therapeutic prospects for lymphoma patients with IFDs.

However, our case study has several limitations. We should assess the patient’s immune status (e.g., PD-1 expression levels on CD4+ and CD8+ T cells) before initiating anti-PD-1 treatment and regularly monitor immune status to provide stronger evidence for the efficacy of ICIs in treating mucormycosis. Furthermore, the toxicity of ICIs should be considered. Early ICI therapy may lead to considerable toxicity, especially in the context of high fungal burden and inflammation, particularly when it is administered at a full dose (34, 45). Although no significant toxicity occurred in our patient, given the risk of additive immunotoxicity when ICIs are used in combination with other immunomodulators, the timing, dosage, and rapid identification of patients who may benefit from ICI therapy warrant further exploration and research, especially in immunocompromised patients. Additionally, there may be a risk of publication bias, and larger case series or clinical trials are needed to evaluate the clinical benefits of ICIs in treatment of fungal infections.

## 4 Conclusion

Invasive mucormycosis in patients with hematological malignancies poses a significant challenge for clinicians. Our case report demonstrates that PD-1 inhibitors may have dual therapeutic efficacy in lymphoma patients with widespread disseminated mucormycosis by reversing T-cell exhaustion and enhancing host immunity. However, further studies are necessary to confirm the clinical benefits of ICIs in fungal infections.

## Data Availability

The datasets presented in this study can be found in online repositories. The names of the repository/repositories and accession number(s) can be found in the article/Supplementary material.
