# Supplementary material for: Case Report: Immune checkpoint inhibitor exhibits dual benefits for a refractory lymphoma patient with disseminated mucormycosis
Source: Front Med (Lausanne). 2025 Jul 2;12:1608828. doi: 10.3389/fmed.2025.1608828 (PMC12263650; doi:10.3389/fmed.2025.1608828)
Supplement: Supplementary file 1 [file Table_1.docx]

Plasma sample: Sample Processing and DNA Extraction

Volume of 3 mL of blood were drawn from patients, placed in blood collection tube and stored at room temperature for 3-5 minutes before plasma separation and centrifuged at 4,000 rpm for 10 min at 4℃ within 8 h of collection. Plasma samples were transferred to new sterile tubes.DNA was extracted from 300 uL of plasma using the TIANamp Micro DNA Kit (DP316, TIANGEN BIOTECH ,Beijing, China) following the manufacturer’s operational manual. The extracted DNA specimens were used for the construction of DNA libraries[1].

**Respiratory and pleural fluid samples：Sample Processing and DNA Extraction**

1.5-3mL sputum/BALF/pleural fluid and other samples from patient was collected according to standard procedures. Saponin was added to 0.45mL sample at a final concentration of 0.025%. Then the sample was fully vortexed for 15s and incubated for 5 min at 25 ℃. 75 μL was added for dehosting process. The sample was fully vortexed for 15s and incubated at 37 °C for 10 min. Then the sample was centrifuged at 18,000 g for 5 min and ~70-80μL were remained at the bottom after removal of 450μL supernatant. 800μL PBS was added to the tube and fully vortexed. After centrifugation at 18,000g for 5 minutes, 800μL supernatant was discarded and ~70-80μL were remained at the bottom. Add 370μL TE-buffer to the tube, followed by shaking. Then 7.2μL lysozyme was added for wall-breaking reaction. 250μL 0.5mm glass bead were attached to a horizontal platform on a vortex mixer and agitated vigorously at 2800-3200 rpm for 30 min. 0.3mL sample was separated into a new 1.5mL microcentrifuge tube and DNA was extracted using the TIANamp Micro DNA Kit (DP316, TIANGEN BIOTECH) according to the manufacturer’s recommendation.

**Body fluid sample: Sample Processing and DNA Extraction**

1.5-3mL CSF and other Body fluid samples from patient was collected according to standard procedures. 1.5mL microcentrifuge tube with 0.6mL sample and 250μL 0.5mm glass bead were attached to a horizontal platform on a vortex mixer and agitated vigorously at 2800-3200 rpm for 30 min. Then 7.2μL lysozyme was added for wall-breaking reaction. 0.3mL sample was separated into a new 1.5mL microcentrifuge tube and DNA was extracted using the TIANamp Micro DNA Kit (DP316, TIANGEN BIOTECH) according to the manufacturer’s recommendation.

**Tissue samples: Sample Processing and DNA Extraction**

Tissue blocks the size of soybeans were collected according to the standard sample collection procedure. The tissue blocks, 600μL of lysis buffer and 250μL 0.5mm glass beads were attached to a horizontal platform on a vortex mixer and agitated vigorously at 2800-3200 rpm for 30 min. Then 7.2μL lysozyme was added for wall-breaking reaction. 0.3mL sample was separated into a new 1.5mL microcentrifuge tube and DNA was extracted using the TIANamp Micro DNA Kit (DP316, TIANGEN BIOTECH) according to the manufacturer’s recommendation.

**Construction of DNA libraries and Sequencing**

Then, DNA libraries were constructed through DNA-fragmentation, end-repair, adapter-ligation and PCR amplification. Agilent 2100 was used for quality control of the DNA libraries. Quality qualified libraries were pooled, DNA Nanoball (DNB) was made and sequenced by BGISEQ-50 /MGISEQ-2000 platform. [2]

**Bioinformatic analysis**

High-quality sequencing data were generated by removing low-quality reads, followed by computational substraction of human host sequences mapped to the human reference genome (hg19) using Burrows-Wheeler Alignment [3]. The remaining data by removal of low-complexity reads were classified by simultaneously aligning to Pathogens metagenomics Database (PMDB), consisting of bacteria, fungi, viruses and parasites. The classification reference databases were downloaded from NCBI (ftp://ftp.ncbi.nlm.nih.gov/genomes/).

参考文献：

[1]. Long Y. ,Zhang Y.X.,et al.,Diagnosis of Sepsis with Cell-free DNA by Next-Generation Sequencing Technology in ICU Patients. Archives of Medical Research 47 (2016) 365e371

[2]. Jeon, Y.J., et al., The feasibility study of non-invasive fetal trisomy 18 and 21 detection with semiconductor sequencing platform. PLoS One, 2014. 9(10): p. e110240.

[3]. Li, H. and R. Durbin, Fast and accurate short read alignment with Burrows-Wheeler transform. Bioinformatics, 2009. 25(14): p. 1754-60.
