# Supplementary material for: Case Report: Immune checkpoint inhibitor exhibits dual benefits for a refractory lymphoma patient with disseminated mucormycosis
Source: Front Med (Lausanne). 2025 Jul 2;12:1608828. doi: 10.3389/fmed.2025.1608828 (PMC12263650; doi:10.3389/fmed.2025.1608828)
Supplement: Supplementary file 2 [file Table_2.docx]

>V300080312L3C004R0600815895

CCCTATTCCACAGCACTCCACAGGTTTGGCAATCCAGGATGCC

>V300080312L3C004R0570617673

AGCTCGCAGCATTACAGGCACAAACTTGTCCAATTTATACTGG

>V300080312L3C004R0190988642

ATTGCTTGGGAACCTTGTATTTGTGTTCGATACAGGTAAGCGT

>V300080312L3C005R0041208237

CTTCTGTGAGTTTTCCTTCAACTCTTTTAGCCTTCTTTAATGC

>V300080312L3C006R0720775052

AGTTTTTCTTCAACTCTTTTAGCCTTCTTTAATGCTACAATCA

>V300080312L3C005R0400064803

GAGAATAAGGGTATGGAGGGCTGATTCATGGATGGTTATTGTT

>V300080312L3C006R0431267374

CACTGACAAAGATTAGTTGAATCGTATTTAGACCAACATATCA

>V300080312L3C001R0591321891

GCACGGACGATTGTTAAGGAAGTAGCGAGATAAAATGTATGTG

>V300080312L3C004R0520062835

GGTTCCTTAAGCTGCTTACCTGCATTAAGCAAACCTTTGCTGC

>V300080312L3C001R0550831175

GCCATCTTTCGAGAGGATCCTCGCGGTAGATAAAAGGGAAAAC

>V300080312L3C001R0590803794

CGCGGTAGATAAAAGGGAAAACGCCGCGGTGCAGATGTGATTG

>V300080312L3C001R0100915173

CATTTTCATTCAAGACGGGAGAGCATATTTTGTTGATGTACCA

>V300080312L3C006R0080108888

AAGTCATCTCCAAAGCCAGTTCCGAGTGTGCGATCGCCTCCAG

>V300080312L3C002R0700611949

GATCATGGATTGCCTTGTCGGCCAGATTCCTAAACTTCAATCC

>V300080312L3C002R0510902587

CTGTGTTTCGATCACTCTGAAGTTGGCTCCCAAATCGCCCATA

>V300080312L3C005R0360002474

TAAGCATTGCTTTCCATTTTTGGATGATGACGAAAAGAAGGGT

>V300080312L3C004R0410403193

ATTCTGATCGAAACAACAACTCCATTTTGCGCCGGGAATTTTA

>V300080312L3C002R0570845235

TCGCCGGCGCTGCTGCTCCTTGTCGGCTTTTGCCTCGGTGTCC

>V300080312L3C004R0060849451

TCCGAGACGACTGCGCTATTGCTACTATATACAAACCTATCGC

>V300080312L3C005R0410074507

GAATTTTGGCCTCATGATATGCCCGGTACGCGGTATCCAACTG

>V300080312L3C006R0330563783

CATGTCGACGAGAGTTGCATAAGAAAAGAGAAATGGAATTCAG

>V300080312L3C004R0010783549

TTGATTACCAGGTATGCATATCTGTGGACATTTTTCTTTCTCT

>V300080312L3C004R0500592675

CCATTGTCTCTCGAAAACGCAAGCGGCGTCTTGTCTTGAGTTC

>V300080312L3C002R0411170647

GACGAATTGAAATGTAAGACTGAAACCTGTATTCAGGAGCGTG

>V300080312L3C004R0640027071

CGACTTCCGACAATTGGATGAACGAGATCAAGCATTGGGCACC

>V300080312L3C004R0610031531

GAAAAATCGCTGCCGAACAAGACTGAGCGAATTCTACGCGTGG

>V300080312L3C002R0601356412

TGCTGGTCGGTGTCGATCGACATGGATTTGGAAACTGGGCCAA

>V300080312L3C006R0570752965

AACACCGTCGCAAAGTGCTGCATCACCTGCACGTCTGAATGCA

>V300080312L3C004R0260116723

CGCGAACTTGCACGACAAATTCAAATGGTCATTGAACGTATGG

>V300080312L3C004R0360600615

GAACGAGATTTCTCTTCGCAGAGAAGAATTGAGCGTGGATTCT

>V300080312L3C004R0570824538

CAGGTGTTTCAATCAATTTCGTTCACGATCGCCGTTCCTGGGA

>V300080312L3C004R0340786467

CCCCGTCATGCCAGGTAACGTCATAGAACCAAAAAAGGGCAGC

>V300080312L3C005R0540081025

ACTGACTGAATTATGCCAATAAATCTCTGTACTCTCACACAAA

>V300080312L3C004R0591219600

CAGCAACGTCGTTTCCTGATCCTCGGCCAGGATGCGAGAAAGT

>V300080312L3C004R0620687457

TGATCAGATTTCCGTATCAGCGCATTTGCGAGATTAGGAAAAG

>V300080312L3C001R0520575129

CCAAGCAAGATCCGAGTGGACAACTGGCTAAAACCATCCTCCG

>V300080312L3C003R0550651323

TGTAGCTGCTCTTTATCTGCAGCAGAGAGACGTGCCAGCGACA

>V300080312L3C003R0040656990

GCTTCAATGAAAAGCGCTCGTTCGTGCTCGATTTCCTCCCGCG

>V300080312L3C005R0510830140

GATAGTCTGTTTCTTCCCTGCTCCAATCCGGATCTGTAGGAAA

>V300080312L3C001R0590011496

CGAGAGGTCGTAATTTTGTAACTTACCACTGTCCATGTTCACG

>V300080312L3C004R0310365272

TCTCGCAGATCTCGTACAAGGTTGAGCCACGTCGCAAAGATAT

>V300080312L3C002R0380222583

ACGTCGCAAAGATATAGAAGAGGAAGCGGTCTATGAAGTTCCA

>V300080312L3C004R0180104207

AGATGAACTCTAAGCTGCTTACGTGATATTTGCAAAGAGAAAT

>V300080312L3C002R0340285656

GTTAATCTTCAATAAAATTGATCTAGCAGAGCGTGGACATTCT

>V300080312L3C006R0120097087

AGCATGCTACCCAAGGTGGCCTGTGCCTTCATGATCTATAATA

>V300080312L3C004R0110791025

ACGCTGGTAATGGGCACGAAAGTGTTATATATAAACTTACCTC

>V300080312L3C006R0550891340

GCACGAGTGCGTCGTTGCGCTTCTCGGTCGCATCAGCGAATGG

>V300080312L3C003R0580678904

GCTTGCACAGTTCAAGTTTGACAAGCTGAAGAAAGAGCAACGT

>V300080312L3C006R0301358787

CATGCTTGCCTTTACGTCGTTGATCCAACGAAGCGCGCGTTGG

>V300080312L3C006R0100038057

GCGTTGGATGATTACGATATCCGTATTTTGACCCGTTTGTCCC

>V300080312L3C005R0090875855

CCACCACCAACGCCGGCAAAAATTCGACTGCTGGTGCGTCGCT

>V300080312L3C002R0020804729

GCACATCATTCCGTTTTCCGTGATTGCGTACGAAGACGATCCA

>V300080312L3C004R0331078386

TTCCGCATTTTGTGTTGTCAACTGTTTGAAACTTTGAAATGAT

>V300080312L3C002R0200765891

TTCATCCGGTCCCTGAGTTCATTGATGGCCGATATATCGTAGG

>V300080312L3C005R0561320647

ATCCACCGTGTTGTTTCTGTTGCGCTTTGCGTCATCACAATCA

>V300080312L3C001R0280400471

ATTGTTGAGCGGAAAGGAAAAGGATTGACCACAGTTGGATTGT

>V300080312L3C003R0551187804

AAACAAATAAACTTGGCACAGACTCGACACCAACAATATGCAT

>V300080312L3C005R0240865863

AAGTTTGCAGTTCCACCACCATCCAGATTAGGCCAGCATATTT

>V300080312L3C003R0590262830

AGCATCAGTAGTCTGGTCAGCTGAGACAGGAATGTAAAATTTA

>V300080312L3C004R0170613781

CTCGTACGTCCACGAACCCGAAAACGTCGGCGTCTTAAGTCTC

>V300080312L3C005R0650065017

GATACGCAGCAATTTGCAATGCGGGTTTTGCTCGACATGGATA

>V300080312L3C006R0691373702

GGATCAGAATAGTCGCGACCGTGGCAATTAAAGCACTCATATC

>V300080312L3C002R0081184517

TACAATCGCAGAGGTATCATTAAACATCCGTCGGCATTCTTCG

>V300080312L3C006R0321232896

GGTTATCCTTGATAGACATTCTGTCAAATGAATCCTCAAACTG

>V300080312L3C002R0200645916

CAGGATCATTGTCACGACGAGGGTTTTCAACTTCCAGGTCGTA

>V300080312L3C006R0720423726

GCAAAAAGAATATTTACTAATGGTTTTCTCAAGATTGGTTTGC

>V300080312L3C002R0480571034

AATTGATCGGATAGCATAGAGTTTAAAGAAACAAAGAAGGGAA

>V300080312L3C004R0190648786

GTTTGCTCCTTGCTTCACAGCATCGCCCGCTCGGTGTGCCGCT

>V300080312L3C006R0320542069

ATATCATTTCTCTGCTGCCTATAGGACCTATCGAGTTCCATGA

>V300080312L3C003R0271401728

GGAATGACCGCTGTGTGGTTGCCAGAGGGCATCCAAGTCGGCC

>V300080312L3C001R0460406002

ATTCGAAACACCTAAGCTATTCAATCATGAAACAGCTTCTACG

>V300080312L3C005R0391382397

GTAACGATAACAGCAGTAATACTCGAGTCGTTGTAATAACTTG

>V300080312L3C003R0590293910

CGCAGTAATACTCGAGTCGTTGTAATAACTTGTGCATTTGCTC

>V300080312L3C006R0541039821

TACAATTCTGCTGGTCGGTGTTTTTATTTCTGCGTATTCGATC

>V300080312L3C005R0650525364

CTGCATGTACCCACATTGAAAAGTACCAATCCGTAAATGAGGA

>V300080312L3C005R0390184676

ACATTGATCAGTACCTTGATATGCTTTGGGTCTTCCGCGGCTC

>V300080312L3C001R0590635246

AAAGTTTTGAAACACAAAGTATAATCTCGCTTTCTCACGCTAT

>V300080312L3C004R0340002619

CTATGGTTGATTATGACTGTATGGATGTTGATCAATGTAAGGT

>V300080312L3C005R0290051667

GGGTGATGAGCACCCGGTCTCTGATCGATTAGAGCGCCACTGA

>V300080312L3C004R0400101031

GGGTCACTCACCAGCTTAATGCATAAACTGCCACGAACAAGCA

>V300080312L3C004R0570637199

CGATGGTTAATATCCGCTTACGGTCCCACCGATCGATCTAAGC

>V300080312L3C005R0411338342

ACAATATATTAAGTTCCGAGTTCTGCTTATATACTAAGAGATC

>V300080312L3C006R0631075887

ACAATGAGTGCTCCAAAGCGGTTTCCGACGAGTTGGATGATGC

>V300080312L3C006R0640155640

CAAGGTAAGAATGAGCCATCTTATGCTACGAAATATTGCGTGC

>V300080312L3C002R0520734767

CGCTCTTTTTTGCTTCAGCTTGTGTTGCGCGACGCTTTTGCCA

>V300080312L3C004R0310144869

CTCCGGAAACGAGAGCTTCCCGGACGCATTCCTCAAACGTGCC

>V300080312L3C002R0610509062

ACGCTTATTGGTAAGAATGAAATCCAAATCTGCCGCGTCCTGC

>V300080312L3C003R0190533375

GGAACCTTCTCAGTTAACGTGCCCCGAATCTGCACCCTCATCC

>V300080312L3C003R0550004380

CTTTTCGTGTTTTCTTTTTTCCTTACGTTGCACGCATATCATT

>V300080312L3C004R0630572803

GCGGCACCCAATGGTGATAGGCACAGCAAATACTACAGTCGTC

>V300080312L3C005R0700734542

AATACAAAGAACGAAGAAGACCAAAGCAAACAAACAACACTTC

>V300080312L3C001R0690512530

CCATAGGAAGAAGATCATTGGCACTTGCCATGCTCTCGGATGG

>V300080312L3C005R0571391094

TCTTTTTGGTTGGGTCGGCCAGGCGACTGAAAGCTGTTGCATT

>V300080312L3C003R0571243862

GACAGTTACGTAAGAAACCCCTCTGTCTTTATACAACTATCAA

>V300080312L3C005R0150596939

GTAACGAAAAAGCAGACCCAGCAGCTTCGTTCATATTAAGATA

>V300080312L3C005R0520537793

GTAAGAATACGTAGGGAATAGGGTACACTTATAACACTGCCGT

>V300080312L3C003R0561092698

CTTACAACAACATGATGAGTCTTGTGCTTGATTTAGGAAAGGA

>V300080312L3C004R0421283932

CACAATTTATTATTACTGGGTCAAAAGTATACACACGAATCCA

>V300080312L3C003R0221195207

TTTAGCCGAGTCCACTTTACCACAACTAGTAGTTTCGACTAGT

>V300080312L3C004R0570609642

GCTTGTTCCAGAGCGCGGCAACAACAACAAAGCATTGCACAAA

>V300080312L3C005R0340603233

TATAATCATTGCTTTGTAATCACGATATACTACGCATGTCAAG

>V300080312L3C001R0610269467

CTCGATAGTAATCATAATCGGGTGCACCGCCAACCTGGCTGCC

>V300080312L3C001R0211353386

CATCTCTAGCTCCAATGGTGTCATTTCGCCATCAAAGTGGACC

>V300080312L3C004R0590934052

TGCTTGCCATGTCCGTCAGCGAAGTAATCTGAAGTTGGTCACC

>V300080312L3C003R0471145476

TCAAGTCTGGAAGCAAGAATGCCCTGATTTGCGATTTAACACG

>V300080312L3C003R0310004309

CAGATTGTTCTAAAAATGGCAGCTTTCCCTTCATTGCTATGTC

>V300080312L3C003R0630443532

GCGCACAGCGTCATCAATGATAGACTACTCTGCATTTCTGTAG

>V300080312L3C005R0210818704

ATTTTCCTGGATGAATAGGCTGCGATAGGAAAACTCGACAGTA

>V300080312L3C005R0010464663

CTATATATTTTGAAAGATGCATAGTCAATAACGCAGGATACAA

>V300080312L3C001R0420393606

TACTGACGAATTTGGTACGATAGCGTTAAGCTCAAGGCATTCT

>V300080312L3C004R0531235419

AGGACAGAAAGAAGGAAAAGCTTATTGTTGGCGATCCTAAGCT

>V300080312L3C001R0460130590

GCTTGTTGCCTCACATGATGTAATTTTCAGCTGCATTCTCTTG

>V300080312L3C001R0111121755

ACCCAACTCTTCTTTTTGTTGATAGCCTTGGCATGAATTTGGA

>V300080312L3C006R0260849247

TAGTTTGAGACGTAACCACGATGAACAAGACTTGGGAGATGGG

>V300080312L3C006R0131050176

TTTGCCTGACGACTCTGACGATAAACTGGCACTACCACCATCA

>V300080312L3C005R0221180593

GATCAAGTTATCGATATTCTTAAGCAATTTTCTTACCTCGACC

>V300080312L3C005R0540002225

CTCGGTTGTCGTGGGAGACGCAGTTAATGTATCGGCGAGGATG

>V300080312L3C005R0440381529

TCCCCGCAACAGCAACAATTATTGTTAGCGAGCAACGTCAAGC

>V300080312L3C004R0050972427

GCGATCGCATTGTTTACGCTGATTTTGGTCGTCGCGGTGGGAG

>V300080312L3C003R0471036455

ACAGAGAAAAACAAAGTAATACAATGGTCCTCTTCATTTCGGA

>V300080312L3C006R0190330764

AGACGTTAGTGTTAGTAAAGCACTGTGCTTATGAGCGAGAATA

>V300080312L3C003R0160261752

TGAAGTCCAAGCAACCCGAAACATGGCATGAAGCCCAATTCTA

>V300080312L3C002R0530435566

CAGCTGACATGATGAAAAGATTAAACGAGAGACTAAGTAAATG

>V300080312L3C002R0311070316

TAACCTTTTTGCGCCAAATCTCTTGTTTAGACACCAGCATTTC

>V300080312L3C006R0270594312

TGCGCCAAATCTCTTGTTTAGACACCAGCATTTCGGCTGAACT

>V300080312L3C004R0660097432

GCCAACTGAACAATCATTCATATTACTTACGCAAAAAGCATTC

>V300080312L3C002R0310442219

CCAATGGTCCCAATAAGCAAACGTATAACAACCTTACCGGGGA

>V300080312L3C006R0411283990

AGGACACTTCATACTTCGATCTAGCATAGCGTTGACTTTGGTG

>V300080312L3C001R0330153053

AGAAGGCAGTGTTAACAAGTAATGTGCTCATTATGTGCTTATG

>V300080312L3C002R0020205752

GGACCGGTTCATTCAGATTGACCACAACTATTGGATGCCATTT

>V300080312L3C001R0301183722

AGAAGCAAGAGGAGGAGCAGGCAAAGAAGGCTGAAGAAGAAGC

>V300080312L3C002R0251080644

GGCTGAAGAAGAAGCCAAGGCTCTTGAACACGCAAAGTCTATT

>V300080312L3C001R0671126460

TCATCGTCTTCGCGTCCAAGGCAAGGATATGATGTTTGTCGTC

>V300080312L3C001R0091115062

TGTTTGGCCGAGTCTTACCGTGCAGAGAAATCACATACCCGCA

>V300080312L3C001R0471259289

TCCTGATACCCCGATTATCCAACAATACCTGAGAATGGAATTG

>V300080312L3C006R0490981137

ATACCTGAGAATGGAATTGTTCTAAAGGGTAAGGATTCAGAGC

>V300080312L3C006R0330692448

CTGATACGTTGTCACTGCAAATCTTCAGGAACAAGAAACTCAA

>V300080312L3C003R0490546102

TTGCCTTGAAGAGTTCAAAAATTGAACAGAAATAAAAGAGAAG

>V300080312L3C002R0170155810

TGCTTGAAATCCGGGTTCCCGCTTGTCCGCTGACGAATGTCGA

>V300080312L3C003R0400435688

CCGTACGCTGTTTTCCTCGATCTTTCTGATCGATCGCAATTGG

>V300080312L3C006R0100235785

TGCTTTCATTGACTAGCATGAATTAACATTCTAAAGATATGCG

>V300080312L3C002R0181090862

TTATTGGGATCAAGTTCTCATCCTGACAAACGTCAAGGTCTGT

>V300080312L3C001R0050585519

GAGTTTGCAGCCTCGCTTTTGATTTTAAATGAAGGTTACGATG

>V300080312L3C004R0700141719

GCTTTTCTTATAAACAATTACGACGTGATTGCTTCCGTTTTCC

>V300080312L3C004R0091285720

AAGCGCACAGTCGAGCAGTGGAAGGTGAACTTGAGCATGTTAG

>V300080312L3C006R0261101424

TAGTTGCTTAGTTTCTAGAATGTTTCACAAATATTACGACTTA

>V300080312L3C005R0421302001

TGGACACCAATATCGAAATCGGTGACTTTAACTGCACTCTGAA

>V300080312L3C001R0720849705

GCCAATCCACCTATATACATCGCTCCAGTGTCAATTCGTGCTC

>V300080312L3C003R0481324084

CGACATTGGCGTCTTGCTGCTTTTGCATTTCGTTGCTGTTTGC

>V300080312L3C006R0540555892

GCATTTCGTTGCTGTTTGCGGCGTCGCCGTTGTTCTTGTCCCC

>V300080312L3C003R0351319880

CATAACATGCAGCCTACATAGAATAATATTAAAGATTTTTTTC

>V300080312L3C003R0290313304

ATATAGATATGCATCAATAAATACGGCTTGCTTGATTCTATTA

>V300080312L3C005R0580399938

CGTAGATATAGATGTTAATGATGAAATTAACTTATTGTCTCCC

>V300080312L3C003R0350926185

CTTTGCACTTGGTGATTGCTCTAATTTACCTACCTCTAAGACT

>V300080312L3C001R0310752949

TCTTTCTTCACATCCTCATTTTGTTTACGGTGTCTATCTTAAA

>V300080312L3C004R0010599085

CTCTCCGCGGAAAATACCCAATGCACGTTCCTGCAAAAATATA

>V300080312L3C006R0060085293

GATATACATGGCAATGATGTAGACCAACTGTGTGCCCGGATTG

>V300080312L3C003R0100700059

AAACTGATGAACGAATGAAGATATCTCATGCGGTTTCGATAGC

>V300080312L3C006R0140436904

GGTACGTGCTGAGAGCTGACATTGCGATCGTGTTCAAACCCGT

>V300080312L3C005R0430803895

AAGATGGAAATCGAGGAAATGAAAAATTGCGCGTTTCATAGTT

>V300080312L3C006R0710113871

TGTTTTAATTTCGTCACCACCATTTCGTATGCAGTGTTCAACG

>V300080312L3C003R0350142366

CCTTTTCGGTCTGACCATTTCCTTTGGCGCGATTGACAAGATC

>V300080312L3C004R0030200625

ATCTCCTGTTATCAAAACACGAAATTTGAGCTTCCAACATAGT

>V300080312L3C002R0620198435

AGCCTCCCGTCCGAGATAAGCATCTAGCAAGTTCTTGTTCAAT

>V300080312L3C001R0361194839

TGAGAGTTATATATTTAGCCTCTTACATAGGTAGTCTCGAAAC

>V300080312L3C005R0030019905

TCGAATGCTTTCCCAGAAATTATAATCAGTCACAGCTGAGCAT

>V300080312L3C004R0190121917

GTCGGCGTGCTGCATAGCCAAGTTAGCATAGCAGGAACCATGC

>V300080312L3C006R0131222709

GTCGCTTTGCACGGTTAATAAGGTCAGCAGAACGCATCAGAAG

>V300080312L3C005R0071243421

TATTCTGTGCTTACAAGTTGTTTTTATTTTCTGCAATAGATTG

>V300080312L3C003R0580471942

CCATGGCGCTCCTCCTCGAGCAAGTGTGCGTACAAAAAAGATG

>V300080312L3C005R0680171156

CCGCAAGGTCCTTGACAGTGGTGTACGTTCCCGTGATCTAGGC

>V300080312L3C006R0631353805

CATCACCATCAACACCATCATCATCACCAAGGCGGTCACGGCG

>V300080312L3C001R0690098980

CAATCATAACATTTGCAGCTTCCCAAGTTTTCCCGCCACAGCC

>V300080312L3C005R0270319271

CGGATCTGGTGGACTCGGAGGTTCAGAGTCAGGTTTTGGTGGT

>V300080312L3C002R0570337968

CCTCATATTATCGTGCACTGTACAATGTACCATATGAGGCCGA

>V300080312L3C006R0061011055

TATTATCGTGCACTGTACAATGTACCATATGAGGCCGAACAAA

>V300080312L3C001R0550456293

ATTGTTTTTCTTTGCAGACTGATCAATAACATATCTTACATAC

>V300080312L3C005R0711204797

AGTCATCTTACCTCGACATCTCCATGGGGGACAGCCCGCTCGA

>V300080312L3C002R0390549018

GCAGTCCTTTATTTTTTGCTTACATTGTTTGCGCTATATTTTC

>V300080312L3C002R0270486296

TGATGATGGCAGTAGCTGCGACGTCAACAGCGAATTGTTTTCC

>V300080312L3C006R0241333615

TTTTAGCAAAGTATAATGTTTAGTCTCACTCTACAAGACTTTT

>V300080312L3C002R0131362862

TGTTGCAAACTTGGAATCACTCATAGCGCATGTCAAACAAGTT

>V300080312L3C006R0361254272

CTCATAGTTTTCCAATTTCATATCATTCAATCCCAACGCTTCG

>V300080312L3C006R0411293695

AAAGTGGGGGATATCCATGACGTGCATGTTGGTTTCACTGGGC

>V300080312L3C001R0080760748

GGCATTTCGTACGTACCATCGCACGTTCAATTTGGGGTCCTTG

>V300080312L3C005R0240018921

TACCTTCGATCGTAGTCAGCAAACCACGCCGCCGGTTGGCAGG

>V300080312L3C005R0540140008

CACGCCGCCGGTTGGCAGGAATTTCGAGTTCAAGTTCTTCAAG

>V300080312L3C001R0580725961

CCATAGAAATGATGCGGTACTGTTGTTACAGAAGCCTGTGATG

>V300080312L3C002R0681345975

AAAGCTCAGCAAGAGATGGGTAGAAGATGAGGTACGGTTCTTA

>V300080312L3C002R0010266035

ACGACGGCAAAGGAACAGACTAACACCGATTATGAAGATAACG

>V300080312L3C005R0561061049

CAGATCTTTGGCCCGAATGGCAATATCAAGATCAAATCATGGG

>V300080312L3C005R0441274020

ACGCCTTAACCTTCATGTTCCCGTTTGTACCATATTAATGACA

>V300080312L3C005R0570784865

CTTTTTCCGTTTATTGATTTAAAAACAATGCTGGAGGATGCTG

>V300080312L3C004R0541093805

TAGCAAATCCGACGCGGATGATGATTTAGCATTGATGCAACAA

>V300080312L3C005R0610282555

TGAAAGCTGGCAAATGTTGATGGCTGGACGCGCTCTCCACTTT

>V300080312L3C001R0541397760

CAGCAGCAGATGCTTGAGGGGTACCATATCCTTCCTGACTATT

>V300080312L3C001R0320854677

TACGTTGGTTGAAGACGCATTGCCTAATATTTGTGTTAGATGC

>V300080312L3C004R0671128956

GAAACATTACAAGAATCTTTACCTGTGATAGAATTTGTGAAAC

>V300080312L3C004R0540576339

GGCGAATTCTGCAAGTTAAAAACAGTTAATAATATGAACTTCC

>V300080312L3C004R0110162817

TCGCAAGTTTTGTAACCACCTGCTTCCGTGTCTCTTTTTTATT

>V300080312L3C003R0590194742

GCGTATTGTATAAACTGATCGCCAAAGATTAAGTGCACTTACA

>V300080312L3C001R0660054146

GCTGTTCGATCTCGCGAACAATCTGTGCAGCGGCATGCTGATT

>V300080312L3C003R0100477457

AAGTTTGATATTGACGCATTTCACAAACCTGTCTAGAAATGTG

>V300080312L3C003R0260955430

ACTCAAATATTGAAGCGTTTGGAACCTTGGCACATTCAACTGA

>V300080312L3C002R0490181565

GGTGGATAATGTGGAGGAGAGAAAGATTTAAGGTTAACGGCGC

>V300080312L3C006R0130242499

ATAGATGCAGACAAAAGCATAGTACACCAATATGGAAAGCGCG

>V300080312L3C003R0050691718

AGACGGTATCCAAGGCACAGCGTACGAAGACCTAAATAGTAGA

>V300080312L3C003R0050548888

TCAAAAAGCTGTGCCTGCTTTTCATACATCTCATCTTTGGCGG

>V300080312L3C002R0110124679

CGCTACAGTTTTTACAATTTCCACGGAGATATAGAGGGAGATA

>V300080312L3C005R0150337932

CTATACGGTGTACGTGCAGCTGGAGGTTTGAAAATACTGCTCA

>V300080312L3C004R0690075776

ACCATCCTTGATGGCAGTGATTACTAAGATGGCAATCAATGGT

>V300080312L3C006R0411327202

GGAAGGTTTGTTTTAATGCGATCTCTGAAAAAAAGTAGTTCTT

>V300080312L3C005R0621057497

TTGGTGATCGTTGCTAGCCGCAATCGTTGCCTGTATCGTTCCA

>V300080312L3C005R0570459504

CAATCGTTGCCTGTATCGTTCCAATGTCATGGCAAAAGAATGC

>V300080312L3C002R0440900215

ACGCTAGATCATTTGGGATGATTATTGGATGTTGTGTGTGAGG

>V300080312L3C005R0120434337

ATCATGTTGTTAACATACATGTACCACTAGTCGTTTCGCTCCT

>V300080312L3C005R0340931119

CCAGTGGGCGTCGGACTCATTGATGTTACGCATTGGCAGTATA

>V300080312L3C002R0400038541

GCATAGTTATTGCTCTTCAGGTACATTTTACTCTGACGTATGC

>V300080312L3C001R0670745883

GCGTAGCTCTTCCAAAAGCATACCTGCGCAAAGAGATGAAGTC

>V300080312L3C005R0680984327

CTGGTTTAAGCTTCGTAAGACGCTGCAACAGAGAATCAAGCTA

>V300080312L3C005R0720774977

GAAGAATCCAATTTATCACATAAAAGAATATAAAATAAAGATG

>V300080312L3C006R0690055472

TCGACCTTAGTTTTTAGTCTCTTGACATTAGCTGGTGACAACC

>V300080312L3C003R0500076549

AATGGAGTCGAGATCGTAACTGGTTATTAGACCAAGTCTTGAT

>V300080312L3C006R0400577661

ACAGAAGGTCCAGGCAAAGTCTTCTCCGTCGAAACTGCATCTT

>V300080312L3C004R0220756621

AAGCGGGGTGTCCCAGCAATTGCCATAGATAAATACAAAGGTC

>V300080312L3C001R0041144110

TAGATAAATACAAAGGTCACTTGAACAAAGAAAGGATGGAGAT

>V300080312L3C003R0390945971

GAGTGTAAAAGAGCACTGCAGTGTATGTTTTTCAGTGCAGTTG

>V300080312L3C003R0520086369

TGGATATATGTCAAATTGTTCTATCCAGTACGCCAATCCTTAC

>V300080312L3C003R0121270790

TTTAAGACTTTCACGTGTCAATTTGTTCTATGACGGAGATAGG

>V300080312L3C004R0190422940

ACGATGTAGATTTACTGAGTTTCGACAGGAGCGGAATGCTTTG

>V300080312L3C003R0511148111

TCCAGGTAGGGTTAGCAGCTTCCTGTCCCAGATGCAAACTGCC

>V300080312L3C002R0620246593

TCCTCTCCGTAACCGAAACGCTGTCAGGATAAATATGCCGATT

>V300080312L3C002R0200509015

AATCCGCTTCGCTCATAAGTAACCGTCCGCACACCAGGTAAAG

>V300080312L3C006R0150577872

GGCAGCAGCACAGCAAACCCCAGTATTCCAGACGACTGCATGG

>V300080312L3C003R0220508328

GACAAAGTATTCGCCGGAAAGATTGTTTGGTGCGGCAAGAACA

>V300080312L3C002R0550896565

ACTGGCAGTATGGAGACGCCACAAATAGTTTGTGTTGACACCG

>V300080312L3C002R0451156507

ACGAGACACACTTTCGACAATCAAGTCGAAAGGAACAGACGAG

>V300080312L3C004R0580164068

TCAATGTGGAGACAGTGTCTATCAGAGTTGTGGAACAAGTCAC

>V300080312L3C001R0500058541

TCCTCGTCGTTGTATGTCTCCAATTCCTCAACGCAGAACATGG

>V300080312L3C006R0581088612

AAAAGTCCACGGCGTTGTCCGTAGGCTCGCCTTCGCCGTAGGT

>V300080312L3C005R0480013210

TAAAGACATTGCCTTCTTCATCTTCATATTCCTCCATTTGATC

>V300080312L3C006R0131167713

TGGTGATTTCGTGGAAGTGTCGTGTATTTGGAATGCCTAAGCA

>V300080312L3C004R0171275708

CAGTATTCAGGACACATTCGCAATACAAGTAGAATCACACTCA

>V300080312L3C001R0540910395

CTCGGTCTTTGAACTATTATTCTAAAAACGTCATTAGAACTTT

>V300080312L3C002R0240944575

CACCCAGAATTTGAACAGGTGTGTGTTGAATACGATGCATGGT

>V300080312L3C005R0430796409

TGATCCGATTTGTAGGGACTGTCTCAATTGGCGAAGACCTGCG

>V300080312L3C005R0480603649

TATACATTAGAGTTAATAAAATTCAATGCATGATCTAACCATA

>V300080312L3C005R0030061159

TGGACAATGATCTGTTATCGACACGGTGAGATTTAATCTTTCA

>V300080312L3C004R0341362549

GCTCGTTCCATTCGTCGTACAATCGTCGAGCAAGTGATCCTGG

>V300080312L3C006R0531127438

CCGTCCAGCCGCAATCCAACAATAATTTGACTTCATCAATTTC

>V300080312L3C005R0630869165

ATAGCTGGTCATGTAGAAGGTGGCGCAAGACAGGAAGAGTGGA

>V300080312L3C005R0291049480

CCTGCGGGTCCGCACGCGCGTTTCACAAATGGACTGACAAACG

>V300080312L3C004R0430043184

TTCTCGATAGCTTTGCTCATTGCTTCCCGCACATTTTTCCAGT

>V300080312L3C002R0150138090

TCGTCGTCGTCGTCAATTGAGGATGATCTTTGCATAAACAGTT

>V300080312L3C003R0260847080

GAGCTCAACGCTCCTTGGTTTGATTCTCGCTTGCTGTAGTTGA

>V300080312L3C002R0720429240

CGACTTTGACGTGGAGAGTGTTCCTTTTGGAAGAGAGGTCGTC

>V300080312L3C004R0400903241

CCACCATTTCCTTTTTCCTTGCACTTGTTTTTGAATCACGGCC

>V300080312L3C003R0210032907

AAACGGCGGCATTATTTTTTATTGACTTGCCTATTTTAGCTGG

>V300080312L3C004R0530482949

AGGATGTATTTCACGGACGTACAAACAGCTGAAATTCACTGTA

>V300080312L3C004R0640172661

CCAGAAGAGCGAGAAGTCACCAGCATTAACCAGTCACCAGTTA

>V300080312L3C001R0620909394

CCAGCAAGTCGATCAAATGCTCCCTTTATTCCATCATTATTCA

>V300080312L3C003R0120912629

GGAGGGCTGTCTTATTATTGTCTTTCATATCTCATTCTTCAGT

>V300080312L3C003R0280421850

TATTATTGTCTTTCATATCTCATTCTTCAGTTGATGCATACTT

>V300080312L3C002R0450185170

ACAGCGTCGACTCATGTAGAAACCTTAGATTGGCCTCGCAATT

>V300080312L3C006R0150007005

GGACGTTGGCTTACTTTCCGTCTCGAATTTGAACCTGACGGCA

>V300080312L3C002R0410834439

CCCGCATCCAACGCTTGAGATCAAGCTGAGCGATTTAGGCCTA

>V300080312L3C003R0150762580

CAAGGAACCGGCGCAAAAGACAGAACCCAAGACGGTCCGCGAA

>V300080312L3C001R0110608462

ACAGCAAGAAAGTAAGCTGGCACCTTCGAGGACATCATCGTTT

>V300080312L3C002R0241158946

TGGTGGGACCAAAGTTCAATAATGATACGATATTCTTGCCAAA

>V300080312L3C006R0090873832

CAGTAAGCAGGGACTACCGCAATCCAGCAAAATTTGCGTTCGA

>V300080312L3C005R0060002398

TGAACAGTTTACCTGGCGAAGAACGTGTCTTCATCGCGACGGA

>V300080312L3C002R0221353058

CGACGGATACGGGTCAAAAACTGCTGATGAAATCTTGCATTGC

>V300080312L3C002R0510626680

TATTCTACAGTTCTGAATGAATGGCCAACATGCTTATAACACA

>V300080312L3C004R0070278351

AGACAAGTGAGAAAATAAGGAATCAGCAACGTAGGAAATTAAC

>V300080312L3C001R0470634890

ACCATTTTCTTTAGCTGATTTCTCAGGACAGTTCGGCGAATCG

>V300080312L3C005R0610369803

CTCGTTAGGATAAGCAACAAGGTTGTACCAAAATGCACATGCT

>V300080312L3C001R0320420043

CAGCCGTCCTCGTCCTCGTCACCTTTCCGCTCTACCTCCACGG

>V300080312L3C001R0300655886

GTGACGAGTCTGACTCTGAGTTGATGAACGAGGATGACGGGGG

>V300080312L3C002R0270474293

GCAGCAGCAGCAACCGCAACCACCATCCTCGGCATCGACCACT

>V300080312L3C005R0110409211

TTGGGTTTCAGTCTATTCAACAAGCAACCCTTCGAACGCTTCC

>V300080312L3C002R0120281659

TACAAGCATGTCCACCACCTCCAGCTCCTACACCACCAACATC

>V300080312L3C002R0640360887

GATGCAAGTTGTTGAGCAAGGAATATGTGGGTCGGGGGGAATG

>V300080312L3C001R0621188685

ACTGGGTCTCGCAGAGCAACCAGATCTGGAATTACGAAAATTT

>V300080312L3C004R0320549979

ACATCATAGTCTTTTGTTGACGCAAGAAAAGTATGCGCTCAAT

>V300080312L3C001R0680447692

TCGACGTAGCATCTCAAAAAGTCTGCAGCAGCACGGCGGCGAG

>V300080312L3C006R0040213759

TGCAAAGAACTGTTTGCCTTACAGCATCGTATTTTCCTTCACG

>V300080312L3C001R0641309079

GTTATACATGACTTTTTTAAACCATGCCACTAACGCCCCAGGC

>V300080312L3C001R0200198760

CAAGAACAATCCGCTGCGTTCATTCCGGTCTGAACCTGCGTAC

>V300080312L3C002R0050401562

CGATATATTAGCAATTGCTGACCAAAACTAGGCACACAGACTA

>V300080312L3C002R0061042507

GACATCTATACTTGTACACGCTTCCTGTAAGCAGTTAAGTCCT

>V300080312L3C004R0651065705

AATCTTGTGTCATGGATATCTGATGCTCCAACACTTTTTCTTG

>V300080312L3C002R0300837273

CCAGCACTTTTTGAGTTTTTGCTTCAGTCATGCATCCTAACAA

>V300080312L3C002R0610594997

TGTTTTCCTTGCGCTACCAAGAGCGAGCCGTTGTGGTTGAGCG

>V300080312L3C003R0430594328

CAGATAAATCGAAAACATTACTCTCATATGCATCCTCCGTTCG

>V300080312L3C006R0230570192

ACCAAGCGCTTTGCGCGCAAACATCTTGCGCACCATCTCGACT

>V300080312L3C005R0331010546

ACCGCACTTCTTCGCCAAGATCGTGATCTGTCGCAGAATAGTA

>V300080312L3C006R0641177764

CTGTCGTTTTTTCGTTCGCAAGCTGTACTCGTTGTTGTTCTTC

>V300080312L3C002R0170540190

GAGCTGGTGCGCGCGGTGCCGGTGCCGGCGGCGGTGTTGCCTC

>V300080312L3C005R0510075539

CGATTTTGGACCGTTTCGGCTGAGGTCCTTGTTGCTCGCCTCC

>V300080312L3C006R0310227238

GGACCGTTTCGGCTGAGGTCCTTGTTGCTCGCCTCCAAACAGC

>V300080312L3C004R0351135344

ACTTGAGGCCCCGTGGTTCACTGACGCTGATTTATGTGCAGGG

>V300080312L3C002R0441138644

AAAATTCAAGACGAATCTCTCTCTGTTGGCACATAATTGACTA

>V300080312L3C001R0291309414

ACTGTGGTCAGCGTTTTCATGTCGTCTCTGATATTCTTTGCGA

>V300080312L3C003R0640835694

TGTAGATATTCTTATCTAGTGGCTTACCAGAGGAGCGTGACCT

>V300080312L3C005R0020280212

AAGCATTCATCGAGTGCGCCGACCTTATCGGTGATGGAGAAGC

>V300080312L3C002R0540397867

GCTGGCGATATCCAAATTACAAAGTCTGTTGATTTGCCAAAGG

>V300080312L3C004R0061378914

GTCTCATTACGTTCCCTCTCTTTCAGTTGACTTTCTAGACGCT

>V300080312L3C006R0250146812

ACGGACATCAGAGCGAAGTGTTCTCGTGCGCCTGGAATCCTGT

>V300080312L3C003R0440784811

AACTGTACGGAGCAAAACACCGTTTGCGAGACCAATGTTCAGA

>V300080312L3C006R0581300041

GGCGGCGCTCAGGTACCTCACCGACTGCAAGGCAAGTGATGTT

>V300080312L3C004R0180863810

AAGTATACGATTTCACCACCAGAGAGTGCGATGACTACCTGGC

>V300080312L3C005R0480829337

TCATCTTGTGCAGAGCATATGCCGAAATAATGATACCTCGATT

>V300080312L3C004R0640823526

CTGCTTCTCTATGGCACCAATCATCACTGCACGACCCTTTGGG

>V300080312L3C004R0310219113

TGGTACTAACTTAATTGCGAAACAGTGCAAGCATGCTGGTTCT

>V300080312L3C003R0250244582

GGACCCGAATATCAGCTTTTTCAGAATGGATTTGCCTGACGGA

>V300080312L3C001R0230886261

GGAACCTACTGAGCGATTCAATCTCCAGTTTGGCCGGTAATTG

>V300080312L3C004R0180331187

TGAAAATTTCTGGGCAACTTTTATTATCGCGCACGCAAGCAGT

>V300080312L3C005R0570252416

GAAATCTGTCAAACGTAGCAACAACGGGAGCCGAGCCGATGCG

>V300080312L3C004R0591193603

AGACCAAGGCATTCTTCTGCGGCAAGCCCTCTGCTCTTATCAT

>V300080312L3C005R0561403870

TGCTAGGCAACGCTGCCCGCCCAGTCGGTCGCAGCAGACTTTG

>V300080312L3C001R0640854176

TATTTTGTTGAGCATGCACGAGCAAATCGATCAAAAAAATGGC

>V300080312L3C006R0301217833

GGTTGCGGCGGAGGTGGAGGAGGACCATTGTACGGTGTTGGAT

>V300080312L3C001R0540281507

GCAAGAACAGGATGAGCCGCTGCATGGACAGGTATCTGACGTG

>V300080312L3C006R0391320976

AGGAGCCTCAGATGACGAAATTGCAGCAGCTGCTGAATGGTCA

>V300080312L3C003R0300998674

CCAAGGTCCCCGAGGCTGCTGAAACCCCATTGAAAGCCGTTCG

>V300080312L3C004R0431217633

CATGGGAAGCCGTGGCGAATCCAACCAAGCTTCCCGGTCGTGG

>V300080312L3C006R0260460719

GATCTTTTCTATATTCTTCTATTTGGAGATGGAATGCCAGGCA

>V300080312L3C001R0720905937

TAGATTGGCCATATTATGAAATGTAGAGAGGCCATCGTTCTTC

>V300080312L3C005R0321094368

TGCATCGCGAGAGGCGGGAGAAAAGACGGAGAGGCGCGAGACT

>V300080312L3C006R0240420418

ATGTTTGAGAGAACAGAGCCAAAGAACGAGATTTTGGATCCAG

>V300080312L3C003R0500881624

AAACAACAGTGAAGTGACTGAATACACTCTTTACGCGTGGATA

>V300080312L3C001R0030827374

ATACCCTCTTTACGCGTGGATATTTTACATTACAGACGAAGAC

>V300080312L3C005R0130320690

GTCCGGAAACGTCCTCTTACCCAAAGAGGTCATTCGTCAGATT

>V300080312L3C003R0110588683

AACCCTTCGTGCTTGGTTTTGGTTGTGGCGGGTATATTCTTTG

>V300080312L3C003R0101174861

TACTTTGACCTAGAATCGTATGACACAATTAGAATGGAAAGGA

>V300080312L3C002R0140570940

GGAGCAGCCCGGTGTGCCAGTATTTTACCTCGATGTCCGTCCC

>V300080312L3C002R0170537965

ACCAGACGATGGAAGGTCGATGTCAATGGCAGACAGGATGCTG

>V300080312L3C003R0340163356

TTCTCGTCATTGGCATAGGAAAACTTCGAAAATATGGGATTCC

>V300080312L3C003R0561057727

TTTGATATCGTTGAAATAAGCAAAGTAGTACTCGTCCAGAACA

>V300080312L3C005R0250189120

GTAAGTGTTTAAAAGAGAGATATCAGTAATAACAGCTTTTTGC

>V300080312L3C003R0650120709

TTTGCAGAGGTCGCCAAGTCCGCTTAATTCCCCCTCTCGATCC

>V300080312L3C001R0380356240

CGTTCTGCTGCAACAGCTACGGAGCTAATACGCACGGTTAGTG

>V300080312L3C006R0381060099

GGCAACGGTGGCCAGCCAGAGCAAACTGTACCTTTCAACACTA

>V300080312L3C005R0420287293

TTTACTCTTAGCAAAGATTAATAATCCAAAGTTATTATCATCA

>V300080312L3C004R0460176644

TGTCTCGTTTATTATGGAATCAGCTCAAGGGTCAGCTCGGCCC

>V300080312L3C004R0091181951

CATTGACATAGTCGATAAGGCGAGTTTCACTGGATCAACGAGA

>V300080312L3C002R0450814654

GGATGCGACCGTGGAACACGTTGCGCTCGGTGCGTTCTCGAAC

>V300080312L3C002R0231333135

GGCACGCCGAGTAGCGCAAGCCCTGCATACCGGCTACGTCTGG

>V300080312L3C001R0320206733

CAGGAACGGAGCATCCGAAACTTGGGTTTTCTTCCACATAGTT

>V300080312L3C004R0500440357

ACATCTTCTTTTGCTTTGAAGTGTTTACGTCAGACTTTTATGG

>V300080312L3C002R0401321246

GCAAGGCAGGCCAAAAGCAGCCCACGCATGTTTGCGAGGATTT

>V300080312L3C002R0690885254

GCTATATGGCAAGCGCGAAAAGATAGCAATCTACGTTTTTCGA

>V300080312L3C005R0520739649

CGAAGATCTTGCCGGAGGCAATCCCACAATACCGGAGGAGCTA

>V300080312L3C003R0630160911

AAGTTTCCAGCGCGGATTTCCAGATTGCCCATCCAAAATGAAT

>V300080312L3C006R0561012243

GTCGCAGGTAACTGGCAACTGCAAAATCCCTCTCAAACATCTT

>V300080312L3C006R0500621504

TCTTCTAACGCCATGTTAATACATCAGATTTGCCATAATTGCC

>V300080312L3C005R0040845570

TAGTATTTGCTTAGTATATCGGCAAGCCGTGATCCATGTTTCC

>V300080312L3C003R0391317825

ACCAGAACTGCTTAGTGGGATTGAGCTCAGGCGCGTATGATGG

>V300080312L3C004R0491342848

GGGCTCAGGCCTGCCATGCTCATCAACAACAAGGCCCTGCCCG

>V300080312L3C006R0291355862

TCTTTTTATCCATATGCTCGTCTTCGAGATAGCTCAGTAGAAG

>V300080312L3C003R0060244290

CCATGTAAAAGTTAATAATTTCGTCGGTGAGCCATGTCTCAGG

>V300080312L3C005R0340059744

CTCCTCTTCGTCTGTCAAAGGCTTTACGGCCACTGGTTTTGGT

>V300080312L3C002R0171202775

AGTTGCTGGTTTCTGGCTTCCTTACATCCAGAATATTTTGAGC

>V300080312L3C003R0711274099

TGATCGTCTGTATCTTTGGAACTGACCATATCAGAAGATCCGG

>V300080312L3C004R0470603841

CGTGTGTTGTAAATTCGAAAGGAACGGTCCGAGCAAGATCTTT

>V300080312L3C001R0481200764

AAGAAGCAGAGCGGACGGCCATGATGCAGCATCTGAAAAATGG

>V300080312L3C004R0270744693

TGTGTGAAGAGGAAAAGCAGTCCAATCCAGAAAATAATAGCAT

>V300080312L3C002R0370516882

ATGGATGCCAGTGGCTCTGGAAGCCATGCCGGAGATGACAGCG

>V300080312L3C004R0061001122

GAACATTAAGCCAATCAGAGTAATTAAATTGGGACTAAAATCA

>V300080312L3C003R0710257622

AATTCGATCGACCAGTTCCAGTAATGGCGAAGGAAGAGCCTGG

>V300080312L3C001R0590046478

TTCACAAGTATGGATGAATCTGATAGATTATGGGCTGATCGGC

>V300080312L3C006R0361332882

CTGGCGTAAGAAGATCGGGCAAGGACTTGATGGCTGCAGTTGG

>V300080312L3C002R0171193295

ACCTGCAATTGCTAGCACTATCAGAAGGTTACCTCGTCAATCC

>V300080312L3C001R0361253505

GGGCGACTTGTGCGACATGAGGTAAAAAAGAAGACAACAAACG

>V300080312L3C003R0070563386

CATTATCAGTGGCATATCGATTCTACCATCCATCGTGCTTTTC

>V300080312L3C006R0620370672

TCCATCGTGCTTTTCATGCGAGGCAAGGTTCTCCATATTTGCA

>V300080312L3C004R0050623018

TAACAATCAGGCATACTGTCGATACCACTACTCGTTGCTACAC

>V300080312L3C003R0690233905

GAGCAGCTTCGCAGAACACAGGCAGAGATGGATCAAAGACGAA

>V300080312L3C003R0421224099

CCCGAATCACAGCAACATCGCAACACCTCACCCGAAGATATTG

>V300080312L3C006R0261172424

TCTATGTGTAATATTACCGTTCTGTGTCTCCATCCGCCCTATT

>V300080312L3C004R0170648054

CATGTGTCTGCGGGCCGCTGTACAGTAAAGATTCTTCTCGCCC

>V300080312L3C005R0461380740

TGGCGGCCATATGTGGTATTCAAAAAGTATAAAGAGAACGATG

>V300080312L3C002R0401217988

CAGCGACGGCGGTGCAGGTGAAATAGTTGAACAAGACCTGTGA

>V300080312L3C006R0260220097

CCTGTGACGCGCATGTTTCCGTGCGTGTTTCTCTGCAAGCATA

>V300080312L3C002R0670443446

AGAGGATGCTTCAGATGTGAATAAATGTGATCGTCCTCTACGT

>V300080312L3C002R0690583483

ACGCGTCAATATCTTTACTGACAAGAGCCCGAGTGAGTTAAAC

>V300080312L3C002R0430760658

TAACGTATGTGTTCAAATCGAATACGTTCATAGGAGATCCATC

>V300080312L3C001R0520061527

CCTTTCTTTGTTCAAATGTATACAACAGAAGGAAAAACCTTGT

>V300080312L3C003R0501345961

TGCTGGTGGCGGTGGTAATGCTAATAGCAGCAGTGCTAGTTCC

>V300080312L3C002R0370185641

AAATTCGTCGTCTCTTTCTCAAGCAGTAACTCGAAGTTGATCC

>V300080312L3C005R0141159159

GGTAGCAGGGAAGTGAATTATAATTTCTGCAAGCCAATATCAA

>V300080312L3C006R0290081455

AGCCCACAAGGCAGGGCACCGCGCTTACCTTCTAAAAGGTGAA

>V300080312L3C001R0240241765

CTTCACATACTCTGTATGCAAAAAGAGCAATTTAGAGTAAACC

>V300080312L3C005R0580760912

CAACATATGATCGTCGTACAGGCATGCGGCGATTTTGTATAAC

>V300080312L3C003R0061265796

TTGCTCCGGAATGGATTGATGTACGTTTACAACTACACGCTGC

>V300080312L3C002R0160420793

TCTTCTTCTTCCAGCCGCTTCAAATTATCTTCAATTTCTTGGA

>V300080312L3C003R0310155871

TGCTCCAGGTCTTCGCTCGCCGATCTTTTAGTTGTCTGCTGTT

>V300080312L3C001R0590176587

TTCAATCATCGAGTTTGGTATATTGTAATGCAGAAAATATGTT

>V300080312L3C003R0060649335

GACGATGATGAGCAACATCTAAATCGTATAAAACCTAATTTTT

>V300080312L3C005R0700009947

ATCAATAGGATACTCTAAGAACTATCATCCTTGCTAATTACTG

>V300080312L3C005R0301398530

TCAAAATATGGGACAAGCTCAATATACTAGGCCCGTCAGTGTA

>V300080312L3C005R0570014178

GTACTCATGCCGCTTGTCAAGTATCTTTCAAGATCGGTTTTCG

>V300080312L3C006R0201212826

ACCTGCGTTGGTGTCTCCGCAAATCTACTCTTATCTCGATACC

>V300080312L3C002R0560802624

ATCAACGCTGGCTGTCTATCTCAAGGCCCTTGACACGTTCCCT

>V300080312L3C001R0060116149

GGTCGCTTTCAAAAAGATCTCTAACATTATACGCGATCGTAAA

>V300080312L3C003R0020728982

CTAATGTAAAAAAGAGAAAATAAGCATAGCGTTATTTCCTAGG

>V300080312L3C003R0030878838

CTTTGAAGCATATCGTCGGAAGATGGCATTTCGGATGTTCGTG

>V300080312L3C003R0660294943

GACAATGTAGTCCTTTCCGTCTTTGGTCACTTTCTCCCTGCCG

>V300080312L3C003R0410407313

AACATTGAACTCGGGAGGTGTAACGACTGTAATGTGAGCTTCG

>V300080312L3C006R0190721371

GCTGGAGTTCTTGTTTTTCTGTCTTTATATCAGCCATCACGCC

>V300080312L3C001R0391136108

TGTCCTTTTGACGAGCAGAAAATGTCACATACTGAGTTGATCA

>V300080312L3C004R0040219590

GTGTGGGGATATCACTCCCACGTAACGGAAAATGACATGTAAG

>V300080312L3C005R0220633495

CATGCGATCCAAAGGTTCAAACAATCGAACATAAAGCAAACTA

>V300080312L3C003R0350542856

GCATCTCCAAATCTGCCGGTGGTGTCGGTGTCAGTTTCCAAAA

>V300080312L3C005R0701308960

TCTAATAAGTACCTAAACAGGTATCTGGAACCTTGGCACTCTG

>V300080312L3C005R0020483914

AAGGTCATCGATGTCAACTACTATCCTGTCGAAGAAGCTCGTA

>V300080312L3C006R0690784396

TGCCTACTGCAAGCACCAGCCAGATCCTCGGAAACAACGAATG

>V300080312L3C004R0630671232

ATTGAGATGCGTGCGATATGACTCGGCGGCCGTGAGAGGGTCC

>V300080312L3C002R0460071148

TGCTGGGAGGGGGCTGCAAAGCGCTGTAATCGCTGTGGCTAGG

>V300080312L3C004R0070278084

TAGGTCGGCTTAGCGGCTTGCTTGTAGTGATACCGTGGCTCTG

>V300080312L3C002R0590809837

TCATTAGAATTAGGGGAACTTCGGATATGCAAAGACTTATTTA

>V300080312L3C005R0371036070

ACGTACCGTGGACCGCTTGTGTTCTTGACAATAACTACACAGT

>V300080312L3C002R0231168159

GCCCGGGAACCAAAGCCCGACACAATTTCTGGTTGGATTCCTT

>V300080312L3C001R0431035069

CAGTCAACTACTTAAGATATTCCAAACGGAGCAGCAGCGAGTA

>V300080312L3C006R0090642128

CCTCACAATCCTTCTCAGCCATATGTACATACTCTTCGCTGCT

>V300080312L3C005R0420621071

GGTACAGACACAACACTTCTCTTTTCCCGCTTTGACTCATTAA

>V300080312L3C003R0621217505

CGCTCTCAAGCACGAGGTAGCTAACGATCATTTCTAGCACGCA

>V300080312L3C006R0560087287

CCTCCTCCTCTTCTCCTGGTTGAACAACAGACCGTCGTTTGCG

>V300080312L3C001R0250662337

AAACGTTTGGTGCAAGAATAAAGCATTCGCTCCTTGATTTTGG

>V300080312L3C002R0121255305

GATATTCTGCAACGACAGTACCTTGACGGTACCGTAGAGGTTG

>V300080312L3C002R0520993369

AGTTTCCGTGTACAAGCTCGTCTTGTGTGCTGCCATTTAAAGT

>V300080312L3C003R0580833149

AACTTTGACTGGGACGCAAGTGTTTTGAAAGTTTTATTGCTCC

>V300080312L3C005R0471033399

CCAACCCGAGGAGTAGCCATCGCCTATCAGCAACTTCCGGACG

>V300080312L3C005R0300690970

ATTTGAGCTCATGGAACGATGGGGATACACGTAAGTCTTGATG

>V300080312L3C003R0390185828

TTGCCGGCAATTTTGGATGTCTCGTTGCCTCTGCCTAGATCAT

>V300080312L3C006R0350164537

TACCCGATGATAGAATCAGGTCCTTCGAACGTTAAGCTATCCA

>V300080312L3C005R0131280556

AGAACAAAACGACGAGAAGATAAACCATGCCTTTATATACGTA

>V300080312L3C003R0391362869

ACCTTGGATGGGTCCTCGGCTGCGTAGAACTGAGTAATGCTGA

>V300080312L3C001R0261291888

TGTGATTGGTAGCAGTATGCAAAAGTTGTTGAAGATGTATAGT

>V300080312L3C001R0030106443

TCATAGGAGCGGACTGGAGAAATGGTGGATATGCAGACCCTTG

>V300080312L3C004R0281276806

TTAAATTACCTAAACAGTCCGAGTCATAACGACCACTGCGAGC

>V300080312L3C004R0530953832

GAGCTGAATCCATGGTGGAAGGATATAGTGCCTGGGGTAAGTC

>V300080312L3C003R0500955692

TCGTTGTGAACTGCACAGGCATGCGTGCTGCAAAGCTGAAAGG

>V300080312L3C003R0621139291

TGAATTTCGGACGTATAAGCAAGCTGCTGGGATTTCCATTTCG

>V300080312L3C001R0150315262

CGGTCTCGCTACTACGATCCAGTTGCATTTTGATGAGCGAGCG

>V300080312L3C002R0690871146

CAGTGAAGGATCCAAAGGATGCACCGGAGGACCAAAGACTCTA

>V300080312L3C002R0490431323

GAATACTTACACAGTGATTCCAACAGGTCCCATTTTCAACAAG

>V300080312L3C005R0370454843

GTATGGAGGACGGTTGCTTGATATACGCCTAGACAAATTCTCC

>V300080312L3C001R0270365137

CAGTTTCTCGACTGGATCAAATACGATCATTACTACTGCTTGC

>V300080312L3C001R0280708696

ATTTCATCGTGTGGAACTGGATGGGCATGAAATTCTTTCGACA

>V300080312L3C006R0170613729

AGCAAAGTCCACCACCACCGCCACCACTGGGAACGACAGCAGC

>V300080312L3C003R0330942608

GGATGTCTTTGTGTACAGCTGATACGCATTCTTTCCAGGTGCC

>V300080312L3C003R0310140400

CGCATTCTTTCCAGGTGCCAAAACCAGAAGCTCGTAAATTTTG

>V300080312L3C003R0330166283

CCAATGCATCGAGACGTACGCTTGCGTGTCGTAAAGCGATGCG

>V300080312L3C006R0420593734

GATAATCAAATCAGGCGTGATACCATCTGCTGTGAATGGGAAA

>V300080312L3C005R0320968138

ATAGCCGGAATAGCACAAAATAGCTACGATGGCGTTTTGACCA

>V300080312L3C005R0710770215

TTGCAGAGCAACACCGGTGGTTGCGGATAGGCGTGATTCTGCC

>V300080312L3C004R0660442602

TAGGTACAAACGATGAATAATCAAGAGGCCCATACAAAGTACA

>V300080312L3C005R0680126854

TCAAGAGCCCCATACAAAGTACAATGAAAAAACCTACGTCAAA

>V300080312L3C003R0101051621

GTAAGTATTATCCTCTCCCTGGAAAGGAAGAATTTGTTAAACC

>V300080312L3C003R0450067209

CTCGAGAAAGTGTTTTTTGAGGTATCTTATCAGTAGCATATCC

>V300080312L3C004R0170544360

ATGATCAGATAAAGAAGATGAATTGGGCCACGCTACATAATAT

>V300080312L3C003R0390562614

CAATAATAAATGAATATACAAAAAAAGCACAAGGTGATGGTAT

>V300080312L3C004R0130511182

CAATCATAAACATGATGGCTTGTTGATAGATGACAGCATCCAG

>V300080312L3C004R0590131778

ACTGCTCGGTTGCCACGGCGATCGTTCCCATGGTGTTTCCGAG

>V300080312L3C005R0330786206

CGTGTGACAATCTTGCAAGCAGCTTCCAACCTCCGCTTCAAAG

>V300080312L3C004R0340852166

TGGTCAGATATACATAAATAGGGATATCATAAAGGGGAAGCAC

>V300080312L3C006R0510956932

TCTGCAAATCCACAATTCCTGCATCCCCTCAAAACAGAAGTCC

>V300080312L3C001R0350481128

AGCGTCGTGACAGTTTGCTGAACTCCTTCCCCGTTGGTTTCCC

>V300080312L3C006R0570803695

AATGGCGTTTGTGCCGAACACTTGCAGCGCAACTTTCAATACC

>V300080312L3C003R0571305829

AGTTCCGACAATGCTCTAGAGGTTGTGGAATCCTTTGTACGTG

>V300080312L3C004R0101192354

ATCACCATGCGCTGTTTCCAGTGGGAAGAAAGGAAAGTTGACC

>V300080312L3C002R0120125686

CCCCGAAGGTGATGTTGTCCGCTACGGGTATGTCTGTCACAAC

>V300080312L3C006R0550208427

GAAATGATCCCCGATCAGGAGGAAGAAGAGGGCGAAGATTATG

>V300080312L3C002R0190228978

TTGCTCTTTCTCGCTGCCATACCTTTCAACTTCACGGGCAGTA

>V300080312L3C005R0270344922

TACCTTTCAACTTCACGGGCAGTAAGGCCATATGCAACTGAGG

>V300080312L3C006R0440522699

TGCCAGTTGTACTTTGCAAGCGTGCTACCTGCACGCACACCAA

>V300080312L3C002R0170281219

GCTTCCGGCTTTTTCCTCCTTCGATCGTTTGGAACGGAACTTT

>V300080312L3C004R0420372887

TAAATTTACTCTTATCGACTCGGCGCCATGATGCATTGGCGCC

>V300080312L3C004R0310514037

TCTTGGAACCGCAATCTCTTTGTATATTCAACAAAATTGACAA

>V300080312L3C002R0591177313

GTAGTGGCGTGTTGCTTACCAGGGCCGGAGAATAGCATTCGCT

>V300080312L3C003R0591012351

GCCGGAGAATAGCATTCGCTTTCTTAGAGCACCTTTAGTGGTA

>V300080312L3C002R0460347212

GAATCAGGTAGCAGCACAGATTGGCGACTTTAAGGCGATGGGC

>V300080312L3C004R0371279317

GTAGGTCGATTGACGGATAGCTTGAAGCAGGCACAGGCGAGCG

>V300080312L3C006R0460860217

AATCTTATCTATCTATCTGTAGTCTGTGTTTGTCTACTGGTTG

>V300080312L3C001R0190650790

AAAATCGTGCATCTCCACTCACCCCGCGTGCCTGCTACTCTGC

>V300080312L3C006R0500938138

CCACTCACCCCGCGAGCCTGCTACTCTGCTATCTTGCTGGGAA

>V300080312L3C006R0150119231

TAGGAAATGTGTTACCTATAGTCAATGGAAGATCTTGACACTT

>V300080312L3C006R0620292828

TGGATTCGCATCTTAGATGTAAGAGATGACAGTTTCCGGAGGA

>V300080312L3C005R0661201390

CACCGGGTCGTGGATCGACGATCTTGTAAGGCAAATCTCTGCC

>V300080312L3C002R0590670778

GTTGAACACTTCAACCAATGCCTTTCTGTCGAGGATATTAACC

>V300080312L3C002R0600389306

AGCCTTGTCTGACATGTCGAGTTCTAATAGAAAGAGAAGAAAG

>V300080312L3C005R0080835973

GGACATCGGACTGCACTGGTCTATGTTTCCATAGACGGATCAC

>V300080312L3C003R0560697942

TTTCTTGTGCAAAAATCATCTCATCTAAATTATCTCTGCTACA

>V300080312L3C006R0140182520

ATCATGCTATTCACGTGCGTCGTCGTTTATATATGTAATATAC

>V300080312L3C006R0401334129

GGAAAGCCGACAGAACTACAAGCAACATGTGCGGAGAAACCAA

>V300080312L3C004R0681396639

TGATCCAAGGTTGTGGAATAAACTCGCTTGGCCTACATAAACC

>V300080312L3C005R0501337776

TACTTCTCTGTATACGATGTTGCGAGTGCTTGTGGTGGGAGTA

>V300080312L3C006R0191014019

TGGATGGCGTTGACAACGGTTTCATCAAGCGTGACACCGAGGG

>V300080312L3C006R0320749712

CACAAACAACTCGAGCGTCTCAAATGGTCTGTTCGACCACAGC

>V300080312L3C006R0420242163

GTAGCATTCTATACTTAAAGAAGCTGGATGGACGACGGCAGTG

>V300080312L3C004R0491112571

GACGGCAGTGTTATTTGGTTGAAATACATGAGACTTGGTCAAT

>V300080312L3C006R0200950710

TCAATGGTAAATGGATATATTGCCTTTCAAATCACATCAAATC

>V300080312L3C004R0400570574

CGCGTCTGCCGAGAATGGACCATGTCATTGCCTACTTTTGCTA

>V300080312L3C002R0070408049

CCATCTTTTCCTCAAGATATTTGCTGTCGTTGGAAACTTTGAC

>V300080312L3C003R0530147753

CATATGCTTCTTCTACTGATTGAAATTGTGCTTTGGTGTCCTC

>V300080312L3C001R0540187873

GCGAACGATTCTTGCAAATGCTGCATCTTTTGCTCCACATCCG

>V300080312L3C001R0070490949

GACTCGATGGTAGTCACAATGCGCAGTCCTTCTCGCAGCTGCT

>V300080312L3C001R0550351953

TCGGCACTACAGGAGTTACTAAACTGGTCATTGTATCGAGCCC

>V300080312L3C005R0161082102

TTATCAGTATTTTCCTCGAATCTCGCAAAGCTGCGACTCAGAC

>V300080312L3C001R0300477566

GCTGCGACTCAGACTATCCATGCAAGCTTCAATGTGATCCGGC

>V300080312L3C001R0250283125

TGCTTTTTTGCTCGCAAAATCCCATAGTTTTCGAGCAAGTGCG

>V300080312L3C001R0690696101

CAGTTTCTGGTGCGATGCAATCAAGGTTTCTGCGTGAGTTACA

>V300080312L3C005R0620227265

TGTCTCGTTCCTTCTCCATTCGAGCGAATTCTTCATCGTCGAT

>V300080312L3C002R0331398137

TGCTTTCGAGATAGCAGCATGCTAAGTAGCATGTGGCGGTCAG

>V300080312L3C002R0490312281

ATCAATACTCGCGTTTGCATCCCTGAATGTGACAAACAAATTA

>V300080312L3C001R0060356038

TAACTGGACACTGACATCAACCCGTGCAAATCAAAGGCAGTTT

>V300080312L3C002R0591333696

GACGTTCGTCTAATCCGTTGAAGGCTCGTGATAACAACCAGAT

>V300080312L3C001R0310415683

GGGGAGTTTTCGTACGAGGATATTCAAAGCTTGACCAATCTTC

>V300080312L3C004R0530394308

GTTGAAGCTGTGACCCTCAAAGATGGTAAGATGGGAAATTGGG

>V300080312L3C006R0431147025

GCATCAACACCGGTCTTGAATATTCCAGAAGTGATCGAGACGA

>V300080312L3C001R0481164110

CTGTCCAAGTTTTGACTACTACGTTCAATATTGCAAAAATGAC

>V300080312L3C005R0520830560

TCAATTGCACAGCATGTCTAGCCATCGACGTAGCCATATTGGG

>V300080312L3C002R0490158249

TTTGCCAAGGAAGATCTATCCAATCTCGAAAAGCAAATAGAAC

>V300080312L3C004R0340515093

AAGAAGGAAGCTGAAAAGAACGCTGCTGCCAAGGTAGATACTG

>V300080312L3C003R0711281995

ATCCTTTGTATGCCAACCGGCACAGTGCGCAAATGTTTGGACG

>V300080312L3C006R0351398707

GAACAAAAGAAACAGCGATCCCAATTCTACGATGCTCTGCTTA

>V300080312L3C001R0691061255

GTATCTTCAAAGAAGATTTCAACATTTCCACCAAAGGTAATTT

>V300080312L3C003R0150392046

TTTGTCAAAAATCAATCAACTGATCGGCTGCCTCAAATGATTG

>V300080312L3C005R0421342207

ACCCATTGCACACAGAGAGAAAATAGCTGGTAAATACGAAGGA

>V300080312L3C005R0271083669

AGAAAATAGCTGGTAAATACGAAGGATCATCGGCAATGCTATC

>V300080312L3C004R0540754948

TCGATCGTAGCTTTCAATGGCAGCTTGGAAACTGCCGGTCGCA

>V300080312L3C001R0051046249

CAGCTTTTGCAGAGCGAAAGCTGGGGAAATAATAAGGTTTGCA

>V300080312L3C004R0280829042

GAAGATTGTTTCTGCATATCCATAGCTTGCTTCCAGCTGTCAA

>V300080312L3C006R0590814211

ATTGATATTGAGTTGATTCCATCTTGATCGCTCTGCGAATACA

>V300080312L3C002R0711110439

TGCGCAAAGATGGAGACATGTTGGAATGGTTCGAAATACACTG

>V300080312L3C004R0651213564

CGCTTTCTGAAAGCAAGTTATGGCCTCATCGTAGTGGTCTTGG

>V300080312L3C004R0451286362

TCGCACGATCGTCCACCAGATATCTTTTGGATGACGCGCTGTC

>V300080312L3C004R0490810556

AATGGATATACTACATTTGGGGAAGGGTTTGCATAAAACAGTA

>V300080312L3C004R0460661861

CCATTGTAAAGTTGATATACTCTCCCGTTCTAGGATCAACTTG

>V300080312L3C002R0260790425

CAAACCAATGGCCAAAGGTGAAGGTAGCAGTAGCGCTTTGAAG

>V300080312L3C004R0470714160

CCGCATAGGCAAACGGTACCTGCTTTTTAGTTTGAATCAATAG

>V300080312L3C005R0280019165

CGTTACTTCGTCGTAAATGGGTGCAATCGTAGATAACAAGCTG

>V300080312L3C002R0021151560

TGGCGTACTAGCAAGTAAGTATAAAATACTGCGGTAGCCATCG

>V300080312L3C004R0440869648

ACGCAAATTTGCATGGAATCAACGAGTATATGATGGTATTCAC

>V300080312L3C004R0680443719

CTGCTTTCTATGTTTGTGGGTATGGACGATTGAAAGTCGTTAA

>V300080312L3C003R0570072008

CCTCCTTTCAGCAGTTCAGCGACATTTATCTTACCGCCACAAG

>V300080312L3C006R0691392402

AACCTTCTCTAGTGAAGACATGATGAAGTGATACGAATGAGTC

>V300080312L3C006R0261114900

ATGGATTGCTCGAGTTCTTCTGTACATCGTCCCAATCTTTTGT

>V300080312L3C002R0300750431

GTGCATTTATCTGTTTGGCATAGCGGATGACACCAATATTGTC

>V300080312L3C001R0180118558

TAAGTGCAAGTACTGAAAACATGCAATACGTACGTCAGTTCTC

>V300080312L3C001R0720857141

AAACAAATTTCCTGCATCAAGAGCGCCTCCACCGAACCTAATC

>V300080312L3C005R0431144088

AAATTTCCTGCATCAAGAGCGCCTCCACCGAACCTAATCGTAC

>V300080312L3C004R0420768142

AACAAGATTCTAATGCCAACGGTACACAGGAACTAGGCATCGG

>V300080312L3C001R0290882367

GAGCTCGGCACCGGCGGTATGTTTTATGATATATTGCAGATTC

>V300080312L3C006R0620237169

CAGCCATTGCTTCCACTTTGACGTGGTCGAGCGTAACTTGGAT

>V300080312L3C005R0480076845

GGTTTGAAGAATATCATCACTTAAACGGGGAGAATTACTCTTC

>V300080312L3C006R0561326372

GGAATCCGCCGTAAGGCTGCCCGAGGTATCCTTGGAAGAATTC

>V300080312L3C006R0150384825

AGCAGCGGCAGCAGCCAACATGGAAGCAACACCGGTGCCAGCA

>V300080312L3C001R0340821877

TCTATTACATACGACTATAAGCATATATGGCTCCTGCGAAATA

>V300080312L3C004R0470078325

GCAAACTTTGGTGTGCGTCGCGCCACGTGGTATCCATAATCAA

>V300080312L3C001R0600769355

AACACGCATGCCGCGTCCGCCGCCACCCATGGCAGCCTTGATA

>V300080312L3C004R0060249943

CGATGACGCATTTTGAAAAAGTCGGAAAAAGAAGCAAAGAAAT

>V300080312L3C003R0490822221

TACAAGTGTACAATTTCTTGCCGCAAACAACAGATGGACTAAG

>V300080312L3C003R0381333736

CAAAAATGAGCTTGTCGATGGATGAGAATTATCTGACGAATTA

>V300080312L3C006R0360199072

GGTGACGCTGCGCGTGCCACGTGTCCTTGAGGTGTCTTCGGTC

>V300080312L3C004R0530622994

TCCAGCCAACGAGTCAATTTAGCTAAAACTGCATCAAAACATT

>V300080312L3C003R0270363069

CAGGTACAACTTGAAAAAGGGCCATGTGTTTCGGATAACACTT

>V300080312L3C003R0160798531

CTGGTTGGCTCGACATGCATGCAGGTTTGTCATTGCATACATC

>V300080312L3C004R0221012956

AGAAAACATGCTTAAATCTTTTCGAGATGGTAACTGAATCGTT

>V300080312L3C004R0310981016

AGCTCGTGCTGCTAGCCTTTTTCTGCGAGAACCTTATCTCCAC

>V300080312L3C002R0260775310

GGAACAGCGCGCGACGTTTCGTCAGCAGCAGCTAGGACTCTTT

>V300080312L3C005R0381136633

GACGGCGAGAAAGTCATTATGGAAGGCGATATCGGCGATCGGT

>V300080312L3C002R0390784027

AAAAATGGCGCAAACTTTACATTGATGGTGTGCGGTAAAAATC

>V300080312L3C001R0070909098

GGACGAGTCAACGACTAGACGCAATGCAAACATCATGGCCGCT

>V300080312L3C005R0240201720

CATGGCCGCTATGCCATTTGCCGTGATAGGATCAACGCAGGAC

>V300080312L3C006R0100606195

TGTGACAAACGATGGACGCTGTGTCAAGGGACGCCAATACAGC

>V300080312L3C006R0641403844

CTCTATTTCCAGAGTAACCAAGCTTCTTCACCTCGCGCTGCTA

>V300080312L3C004R0201260741

GGTGCTCTGTACAGCTTTTTGATGGTAGAATTGCGTTGCGTTA

>V300080312L3C004R0361284400

CAGATAACTCTCTTTCGGTTTTCGTGACCTTATAAACTGTTCT

>V300080312L3C004R0300678861

ATGTCTGCATTATTGTTACCGCGTGCTTGACTTTAGAAATAGC

>V300080312L3C001R0620887355

AACGCTGGAACCACAGCAGATGCATTTGCACGCGCTTTGGAAC

>V300080312L3C006R0480084177

CTGCTATCGGTCACAGCCCAGTACCCAATCGATCAGATGACAT

>V300080312L3C004R0560309054

CCTCCACGTACGTTATGCTTATTGTAGCTCCATTCAATTTTGG

>V300080312L3C005R0620558417

CAACCTCGATGACGATACACTATCTGCTGTATCAAGCGGATCC

>V300080312L3C005R0420044613

TCGATGCGCTCAAGAGGAAGTTTAGACCTGGATGCATCCTTAT

>V300080312L3C002R0160042444

TAATCGCAAAAGAACTTTGGGCGCCATGGTTGGAAAGATTTTC

>V300080312L3C006R0680642491

AAGGCGGGAATACGGGAATGAATATGAAGAAGTCAAGAGCGAG

>V300080312L3C001R0291209144

ATGCATCATACAAGCACAAAATACGCGCGTTGCAAAATGCATA

>V300080312L3C002R0721329517

ATGTAAACTGAATCATTGTACGTCTGGGAACCAGCTCATGATG

>V300080312L3C006R0360620185

GAATCTGCTTACTGATATTCGACGATATGTTTGAAGCAAAGGA

>V300080312L3C002R0270556174

CTCCAAACGTTGCTGTTTTACCAAAAATGTTTAGCACGGATGA

>V300080312L3C003R0320349453

GAGTTGGAAAAGCTCGCAAACGAGTCAAGAAACTTCATGGATC

>V300080312L3C006R0660789599

CCCAAAGAACTTCCTTCTGCAACATTTGTTCTTGGTGTCTAAC

>V300080312L3C002R0340691539

TGAAAGAAACAGTGCCAACCTGTCCACCTCTCAAAAAAATTGG

>V300080312L3C001R0640141195

TGACCAGATGGAGCTTGCATTGTATCAATATGCAAACAGCTCA

>V300080312L3C002R0500769606

CATGAAGTACGATTACCGCCATATCCCAAAAGATATGCTTCTT

>V300080312L3C006R0211327349

TATCCCAAAAGATATGCTTCTTCGAATTTAAAATCCCAGAGAG

>V300080312L3C001R0051198356

GCATCGTCAGCAAATTCTGCCGATTCTGCACGTCGACTACTGG

>V300080312L3C003R0680798563

GGATCTGCTGCCGTACTTGATCTAGCTGATTTTCATAGTTTTC

>V300080312L3C003R0620695106

CACCTCCACGACATTTGGTGCTTGGTATAATTCGGAGAAGACA

>V300080312L3C006R0301066374

TATTATGATAACAGAAATGATATAAATGCACAATGTCTTCAAG

>V300080312L3C002R0190539613

GTTGTCACACAGCGACATACCTAATCAGCTTTTTATTCTTGTG

>V300080312L3C003R0140681896

CGTTGGTCGAGGATAATAAAGAACGGGATACAACTCGCTCGTT

>V300080312L3C005R0221294792

ATGGTTCCAGTCCAAAAAGCTGAAATACAGCACCCATACATTA

>V300080312L3C005R0101358268

AGGAGATCATGCCTTCTAGCGGAATCGAACCGCTGACCTTCCG

>V300080312L3C004R0150529008

ATCAACGTTGTCAATCAGTTGATTCATCGAAAAATCCCAGTTT

>V300080312L3C003R0400609961

TCGTCTGGGAGATGCGATATATTATTTATTGTTCCAAATGTGA

>V300080312L3C005R0180952621

CCCTGTGCTTATAAGCCCAACACAGCCTCCTCGCTTTCTCTTC

>V300080312L3C005R0570935850

TGCGCTTCACGTTGACACGAAAGGCAGACTCATCTAGAAAGAC

>V300080312L3C005R0380923401

ATCTCACATTGACATTTTGCGGAAATAACAAATTCATACTCGG

>V300080312L3C005R0221034587

CTGTCTAGTGACGAGTGCGAGAACCGGCTTCCCACTAGATGTT

>V300080312L3C005R0441276055

CATAACATCATCGTCGCTGTCTTTACTTTCCGCCAGCATCTCT

>V300080312L3C001R0511311652

TCCCTAGATTTAGTAAGGTCATCTAGTGATTCAGTTGCTAATT

>V300080312L3C005R0301183372

AGCCTCTTTTTTCGTTCTTGATCTGTCAGTTTGTAACATTCTG

>V300080312L3C003R0380902024

AACGTAGGCGGATCTTAAGACCGGACCATAGATGTATTATTAT

>V300080312L3C003R0480266252

CGCACCGTTTTCAGGGCCTTAGTTGCTATCCATGTTTTGGAAG

>V300080312L3C004R0120376412

CAATGGAGGTGCATACTTGGTACCGCCGCTGCCTCCGACCCGT

>V300080312L3C004R0070535430

CACGAGAGAGAAGAGTGAGAGTTGCGGGCTTCTCTTTGCAATT

>V300080312L3C003R0140905593

ATAGGCTCTTATAAAAATTGCGTGCCCAGAGGTTCTGAAGTAG

>V300080312L3C002R0430946647

GTACTTCTGCCGCTTGAGGCTTCTTTGACATTTGTAAAGTTCA

>V300080312L3C004R0251380003

ATCCCAATATCTTATCGACTCATCAGCCGCCGTATTTCCTGGG

>V300080312L3C001R0400643816

CTTGGAAAAGCTTGGATAGGTAAGCACAAGAATGTACAATCAT

>V300080312L3C004R0680913638

CCTTTTCAAGCTTTCAATATTATGCAAGGGTTCGTCTATGGCA

>V300080312L3C005R0580230018

CATGGGTTCGTCTATGGCAAGATCTAATTGCTCGCAGGCTGAA

>V300080312L3C005R0390564236

TATTTAGTATGTTTTCAGCACCAGATTTTTCATTTACTATGCT

>V300080312L3C001R0620356028

CAGAAATTGTTACTTTCGCGGCGCGAAGCACCGTTGAATGAGC

>V300080312L3C003R0540639234

AGCATTACCCATTCAACGAGCTGTATTACATGACTGTTGTTGC

>V300080312L3C004R0571100370

TACACCGTGACGGAAAGAAATTGCGCACGAATTACAACAGCAG

>V300080312L3C006R0380970892

CTTACCAAATCCACTAAACGCAATCAATCCCATATATAATTCC

>V300080312L3C003R0440239300

ATATAATTCCGCAGAAAACATTGCGTCCGAGCGTATAAACAGA

>V300080312L3C003R0180274139

GCCATGTACATTCGTTGTGCTGTTCGGGATCATGGATGTACTC

>V300080312L3C003R0380440973

GGAGATCTATCGACATCAAAGCCGATGATGAAAGAAAAACTCG

>V300080312L3C001R0620348662

CCCACCTTGCGTCAGTTGATGAATATCCAAAAACTTTCTAGTC

>V300080312L3C004R0280993781

CCAAGTGAATATCTCCTTCGCTGGGACCACTGCAGTAATCAAC

>V300080312L3C002R0100451774

GAAACCTAGGCTTCATCTTGTCACCATGGTCCTTTTGGGTATG

>V300080312L3C005R0320194168

ACAAACACGAGGTAAGCCGCTGCAAAAGCTACCACTGCAATAG

>V300080312L3C006R0520099475

GGTCACATGCGTTTCGTTGACCTTCCTTGGTTATTACCAATTT

>V300080312L3C006R0620069307

ACCACGTAGCTACAAATAGCTGTAACTATATTTTGATGTATAT

>V300080312L3C003R0460485384

CACTCCGGTTCACCCATGCATCGCATGCTCTGCTGTATTATCT

>V300080312L3C005R0331019227

TTCTTTTGATTCTTCTGACAAAATGTCAAGAAGATATTCGAAA

>V300080312L3C005R0370543344

GTCATAGGCGAAAGTAGTATGATGAAGAAGAAGGTTTGTACGA

>V300080312L3C004R0460568066

CTCGATTTCTGCAAAGCTGAAAATAGGTCGCCTTTTGTAGCGA

>V300080312L3C004R0131134080

CTAGACGATGGCCACAGCCCGCATAACCTTTCTCGAGTGTTTC

>V300080312L3C002R0301111239

AATATCGGGGGACAGAGTTTATTTCAGGATGGGGAGTGCAGCA

>V300080312L3C001R0290175532

GGGTGGTCTAATCGTAGCCGTGCAAAAGTCAAACACTTTTATA

>V300080312L3C004R0311224274

GATGTCTCAGCTCGCTCTGATGGTGACGGCGCATCCTACACGA

>V300080312L3C005R0471218599

CGAAAACGACAAGTACTTTGCAACGTTCAGAAACTGGGATTGG

>V300080312L3C004R0311184620

CCAGAGGTAGGCCTTAAAATTGCACTACGGCGATACATCTTTT

>V300080312L3C003R0190347209

GGTAGAACCTTGCTTATCCACTGATTCTACCCACTGTGTCAAA

>V300080312L3C002R0170901678

CAAGAACTCCTGCTTGGATGTAGCTGCTTTCTCTGCTATTTCC

>V300080312L3C001R0220376569

ATTTTCAAATCTAACATAAAGTTAACACTTTCAGTGTTACTTG

>V300080312L3C004R0261260069

GGGAGAGTCGCGATTTGGAAAGCCCGTAATCGGTAACCTTCAG

>V300080312L3C005R0040904642

CTTGAGAATAGTTCAGCATAGATGGCGACGATATTGTTGACAT

>V300080312L3C004R0520307275

GGCCAGGAGGATGTCCAGCGAAGAGAGAAAGCCGACAGAGAAC

>V300080312L3C004R0151283327

GCGATCATTTAGCGGAAGAAGAGTCGTCTGTTCGTTATTCATG

>V300080312L3C005R0690934589

GAGTCGTCTGTTCGTTATTCATGACTTCCGCCAACGTATCATT

>V300080312L3C004R0340866476

ATTGACGACTAAGGTCGGACCAGTTTTGATGGTTTCATTCGTG

>V300080312L3C002R0670447725

ACATGAGAGAGCAACGTACCCGAAGATACATGTAAAACACAGG

>V300080312L3C005R0490657832

CAGAAGCGTAGGCCTTGATTGCACGGCATTTGTAAAACTTTCG

>V300080312L3C003R0640535525

TATCGATCGACATGAGTGCACCGTATCTTATATATTCTTGTAC

>V300080312L3C004R0040251810

TTTGAAGCTTGAGATATCGGATCCTGCATTCGCGCGAGGTTCG

>V300080312L3C002R0030961795

AAATTTCGTTAATAACAGACGCTCAAGACACTGATGGTCCAGG

>V300080312L3C006R0010387419

TATAAAGTAAATTTGAAGAGAACCAAATTGATATCTACGAAGA

>V300080312L3C005R0620095748

CCGGCTTTAAACTGCGTCGCGGCGTGTACAAGTCCTTAAGGGT

>V300080312L3C005R0700314146

CCAAAGCCATTCAACGTGGGTGACACGCGCCAAGTGACGATCG

>V300080312L3C002R0241193901

CATTCAACGTGGGTGACACGCGCCAAGTGACGATCGATTACGC

>V300080312L3C001R0130294397

CAAGAAAGGCACACACTCCGCTCTAAAGTCTGTCAAGGAACTT

>V300080312L3C005R0621337299

AGCCGAAAATCAAGCGCAAGCTTAACAGGCTGGCATCTATGGG

>V300080312L3C002R0530387088

TCCAGAAAGAAATGCCAGGCGCATGACTTTGGCTCCTCAAACA

>V300080312L3C002R0700679502

AAGACACCACCAAAACTTCCAAAGCATTTGGAAGAACTTGGAG

>V300080312L3C004R0350129123

AAATATGTTTTACAACGAGAATCAGGAATAAAGTCAACTCTAA

>V300080312L3C002R0640209782

ACAAACAGAAAATCTACTGGATATTGATTTCCCTAGTCAAGTC

>V300080312L3C005R0370997647

GATCCTTTCAAATCATCACCCTTATCACCACGCAAGACACCGG

>V300080312L3C005R0090901219

ATACGCTTAAACCGTAATATCCAATACAGTTTTCGATGCTGTA

>V300080312L3C003R0460427894

TATGGTTTAAGGAAAAAGACTTTATCGAATTATTTTTCAAGTT

>V300080312L3C004R0300153512

CAAGAGTTCCTCGGTGGGCGGGTCGGTTGCAAGTGCGAGAGCT

>V300080312L3C004R0040275658

AAGACCGATTTTTGATTCTCGCTGATAACAGAAGAGAGGAAGG

>V300080312L3C002R0550627366

TCGCGTAGCTTTGGATAATGCGCTCCAGTCGGGATCGATCTTC

>V300080312L3C001R0330968831

AATTTTGCATTGACATGATCATGGAGCAGTGCTGCAGGAGAAT

>V300080312L3C001R0690530408

AACAATGTCACCTGGTTCCAGATGGAGAATTTCGCCAACTTGG

>V300080312L3C001R0530341767

CATTTGGATGACACCTTCGCGCGTTGCCTAAATAATCCAATCA

>V300080312L3C002R0260357416

TGATTTGACTTCGGGCAGGATATTACTGCCAAAGACGGCAGCT

>V300080312L3C001R0090331445

TTAAATCTTTCTGCTTACGAGAAGAAATTGAGGGTACAACTCG

>V300080312L3C001R0151110419

AGCCAATTCTAGCACAAACAGCGAAGACTATGATTATGTGCCG

>V300080312L3C002R0670309440

TCTTCGCATAGTGCCATATGTCGGCATCCTGTATCTCTGCTCA

>V300080312L3C001R0410466889

AAGTTGTGACTAACTGGAGCTCTACATAGCACCCTGACTTTAG

>V300080312L3C002R0240406919

ATGAGCACAATGTTCCTACTGAGGGGCCATTGATCGTGTAAGC

>V300080312L3C001R0490008385

GGTACACTCTTTTTGGTGGCACTTTTTTTGGTTTCTTTGCGTC

>V300080312L3C003R0410238827

CAAAAAGTCATCGGTACTAAGATAAAGAGCAGCGATGTGCTGG

>V300080312L3C003R0500975901

GCAAACGAGAAACAGCATCTCCCACCAAATTAGTCCCCGAACA

>V300080312L3C005R0691243130

AGAAAAAAAGATAAAGCCGAATGTATGTTTGGGATGCGCAGCG

>V300080312L3C001R0040627177

TAAACTGTTCACCATGTGTACTGCTTCTCTCGGAAACACCACC

>V300080312L3C006R0711068779

AGCGCCGCAACATGCTCCTTCTAGTCCCGGTACGTTTCATGGG

>V300080312L3C003R0220326361

CACACAGGATACTTGGCTGGTGTCAAGAAGATCCTCGACAACA

>V300080312L3C005R0101184103

CCATCCTTGTAATATTTTTGAAAATGCACACGTAGTCATAATA

>V300080312L3C004R0111324183

AATATTCAGTACATAGGGATAATGGGAATCAGGAGAAGAAACA

>V300080312L3C001R0310206943

GCGTTTGACAATGACTTCATTGTCGATCGAAATCGGAAAGCAA

>V300080312L3C005R0550313279

TGCTTTGATGGGTGAAGAATGAACATTGTTGGATAATCAGCTA

>V300080312L3C001R0040574652

CATAGAAACTTCAGGAAGTTTGTACAGTGGTTCCAACAAGCAA

>V300080312L3C006R0260583622

TAATCGGCACGACGGTCGAAAGACAGATATTGTATATCTTCCC

>V300080312L3C004R0121222219

AGTAATTGGATGCATTACAGGAATGGAAGATCTTTCCGGAGGT

>V300080312L3C005R0390152397

CACACTAATAGTCAGTCATATCAAACATCGCTGTGATAGCCAC

>V300080312L3C006R0610404985

GATAGGCCGCCGATGACATAATTTTTATAATTACCTAGCAATG

>V300080312L3C005R0390748847

TAGCTCCGAGTTGACTTGTGATAATTGACTTTCTGGGCAGCAA

>V300080312L3C001R0240150164

CCACATTTGGCAACTGTCTTTGAAAATCTCTTTCTAACTATCA

>V300080312L3C004R0260780877

CCATAATTTCAAGGCCTCGGTTACAGGAATTTGCGTAAAAATA

>V300080312L3C004R0560932872

GGCCAAGCGACTTTATTCTACAATTTCGGATCAGCGAGAGCCT

>V300080312L3C005R0240284325

GATGTATGGGAATTTCCGCAAAAGGAAGCCACTTCCGACGGAA

>V300080312L3C002R0280108543

AGAACTGGTCCAAAGTAAAGAGTACCGATGAAACCGAGGCCGA

>V300080312L3C005R0080626515

GCAAGACCATAGACAACGGAGAGAAGGCTTTGAAGACGTGGTC

>V300080312L3C001R0151354560

CTTCATATCATCATCATGGAAGCCTTGTGGGACCATTTTCGGG

>V300080312L3C006R0480264273

GAAACAGTTTCTTCAAAACAGGTGTACGTAGTAGGCCTGATCT

>V300080312L3C003R0560355310

CATCAACAATAAGAATTACATGCAGACTTCGAGAAATATAATT

>V300080312L3C006R0280930829

CAAAACAACAAAAAGGAATTTTTTCCTGCATTCTACAAGCACT

>V300080312L3C001R0540863010

CGCAACCTTTCATTGCTTTGTCGGTCGCGCCCGCAGTACTGCG

>V300080312L3C001R0510426837

GCAAGGTTTCGGTGCTGGTGGTATCATGTCCATGCTTTTCATC

>V300080312L3C005R0660938979

TTCGGTGCTGGTGGTATCATGTCCATGCTTTTCATCATCATTA

>V300080312L3C001R0520502271

TTAGCAGGTAAGCGTTCGTGACGCAAACCATCGTAAAAGTTCA

>V300080312L3C002R0410498206

TCGTAAAAGTTCAAAGCTGTCGACAAGGCTGGAACGGGAATAC

>V300080312L3C004R0031233856

TCGCAGCAAGGTTGACCATCAGGTGCTTTGGCGGCTATAGCTT

>V300080312L3C003R0520496757

GCACAACTCTTCAATGCTATGTGCACCAATGATATTAGTGTCT

>V300080312L3C002R0300132277

TGACGACAGTCTCTGCAGCAATGAGCAGCAGGCTCAGCGTACA

>V300080312L3C006R0591319321

GTCTCCATTAGCGACAGCGAGGGATAAAGCTTCGGAAACGTTG

>V300080312L3C006R0211360153

GTAGTCAATATCAATGCAAACAGTGTAAAACTGGCACCCATCT

>V300080312L3C003R0721338183

TCCCATCCTCTTCATCCTGCTGCACAATGTCCACGACGTCTTG

>V300080312L3C005R0301293498

ATTATTTACGTTTGCTGGACTCACTGTTGGATCGCTTGGAAGA

>V300080312L3C004R0591099492

GATCCTTTCAATGATGTATCTTTTGCATCTGGAGCTCTCGTAT

>V300080312L3C001R0090166046

CCAATATTCACTATTTTCTGATTCAATAGATTTCGCCATTCGC

>V300080312L3C005R0601280839

CCGACACAGCTGCATCCGCCGGAAGCATATGCTAGACCAGGTG

>V300080312L3C001R0490981366

CACCTCCGTTGATGACAATTTCTCAGTTACTTTCTGATGCGCC

>V300080312L3C003R0140634067

GAATATGAAAAGAAGCGAACAGTAAGCTTTAAAACTTGTAAAT

>V300080312L3C002R0150513647

CGAAGCTCAGCGACAAGCTCGCAAGCTTAATTTCTTGATCACC

>V300080312L3C001R0400183583

ATTGGTCGAAAGATTAAAGCTGATACAGGCGAGGATGATGAGA

>V300080312L3C004R0381178499

GCAAAGATGCACCTTTTCATGTTGTCATAACCAGTTATCAGCT

>V300080312L3C006R0200433906

TCTGTTTGAACCATCAATCCGCGCCTGTTTCTTTGGTACTCCA

>V300080312L3C001R0120981082

ATTTTGGATTCCGGCAAGTTGGCAACATTGGACCGTTTGCTTG

>V300080312L3C002R0240596210

TGTTGGATGATGAAGGTAAGGATCTGATAGAAAACCGAGTTTA

>V300080312L3C005R0281003270

AGCATGTGGTCAGTTGCCTGGTGTGGCGAACAAAAGAAAGTTT

>V300080312L3C002R0360749001

CAAGCCGCGGTGCATAGTTCAGATGGTGAGCATTAAGAACATT

>V300080312L3C003R0390777814

TTCTAGATACAGCGAATAATGATCTAAGCAACTGGTGCTATGG

>V300080312L3C005R0190840932

TGATATTCACTGAAAAATGTTTACAACGCATGCTATTGGAGTA

>V300080312L3C002R0651165392

GTCGAGTACTGGGAAAAATATGCCCTGCATGCCTTTGACAACA

>V300080312L3C002R0520990251

TATTGTCCTTTCTAGATTGAAGAAAAACACAAAATTAATTGTA

>V300080312L3C006R0280365154

AAGTGCTGCAGAGTGGCCTCTTGAAAATCGTCGCGTAGAAATG

>V300080312L3C003R0250074012

GATGTCACATATCAAATTGTATGCTGAAAGTTTGAATGCATTG

>V300080312L3C003R0680402648

TCGTAATAGCAGAAGTGTAATTTCATATTATTCGTGAATCGGG

>V300080312L3C004R0020595286

GCATGACCCTTTTACTACCGGCGTTGTTTTGCTTTCTCTCAGT

>V300080312L3C005R0591008985

TCCTCGCAAGGTGACCCCGGAGGAGATATTAAAAAATTGCGTT

>V300080312L3C003R0470769854

AAGATATCTTTTATAGACTCTACGGCTGTCTTACTATGAAAGA

>V300080312L3C001R0050316136

CAATTTTCAGCATTTGCGCGCTGTGCGGCCTTGAAATACATAT

>V300080312L3C003R0511158930

CTGCCTTGTGCACAGGACTCAACTGATAATGCGCATAAGCTGA

>V300080312L3C002R0690635280

CGCTTACCGTAATATCTGCGCCTAATCACCCCAAATTAATATC

>V300080312L3C002R0070647835

CTAAATCCTATTTCAACGTCAGTATCTTAACGGATTTATAAGC

>V300080312L3C003R0250884786

TAAATCCTATTTCAACGTCAGTATTTTAACGGATTTATAAGCG

>V300080312L3C001R0140483537

TCGGTTGCGTCTGCTTCGGTTGTATCTGCTGCCAATTTTGACT

>V300080312L3C005R0580802728

TGTATCTGCTGCCAATTTTGACTGCTGCTTCGCAGCAACAGCG

>V300080312L3C002R0441263574

TCCGTCCTTCATTCATCCATCCAACATCCGTCGATCGCCGTAT

>V300080312L3C006R0090060825

GGCAGATAGAAGAGATGCAGGGCTTTATGATGACACCAGCTTT

>V300080312L3C005R0430354486

GGACTGCACAAAGAAAGGCATTGTTTTGATCCTTGCCTCGGCG

>V300080312L3C001R0530336047

TACAATATATCCACCATTGACAATAACGTCGAAAGTTAAAATG

>V300080312L3C004R0621305709

TACTGCTGAAACTATCGTATGTTTTGACCGAACATTATCTATA

>V300080312L3C006R0300150668

TGTTATTTTCAAAGCAAACACGCCGAAATCGACGCTTATTTCC

>V300080312L3C003R0520764403

GTAGGGTCGCTTTGTCTGTGGGTTGACGCAATTATCGGTGACG

>V300080312L3C001R0351060097

TCATAATTACTCACTTCTTGGATAACCTTGTCTGTATCATCTG

>V300080312L3C004R0650775234

CATCTGTCTTGAAACATTTCATCAAATCGTCTTTGCTTGCCAC

>V300080312L3C005R0140153870

GAAAACTATGTATCTAATTACATATCATATACACACGCATTGA

>V300080312L3C003R0481128149

GCCGCCGTGTCCAAGACGGCTGCCGCCCCTATCGAGCGTGTCA

>V300080312L3C003R0640209804

ACATTTGGCGTTGCCTCTGTCTAGATAGCTCAAATTCACGTGC

>V300080312L3C006R0721120365

GTGCAGGTCCTCTATTCCTTCAAGCACATTTTCATCTTCCTCA

>V300080312L3C002R0420370907

AGCAAACGCACTCGCAAGGTCTTTATCGGGATCTCTTGCAACA

>V300080312L3C002R0720917551

CGATGAACATGAAGCGATCAGATGTATGTAGGATTGGTTCCAG

>V300080312L3C005R0080515050

ATCTTTTTGGCTGGAGCTCTAAGCTGAGTGCCACTTTTTATTG

>V300080312L3C005R0410610330

TGAGAAGGAAATGGGATGGATGTTACGGTGATATGACGCCAAG

>V300080312L3C006R0590857889

ATGGGATGGATGTTACGGTGATATGACGCCAAGATAATCTTCC

>V300080312L3C005R0401160772

GGCGTGCCGCGAGTGTCGAATGGATGTTCCCTAGCAACCTGTC

>V300080312L3C002R0230716007

CACCCTTGTACGGCTTGAAAACAATGGCTTGATATTTTACATT

>V300080312L3C002R0010471079

AGAGGCGGCGCAGGCGCAGAGAGGCAAGATGAAGGAGTTTCGC

>V300080312L3C002R0241142956

CGGATTTGCAGATAAGCATTCGAAAGCGGTTTGCTGTATATTA

>V300080312L3C002R0301285754

TTCCACTAGTCATTCAAGAGCTACATCAAAGGATTGTCCTTAC

>V300080312L3C006R0301117984

ATACAGTGAATACGTGCGATATCTTTTCTTACACTTGGAAACG

>V300080312L3C001R0070205873

ATCTTGCTTCCACATTCCATTGCTTCCTACTGTCGGGGTGGCC

>V300080312L3C004R0081137962

AAGCTCGCAGCCATTTGGCCTCCATTTCTAGTTCTTGGACGCG

>V300080312L3C001R0680009488

GTCGTGGTTGAGACAGATGTAGTTGGTTGCATTGCCCAGGATG

>V300080312L3C003R0690082187

CCAAGGCAGCAGATGTGGTCGATGCGCAAGCAAAGTCTTACGT

>V300080312L3C004R0580944289

GTTTGTTCCGTGTTTGCTGTTGTCTATGCTTTTCCTTCTCCAA

>V300080312L3C003R0511076358

GAACCGGTAACAGGGCAATAAGGTCCAGCACATTTGAAGAATT

>V300080312L3C003R0331264887

AATAGCAGCCCAGCAAAGCACCGCAGGATGAGGATATTAGAGT

>V300080312L3C006R0350242560

ACTCGCTTGCTGCCCTTGGACAAACATGCCGTTTGTTCTACAC

>V300080312L3C004R0230688773

TCCAAATTATACTCCGTTTGTCACGCCTGTGGTATCAATTGGG

>V300080312L3C006R0051048901

GTCACGCCTGTGGTATCAATTGGGACCCGACTTTACATGTTTG

>V300080312L3C001R0680568853

ACATGGACTTGGTACAAATACGATCATCCCGAGGCTTACAGCT

>V300080312L3C005R0020476249

AAACTTTGCCCTGAGAGCCTAACTTACTTCAATACCATCAGTG

>V300080312L3C005R0531088290

GAGCTTTGTTGATGTTATCTATTTGAATTGGTCTTCAGTGACG

>V300080312L3C004R0091321001

ACCAGTTAAAGGAAGGTGACAAGTTGTAGCACAAAAGAGTTCC

>V300080312L3C002R0431027090

TGGAGCGAGAAATGGCAGCATGAGTGCCTCCTTGATAGCGATA

>V300080312L3C005R0500962466

TAGAAGCAGTGGAAGAGCACTGCCAACCACTCACGTATCTATG

>V300080312L3C003R0660779908

GTGAAGATTTGTAAAATCCGAGTTACATACACAGTGTCAAGGT

>V300080312L3C005R0570684098

GGGACCCTTACGATCGAATTAATCGTTACTCCTCTCTGCATGT

>V300080312L3C004R0330692932

TGGGATGCAGACGAAGAGTTTCTGTTGATCGAGGCGGCAGAGA

>V300080312L3C001R0460669623

CCCGCTATATTCTGTGGACAGCGAATGACAAACATGACTTGTC

>V300080312L3C003R0150400962

TGAGCTCCTGGGCAGTGAAACGGTGTTGGAGCGGTACTTTTAT

>V300080312L3C001R0710543030

TGCTATGCACGTATGTTGTGCAAGGCCGACCAAAGTCGTGCTT

>V300080312L3C004R0261193803

CAATCTTTGTGCATCCTATTAGAAACACAATTGGTGATTAACT

>V300080312L3C003R0130258012

TGCTAGCGCCATGGTAAGTTGGAACTCGTGTTGCAGGAGATTC

>V300080312L3C002R0680834805

AGGATGTTAATCATGCAATTCTTTTCCCCAGGCTTAGGCTTGT

>V300080312L3C002R0011309927

CCCCAGGCTTAGGCTTGTATACTTGCAGATGCTGGACAAGAGT

>V300080312L3C001R0080231575

TATCATTAATCTTCGTGCGTATCTCTCGGTTCAAAAGAATTGA

>V300080312L3C002R0530593685

AAGTGGATTCAGGGCGAGCGAGATAAAGAATAAATGGTTTCGG

>V300080312L3C006R0340746850

AAAACAAAAGAGAACGCACTAGGGTTATTATGCGGATAATTAT

>V300080312L3C002R0710717783

TCGACAATATATAAATAAATTTTCACAATACCTTTGGCAAATT

>V300080312L3C004R0360686793

TTTCGTAGAACGTGTAAAAAATGACACCCGCTGATCGATTGGA

>V300080312L3C005R0680698534

TCCTTGATATTGCCCATCGTCATGACGTTGAGACCCGCAGAGT

>V300080312L3C001R0370893697

TCATTGGTTTGGATGCATCATCGTGTATTATCTAGTTCTTACC

>V300080312L3C002R0321100413

TTCTTCCTTCAAGATCTTATCCATCTGTCGATAGTCATTAACA

>V300080312L3C006R0250222192

TATGAAGCTCCGGTCAGAGATATCCTCTTCTTTGTACAGTTTC

>V300080312L3C002R0330336113

CACGCGATGAACAAATGTCAAAATCAGGGGCCGATTGAAAGAA

>V300080312L3C003R0550054164

GAAGTTGTTCGAGATGACAATACCTTTGGCGGCGACAATATTG

>V300080312L3C001R0481210057

TTTCCTGCATAGCATACAAAATGTTATTCACGCTGCCTGCTTT

>V300080312L3C004R0230358449

CACGCTGCCTGCTTTTTGGTTAACTTCTGACTATTTTGTCTGT

>V300080312L3C005R0591356306

ATTTTAATAACAAGGGAACAATAAATAAAAGGAAAGAAAATGA

>V300080312L3C004R0180446476

TAGAAGAAATCCAAGCAATTGCCACGGTCGGCCGAGCAGGGGA

>V300080312L3C003R0690835893

ACATCGATCCCAACTCGGCTGTATATGCGACTGACGAATACGG

>V300080312L3C005R0280044661

TGCTTGCAAATATTGTCCGCAAAGAATGAGTAAGAGCATCTAA

>V300080312L3C004R0590904974

GATTTTAATCTCCCCAGATGGAGACATTACCGTTACAAACGAC

>V300080312L3C003R0640075837

CCCCTTTGGAGTCGCTTATAAAACCTATAAATTATCACAATCA

>V300080312L3C004R0150034666

TCATTACTTTGAGCTCGTATTGCTGCGCAAACGTGCTCGGACA

>V300080312L3C004R0291256369

TAACCTGCAGGATACGAAACGGCTCAGCAGAAAGGGTTAAATA

>V300080312L3C001R0100659088

GCAGTAGTCGGCGCAGCACCTCCTCCTCCCCCCCCACCACCGC

>V300080312L3C002R0261266504

GTCCGACGTGCTTCAATGTTTCGAGGCAACGGGGCAGGCGATT

>V300080312L3C002R0221295508

CAACTTTGCAAACTATCGGCTGATGTGTGTATTTGGTAGATGC

>V300080312L3C003R0370054743

AAGTGGAAACAATCAAACAACAGCTGTCAGATATGCAAGTACA

>V300080312L3C004R0130791562

TATCAACGCAGCATCAGAGTGCTACAGTTTCTACCGATATGCT

>V300080312L3C006R0030140163

ACGCAGCATCAGAGTGCTACAGTTTCTACCGATACGCTTGCAA

>V300080312L3C002R0380320990

ATTGTTACAGTCCCCAGCATCATATTCGAATTGACAAGCACAA

>V300080312L3C002R0221154987

ATCTTTGCAGTAAAGCCACGGGTGATACTTGTCTACGGGTGGA

>V300080312L3C003R0570110971

TCATTTCGGTTCTTTCCCCGAAACCATGGGGACACAAAAAAGG

>V300080312L3C004R0260371460

GGTTTCCAAACACTTTGCAAACTTCTTCTCGTCTTCATCTTCA

>V300080312L3C001R0150014543

AAAAAAGCGAAACTCAATATTTTCGAATTACAGCAGCAGCCAA

>V300080312L3C005R0460325000

CGCTCAGTCGACGAGCGTAGCAGTGCTTCGTCGTCGGATGAGT

>V300080312L3C006R0040561970

CCAAGAACAACCAGCAGGAGCGTGAGAACCCGTAACATTTGGC

>V300080312L3C004R0240069878

GGCATTGACATTAAATTGGGATCCGACGTTTTCTTTTTTTTTT

>V300080312L3C001R0550857864

TTCTCGAGGAAGGATACCAAAGGTATGTTGTTGGTGTTTACTT

>V300080312L3C006R0360226089

CAACTTGGAGACACGGCAAACATGGCTAGCTGGGCAATCCATT

>V300080312L3C004R0050480790

ATTGCTTCCTCTTTTAGGTGAAAATTGCATTTGGTAAATATCC

>V300080312L3C001R0160293409

GCAAAATTCAACAGTCAATATTCTTGAGAATGCATATTTTGCT

>V300080312L3C003R0680370454

GGCGCAATTAGATTGTTATATAACACTAATTAACTCCAAACTG

>V300080312L3C004R0570499020

TGAACGAGCAATTGCATCCAAAACCGCAACCGGGGCAAGAGCC

>V300080312L3C004R0110224593

AAATCCAGGTATCTCTAGGTCGCGTCAATCGTTGGTATCATTC

>V300080312L3C002R0220325978

GCATTGCTGGACGACGCATTCAACTGCATCGTTTCACACGGTG

>V300080312L3C001R0671093739

CAAAGCTGGTAAGCTGATCAGCGCTGACTCTTGATAAAATCTG

>V300080312L3C006R0050649522

AAGCCATCGAAACCGGCCGCAGCGTCCGGGCCGACTACAAACT

>V300080312L3C005R0330702778

TTGGAGCGAATTTTGGGCGTTTTCTGATCAGCACTTTATTTTA

>V300080312L3C004R0070751469

CTTTTGCGCCATTACTTAGTATACGGTACGTGAAGAAACCTCC

>V300080312L3C004R0530019046

CAGGAAAACTTGATTGCAAGCTGCGATATGGATAATATTGTTC

>V300080312L3C002R0250757268

AAGGACAATCAATGAACCCGCTCAAGATCAGCAACAACACTTG

>V300080312L3C005R0500378695

GAATTTTCGGTAAAGCAAATACTTGCCATTCAAATCACTTGAC

>V300080312L3C005R0030643424

GGAGATGTAGGGCTATAGCTTGGAGACGTCGGGCTGTAGCTAG

>V300080312L3C003R0480750436

AATATCCAGGCGAAGCACCATATGGGCTTGTAGGGGAATAACC

>V300080312L3C004R0260867431

CTTTCTCGTAGCTTCAATACCCAGGACTTCCATAATTTCCACC

>V300080312L3C005R0430790070

CACCTTCCTGACACATGACTTCTTGCAAATTATTACCGTCGGT

>V300080312L3C005R0280122986

GTGTGTTCCAGGCAAACTTGAACTTCTTTCGCCAGTTCAATAC

>V300080312L3C006R0110828970

TAACCTTGTTTGGATTGCTTACCAGTCAAAGGCCTGAGAATTG

>V300080312L3C002R0660273772

ATCGCCAAGGGAGTTTCGGACAGTACCGTCGTATTTGACCATG

>V300080312L3C002R0410062986

CGAGGAACGCCGACTTCGTCTAATTCAAGGTTTGGATCACCTG

>V300080312L3C002R0011101150

CTCTGATAGATTTAAGCTAAAATTGAATGAGTTAATGAATTTC

>V300080312L3C002R0550065016

ATATCCTGATCCGAGATATTCTTCAAAATTTGCAGCACTCGGC

>V300080312L3C004R0661198684

CTCAAACGGGCTTTAGGATCGCGGATCTTTCGCGCTCTGACAA

>V300080312L3C001R0710888055

CAGCTGGAAAGCAAGCGGCATTGAATAAAGTAGATACAAGCAA

>V300080312L3C005R0051238306

CCTCTAACTAATAATCTAAATAGCTTCTATCCTCTTGACTCCA

>V300080312L3C003R0080686730

CCTATGTCTGCTCTAACATTATCTGTCGAGGATAACGTTCAAA

>V300080312L3C003R0480286893

GGAAATGATAGATTGCTTTCTTCTGAATGTACTTCATTCTTTT

>V300080312L3C002R0180929221

CAGCCCCAGATGCTAATGATGAATCCTATAGACGCGTCGAGCG

>V300080312L3C001R0240724672

GTTTTATCCTAACATCGTTCAGTTCAAATTATCGAACTGCCTT

>V300080312L3C001R0310999695

TATATTATGACATCTAGTATGACCAAAGTAAACAGAATATGTC

>V300080312L3C001R0581026582

AAGGGCACTATCTTTGAAGAAGAGTACCTTGTTAATTCCCTCA

>V300080312L3C005R0680707322

ACGAAGTTTGGTGAATTTTTGGAATCTCTTGGATCCGCTGTGG

>V300080312L3C006R0630562641

GGAAGCACCAACTGGTATCAAGCAATCTACTCCTGTTGCAAGC

>V300080312L3C003R0610886993

TATCACGACGCTGTACCCACCAAAGTATGTAGGTAGGAGCCAC

>V300080312L3C002R0410424895

TTCAATTTTATCGCCTAGATACGATAACCCGAAGTTCAATTCG

>V300080312L3C001R0340889066

CTATGAACTTGAAATGAGTCCATTGGCTGATGTACACATTGAA

>V300080312L3C001R0290480109

TTCAGAGATGAGCAATTGTATCAAGTATGCATAGATGGTAGCT

>V300080312L3C002R0191060401

AGAACTTTCTAAAGCAAGACTCACGCCAAGCTCTTCGTCTATA

>V300080312L3C002R0590144641

CGACTCATTTCTACCCTAGAGAACCGATGCCATTCGACTTTAG

>V300080312L3C002R0400213456

CAAGTGAGCAGGAGCGACGATTGGAGATAGCAGGTAATCTGAT

>V300080312L3C006R0541163459

GTAATAATACGGTCATTGAAGAAGCTCATACGACTAGTCATTG

>V300080312L3C003R0171306257

AGTGTCACCGCTGCTGTGAGGTTACCACCACTAACATCCAATC

>V300080312L3C001R0590441296

AATAGTTAGAGGTGCTCGCAGAAGCTTGGAGACAAATGAAAGG

>V300080312L3C006R0720297623

TATGTGCCTCAGCCATGCGGGTTTCAATGGCATCGCAGTGAAG

>V300080312L3C005R0610560889

GATCAAATTCCTCCAAGATGAAGATAATAGGCAATGTGGACTT

>V300080312L3C003R0720665946

ATGTTTAGCATGCGTGTTCTGTGTAAGTCGTGTTTTGGACTTC

>V300080312L3C006R0680952016

CCACTGCTGCGCAAAGTACAATTGCACAGTGTACTTCATACCC

>V300080312L3C002R0250783814

ATTGGATGGGGTGTACCCGCTTCAATTATGAGTATCGCTCTTG

>V300080312L3C002R0401037001

GATCAGAACAGTTGCGTCTCCGTTATTAGTGGACATTTGCCTC

>V300080312L3C006R0200726682

TATATGCAAAATACTTTGATCGGCACATCTTGGCGGTGCGGCC

>V300080312L3C005R0470440607

AACAATAGGACCATATCGCCACGGCAGATATTTCTGAGCAATT

>V300080312L3C004R0500984558

TCTCTCGTACCAATCGCCAGTTGCCCTTTCCAAATTCTTCCAC

>V300080312L3C005R0550102333

GTCGTCTTCTAAAAGGTAGTTCATATGTTTTTGTAATCTATTC

>V300080312L3C002R0330432805

ATAATTTTCGTTATTTCTCGGTTGTATGTCGTTCTTACCACTT

>V300080312L3C002R0100185163

TAATGGAATGAAAATCGTGCTTTTTGAGCACAGAAATAGTACC

>V300080312L3C005R0270841225

AGAAGAAAGAAAGTTTTATTCATGTACATTGCGATCTGCTTAC

>V300080312L3C002R0100357572

CGTTTGTACACGATGACACCGACGGCGGAGGGGGTGACGTTGT

>V300080312L3C001R0400845666

ACTATACCATGTTTTTGCAATAGGTTTGTCTATGAGTACTTAC

>V300080312L3C003R0290251723

TGCCGTCTTCGCCACACAAACCACCAATATCTTCATCCTCCCA

>V300080312L3C006R0240211883

GTGAATTCTCTGTCGCATTTTATGGTGTTCCTGAGATCGCTCG

>V300080312L3C003R0610901897

GAGCAAAGGCTGGTGACAAATGTATCTTCACCGGCACGTTGAT

>V300080312L3C005R0150107580

AATGTCTTTGGTAGGTACCTCAGTACTTGTACACATGTGTAAG

>V300080312L3C004R0630778849

GTCTTCGGATCGATGACACTGCTGCCGAAGAAGAACAGGGAAG

>V300080312L3C002R0330802654

GCGAACTGATCCAGTGGTACCTCGAACAACGAGAAAGCAGCGT

>V300080312L3C006R0621075768

CAAGAGACTGCGACGACGGACGCTTCTGGCGAAGATGATCCCA

>V300080312L3C002R0470383423

GAGACTGCGACGACGGACGCTTCTGGCGAAGATGATCCCATCA

>V300080312L3C003R0091108842

TACATTTGATGCAATTGAAAGCAAAGAAAGGTTACAGGCAGTC

>V300080312L3C003R0701192615

ATGACTCGATACCAGTGCATTCCAGAGAGCATTCCACACCGTC

>V300080312L3C001R0300268619

TGAAGCCATCTGGCTGGGTGGGTAATGTATTTAGCGAGAAGAC

>V300080312L3C004R0370418806

GCATGGCTTGAGCTTACTGATCCTTTGATAAAGGTTTCAGCTC

>V300080312L3C002R0550512556

ATCGAGTATTACTTACATCAATGCGTCGACATCCGGGTTCGTT

>V300080312L3C005R0300527577

TAGAAAAATGACAAACGCAATGGTAAGGTCTAAGTTTACCACG

>V300080312L3C005R0190119259

CCCCGCCCATACACATGAGGTCATTAACTCATACCTGGCATTT

>V300080312L3C002R0210894475

GAGTTTCTAGGATAAACTTTATAACAACCGGACTCCTCCACAC

>V300080312L3C006R0340846788

ACAAATAGCCCTTCAAACTGTTATCCTATGTGTCTTTTCCAGA

>V300080312L3C002R0030726727

AGGAACCGAGTAATAAACAACATCGTAGGCAAATCAAACCTCA

>V300080312L3C006R0661391592

ATCAAGCAATACCAAGAACAGAAAAAGGAGGCCGAGAAATTTG

>V300080312L3C004R0221177123

TGTGGAAGCTCTTCCATGTTGAACAGCACACAGCAGCTCTGGA

>V300080312L3C005R0600725441

AGCATCAGGAATCTTTGAAGCTAGATACACAACGCATGCAGGA

>V300080312L3C005R0180064566

TGCATATTAGCATTCGCGAGACTGACCTGAACGAGAAATTGCA

>V300080312L3C002R0681090598

AAATACGATGTGGCAGTTGCAACGACATTAGGACGAAACATGG

>V300080312L3C004R0110499667

GTATGTTTCTAATTTCTTTTTGTTTTACGCGTAATTATATGTT

>V300080312L3C004R0241056910

GTGGTCTCATACCTGGTGGTCAAAGCGGCAACCAGGCAAGACC

>V300080312L3C004R0490078826

GTTGTTACCCAACAAAAGTTGCAGGATGTCCAAGCCGAAGGAA

>V300080312L3C001R0530554175

CTGCAGCTTCCTTTCGAGCGCGCTTAATATGCGCTTTTCGTTG

>V300080312L3C005R0610167752

TCGAATTTCTCAATCGCAGTATCGCGGAACGCAGTCATGACCT

>V300080312L3C003R0600175652

CGTCGAAGCATCCATGACATAAGCCATATTCTACGGTGCACGA

>V300080312L3C005R0590215639

GTGACATTGGGACTGAATAATAGATCCAACGAGTATTTACCTC

>V300080312L3C006R0031293556

GGTGGCCACGTCATGTACTGTTCTATGGTCATTGGCATTTGTG

>V300080312L3C004R0190931490

GGTGGATAGAGTGGTATCAGCCGCGTAGGCTGCATTATTAGTA

>V300080312L3C006R0550052915

CATGTCGAGTATAAGCAGAGTCGAAACCGGGTATCTTTTCATT

>V300080312L3C005R0310943332

GAACCCTCGCCGTTTCCTATGAGCCAGATACGGTTTCAGTAGA

>V300080312L3C001R0250710693

CTCCTGAAGGAATTGTAGGATCATCTTACATTTGATTTGGAAC

>V300080312L3C005R0251136295

AGCCTGATTTAGACACCGTTTGCTACAGGCGTTCGAAGGAAAC

>V300080312L3C004R0210210368

GGTTTGATTAATAATGTCAATACTTGCTTCATGAACGTTATCT

>V300080312L3C002R0450473807

TGTCTTGCTGACCGACCATAATACCCGTACATCTGAAATGATC

>V300080312L3C002R0541069493

GCGTAACGTATCCTTTGCCTTTGTAACCCTCAGAGGAAATACC

>V300080312L3C005R0551096170

TGCAGAGCTTTTGCAGTTCTCGTGGCGGTCGCGCGGAACATGG

>V300080312L3C002R0480131944

AGATTAGATATGAGACATTTTTACTTAATAGGTTTTAGATGTA

>V300080312L3C003R0210731523

AAAAGTCTTTGAATGCTTCTTTTACCTGCTAAATCATTATCCC

>V300080312L3C004R0440295176

CCTCAACCAACTCATCTACGCGGTACCAAGCAGCGCTCCTCTT

>V300080312L3C001R0541108233

ACCCAGCTACAAGCGTTAGAGGCTAAGCGCAGTCTACGTGAGG

>V300080312L3C004R0240928232

TATTTTGTGTACGCAAAGCATCTATTTCCTCCATCATTCCGAC

>V300080312L3C002R0510593792

TGTGCAGGCGATCATGCTTGCGCCGTGTTGAATGGATGAATAA

>V300080312L3C006R0681276618

GTTTTATCTGATCTCAAAATGATTCTATAGGACTGGGCTAATG

>V300080312L3C004R0010805538

TGAAGAAGATGATTAAGAAGGAATAGTCGCGAGAATTTATTCT

>V300080312L3C003R0630242219

GGGGATGCCACGCTGGCATAGCACGGCAAAGACCGAGCAGCGC

>V300080312L3C003R0050627580

GTACCAATCTCTTTAAAGTCGGTGTACTTGTTGTTAGGATCCA

>V300080312L3C006R0320788804

TGCCTAGAATATCTGGAATTTGGTAGAATCCAGGGGGACGCCA

>V300080312L3C002R0650420147

GTATTCATATTCATCGCCGCGCACACATAATCTCTCTCTGATT

>V300080312L3C001R0611087816

GTTGTCAAAGATTTGGACTTTCTGAGATCGCCCAGGGATGTCC

>V300080312L3C003R0250683778

GCCAGGTGGCGACGCTGAGTAATCAGGTTGCGGGTGAGCCATT

>V300080312L3C003R0130195104

CAAGGTAGACGTTTTGTGATATTTAAAAATACTATCTACTAAT

>V300080312L3C005R0181373398

TAAGTAACTCAACATCATCATATAAGCACTGGCGCATCTGATA

>V300080312L3C006R0330883223

GCCTTATATGCTGATGAGCTAACAATACCGTGTAGCGAGCACA

>V300080312L3C003R0520043332

GAAAAGCGAAAAGGAAGAATATTCTTGCATCTGGAGGAGTGAG

>V300080312L3C003R0401155846

ATCTGACGATCAGCCGCTGGTGAATCTTGCGGAGGTGCTTTAC

>V300080312L3C002R0090095760

GGTAAATGCTGTAATACAAAACATCGGCTATTGCTAACTTAAA

>V300080312L3C006R0150331482

TAATCGGAGGGGTCGCTTTCGTCTACTGATTGCGCTCTGAAGG

>V300080312L3C005R0271263575

CGGAGGGGTCGCTTTCGTCTACTGATTGCGCTCTGAAGGTTGG

>V300080312L3C001R0260061756

TGCAGAGAGAGGTGTATCGGCTTATACGACAGGTATAGCATTG

>V300080312L3C004R0370111924

GGTATAGCATTGTGAAGTCATCGTAGCGATCCATGGATACTCC

>V300080312L3C005R0090241750

GTGAAGTCATCGTAGCGATCCATGGATACTCCAACGACAGGAG

>V300080312L3C006R0700212166

AAAAGAGGATGGGCAGGGAAGGCAAGAAGGTAAAAACGACTGG

>V300080312L3C004R0601288422

CCTAACTAAATTACAATGGAAGCAATTTGTCATGAATTTGAGT

>V300080312L3C002R0480888059

TGAAAAAGGAAGACGTCGATAAAGCTCACAAGATTTTGTACCT

>V300080312L3C002R0180878397

GACGTCGCGGTCTTGAATGAAGTAGCATCGAAAATGATGGATA

>V300080312L3C005R0370110888

CATCCACCAGCACCCTTTTTACGTCAGATGGGTCAACGTTTTG

>V300080312L3C006R0450879740

GTAACGTTCTGTGCGAGCAGGACACCAAACTTTAGTCTCTACT

>V300080312L3C001R0410755558

CGACGAAAGACTCGCCATGTGCCCCAGCTGGAGAAACAAAGCT

>V300080312L3C006R0710371661

ACTCGCCATGTGCCCCAGCTGGAGAAACAAAGCTCTTCATTTG

>V300080312L3C002R0610516206

AAACAGAATTTGTATGGCTATCGTGGTGACCAAAAATCCCCGT

>V300080312L3C001R0661008550

TAATCTGTCGTATCTTTTACGATTCATGATCGATTGCAAGGTA

>V300080312L3C003R0080539844

ATATTTCCGGAGCCTGATAAAGACCCTGTTATCCAAATTGCAT

>V300080312L3C006R0050006542

AAGACCCTGTTATCCAAATTGCATCGGTCGTCAAAGTACAAGG

>V300080312L3C001R0151043841

AGACGTGGTCATCGGCTATAACATCTGCAACTTCGATTTTCCA

>V300080312L3C004R0660377050

CGATCATATACGCTTAACTCTGTTTGTGCTGAATTTCTTGGTA

>V300080312L3C001R0160372690

TAATGATAAAATTGGAAATGCATTGGATTGTTAAATTCCTTTC

>V300080312L3C005R0360371184

GAGCAATACGAAGGAGCAACCGTTATTGAGCCTGTCAAGGGTT

>V300080312L3C002R0451078461

ATTTTTATGTACTTAGTTCGTTGCTCTCTCCTAGATCAGCGCC

>V300080312L3C002R0211371543

TGCGGAGGAGGCATCTGACCGTCTAGAACGGTTTTCGATCGAA

>V300080312L3C003R0230665906

GCACGCAAGAGATCACAGAGCCCAGGGGAAGATGCCAGAGAGC

>V300080312L3C004R0510635447

TGCTATTTTGCTTTCACAAAGAGATACTCCCTCTGGTCTTGTC

>V300080312L3C005R0310694329

AAGAGATACTCCCTCTGGTCTTGTCCGGGCAAACAGCGCATCC

>V300080312L3C003R0411016184

GGATATGTTTCTACCGCAGATCGACGGACTGTTCTACAATACA

>V300080312L3C006R0230094837

GCTCCATGCAAAAGTAAAAGATTCTCGAACTGGAAAGCGTTCA

>V300080312L3C004R0380394069

TTGTGGAAGCTCTGGACGATGAAGAGCATGAATTTGTTTATGA

>V300080312L3C003R0301237702

GACATTTTGGGGATTGGAGAATTCGGCAAAGTCAAACTGGGCA

>V300080312L3C004R0050558279

TGAAGAGCGATGATCTGCTTGCTACATGCTGCGGGTCTCCGTG

>V300080312L3C002R0370354186

ACTACATCAAGGCGCAGGAGGTAGAAAATACAGCAAGAGCAGC

>V300080312L3C002R0540922090

AGCGCACGAAACATCTCCGCAGAAGAGACACAGGAGCTTCTGG

>V300080312L3C003R0011150611

TGTTTGGAGACCCTTCTGTGGACAATGGAGAGCAAGTTACATT

>V300080312L3C004R0490765514

TTTAGCGAATAGATAACGTTTGATTAAGCGGTTCACAGCTAAA

>V300080312L3C001R0390471300

CCGATGGCACGGAGCTTGCTCACATGCTACAAGCCGGGACAAG

>V300080312L3C001R0641283512

TGCTCAAAGCCTGCATAGATCACATTTACACTTACTCCAGTCA

>V300080312L3C004R0011179017

GGGCTGCTTTCGAGATCCTTATTGCAAATATCTACATGACTGT

>V300080312L3C004R0241333515

CGCCTGATGAGCATGTACGATGATCTCTTTGGACAGTTCAACC

>V300080312L3C005R0210805021

TGATGCTAGATGTACATTATTATCCAAAATGGCCGAAAGTAGA

>V300080312L3C006R0200269865

TGATAAATGGCGGCATGGCGAGCAGTCCAAGCCAACGCAGCAT

>V300080312L3C005R0560069663

AAAGGATCTATCGTGCCGTCCTTGTAACGTTGTCGTACTGATC

>V300080312L3C006R0540471001

TCCGTCATAGGGAGACATAGCAAGCATTCTGTATATATATCCA

>V300080312L3C006R0080203966

GAGAGGAGGAGTCTGAGTCTCGACTGCTTTTGCTGCACTGCTC

>V300080312L3C004R0390090431

GTCGCCGAGCCGTCGTAAGTGCATTCAGCTAGAAAATATACGG

>V300080312L3C004R0710863946

CCTCGGACACAACATCTAAGAATCGTTCGAAATTATCTAGTGT

>V300080312L3C004R0550798104

GGACAATGCTCTAGAAAAGGTTTTGTAAACATATCCCTTTGTT

>V300080312L3C002R0391146464

GACGACCTGTTCCTCGCCGCCTTCCCTTGGGCTTAATTTTGCT

>V300080312L3C003R0231054121

AACTTCTGGATCACATCAATAACCGGGTCACCGCCAAGAATCA

>V300080312L3C003R0370268555

GTCTTTGTCACAGGCATCTACAACCCTAAGGCTGTTGGTGATT

>V300080312L3C004R0460720762

TTTCCGTGGCCATCGATCAACATCCAACATAAACATGCTTGCA

>V300080312L3C004R0201319542

TGCACCATAAAATGGGCACGGAAGATCTCATCTGATCGATCAG

>V300080312L3C005R0401295958

TTAAGCTTCCTCGTCTGCTATCCATTCTGTCACTAACCGGAGG

>V300080312L3C003R0470070239

TACGGATATCTTCGAAATGGCCCTCCTACAGGATACAGCTGCA

>V300080312L3C004R0421315429

GGCGTGCTGATGAAGCTGAAAACTGGTACATTGCTTGTCTGCA

>V300080312L3C004R0700277723

ACTTAAATACATTCTGTTGTTATAGTAGAGGGCAGAGTGTAGT

>V300080312L3C006R0420378304

GCCGTGGAAGCCAAAATGTCGAAGATATCCGGTCTTTGAAAAT

>V300080312L3C003R0570584183

GGAATAGAATTACAAGCACAGCATCAAGAAGTATCGGTTGAAT

>V300080312L3C003R0701401233

ATTATTCTGAATCAATTAGCTAAAGAGTCTTAAAGAAAGTTGA

>V300080312L3C005R0460892879

CCAGGGAGCTTGAAGAGATGTATGATCGGCATATTAGAAAATG

>V300080312L3C001R0071071872

CGTGATTTGAGGTATAATAGCGCTTCAGGTTGCTATTTGTGAT

>V300080312L3C003R0300988343

TAAATGCTGTTCTGGATCTGCGTCGGCGACGTTTTGTGAGACA

>V300080312L3C002R0060348088

GTACTGACCACACTGGTCCTGAAGCTTGCTAATTTTCTTTTCA

>V300080312L3C005R0210638977

CGGTTTGCTCCTACTGTCCAATTATACCTGTGCTCGTATAATT

>V300080312L3C006R0060303863

GATTCAAGAATATTTGGTGCAAGCATAGTCGCTAGGTTAGGCG

>V300080312L3C001R0700847575

TTTGGTGCAAGCATAGTCGCTAGGTTAAGCGTATCCATCTTGT

>V300080312L3C002R0190224693

ACGTGGCATCTTTGGGATCCCAGAACGCAGTGGTATACTGATC

>V300080312L3C004R0330375503

TACCAAATTGAGCGCAGCTGTCAAGCTAATGCGGATCAGAACT

>V300080312L3C002R0360078524

TGGGCGAAGCCCGGTCTCGCGGGCGTGCGACAAGAGCGTAAAG

>V300080312L3C004R0600690022

TGAGCGACTAGATCATCGATCGCGGCAAACACAACCTCAACAT

>V300080312L3C001R0390356706

AACTGTCCTTCCAGCGGCTTTCGGCATTTAGTACAAAGGAGTG

>V300080312L3C003R0270350272

GTACATGAATTGTGGTCTGAAAAGTATTCGCCAAAGGACGTGG

>V300080312L3C006R0400276296

GATATAAAGTACGTGCTTGATACATTAGCTGCAACTTTTTTTC

>V300080312L3C005R0630842067

AACGCCATCAACGCACTCCAGTTTTTCAGTATACCCGCCCTAC

>V300080312L3C001R0041270362

TTTCGACATGGTATTGAAACTGAACTCTCGGATGTAGTACATC

>V300080312L3C003R0360206551

ACCCAGCTCCCCCAAAGAGCGTTCTCTTCTGGATCAGGTAAAG

>V300080312L3C002R0511153797

TATCTGAGAATGCTTACAAGCTGTTCAGTCCATACACTGTCCA

>V300080312L3C005R0121023264

GGCTGTGTGTACGAACGTGATCACTCATCTTACTCAAATGCTA

>V300080312L3C002R0180487076

CTGCGAGTCGATGGTAAGATTGGTTAGCAGATTTGGGAATTAG

>V300080312L3C005R0410396223

CTTTTAACAATGATCGGACGACATTGTCTTGACCTTCTTCCGT

>V300080312L3C006R0461164554

GCGCTGCAGTTCGAGGCTGTTGAGACGGGATGTTCGAAGGAGG

>V300080312L3C004R0571164562

GGCTGATATCAGATTTATTGTTGATGCTGAGTCGTCGGCGGTG

>V300080312L3C006R0010718096

ATCTTGAGACATCGATCCAGCGATGCTTTTGTCAAGGCGAATA

>V300080312L3C005R0630127757

CGATTCTTTTGTCAAGGCGAATATTTTGCTGACAAGTTTGTTG

>V300080312L3C002R0540566971

GAGCCGGTCCAGGTCTATCCTTTGGATCCTTGATAAGGCTAGA

>V300080312L3C002R0670194516

CGACAAAGTTGCGGCACTCCTTGGAAATATGGTCGCCCTGGAT

>V300080312L3C005R0661132345

ACGGTGCTCCTTGGATTCGTTCCGGCTGCAAAGACCCAGACAA

>V300080312L3C003R0451102508

ATAGAGTTGATGAGTTCACCAGATACACCAAAGTCGCATAATT

>V300080312L3C006R0170234268

CATGCACAGGGTGACCATCGTGTCACCATCCTCGAGAAAGGCG

>V300080312L3C003R0401172238

TGTTGCCACTCACAATATCGTCGACTTGCTCTCGAAAAGTGTG

>V300080312L3C004R0320143811

AGGCAAAAGCAGAGATCAATATTAGTGGCGGATGAGTTTCCCC

>V300080312L3C001R0400501081

TTTCAAAAATGGAAGGAGCTTAATGCACCTCCACTTTTGATAC

>V300080312L3C005R0531098187

CCGATTTCTAACCATTGTAATCCTTACAGCTTGGACACTCTTG

>V300080312L3C001R0031329008

ACTTGTGCGTGCAGGTCTCCGCAACCCCGTTCGCATTGTTGTC

>V300080312L3C001R0420900917

ACCGCAAGTTACACGACCGCAGTCTGAAAGCGTTTGTCTCTTG

>V300080312L3C006R0670461300

AGCGCAGATACCCCAAGACCAACGCAAGAAGAAAAAGTAATTG

>V300080312L3C006R0690326056

CCAAGCTACCGATTCTGCCATATTGCGTAATGACAATAAAGAT

>V300080312L3C001R0630926277

CGCGTCTCTTCGGCTTGCATGGGGCTGTGCTGGATACGAAGCA

>V300080312L3C002R0590727511

TACCTAGGATGCGGCAATGGTTGTACCAGAAGGATCGTGTGGA

>V300080312L3C006R0450330025

ACACGCGGGAGAGGAGGCGCTTTGAACGGCGAGGAGAGCAGCG

>V300080312L3C003R0621034665

AGCATGCGAAAGAAATGCCTATCTTTGGGGTTTCAGCGATCAG

>V300080312L3C002R0160284657

ACTCTCAAGTACCTTCTTCTTCTTTCGATATCGAAATTACTTA

>V300080312L3C004R0180223730

TCGATATCGAAATTACTTATTAAATATGGAAGGAGATCAAGCA

>V300080312L3C001R0080068099

AGCAAGTCTAGGGCGCATCCAGACGTCGACGTTGGAGGAGTTT

>V300080312L3C002R0481160234

AAAAAGAGAGTTGCAGCTGACGATCCTTATGGAATGGCAGACA

>V300080312L3C006R0620720940

TTGGAAAAGAATGCAGCAAAGTATATTTCATAGGCGAAACATC

>V300080312L3C003R0480935564

AGAATAAGGTCAAGGTGCGCGGGAGAAAACGTCACCTCTGCTT

>V300080312L3C006R0150857686

AGTAATTTGATTCGAAGCACCGCGTCGGTTCAGATAAGCTGAT

>V300080312L3C006R0530316067

TCGATCTGCACCTCGTTATCATGTTTTGCACTAACATATTCAT

>V300080312L3C005R0640516784

AATAGTCGAATACAATATTCTGGTTGGCTAATCTTTCAGATCC

>V300080312L3C004R0471236155

TTGAGCACCAAGTTAGCCAGGTAGGATACATTTATTCCAGGTC

>V300080312L3C004R0270907457

GGCACACCAGACAATCATAAGCGGAATCGACCTCTTATACTAT

>V300080312L3C006R0670438897

CAGAGTCAACTCTACTACATTCTATATCTACAACGATGTATTC

>V300080312L3C004R0390790486

GTTTGTTGGCAATTTTTAAAAGACGAGAGAGGGATTCCATTTG

>V300080312L3C001R0251233803

AGTTTCGCTCTCGATGCTGCCATCTCAACAAAGCTTCTATTCT

>V300080312L3C006R0690996262

AAGAAATATGATGACTATGACAAGAAGTACGATGACTATGACA

>V300080312L3C002R0250875333

ATGGCACAGCGATGCTCACAACGTCTATGGAAAGGATATTGAA

>V300080312L3C005R0371247803

CTAGAAGAAGCGATTAGCAGGGAGAGAACCATAGGAAGCAATG

>V300080312L3C004R0361196791

CTACTGATGTACATGGTCTGCCTCTTATTTCCACAACTTTTGA

>V300080312L3C004R0330224847

CAGAAAATTCGGCGAGATTAAGGATAATAAAGGTAGCAATCGA

>V300080312L3C001R0721270760

GTCTTTCAACGCTTTGGATCGTTTCCGCCGCTGGCTAGCCTGG

>V300080312L3C002R0100803758

ATCGTCATTGGGCAGAAAAGTATTCAAGGCGTGTTGTCGCAGG

>V300080312L3C004R0540289023

TCAGTCCGCTGCTTGCTCGCCTGGTTGACCGAAAACAAAAAAA

>V300080312L3C001R0050812254

GACGATCAATCAAGTTTTGACGGTGTTTGCAGGAGCAAGGCAG

>V300080312L3C006R0010791310

TGGAGAGCAACTTGGGGAGCGAGCCGCCGGAAACGGCGACAGT

>V300080312L3C005R0040149379

TGATATGCACCAAATGGATAATTAGTGGCAGCAAAGCTCAAAG

>V300080312L3C005R0180422666

CTTACTCCCATGTCTCTGAGCCCAGTTTCAAGTATGCAAGCAG

>V300080312L3C002R0070597901

CTATCATCTCGTTTGTCTTGTCACCTTTTGAGGGCTCGATCAC

>V300080312L3C004R0531382238

TATATGCAAGAAAGGAAAGGAAAAGACAGAGGCAGCAACAGAG

>V300080312L3C006R0340946652

ACTTAGTCTTAATATGTATGTGCAGAACCAAACGATGAAAGTG

>V300080312L3C003R0541004424

CGCGAAACGGCTTGAGTCTTGAGCAATTCTCGATGCAAAGCCT

>V300080312L3C006R0300080313

GGTCACCCCTGGCGCTGCTGCAAGAACTGAGTTTGTGGATCTC

>V300080312L3C006R0650306332

AGTCAAATCTTATCTCCATATAAGGCATGTACATTCGTGTGCC

>V300080312L3C004R0110114064

GGCATGTACATTCGTGTGCCTCGCACCACACACATACACGCAC

>V300080312L3C005R0550830269

TTATCGTCGTCCGGCAACGGTCGTCCGTTTCCGAGCACGTCCT

>V300080312L3C002R0600982593

CCTTGCTTGTTACTGCCCTTGCTATGAGCATATTATCGTGGCT

>V300080312L3C005R0601245139

CAACAGACATTCGTAGGAGGCTACACGGATTTTGCAAAGAAGG

>V300080312L3C002R0710168195

CTACACGGATTTTGCAAAGAAGGTGAAGGAAACATATAAAATG

>V300080312L3C005R0320552681

CCAAAAAATCATTTTTCTGCAAGGAGAATACGTTGATCGAACA

>V300080312L3C002R0520704211

CACCGAATTTCACAAGATTGAGCGAGAGTATCCGGACCTTTTG

>V300080312L3C001R0300115701

ACCTTTTGTGTTCTGAAAAATGTGTTCTAGTCCGGGTTCATCC

>V300080312L3C002R0450526183

TGTCCATTTCCTGACTGACACACGACATGACTTACAAGGTTTG

>V300080312L3C006R0330873233

GTGTGCGAGGCAAAGTAACACGGTTCATCGGCGAGTACTTTGA

>V300080312L3C005R0720122781

GATCAGGGTGACGTTTAATAGCATGTAGAATAAGAAAAAGATG

>V300080312L3C002R0170072067

GGATGCATGAGAACAAAAGGGCATCCATTGCGACGCTTCTAAT

>V300080312L3C001R0430526591

TGTTAAATCCAACAAGAGAGCTATGTATACAATCAAATTTGCT

>V300080312L3C001R0700786890

CCAGACGCCCTCAACTTTGAAACGTGCATTTCATACATTTCGG

>V300080312L3C001R0460503934

ACTTGCTGCAATCGATACTGTGCCTTTGCAATCGACATTTGGC

>V300080312L3C002R0340107678

CACGATCGCAAATGAGAGTGCCTGGATCCGTTGCGGCAACTTC

>V300080312L3C002R0070046676

CCTGGATACGAGAATTTTCACGTTCCAAATGGGCGAGACGAGG

>V300080312L3C002R0320401665

GACTCTGTTGTCCCTGCATCGCCAAGCCAGGTCCACTTGCTTC

>V300080312L3C003R0641373892

ACAATGTAGCTAACTTGCTGATAAATTATTTCATGTAGCTGTT

>V300080312L3C004R0230046350

GTTGAAGTGTCGGACGGATTCAACGAGATTCTCTAATCCTTGA

>V300080312L3C006R0680896072

GAAGTGTCGGACGGATTCAACGAGATTCTCTAATCCTTGATAG

>V300080312L3C005R0050234673

AAACCGGCAATCATAGCAACGCAGGCGTAAGCACCGCGGCACT

>V300080312L3C004R0290875654

GAAACGACAACAACCTCGTCCAAGTCGCCGTTGTTTACCTGAC

>V300080312L3C005R0391148896

GAGCAATTCGTGGTAATTCTCAAGTTATATTGGAAAGGTCTGG

>V300080312L3C001R0690289212

CGTTGCGGCTGGTTTTTGCATTTGCAGATCGCTGTTATTCTAG

>V300080312L3C006R0020810510

GCAGCTACCTTTGAAACACGAAAGCGGGCTTGCTTCGGTGGAG

>V300080312L3C003R0181403898

GGTGTTGATGCATCCTTGATCCAAACCTGGGTCTGCGAAGCCG

>V300080312L3C001R0331000318

CAATTTATAGTGGTCTTGAGAGAGAATAAGAATGTTCTCGACC

>V300080312L3C006R0330139074

GGAAGTCCCTGTTTCCTCGCTGCTGCCCAACAAGACCTACATG

>V300080312L3C001R0510995056

CGTCAATGCAGGATTATCAGGATTATCTCCACAATCCGCACTC

>V300080312L3C002R0350571528

TGTGGTTACAGGAGCTTACTGGGACGACAAGTATCCTCGCCTC

>V300080312L3C006R0330027888

AAGATGCTGCCGCGCACAGTCAATAATAAGAAAACGGTGTTGC

>V300080312L3C001R0451261301

ACAGCAAGTGGAAACGAAGCCATCTATTGGTCTCAGGGTCAGG

>V300080312L3C002R0391026546

GCTGGCAGACCAAACATGTAGTTTACACGGCCGAGCTAACTAG

>V300080312L3C005R0330515142

GGTTTTGACCGGCGACGAGAGAACAGCAGGCAGCAACCATCAG

>V300080312L3C006R0080380700

TCCCATCAAATCCAGCTTAGCATCCATTGACGTTTTCTCCACA

>V300080312L3C006R0260153252

TGCCGGCGAGCGTGACATGGTAGCGATGCACCATGAATTCACC

>V300080312L3C004R0030968396

GTCTTCAATGTCTTTTATCGAGCGCATATTGTCCCTTTTTATG

>V300080312L3C003R0160393182

GAATACCTTCCACCGGATGATGATGACGATATCGTTGATGATG

>V300080312L3C002R0180002840

ACCTTCCACCGGATGATGATGACGATATCGTTGATGATGATGG

>V300080312L3C005R0710743121

TTTTCTTTTTCTTCGTTTGCAATAAGCGACCACGTAATTTGTG

>V300080312L3C005R0681144857

CAAAGTTGATGTTGCTTAGAGAACAACATGTTGCATCCAATTT

>V300080312L3C005R0360051765

CTCCGAGCTACTTTGTTACATTGCCGAGTATCCTATTACCATC

>V300080312L3C005R0230325926

CATTCTTGAAGGCCGAAAAGCCGCTGAGAACGTAATCAAGGGT

>V300080312L3C002R0710710166

AGGTTACTGGAGACAACATTATAGGTGTTATGATTGAATCGCA

>V300080312L3C005R0471274198

ACGTATGTGTCACATTCTGGGGGAGGAAAGGATCATCATCGTT

>V300080312L3C006R0360211877

AAGCTGATTGACTTTTCACCAAACGAACGTTACCTTGTCACCT

>V300080312L3C003R0520978766

CGTGTTGTACCCGGTCAACAATTGTCTGTATACGAAGCACCTT

>V300080312L3C001R0491395443

CCAATCTCGAGATTTTCCGTGTTACAGAAAAGGCCATCCCCGT

>V300080312L3C001R0390428817

CTGGGATCTTGATTTCGAGCCGCTCAACGCAGAAGGCAAGAAG

>V300080312L3C005R0700815509

CGTCGGTGCAGCAGTTGTCTGTGCAGGAGCACTACGGTGTCAC

>V300080312L3C004R0020512921

CCGATATCGAGTGGGATCCTACCGGTCGCTACGTAGCAAGTGG

>V300080312L3C004R0081387636

CCTTGATACCGGCCGTCATCCAGGCATCGAGATCGAAGTTGGA

>V300080312L3C002R0361194557

GTTTGTGCTGTACGCTGTATCTATCATCAATACCTATCTTTCG

>V300080312L3C001R0230656850

TTATCTTTTTCTATTGTAGCGTATAGCAGAGTGAACATTCGTA

>V300080312L3C006R0270698419

TAAGTAATATCTTGATGATTTCACTGACATAGACCCACCAGGG

>V300080312L3C003R0660042716

CACTCATCGTGAGAAAGGTGGCGAAGGGGTAAGGGCCAAGGGG

>V300080312L3C006R0501014306

TTATTATTATTGAGTTTGCTCCTTTTTCCGCTTAAAGTTTCCC

>V300080312L3C003R0610297808

TATGCAAAGTCCTGAAATATACGACTTTCCAATCAGTAGCTAG

>V300080312L3C002R0400294465

ATTGAAACTCACAAAAGAATGTGCCTTCGTTAGCTGGGTACAA

>V300080312L3C001R0620350522

AACAACAAAGTGGTAACGGGCAACATATTTTAGTAAGACTGAT

>V300080312L3C006R0661233348

CAGCATGTGTAAAGCGGCTGAATGTCTTGTAAAAGGCGATAGA

>V300080312L3C001R0570637535

GCATGTGTAAAGCGGCTGAATGTCTTGTAAAAGGCGATAGATG

>V300080312L3C002R0720009005

CGTACCTCTTGATGGACATTATACAGCTTTATCATAAAGCGAT

>V300080312L3C003R0681319354

CCCTGGACGTTGATGGTAAAACATTGTTGCAAAGTTGCACAGA

>V300080312L3C006R0600979516

AGAGCACAGCGCAAATTATTAGGAAAAATCCTGTAATTTATTG

>V300080312L3C004R0320907972

TAAGTCCGGGCTGCTGTGCTTGCTTTATCAGGTCGTCTAACGA

>V300080312L3C002R0570571347

TATTAGTTGTTGAGCTCCTTTGCCGGTACAAAGAACAATATGT

>V300080312L3C005R0691358886

CACAAGAAAATAGAAAATGTAACGTGATTGCAGGAAAATCCGG

>V300080312L3C002R0410876498

CAACAATGCGGCCACGGATTTCCTTGGGAGCGATTTCGGATTG

>V300080312L3C002R0631228268

GTGGCAGAAGGAGCAAAAGTTTTTATAGAAGGGCAGGGAACCT

>V300080312L3C004R0211271643

CTCTTCTGAATTTGACATTTTGAGTTCTTTCTTTCAACAGCAG

>V300080312L3C002R0260140171

TGAATAAGGAAGGTGACAGAATAATGACAACACACGAGTACTG

>V300080312L3C001R0610395220

CATTTCTTGAGACGGATAGAGTGGATCTTGATAAAACCATTGG

>V300080312L3C002R0280652699

GCGTTGGCTTGGATGGCAGGGAAAATCAATTCCTTGACAAACT

>V300080312L3C002R0090977573

CAGCAATGTAGGCCATGACTTCGTAGCCCTGCTCAATGAGCCA

>V300080312L3C002R0240319832

AGTATTTTTAAGCCTGTTTCTTTTCGAAACATCGAAACAGATG

>V300080312L3C006R0521047959

AACAGATGTTCATTTCCTAGCACCATTTAGTTTTGATCTCCTC

>V300080312L3C003R0671057763

AAGTCGACACGAATCGTTTCAAAAAAAGGAAACATCTCCTGGA

>V300080312L3C004R0400880136

TACATGCGTCAGTTTTCTACAACAGTCACCGAGGCCTAAATCC

>V300080312L3C003R0461045917

TCTATTGGGTTGAGTTCAGAAGAGTATGATGGAAGATAAGCAC

>V300080312L3C004R0610887347

TGCTCGAATATTTTTTCACTTCATCTCTGCGAATAAAAGCGTG

>V300080312L3C003R0330199343

GACAAACACCTGCTCCACTGATGATATATCCATCATCATAATC

>V300080312L3C004R0080305632

TGTCATTGGTAAAGCATTGCGATGCAGAGCGATATATGCAGTC

>V300080312L3C006R0021108152

GTCTGCTGATCAGGTTTGCTCATAGAATACAATCGCGGACTCG

>V300080312L3C006R0220454735

ATTGGAGCAACCCGACACTCTACAACTGCAGTTCTATTGATCT

>V300080312L3C003R0641254453

CTAGATTAGCCTTGTTAGCATCGTGTTCCGCGCTGCAAGAGCA

>V300080312L3C004R0460134677

ATTCATTTCTGCTTCCGAAGATGAACGACGCGTGGGTTGCGTA

>V300080312L3C005R0010237795

TATCCGCAATATCAGCACCAATGAAGAAAAAAAATCTTTGTTC

>V300080312L3C004R0320571785

ATGAGGCCCTGGACAACAGCGGTTTGGCTGTTCCACCAGTCGA

>V300080312L3C005R0590560271

TGCTACCTGCATGGTCAGCAGCAAGCAAAGGAGTAGCTATTAA

>V300080312L3C001R0501363453

GCCATCTTCAGTCTTCATTTCGTCGACGATGGAAGCAGTAACC

>V300080312L3C001R0490339598

ATTAACTGTGCATCTACGGAATGCATAGTACAAAAATTTAACT

>V300080312L3C003R0280099490

TCCATAAGTTTCCTCGTGTAAGAAGTACAATAGCTATAAAACG

>V300080312L3C003R0381010743

TAAGGAGGAGGCAGGATCCAGCTGTACTAGGCCGCCCGAACAG

>V300080312L3C006R0650820836

ATTGAAGTTCAGGTAAATGTCATCTGGAGTCTCAATAAACTTG

>V300080312L3C005R0280745465

CGTTCATTGTACTGTCATGGATGTTTTTGGAACTGCAGTGGAA

>V300080312L3C002R0080374371

GTTAACTTTTGAGACGTCAATGCAGTCCTTTGCAAAGACAACA

>V300080312L3C004R0451157151

CAACACGCAGGCACAGCACACTCCCAGTGTGGCTAGTGCGGGT

>V300080312L3C006R0431359561

AACTGCAAGCATGATGGTGGTGCTCGCTTTTGAAAAGAAAGGA

>V300080312L3C001R0021101148

CTATGGACGAGCGCGGGCATAATATCGTCAGTAAAGTGGAAAG

>V300080312L3C001R0690827287

AAAGTGGAAAGAAGGCAGCACGAGGAATGATGTAAATTTTCCG

>V300080312L3C002R0620919281

CCTTCCTTCAGTCCTTTCTTTTCTGTTGTCAACAACAACCGTA

>V300080312L3C004R0670126508

GGACGGTTAGACTGTCTTTATTACAAGTAATCAGGTCATAACA

>V300080312L3C002R0440172829

GCAAAAGTTGTTGGGCATTTACACAGATTGTAGTAACGGTTGC

>V300080312L3C005R0670038645

GTACGTGGTATTACAAATTGCCGATCTCGCGTCGCAGTGTATT

>V300080312L3C001R0521255398

AGTATATTCCACCTGCGTCGTGCCTTTATTACAAAAATATCTA

>V300080312L3C002R0200290241

TAGATTGCTCAACCGAGGCTCCATTGCACGGACCACGGCGTCA

>V300080312L3C003R0040535304

AAGAGCAACACGCGAATGCAATATCTGAATTGTCGAGTAAGAA

>V300080312L3C005R0701402375

GATCGCATCGATACCATCACCGTGCAGCTGGACTTTTACAATG

>V300080312L3C003R0610349789

TAAATTCCACGTCGACGTGTTATATATAAATTCCGTACGTATG

>V300080312L3C004R0710229405

ATTATTGCTGGTGCTGCGCTGATGATGTTCATTGTTGTAATAG

>V300080312L3C004R0440367309

GCGGAGGTGCGGTCATGTAAGGAGACACAGGTTCTCCGCTCGA

>V300080312L3C004R0431086479

CATACTTGTAATATCCGTTCATTTCACTGTGTTGTTGCTCGCT

>V300080312L3C006R0040740098

ACGTGACAAAAAACATACACAGTAGTCCTGTCAACGGCATAGA

>V300080312L3C003R0210306985

GGGGGCTGCACGGCTGCCGGGTGATGCTGCGATGGGTGAATGG

>V300080312L3C003R0670399628

GTAAAATGGTAGTTCCCTTTTGTAGGGATCTGATCTCCCAGTA

>V300080312L3C001R0661163146

AAATGATTCCGAAGTAGAACAAGATTGAGGGAAATAAAAAACT

>V300080312L3C002R0540497112

CCTCCACGGACCCTATTGGAGTCATTTCTGAGTTGCTGCTCGG

>V300080312L3C004R0580929437

CCATGGCCGGGACGGCCTCGGCGGGCGCATCGATCGTGCCATG

>V300080312L3C006R0470142910

AAATAAAAAATGTGCAAATTGAAAAACACCAAACTTTGTTGTT

>V300080312L3C002R0060670304

GCGCCGTACGACATCCTCCTCGCCCAGCAAAGAGCCGTTGCCG

>V300080312L3C002R0061075878

GTGGCGAGCTAATGCGTGACTGAGATGGGTATATCGTTGAGGG

>V300080312L3C005R0631344696

TAAGATCAAGCCCTTGTCGCCTACGAAGCGTTGCTGCTCTTCT

>V300080312L3C005R0200420481

TAAAGTCTTCGGCCATACGTTGCCAGGTCTTACATACTCCTCG

>V300080312L3C004R0040075246

CCCTGCTTGCAGGTGCAGCATACTCAGCACTTCTTCAGGTACA

>V300080312L3C001R0251025585

CGTATCTCGCTAGATATCACTAAACACAGACTCTATTAAAAAA

>V300080312L3C005R0590217155

TTGGAAGCTGTCAAACAGCTTCCTAGAAACTAGCAACGAAAAC

>V300080312L3C005R0681223956

CAGATACTTGCATTTAACCTTCAATTCAATTGCTTATCCATGT

>V300080312L3C004R0140591382

CGGTTACATGTTGTTGTTGGCCCCGACAGATTTCTAGTGCATG

>V300080312L3C006R0201319494

CCCGTCAGATTTCTAGTGCATGAGGTTTTTCCTTTTTTCCCTC

>V300080312L3C003R0220453515

GTAAAGTCTTGCTGATTCGATGAAATGCCATAGATACTACAAA

>V300080312L3C001R0370141375

TACACCGACAAGTCAGTGAAGTCACTATACATATACGTGTTCA

>V300080312L3C002R0191287843

TGAAGAAAGCATGGAAGCGCTGCAGAACCTCGTAACAACAACG

>V300080312L3C004R0570106708

GAAGCGTTACGACAGACATACATTGCCTGGTGTGATATATACG

>V300080312L3C006R0640883643

TGATGTCCGCATGTCGGTACTTCATCCTATGACTGTTACAGCG

>V300080312L3C006R0460874437

ATTTATGAAATGCATTGAATCTCCAAGACTACATGATCCGTTC

>V300080312L3C004R0630352832

GAATCTCCAAGACTACATGATCCGTTCTCGTTGCAGAGAAAAC

>V300080312L3C004R0151103536

GAAACGTGTCTTGAATTCCAGAGCGACTTTGCATGCCTGGCTA

>V300080312L3C004R0310682503

CTTCCGGAAGACGGTTCAAAACACCTTGAATGGTACGCCAATC

>V300080312L3C005R0381113054

GCGCATAGGACCCATTTCCTGATAAGCGATCGACGAGTTACTG

>V300080312L3C001R0060883340

GAACAACTTTGCAGAACAGCGCTACTCCTTTGTTATCTCGACC

>V300080312L3C002R0610380699

TGAGCGCGGTACCTCGCAAGGACGAAGAGTTTAGCACGATGCG

>V300080312L3C002R0240837329

CATGGTCCATTGGAGAAATATTTCAAGGGCGAAGCACTGCACG

>V300080312L3C004R0370962575

CAGGATATCAAATGGACATCTGACAGCAGCGTCAACCTTGTAC

>V300080312L3C003R0091276716

CGACTTAGCAAGGCGCGTAGGACTGCCTGATGGATACTACTCT

>V300080312L3C004R0361112475

GCGATACTGATCGGATATCGTCTTTCAGGACTACTTTCAAGCG

>V300080312L3C006R0120208856

ATCAATGTAGCAGAAGAAGGTCATGATCACATAAAAGGACGGT

>V300080312L3C001R0060981173

CAGAGCCAGAGAATCGTTTGGCGGATGATTTATATCAGTATAC

>V300080312L3C003R0350181589

ATATTAGAAATATTTCTGCCGTGCAGTACGGTCCGCAACCGAG

>V300080312L3C004R0370046080

TAATTTAAACTGGAGGAAGATGCTTCATAGCTGAACAAAGATG

>V300080312L3C003R0181294399

TAACATTGTTGAGCACCAAAATCTGTCCTCGGTAGGCAAAATG

>V300080312L3C001R0450131274

TTCACCCAGGTACGAAAGAGAATGAGTTGACAGGGACCTGGTG

>V300080312L3C002R0461179848

TTCCTCGTGAGATAGTTTGCTCCTGGAAGACATGGATGAAGCG

>V300080312L3C006R0280343756

AACATATGCAGGCTGTCCGCCATCTGCCGCATATTGCCGAAAT

>V300080312L3C006R0650058736

CGATGGTTACTGCTTTGATACTCTTGCGCTTGCTTTGTGTTCC

>V300080312L3C004R0090553064

GACGAGTTTCTTTGAACAACCGTACAAAGAATGGATGCGTGGA

>V300080312L3C002R0351190587

AACACAACCTCTGGATGGATTCCCGGTGCGCGCTATTGGAAAT

>V300080312L3C003R0430706292

AGAAAGGCGAGGTAACAACATAAAGCAATCATTCTTGTTATCA

>V300080312L3C001R0281259080

CCGTAACTAGACCGCAGATAAAACAGGGCTTCGAGAAGAAGAG

>V300080312L3C002R0350775923

GATAACCTGTCCACGACGGTGCTGCTCAGCAAAATTACCGGAG

>V300080312L3C003R0311384229

TACACAAGATTGTCGCGCAAAAGTGGTAGCAACCGTCCCGGCC

>V300080312L3C004R0360427448

TGGCGCACCTCCCTTGCCTGCATCCTACTACTCCTGCGTATCT

>V300080312L3C004R0391339823

TTTCTGCAAGGCCAGAGCAACCGATTGACGGATGTGGTGAGAT

>V300080312L3C006R0011336804

CCGACACGGCTGTGATTCTGCTTTCCGCCTTTGTCAAGGTATT

>V300080312L3C006R0501215697

TTCAACGGCGTGCAGCCTATTTCAGACATTCAAACTGCTAGTT

>V300080312L3C004R0490071234

TATCAAATTCATCAGTCTTATTATCTTCAAGAACCAGCTCAAG

>V300080312L3C006R0630377652

GTTTATTAACAACGCAGCCGTTTTCAACTTTGGCTATAAATTC

>V300080312L3C002R0491404740

TAACAACGCAGCCGTTTTCAACTTTGGCTATAAATTCCGCCAG

>V300080312L3C005R0601215460

TAATAGCTTACATAAAAAACAGATGATTATTGCTCCCTCCCTC

>V300080312L3C002R0410406473

CGAGAGATTCAAGATATACCAGGATAGCAGTGATGACCGGTAA

>V300080312L3C002R0230871345

AGATTGGCCAGCTGCTCTTAATGTACTAAGATAATAATAATAC

>V300080312L3C006R0030452692

CTTGTTTATCCGATGTCGGCTGCTGTACACAACCTTGGCGTAG

>V300080312L3C002R0181370937

AGAAGAGCTCTGTCAGAAGCCAAATCAACGGTCAGTGCTTGCA

>V300080312L3C005R0571017176

GTGCTTGCAAACGACTCGTTCTAACATTGAGGGAATACTTGCA

>V300080312L3C001R0620329844

ATAGAACAGACTGTTGGATACCGCAGCACTGCCATTCTCGTCC

>V300080312L3C006R0310156893

GATCAACTCTAACATGTCTGGACGCGGATGCTACAAATGCGGT

>V300080312L3C002R0140637850

GAGAATAAAAGACGCACGCGGGCGAGCGGGTTTGGGCAGAAGA

>V300080312L3C003R0391227767

CTCTTTTCTCTTTTGTGTATGTTTGAAAAATCAACAACAGCTG

>V300080312L3C006R0430647308

TACAGTCTATTGTGTACACACATTTTAGCTACAACTGCCGCGG

>V300080312L3C002R0560965533

ATATTTCAAAGACAAGATCGAGCAGAATCTCGGATGACTGGTA

>V300080312L3C001R0150363382

AGATTGCTGTTAACGTAATCTGGTGTAGTCAGCCAGAAGAGGG

>V300080312L3C006R0270504442

GGCAAATCGGTGCTCTCCGTGTTTATGCAGCGGCGGTGGTGGT

>V300080312L3C002R0521054383

CTTGCATTATTCGTCTGCCTGCATGTCGACCGAACAAGAAGGA

>V300080312L3C004R0720438159

GGGTACGCGGAAAAGTGGCAATCACTGGATGTCACGCGACTCA

>V300080312L3C005R0570866410

AAAAAGCAAAGTTGGTTGTCAAAAAGTGGAAGGATGATGTGCA

>V300080312L3C003R0471037401

AAGCAAGTGCAAGTTTTGTCTCTGGTACAGTAGTGGGCAAGAC

>V300080312L3C003R0360400077

GATGTGTACACGCTCACTCAACCCGTTCGCGGTGCACCGACAA

>V300080312L3C005R0321088436

AAAAAAATCTTAACAAGGGCAACTGCCAACATTCAGCGCAGAG

>V300080312L3C004R0220938926

GATTTCTCGTACTGGTGCAATGTCTGGGCAACTTTGCTATCTC

>V300080312L3C003R0160000636

AACGGACATTCACATAGACTTGATATTTTCTTTTCCAATAACT

>V300080312L3C004R0130994363

CGCAACGACACAGAAAAGGTGTACGAGTTGTTAAAGAAAGAGG

>V300080312L3C006R0090693308

AGTAACTTGGCACGACTACGGCATCCGTCTATCCTCGAAGTCG

>V300080312L3C002R0050336592

CATGAACGATGCTCGTGTAGTGCATCATAATTTGACACCAGAG

>V300080312L3C006R0261174333

GGAAAATAGGAGGGTAAGAGTAACAGAAATACCACTGTTCTAA

>V300080312L3C002R0630806145

CCTTAGAGGATGGGACACTAACATCGTATCGTAGCTCCCGAAT

>V300080312L3C002R0261241349

AATCCATCAAAACCGGCCCAGTCGTCGCAGCAATCACAGAGCC

>V300080312L3C003R0500878558

TGTTCCTCGGACCCCAACATCATGGCACCCATGCGATGCCTGA

>V300080312L3C001R0520954361

AGCCTCCAAGTATGGCCTGAACGTCAGCAAGGCTGCGGTCAGC

>V300080312L3C006R0431311867

TCACGCCAAGGCAGTGCAGAGCAAGAGACATCTTTACTTCTCC

>V300080312L3C006R0710752815

TTTTCAATTTCTCGGATCGATAGTTGCTGCATCTCTTCTTTGG

>V300080312L3C001R0420272666

CTTGTAGTATATATACTGTTGATGTATCAATTTTACAAAAGGA

>V300080312L3C004R0610000413

ATATTTGAAGTATTTCCTCGTCCTTGAATAACGAGAATTTGTA

>V300080312L3C003R0210031199

GGAATATCTTTGGATGCGAAAATGCCGAAACAATATTCACCAC

>V300080312L3C005R0540683131

GAATTTGTTGTGGCTGCTCCTCCTTCCTTTCTTGTTGCGTCGG

>V300080312L3C006R0710422590

CATTGTTTGTCACGTCGTTGAGCGCGTTCGGATCGTCAGAGGT

>V300080312L3C001R0210133206

AACGAAGAGTTGCAGTCTCACATCAGGCAGCACCACAATCCTG

>V300080312L3C001R0220241202

CATGAACGTACGCATCCAGCGGACCATGTAAGTTTCAAAGACT

>V300080312L3C004R0100990821

GGTTTCGAATCAGCGCCAATCTGTATCGCCACCAAAGGCGCTT

>V300080312L3C003R0590766330

CAGCTTCGTCCAACAAGCTGTCCTCGACGTCCTCAACGACGGG

>V300080312L3C006R0650490002

GCGGTATTCGACAGGCATGACCATGACGAACTTGGGCAAGTAC

>V300080312L3C001R0440310998

GTGGTCGCCAACACCTTCACAAACAGCGTGGGCGCCAGAGTTA

>V300080312L3C004R0141082698

GGCGCTGCTTGGTGACACAGTAGTTGGCGACACCAGGGCGAAG

>V300080312L3C004R0540842014

TCTAATAGATAGGAATACAAATATAAATAAAAGATGAAAGGCA

>V300080312L3C002R0410474100

ATTCAAGAGACATGACGATCTCTTCACGGATAGGGTCAATGGG

>V300080312L3C001R0710714963

ACTTGGCAGCAGGACCAAGAATGGAAGAGTCGCGAGGAACAGA

>V300080312L3C004R0470584395

GATGCGTGGTCGTAAAGTCGCTGGTGAATTGCTCAGGATGGAT

>V300080312L3C002R0030185125

AAGCGAGTATGGAAGGGTCGATGGTTGAACGGAGGTGTTTACC

>V300080312L3C003R0470167012

TACTCTGAGATAACTGTGATCGATCATTCGAAAAGACAGTAGT

>V300080312L3C005R0141211322

AGGGTTTACTAAAACGGTGTGACTGTTTGTCTATTTACCAATG

>V300080312L3C006R0141294855

TGATCAATAAATTCTTATTTACCTTGCACCCTTTCTTTCCTTG

>V300080312L3C005R0191401946

AGCTTTGCAAAAAGTACAAGGTGCGCACATTTGTTACTGCGTA

>V300080312L3C006R0300944845

AATCCTCGATAAGTCTGTTTCGACTTCTGAGAATCCTGGGCTC

>V300080312L3C006R0560912190

TGTACCCACAGCTCGTAGCTGCTGCTATATCCGCAGATAAATA

>V300080312L3C006R0430476810

ATTACCATCATCTTTCATTTTACCGATTCTTACGCGTCAAGTA

>V300080312L3C001R0660027268

TTCATTTTACCGATTCTTACGCGTCAAGTACGGTAGACGATGA

>V300080312L3C002R0390697938

AGGACTTACTCAATGTCGACTTCTTCTCAGAGCTTTGGCTGTC

>V300080312L3C005R0691144802

ATTAACTCGTCGTTTCGAGAAGCGTGCACACAACAAAAATACG

>V300080312L3C005R0421363810

CGATGATTTGAGAAAGCAGAACAAAAACTAAAAAGAAATTAGA

>V300080312L3C006R0350359521

CTTGCAATAGCTTCAGGAAAGCCTCTGGATTTTCCAGACGACA

>V300080312L3C002R0231284645

GTTTTGAGCTTGGAAAGCTGCCATTTTGCACCTGGGACGAGTA

>V300080312L3C005R0330425681

TGCTGGTCCATATTTTACAGAGCCATAACCAAGCGAGAAACGG

>V300080312L3C003R0401291152

GACTTGACGACGTCCTTGCGAAGCAGTAATAGGTATCTCATAT

>V300080312L3C004R0531352680

AAGCCTTTGGATATCGAAATTATGAAGCACCTTGGCTCTAGAG

>V300080312L3C005R0250645138

ACAGAGCAAGTCAAGAAAGAGGAAAGTCGATTCCGTCAGTGGG

>V300080312L3C006R0700166761

GTTAATGTGTTTCGATAGTTGGTCAGCGAACGCGACCGTCTCA

>V300080312L3C002R0551220783

CAACAAGGATTTGGAGGAGCAACATGCGAAAATCAAGGCTATG

>V300080312L3C001R0611268461

GCGGTAGTCGCACTGGAGAACCATTTGAATCCGGTGACGTTCC

>V300080312L3C003R0070508754

TCTCGGTCATCCACTGTCCACTAGTCCAGTTTTGATTTGGATC

>V300080312L3C005R0580697275

TGGATTGCATCGAGCTGCTATCGTTGCTGTTGCTGCTATTGTT

>V300080312L3C005R0680483068

GTGGAATTAGACATTCTTTAGTACTTTTGAATTCAATTCAGGG

>V300080312L3C006R0081178766

GAATCAGACATTCTTTAGTACTTTTGAATTCAATTCAGGGACA

>V300080312L3C005R0560270797

GTCAAGATTACGGCAAATCTCATCGCCAACGTCATGTTTTGTC

>V300080312L3C004R0441184501

CCTTGGAAAAGGGTCCAGATCCTTTTCGTGCAACTTCATCGTT

>V300080312L3C001R0211171460

GCCAGATACTCGATCTACTACTAACTCACCTCCAGACGGGCAC

>V300080312L3C006R0450910468

ACACGTATCCAAGCCCTTTTACGAAATGCTGCAGCGGTGGATA

>V300080312L3C002R0230509768

ATCCACCTCCATACATTCTCGTAAGTTCCATCGGACCAAATTC

>V300080312L3C002R0630693962

CCTTATGGATACGCTAGGGTGAGCAGTTTGCACCTATCTAACC

>V300080312L3C006R0561321251

TGCTATACGCGCTTCTAATGCACAGTATGACGATCCTGAAATC

>V300080312L3C003R0461335772

AAGCGTCCATATTTTGCGACTTGATTCGTAACAGCGTATCACG

>V300080312L3C005R0660343924

GGCCGCTAAGCAACACGCATTTAGTCACCAAGGAAAAAAACGG

>V300080312L3C001R0270839317

CGTCAGAGGAATCTGAAAAAGCCCCGTAAATAACAGGATTCGT

>V300080312L3C003R0280011889

AAAGTCGGGGCGGCCACCAAAATTTCAAATAGGACATTCCCTT

>V300080312L3C001R0190591182

TTAACACAACTCTGCTAAGCTCCCACGTGGCAGAAGCTCGCAG

>V300080312L3C003R0620732727

GACAACCATTGTTTATCAAGTACTATGCACCGTGGTGTCCTCA

>V300080312L3C006R0350114332

CCGTGATGTTTGTATTGAAAATGGAGTTACATCCTTTCCAACT

>V300080312L3C006R0190842113

TTCGTTCGCCAATACAGAGCAGACGCAGAGTTCATTGACAGCA

>V300080312L3C005R0650511643

ACACCTTCCACATGCGTTGGATGGAAGAGTGTATAACACGACC

>V300080312L3C004R0631361278

ACAGCAAATCAAGAATATACTCCATCGAGCGCCGGTCTGAATA

>V300080312L3C006R0021203418

TGTCTTTATATTAAAAGAATATAATCAAAGTTAAATTACAAAG

>V300080312L3C005R0561212493

GAAGACTGTTTGCGGATTTGTTTCGAAGCAGCAAGAAGCTGGA

>V300080312L3C005R0460184297

CGGGGATACCGCGGTCCAAAGAGTCAATGCCGGTCCACTTGCC

>V300080312L3C002R0490449131

AACTTCAAGATATCACGGGTGATCTCAATCAAGAATGAGTCCA

>V300080312L3C001R0160871574

CTTCTCCAATTCCTCGCAGCGGCGGATGGTATCGGGGAAGTGA

>V300080312L3C001R0280252752

CTCCAAAAAGTGGTCCACCTTGGCAGTAGTGCGGTTGTAGGCG

>V300080312L3C005R0241092383

CACTAAATTACTTTCTGTTAGTCCAAGACGTCCGGCACATATT

>V300080312L3C003R0080188628

AAAAGCAAAAGTGTAAGGAAAAGAAGAAGAAATGACCTGCGAG

>V300080312L3C002R0391165464

CATTGGCTGATCAATATCATCTTTTCCCGCTTTTGCATCTTTG

>V300080312L3C003R0450621626

CGGCACAACGGCATCCCGCAGAATACTCTCGCTCTTGAGCGTG

>V300080312L3C003R0441262129

GCCGTTGCGTCCGCTTGTCAATTCTAAAACGCGTGTTTAGGGC

>V300080312L3C004R0310575207

CAATTCTAAAACGCGTGTTTAGGGCTACTGTTCGCAAGCGTAC

>V300080312L3C004R0211127137

CGTACTTTAGCGTACCTGGCGACTGACACCTAGGTTAACGAGT

>V300080312L3C001R0430266041

CAGCTTCTGTCCATTAATCTCTACATTGATATAAAGCCGAGTG

>V300080312L3C004R0280874967

ACGATTTTGTCGCTTTCGTGGCGCCCGCTTAAACAGCTCAAGC

>V300080312L3C002R0400228843

GCGATCGTAACCACAGATCGTTGCAAGGTGTAGAAGGGTATGC

>V300080312L3C005R0581215895

CTTCTAAACGCTGGATGAAAAGTGTTTTCGCATTGTCGTATAC

>V300080312L3C003R0640476748

TGCAAACAATGCTGCCAGTTTGGCAGCATTACCTTCTTTGTTA

>V300080312L3C003R0460578720

GAATTGCTGACCGTCCATTCCCATTTCCGGAAGATGGTGAGAA

>V300080312L3C002R0101027953

ATACTAGCGAACACAATACGGTAAAATTGAGTCCTGCGTTTTG

>V300080312L3C002R0690028781

ACAATGGAGGAATACAACTCTAGTGACATCTGGACGCTCATAT

>V300080312L3C004R0050909138

ACTCTATTATTGCAAACCGTTCCGAGAATGCATAACGAATTTT

>V300080312L3C002R0360916765

GGATATGTATTATTCTACCAGGCGGTGAATCTGACTATGGATT

>V300080312L3C004R0320279127

TACCACCGCTAGCTATCCGAATCGCATAGACTCACCTTTACGC

>V300080312L3C003R0660116961

CGTATATGTATCGCATCATCTTCCTAATATACTGTATAAATAA

>V300080312L3C002R0160975523

CTTCTAAGGGCTGTCCGGTTGCCGCTTCTGGCCTCTGTCTCTG

>V300080312L3C003R0101004870

ATCGCAAAGGTACAAAATGAATCACCTTGAAGATACTGAATTA

>V300080312L3C002R0611016911

CCGTCGAGTTGAAAATCATTGATTTGCAGCGATACGCTCGTCA

>V300080312L3C002R0091182636

GGCGCATCCTTGTCGCAGCCTACCTGAAAAATCAATGCCGTTG

>V300080312L3C005R0070149206

GGAGCGTCGTCGAGCGCCTTCAAAGGCATTATGCAGGGATAGT

>V300080312L3C003R0091297575

GCAACAAGTTGATAGGTCTGACATCCTGCAAGAGCAGTAGCAC

>V300080312L3C002R0041023771

GAAATTATCGGGTCCATCACTCTTTAACGATTCCCAGCTGACG

>V300080312L3C003R0401322852

CGCTTCTGTCCAATTTTGGACGTCTCGGCTTCTGGTGTCTTTG

>V300080312L3C006R0060731391

TGGTGCCTGATCTTTTGAGTGGTTGATATGTAGTTTAATGTAT

>V300080312L3C001R0380500458

TGCTGTTTGAATATGTAGGTGTATCCAAAAGGATCTCTCGTGT

>V300080312L3C001R0340699264

GAATTGTGCGCCAATGATTTGAAACAGCAGAACTGGCTACTAT

>V300080312L3C001R0481215421

TCAAAGGTCGCATTGTGTGGAAAGAATATAATATGTACTAAAA

>V300080312L3C001R0380297851

CATTAGCAGACTTGCGTGCAAGTGCTTAGCATACCGTCGCTCT

>V300080312L3C003R0200312090

AAAAATTCTCTACAGAAACCGGAGAAATTGACTAATTTCTACT

>V300080312L3C006R0650307506

ACCGTGACCCGTTAAACTTTAGTTTATCCGACCTTTCGAATAC

>V300080312L3C004R0671260352

GAACTACCAATATATATGTAAGCTGAGCCATATATAGAAATTG

>V300080312L3C002R0070544352

TTCCATCCAATCGACTGTACAAAATTGCCGCTGAATTGTTTGC

>V300080312L3C001R0040740525

GATCAACTGCCAAGTACTTTGCCAAGTGGATCGCAAGTTTATC

>V300080312L3C001R0620451945

TGAGCGATATGTATTGTATTTGAAGCGTTGGGGCGTCCATTTC

>V300080312L3C001R0600295981

AGTGAATAGAAAAAGTTGAGCTATAAGTACACGCGTTGAAGCA

>V300080312L3C005R0660057179

AGTGTTTGTGACAGGGAAGGACATGGTAGGGCTGTGGAAACCA

>V300080312L3C002R0530094067

GGAGCTCGCGCTCAGGGAGACCTTCGCTTAAAGCGAGAGGTTT

>V300080312L3C004R0411380064

AAACAACGGGACCCCATAAGCTGTATGCCAGCAGCAGCGGCAG

>V300080312L3C004R0191089516

TAACTATGCAGTGATTCAAACGACTCCACGGCCAAGCTTGGCG

>V300080312L3C004R0251234202

GTTTGACAGGACCCGAAAACGCCTAGTTGATTTACCTTTTCCC

>V300080312L3C005R0691271347

AAGGGCCGGGTAATCATCTTCTAAAGATAGTGGTATCTGCGCC

>V300080312L3C004R0281338907

ATAGTCTTTTGCAATGTACGTTCGCCCGGAACATGCTCTTTTT

>V300080312L3C004R0310721070

ACATGACAATTTGACTTACGCCGACGTCCGTAGTAAAAGCAAC

>V300080312L3C001R0470131733

GACAATTTCAATCGCTTGCTTTGCCGATCTTTCTGCATAATAT

>V300080312L3C003R0561374951

TCAGCGCGCTCAGCTATCAGCTTCCTTTTCAGTCATTAGAATT

>V300080312L3C004R0480630662

GTGAGGACAATGGAACAACTCTTCCAGGGTCTTCCTTCGTTTC

>V300080312L3C002R0630235667

TACGCCGAGCTCAGGGTGTCAGCAAAGTACAGAACTCGCAACA

>V300080312L3C002R0090584964

GGTACTCCGGAAGGATCACGTAAGTTCGCTGTATCACGTTGAC

>V300080312L3C002R0690448878

AAAGAGTTCTCTTCGCTTGTAACAACAAAACACAACAAGGTGA

>V300080312L3C006R0360411424

ACGAGCAGTTTCTCGACGAAGCGAAAGCTAGCTCGAGTTTGGA

>V300080312L3C001R0270060773

GTGAACGCAGCGGATTGCTCTGGTATGGAGCTCACCGAGACTA

>V300080312L3C005R0531161316

TTAAGGTTGCCGGTGTCCAGGCAACAAGGTCGAAAACGCAAAG

>V300080312L3C004R0280067288

AGTGGTGCCAAGGTGCTTATCCGCTACTGCTTATACGCTTCGA

>V300080312L3C004R0451060771

CCTTCAAGCCCGACAACCTGACGAACGCTCAAAGTGCATTACC

>V300080312L3C006R0521169676

ACTCACATGGAATCCAATCTCCCTTCCAGGCTCGAGGTTACCA

>V300080312L3C006R0500268383

CTTGCATCGGATGGACGCGTGATGTGGTAAACTTGCATAAATA

>V300080312L3C004R0330010561

GCAAGCGGTGCAGCCGCTGCACAGAAATTACGTGAAGTGACTA

>V300080312L3C001R0641378276

GTTGGTTACTTTTCGAGTAAGTTTATGGCTGTAAATAATTTTG

>V300080312L3C004R0360786044

CACGTTCAGTTTTCAATTTTTCCATCTTTTCTACGGCTTCCAA

>V300080312L3C001R0350078367

TTCCTTTATTACCTTTGGTCGAAGTTGAGCTTCAATCTCTTCG

>V300080312L3C001R0400026849

GCTTCAGCTCATCGCATAAATGCTTTTGATTGTTCTGCTTTCG

>V300080312L3C006R0691347996

CTTGCAGTTCTGCCTCCTCTGAGTGTAAGCGTGGATCACGCCA

>V300080312L3C001R0660504332

GCCAAGTTTTTTGGTTTTGTTGGTCCCTGGCTCAATGGTTTCG

>V300080312L3C005R0290829277

TGCAGGTAACGAAAGATGGCTGGCGACCAGTAGCGCTCCTTGT

>V300080312L3C004R0671266227

GAAACGATTGTGTAGATAGACTAAGATTGCGAGCCAATCGCGA

>V300080312L3C003R0460273120

TACTTCTATTGCCGGTTGGGTGTATATATTTGAAAGTATCTGG

>V300080312L3C004R0690865996

TATTAGATTTGATGTTTTTCAAATCGATTCCATGTAAATCTAT

>V300080312L3C006R0450669595

TTTTCTAAGCAAAACATCTGTAAAATTCTGAAAATGACCTGTC

>V300080312L3C002R0510867958

TTGCCAAGCGCTTCCTTTCTCCGTTCGTCTATTTGCTTTATTG

>V300080312L3C003R0681189310

AGACGCCCGGAAAACAGAAAAAAGAAGAAGCCTCGACCTCCGG

>V300080312L3C006R0150690582

TTACATGTGGCTGTCAAGCAGGAGCAAGCAGGCGCTTCAAACG

>V300080312L3C003R0230180977

CTCTGGTGGATCAGAGACTCAAAAGTTGCTTGACACCTATACG

>V300080312L3C005R0080819244

TGTGCGCATCTTAAAGCTATCCAACGATTTCAAGATCCAAACA

>V300080312L3C003R0680122769

GACGCTCACGAAACGAAACGAAGAAAGCTGAATGATTAGTGTA

>V300080312L3C001R0540722204

AACGATAGCGTGAAGTTGTGGGAGAAATCCGGGCTTGTTTGCC

>V300080312L3C003R0581222077

CGATTGTGGAACACATGGCAAAGAGAGGATGCAAGCTGTAAAG

>V300080312L3C003R0320298186

CATTGCAGCTACGAAAGTGTTTGTCCGCTTCGCTCTCCTGCCC

>V300080312L3C002R0280122812

TTTTGATATATCTTGCCCTTTTCTTAGCGGTGCCAATATGCTG

>V300080312L3C005R0670427512

TATGGAAATCAAGGTCCAGCAATCCTAGGCTTCCTTTTTCATA

>V300080312L3C005R0510157246

CAAACATAGGCTTCAAGTTGCGACCTTTCAGATGTTTGTGAAG

>V300080312L3C006R0580848978

TCGACAGTGGCCCAAATAAAGAAAGCTGCATCCACTGTTCCTG

>V300080312L3C002R0701358151

ACATGGCAGAGTTGTATCTGATTATTAACTTGATCCTCCTTTG

>V300080312L3C006R0051324854

TACCCATACAAGTCCCTAAGGTAAAATTGCAAGATTAAAAGAC

>V300080312L3C002R0650732667

GACCCACATACAAGCGACGAGCTTGGCGAGCCAAAGTTGCGTT

>V300080312L3C002R0691007997

TGCATTTATTCAACTGTGACGGACTTGGCTAGGTTTCTAGGGA

>V300080312L3C006R0171189932

CAAACAGTCAACTGTCTGAGGCACACGAATGGTCTTATAGTCC

>V300080312L3C001R0630539997

CCTTAATATGACCAGGAAGGCGGTAAAGTCCATCAATAATTTC

>V300080312L3C005R0170570354

AAACAGTATCGCATGGCCAAGATAGTATCAGCAGTCTCGCCAG

>V300080312L3C005R0390340454

AGTAAGCTCTTCAAAGATGGCACGTGTCTGTATATGCACAAGA

>V300080312L3C001R0141221986

AATCTGCACCGGAAACCAAAGCAGTAAAGCTTACCAGCTCCTT

>V300080312L3C003R0230172491

CCGTCAGCAGCGAGACCTGTCAAGTTTCGGGATAACTATCCGG

>V300080312L3C003R0360189075

GGAGACTGCATACATATTTGCCGCGGAATGGTTTAAATGAACT

>V300080312L3C003R0340538795

TGATAATCTGGACCAACTCAGAACATGGCTGAATCCTAGCCCT

>V300080312L3C006R0681012669

CAAGCAGGATACTTGCAGGATACCCCGCGCCTCGATTGCATCT

>V300080312L3C001R0370931373

CCTCGATTGCATCTATCCTGTGCACGATCGAGAGAGGGTCCCT

>V300080312L3C001R0150421554

CGGCTGTTTGGGTAGCATGTGAGCAAATTTGTTCTTTTTAATG

>V300080312L3C003R0470132427

CAATCTTTGTTCGTGGAAGAGCAGCATGCTATCCCCAGATTGC

>V300080312L3C005R0460324557

GCAAGTCGTGTGGGCTGCGTTTGATGCTCCTAACCAGGCCAGA

>V300080312L3C002R0220643368

CCGCTGTTGTGCATAATCAGCCGATACGTGCAACACCGTTCAC

>V300080312L3C001R0610943672

TACCCGGGACAACCGTCGTGTCACCTCTTGCAGGCCGTGGATA

>V300080312L3C005R0141077728

GGAATGCATTTTTCTGATGAAAATATATACAAGCAAATAAGCG

>V300080312L3C005R0020876376

GTGCTCGGGTCACCTCGTTTTCTTCATTGCGGTATTGCTCTCT

>V300080312L3C006R0280286735

AGAGTTATCGCATCCTCCAGATGCTTCTCCGTTGGTTCCGCCA

>V300080312L3C006R0210137749

ATTTACTGTTAAGAAAGGATTAGAATCATGTACAAGTTTGGAG

>V300080312L3C004R0390588225

TACCGGCAGTGCTCGAACGTTTTCCTATCACACCCATTGATCC

>V300080312L3C002R0720176824

TGCATTTATCAGGATCCTGGGCTACATTTGTAGAACCGAAATC

>V300080312L3C004R0540970503

GCTACATTTGTAGAACCGAAATCCAGCAGGAATGCGAATGACG

>V300080312L3C003R0320271073

CGTTGCACCGAGAAAATAGTGGAAGATATAATATGGGCTCCAG

>V300080312L3C001R0120130451

TGTATGTACGAGAAATACCAAATACGTTTGATTTACCCTCGCT

>V300080312L3C005R0670977637

CTGCAAGCCAGCTATGGAACGGCGTACGCAACTGTCACGAATC

>V300080312L3C005R0461260168

GGCGTACGCAACTGTCACGAATCTGCGCCATTCAGGATCCTTC

>V300080312L3C003R0711085998

CGGATCGCCTGAAGCAATGTATTGCGAGACGCGTTTCTTTCTT

>V300080312L3C001R0150009330

GATCGAATTTTGCATCATGGGCATAGCAGCACCTGGTTCGAAC

>V300080312L3C002R0370183349

TCATGGGCATAGCAGCACCTGGTTCGAACAATTCAAGGCAAGT

>V300080312L3C004R0591113706

CACAACAAATTTTTGCTATGCTACAACAAGTTCACTTTCTTCG

>V300080312L3C002R0621116636

GCCACGTGATGCCAAGTCGGCCTACCTTTGCGAGTGGGAAACA

>V300080312L3C005R0240317017

ATTATCTCGGGCAATGATTGCCGCTTGACATATTACTGCCCTA

>V300080312L3C003R0411320150

TCATGACATATTACTGCCCTAACCCAAACATAATCCAAGGATG

>V300080312L3C002R0471384101

CGACAACGACAAACGGAAGCCAGCTTCTTTATTCCATTCTTTC

>V300080312L3C002R0490390172

CTTAGGTAAAGAGCGATACACTATATCTCTAATTGCGCAATCT

>V300080312L3C004R0341356060

TATGGTACTACCCAAAACTTTAAATTATATATAATCCGATGGA

>V300080312L3C002R0340819114

GAACTTGACGAATCAATGCACAGTACCTCCCGACAGCTGATAC

>V300080312L3C006R0580981092

CAAGCGACATTTCCGCATCAGCGCGCCGTGTTCCACAGCCAAC

>V300080312L3C002R0670600496

CTATCACTATACTTGACGCTAGACGTACAACTTTTGTCTGACA

>V300080312L3C006R0700953990

CGAATGATACCAAGGACGTCCCAGCATCCGAGTCTTTCCTAGA

>V300080312L3C003R0301377528

CTTGGCAGTCTTGACGACTGCTTTGGGACGTTCGCCTCCTGAC

>V300080312L3C006R0120384178

AACGCGGATTGATTAACTGAGCCACGCCCTAGATTCCTGGCTT

>V300080312L3C005R0170496476

GCGATGCCACCCCCATCTACTCGTAAGCGTGCAACAGCTACTT

>V300080312L3C006R0420240551

TACTCGTAAGCGTGCAACAGCTACTTTTCGCCGGGAAGGATAC

>V300080312L3C001R0080832414

TACTGCCCTGACGCCAACTCAAACTGCCAACCCCTGGTGAACA

>V300080312L3C002R0310896057

AGCAAATCATCATCCGAGATAACTGCACAACCGGCACCTACTG

>V300080312L3C002R0570499202

GGACCACGAGGGAATTGGGGGAAATAGGATGAAAACATAGAGG

>V300080312L3C004R0470278850

GAATGCAAACTCGTGTCGAGACAGCATGAGGTGTGGAGAAAAG

>V300080312L3C006R0430204550

GCACTGTCAGCAATACGCTCCACCTTCCTTCTTTTTACAAACC

>V300080312L3C002R0061360172

CCAGCACCGCCTCTTCCACTGCTGCCGCCAGGGAAGCCTCCGA

>V300080312L3C001R0150851479

TGCGGTCTGCAGCATCCTTCGATATTCTGCAAAGTAGAAGTGA

>V300080312L3C003R0270727137

TGCAGTCAAACCGGGAAAATTGTACCTCTGTTGCTAGCATCTG

>V300080312L3C002R0070966404

ACAATCCGACAATGCTTCCTCGAATTTTTTAAGCTAAATATGT

>V300080312L3C001R0520217036

GCACTGCTGCACGGTTAGAATACAGCTTGGAGTTCATGGCATC

>V300080312L3C002R0520741843

TTTTGTTCCTCAATTGCTCGAGCCTTTTTCAACAGCGTTCTGG

>V300080312L3C003R0170260131

ACCATCGATATTGCCCGATCCAAGCTGTTTCCAGCCAAAGGCC

>V300080312L3C002R0560473348

ATTTGGCGATGCCTCTGATAGCAAATCGATCCATGAGTTTATC

>V300080312L3C003R0370520345

TACTGTTGAAATGATCTCAATATCTAGCATTTTCAGATATTCA

>V300080312L3C002R0600982422

CCATACATCCTTGGATGGAGTGATTTAAAGACCAGGATGACTT

>V300080312L3C001R0260337002

GGATTAGCAGAGATTCGAAATAGGGTGTACTTCGGACAGACTG

>V300080312L3C003R0590495768

TCGTCATTTAGAGTGCTTGCGAATGGGGACAATCAAATCCTAT

>V300080312L3C003R0150921257

CATTGGAGATATTGTCAGTGCTGTTTCGATTTCAGACCGAGGA

>V300080312L3C005R0500951488

TGGAAATACATAACGAATAGAGGTTTATGAAGGCCGAGCGAGT

>V300080312L3C002R0420737687

AATAACTTGTTTGTTTCTGAATAAATAAACTCGAAGCTCATGT

>V300080312L3C001R0340249133

CACAAGTCATCGCTCTTGATGTTGAAAGCTGAGGGCGGCACAA

>V300080312L3C001R0210317118

GATCGAGAAACCTTCGCTTCGAGACTGCGACGCAGGACACCTA

>V300080312L3C006R0640552371

GATGAAACCAACAAGAGTCCCTTGCGAAGTCGCGTCGCTGCTA

>V300080312L3C005R0451300446

GATTCTCTGCCCGCGTTCGGTCCACCGCTTCCTGAGGAGGCGT

>V300080312L3C004R0570799518

CGCGTGAAGGCATTTTGAGCAACCTTGTCGAGCGTGTTAGTAA

>V300080312L3C004R0330102475

GCGCTGGCGCTGTTACCGGTGACGATGTTGTTCCAACAGCTTC

>V300080312L3C003R0150138322

AAATCCATAAAGGAGCCCATTTTACCCTTTCGGCATGGGAGGT

>V300080312L3C003R0340806921

CCATTCTTCTTGTGCTTGACCATTCAAGAAAGAATTCTTTTCA

>V300080312L3C001R0541093309

GGTCAAGAAAATGTTCCAATTGATGCCGTTGGATAACATGGAC

>V300080312L3C004R0711342275

AAAAGAATTAATCGCACGTTTTGTCCGTCAATGTCCATTTTGT

>V300080312L3C005R0210403409

CAATGCAAGATGGCACCTTTTTCGATTCTAGAGCACACGAAGA

>V300080312L3C001R0590130159

AAGAGCAACAAAACAGAGAGCATTAGTATAAAACAACCACCTG

>V300080312L3C001R0720368863

TCGAGACTTCGCAACAATTTCTATTATACAAGTACCTGTCGCA

>V300080312L3C002R0260606608

CCCACACTATACTCCATTGCTGAATATTTCTTCATAAAGAATC

>V300080312L3C005R0120345585

ACTTCGATCTGTTCTCGAGCTCGCCCCTTTCCCATCTGCGGTT

>V300080312L3C006R0370978227

CAAGAATGCTCAAAGCGAAAAACCGCAAGCAGGTATGCCTCGC

>V300080312L3C004R0580812105

TCATCATAATCGAGAGAACCTATATAGATGTAAAGCATGAATG

>V300080312L3C005R0510052414

CCCCGCTTTCGGACTTTTAATTTTCTTCATTTCCATGATTTGC

>V300080312L3C001R0641343659

GCCGGGGGGGAGTTTATCCACCATTCAGCTGCTTGTGGGAGCA

>V300080312L3C001R0060817426

CTCCTTCGAGCCGTTGACAATTTCTACACACATTACAAACAAG

>V300080312L3C002R0570567488

TATGAGGTCGAGTGCCTAATTTGAGGTTATCCTCTCAAGTTGA

>V300080312L3C001R0260033078

GCCTAATTTGAGGTTATCCTCTCAAGTTGAAAGGCTTGAGTCG

>V300080312L3C006R0471112500

GGATACCTCTCATGTTAATGTCTAGCTTTAAGGTTGTAAATCT

>V300080312L3C003R0490623905

CATGTTAATGTCTAGCTTTAAGGTTGTAAATCTCAAGCAGACG

>V300080312L3C002R0071201187

TAATGTCTAGCTTTAAGGTTGTAAATCTCAAGCAGACGACTTG

>V300080312L3C005R0700006012

GTCTAGCTTTAAGGTTGTAAATCTCAAGCAGACGACTTGAAAT

>V300080312L3C003R0490549245

TCACATCTAAAACGCACTTTTATAAATAAAATGAAAGCTATGT

>V300080312L3C002R0400225931

TGTTGCTTTCGGAACGGGTCGGTATTTCCTATTTTTCAATCTG

>V300080312L3C005R0460550799

TGCTTTCGGAACGGGTCGGTATGTCCTATTTTTCAATCTGTGG

>V300080312L3C006R0651066620

CGGAACGGGTCGGTATTTCCTATTTTTCAATCTGTGGTAAAGA

>V300080312L3C001R0081249872

GTAAAGACTTTGGAACGGGTCGGAATTATTGAGAGGGAGCAGG

>V300080312L3C002R0051390710

AATTATTGAGAGGGAGCAGGGTGCTTGTAAAATTTTTTTGCTG

>V300080312L3C003R0060190664

GGCACAACACAGGCATACTAAATACTTTACGCAGGAACATACA

>V300080312L3C001R0040766028

CAGGCATACTAAATACTTTACGCAGGAACATACAGGCAAAGAA

>V300080312L3C002R0060168132

TACTAAATACTTTACGCAGGAACATACAGGCAAAGAAGAAATT

>V300080312L3C005R0590196373

CATACAGGCAAAGAAGAAATTACATCTACTTATAGCATGACCA

>V300080312L3C001R0260482427

ACTTATAGCATGACCATAGGAGACATGTTTAAAGCTAATAATG

>V300080312L3C004R0041353016

TATAGCATGACCATAGGAGACATGTTTAAAGCTAATAATGATA

>V300080312L3C003R0050530288

GCATGACCATAGGAGACATGTTTAAAGCTAATAATGATAATAA

>V300080312L3C002R0521136023

GACCATAGGAGACATGTTTAAAGCTAATAATGATAATAAAGAC

>V300080312L3C005R0360751556

CATGTTTAAAGCTAATAATGATAATAAAGACAATAGAAAAGGA

>V300080312L3C002R0340042907

CAATAGAAAAGGATAATGAAATAAATAGCAAATTGTACAATAA

>V300080312L3C005R0640651936

ATGAAATAAATAGCAAATTGTACAATAAAAAGAAAAAATGCAG

>V300080312L3C002R0240142027

CAGTTGGTGTAGTTTCGCCAAGTGGGGACAGGCTGGAAAGTAA

>V300080312L3C002R0371000478

CGGTGTATTCTTCACTGTATGGCCGTAAGTTAAAGACTACTGT

>V300080312L3C002R0200598818

ATTCTTCACTGTATGGCCGTAAGTTAAAGACTACTGTGCAATT

>V300080312L3C002R0571162459

ATGGCCGTAAGTTAAAGACTACTGTGCAATTAAAGGATATATC

>V300080312L3C006R0110665688

TGGCCGTAAGTTAAAGACTACTGTGCAATTAAAGGATATATCG

>V300080312L3C005R0150930891

TAAGTTAAAGACTACTGTGCAATTAAACGATATATCGTGGAGC

>V300080312L3C003R0661251547

CTGTGCAATTAAACGATATATCGTGGAGCCAGTAAGCAAAAAA

>V300080312L3C003R0371341129

TGCAATTAAACGATATATCGTGGAGCCAGTAAGCAAAAAAAAC

>V300080312L3C002R0650743609

TCGTGGAGCCAGTAAGCAAAAAAAACCCCCGGAAAAGCTAGAC

>V300080312L3C002R0700913605

AAAAACCCCCGGAAAAGCTAGACGGTGTAACAGTGCTGGCGGT

>V300080312L3C005R0200425013

TGTAACAGTGCTGGCGGTTAGGCAGGACACTTTAGAGGCTCGA

>V300080312L3C002R0251310096

GCGGTTAGGCAGGACACTTTAGAGGCTCGAGGAGCTAAGCATA

>V300080312L3C003R0591014812

AGAGGCTCGAGGAGCTAAGCATAGAAAAATGAGGTATGATTGG

>V300080312L3C004R0271282393

AGAAAAATGAGGTATGATTGGCCGATCGGAATGGCGAAACGGA

>V300080312L3C001R0391175745

AAAATGAGGTATGATTGGCCGATCGGAATGGCGAAACGGAATG

>V300080312L3C004R0011057130

GGCGAAACGGAATGGAGTGTAACCAGATATTGACAGGGGCCGG

>V300080312L3C004R0361378389

CCAGATATTGACAGGGGCCGGAGAGCGTGAATGGGTGTGAGGT

>V300080312L3C006R0101078327

GATATTGACAGGGGCCGGAGAGCGTGAATGGGTGTGAGGTGGA

>V300080312L3C004R0660917430

CCGGAGAGCGTGAATGGGTGTGAGGTGGAAAGCAGATGCAATG

>V300080312L3C006R0280346091

AGAGCGTGAATGGGTGTGAGGTGGAAAGCAGATGCAATGACAA

>V300080312L3C001R0570754720

AGCGTGAATGGGTGTGAGGTGGAAAGCAGATGCAATGACAAGG

>V300080312L3C002R0650281430

TGGGTGTGAGGTGGAAAGCAGATGCAATGACAAGGAGTGTTGC

>V300080312L3C006R0230650120

TGAGGTGGAAAGCAGATGCAATGACAAGGAGTGTTGCAAAATG

>V300080312L3C004R0470339817

GAGGTGGAAAGCAGATGCAATGACAAGGAGTGTTGCAAAATGG

>V300080312L3C005R0600695883

ACAAGGAGTGTTGCAAAATGGCGAGGTGCAATGAAGTGCAAGG

>V300080312L3C005R0300759990

AGTGCAAGGACGCGCCGGCATGCCGAAATATGCAGCATACGAT

>V300080312L3C001R0180781908

AAATATGCAGCATACGATCTAGTGTATTCAAGCTGTTGACTCT

>V300080312L3C002R0140798144

CGATCTAGTGTATTCAAGCTGTTGACTCTGACTGTCTAAGTCC

>V300080312L3C002R0280260330

AGCTGTTGACTCTGACTGTCTAAGTCCAACAAAATATGCTTAT

>V300080312L3C002R0721073823

ATATGCTTATAGTATACGACTCAGTAACGAGCTACGGTGTCCA

>V300080312L3C002R0120464166

ATACGACTCAGTAACGAGCTACGGTGTCCATCTTTTTCTTCTC

>V300080312L3C004R0720023756

TGTCTTTGGGTGTGACATAAAAAGAACAAAAAAGCAGCATTAC

>V300080312L3C003R0100471826

TGGGTGTGACATAAAAAGAACAAAAAAGCAGCATTACAACAGT

>V300080312L3C005R0450140488

GTGTGACATAAAAAGAACAAAAAAGCAGCATTACAACAGTACG

>V300080312L3C005R0280925325

AAAGAACAAAAAAGCAGCATTACAACAGTACGCGAAAAGCGAA

>V300080312L3C001R0040121444

AAAAGCAGCATTACAACAGTACGCGAAAAGCGAAAAGGGAATG

>V300080312L3C001R0150680884

CAACAGTACGCGAAAAGCGAAAAGGGAATGACGATGTCTTGCA

>V300080312L3C004R0720481565

TACGCGAAAAGCGAAAAGGGAATGACGATGTCTTGCAAAAGAA

>V300080312L3C002R0171370714

ACGCGAAAAGCGAAAAGGGAATGACGATGTCTTGCAAAAGAAC

>V300080312L3C006R0120295666

AAGGAAAAACGCAATCAGCGGTACGCAGAAGTAGGTGAGAGCA

>V300080312L3C003R0561388193

AAAACGCAATCAGCGGTACGCAGAAGTAGGTGAGAGCATTGTG

>V300080312L3C005R0430813460

GGTACGCAGAAGTAGGTGAGAGCATTGTGTACTGGGATCCATT

>V300080312L3C001R0430850892

GGGATCCATTTTTATCAGTTTTCGGTGACAGAGTTGAAAAAAA

>V300080312L3C005R0390979673

TCCATTTTTATCAGTTTTCGGTGACAGAGTTGAATAAAAGAGA

>V300080312L3C005R0530441103

CATTTTTATCAGTTTTCGGTGACAGAGTTGAAAAAAAGAGAAA

>V300080312L3C004R0620702602

TCAGTTTTCGGTGACAGAGTTGAAAAAAAGAGAAAGGGTTGTC

>V300080312L3C006R0721154818

CACAGTTGAAAAGAAAAGAAAGGGGTGTCATCACTAACAAGTA

>V300080312L3C005R0450878914

AGTTGAAAAGAAAAGAAAGGGGTGTCATCACTAACAAGTAGCA

>V300080312L3C001R0700442472

GTGTCATCACTAACAAGTAGCATCCATCATTTTTCTTTTTGGG

>V300080312L3C006R0110522568

AGGGTAATAACCAAGGTCAAAAACATACGCAGTTCAATTTTGG

>V300080312L3C005R0340561860

GGGTAATAACCAAGGTCAAAAACATACGCAGTTCAATTTTGGG

>V300080312L3C004R0421009677

GGGTAATAACCAAGGTCAAAAACATACGCAGTTCAATTTTGGG

>V300080312L3C004R0630684759

AATAACCAAGGTCAAAAACATACGCAGTTCAATTTTGGGAGTC

>V300080312L3C001R0451158566

AGTTCAATTTTGGGAGTCCAAAAGTTATTGCATTTACCTGTGA

>V300080312L3C005R0660248860

TTCAATTTTGGGAGTCCAAAAGTTATTGCATTTACCTGTGAGG

>V300080312L3C001R0531002277

AAATTTTCGCGGAATGTAAACTTCCGGGAGGAAGGGTTGCGGG

>V300080312L3C004R0601166717

GGAAGGGTTGCGGGAGGTTCCACCACTAAATTTCACTCAATGT

>V300080312L3C002R0430345896

TGCCTAATTAGTGGCAAAAAGGTATTTTTGTAGGAAAGAAAAG

>V300080312L3C001R0391274877

GGCAAAAAGGTATTTTTGTAGGAAAGAAAAGCAAAAGCAAAAC

>V300080312L3C005R0561055731

GCAAAAAGGTATTTTTGTAGGAAAGAAAAGCAAAAGCAAAACG

>V300080312L3C003R0630220461

ACTGAAAAGGGCGTTGCTAAAGAGCAATTCTTTCCGGGCCTGG

>V300080312L3C003R0391008174

AATTCTTTCCGGGCCTGGCAGCTCGCAAAGACTTTGACGGTGT

>V300080312L3C005R0240346947

AAAGACTTTGACGGTGTCAAATCGGAGGGGGCTTGCACAGACC

>V300080312L3C005R0170111628

ACGGTGTCAAATCGGAGGGGGCTTGCACAGACCAAAGAGATGG

>V300080312L3C005R0130149444

GGTGTCAAATCGGAGGGGGCTTGCACAGACCAAAGAGATGGTA

>V300080312L3C003R0480424287

TTTGGGTATCGCTGAGGTTTCAAAACATTCACCGAACAACTCT

>V300080312L3C001R0380225667

TGGGTATCGCTGAGGTTTCAAAACATTCACCGAACAACTCTGG

>V300080312L3C003R0310178513

CGCTGAGGTTTCAAAACATTCACCGAACAACTCTGGATGCTCA

>V300080312L3C005R0340250770

CGCTGAGGTTTCAAAACATTCACCGAACAACTCTGGATGCTCA

>V300080312L3C006R0650413479

AACATTCACCGAACAACTCTGGATGCTCAAGCTTATTGCTTCA

>V300080312L3C004R0211311614

GTAGGAAAACTTTAGACTGGCAATATGGATTATGGAGATAAAG

>V300080312L3C005R0460977234

ATTATGGAGATAAAGACTCGACCGCTTTCTTTCAGGAGAAGCA

>V300080312L3C005R0020465129

CTTTCCATAAGGTATATTCGCTTTGAAAACCTACATTGGTTTT

>V300080312L3C004R0670445499

AAGTGAGAAAAAATAGATTCAAATCTGTCTTTTTTCCTCTCTC

>V300080312L3C004R0190451894

TAGATTCAAATCTGTCTTTTTTCCTCTCTCTTGCTCTGGTTAT

>V300080312L3C005R0120206266

TCAAATCTGTCTTTTTTCCTCTCTCTTGCTCTGGTTATGCCAT

>V300080312L3C002R0100688629

ATCTGTCTTTTTTCCTCTCTCTTGCTCTGGTTATGCCATTTTC

>V300080312L3C006R0221287276

GTCTTTTTTCCTCTCTCTTGCTCTGGTTATGCCATTTTCAAAG

>V300080312L3C004R0560466698

GCGAATGGCTCATTAAATCAGTTATGATCTACGCGAGCGAACA

>V300080312L3C003R0450917117

GATAAAGCCAACGTAAGGGTAAAACCTTTCGCCTTGGTGAATC

>V300080312L3C002R0561003354

CAACGTAAGGGTAAAACCTTTCGCCTTGGTGAATCATAATAAT

>V300080312L3C006R0140145349

CGTCAGCCTTTAGCAGTGCACAGCTTCCTTCACTGGTTGCTGT

>V300080312L3C001R0720063094

TGCTATGGCTTTAATAGGATCTTCCTTCGGGGAGTTCCTGATT

>V300080312L3C004R0410178565

AATAGGATCTTCCTTCGGGGAGTTCCTGATTACCGTGAGCAAA

>V300080312L3C003R0481013782

CTTCCTTCGGGGAGTTCCTGATTACCGTGAGCAAATCAGAGTG

>V300080312L3C002R0520642026

GTTGGATCATGAGACCTTCGGGTCTCCTGTCTTCTTAGAGATA

>V300080312L3C001R0521220178

GTGAACCTGCGGAAGGATCATTAAAAAGTTGTGGAAATCGTGG

>V300080312L3C006R0400587760

CCTGCGGAAGGATCATTAAAAAGTTGTGGAAATCGTGGTGACC

>V300080312L3C003R0441168147

AAAAAGTTGTGGAAATCGTGGTGACCTCTATTGGTGAGCCGCG

>V300080312L3C004R0340556909

ACCTCTATTGGTGAGCCGCGATTCTCTCCTTTTTGTGAAATGT

>V300080312L3C004R0211279173

CCTCTATTGGTGAGCCGCGATTCTCTCCTTTTTGTGAAATGTT

>V300080312L3C005R0170153975

CTCTATTGGTGAGCCGCGATTCTCTCCTTTTTGTGAAATGTTC

>V300080312L3C002R0510945732

ATGTTCTGAGGGATTGCTCCAGATCTCTCGACCTTTTATTTTA

>V300080312L3C004R0281136769

GACCTTTTATTTTACATATTTGATTGACTGTTGTTTAACAAAT

>V300080312L3C001R0070827451

CCTTTTATTTTACATATTTGATTGACTGTTGTTTAACAAATTG

>V300080312L3C005R0711363402

TTACATATTTGATTGACTGTTGTTTAACAAATTGAAAGTTTTG

>V300080312L3C001R0320119124

TGACTGTTGTTTAACAAATTGAAAGTTTTGGATCAGAAATGAT

>V300080312L3C005R0511020709

GAAATGATTCAAGACGATAAAATTTCAAAACAACTTTAAGCAA

>V300080312L3C006R0560960810

GACGATAAAATTTCAAAACAACTTTAAGCAATGGATCACTTGG

>V300080312L3C006R0210874655

ATCTTTATTAACCCCTAAAGGTTTATTTTTTGATAAATCTTTG

>V300080312L3C004R0430633641

CTAAAGGTTTATTTTTTGATAAATCTTTGGATTTGCGGTGCTG

>V300080312L3C002R0211256871

TGGATTTTCATCCGTTCAAGCTACCCGAACAATTTGTATGTTG

>V300080312L3C006R0650481467

GTTGTTGACCCTTGATATTTCCTTGAGGGTTTGCATTGGTATC

>V300080312L3C001R0160459238

TGATATTTCCTTGAGGGTTTGCATTGGTATCTAATTTTTTACC

>V300080312L3C003R0281380642

CTAATTTTTTACCAGTGTGCTTCGAGATGATCAAGTATAAAGG

>V300080312L3C006R0350471457

AGATGATCAAGTATAAAGGTCAATCAACCACAAATAAATTTCA

>V300080312L3C006R0450948559

GATGATCAAGTATAAAGGTCAATCAACCACAAATAAATTTCAA

>V300080312L3C002R0691205809

CAAGTATAAAGGTCAATCAACCACAAATAAATTTCAACTATGG

>V300080312L3C006R0080666922

AAATAAATTTCAACTATGGATCTGAACTTAGATGGGATTACCC

>V300080312L3C004R0060125154

GAACTTAGATGGGATTACCCGCTGAACTTAAGCATATCAATAA

>V300080312L3C003R0430941440

CAAACCGGATTGTAAACTAAGGACGTGCTATCCAGGCTCTTTG

>V300080312L3C002R0200589900

GATTGTAAACTAAGGACGTGCTATCCAGGCTCTTTGGACCTTC

>V300080312L3C006R0140282690

TTGCGGAAGCCTCTGGTGGAGCGTTGGTCTGCCTTGGCCCTTC

>V300080312L3C001R0211345267

GTCTGCCTTGGCCCTTCTGAACCTATAGTTGGCTTTATGGCTC

>V300080312L3C002R0441230092

TTGGCCCTTCTGAACCTATAGTTGGCTTAATGGCTCTAAACGG

>V300080312L3C002R0020878527

TATGTAAGATGTTCTTGCTGCTTGAATATGAGCTTGAACTATC

>V300080312L3C004R0560785274

ATAAGGCGTCAAAGAGCACGCTCATCAGACACCACAAAAGGTG

>V300080312L3C006R0420233328

CTTCCAGGCTTGGAGCTCCTGGGCACGCTTAACAACCAACTTA

>V300080312L3C001R0271127076

CATATTGATTAGGTGACGATATGTGATTTTTAGTCTTTCTGAT

>V300080312L3C002R0710776901

CTAGCTGGAATGTCCTAATGCCTATAACCATTCGGTTTTTCGA

>V300080312L3C004R0150210213

AGCTGGAATGTCCTAATGCCTATAAACATTCGGTTTTTCGACC

>V300080312L3C003R0470834795

GAAACCAAATGCTATAGGATGTACCAATGGACATGACTACCGG

>V300080312L3C001R0330728117

ATGGACATGACTACCGGCAACCGAGGACTTTTTCTCTTTGAGA

>V300080312L3C005R0670707863

AGGACTTTTTCTCTTTGAGAGAAAAATCAGCCAAGGTTTAGAG

>V300080312L3C005R0650845169

GGACTTTTTCTCTTTAAGAGAAAAATCAGCCAAGGTTTAGAGA

>V300080312L3C002R0460486400

AAGGTTTAGAGACTAATGCTGGATGGGGTTTCTAAAAGAAACC

>V300080312L3C006R0211321776

AGGTTTAGAGACTAATGCTGGATGGGGTTTCTAAAAGAAACCT

>V300080312L3C005R0690342793

GGGTTTCTAAAAGAAACCTTGTGATATAGTCCAGTTTTAAAAC

>V300080312L3C001R0280101488

CTTGTGATATAGTCCAGTTTTAAAACAATAACTGCTAATTAGT

>V300080312L3C001R0250641466

CCAGTTTTAAAACAATAACTGCTAATTAGTGACGCGCATGAAT

>V300080312L3C003R0430735030

AGTGAAATACCACAACCCTTATAGTTTTTTTACTTAAATAATC

>V300080312L3C005R0071054345

GAAATACCACAACCCTTATAGTTTTTTTACTTAAATAATCAAG

>V300080312L3C003R0571236054

GTTAAGGGACGCTAAAACCAGACCTTAAAAGCGCAAGAAAGTG

>V300080312L3C002R0281199458

TAAGGGACGCTAAAACCAGACCTTAAAAGCGCAAGAAAGTGCT

>V300080312L3C002R0710541101

CGCTAAAACCAGACCTTAAAAGCGCAAGAAAGTGCTTAAATAG

>V300080312L3C006R0460515021

AGACCTTAAAAGCGCAAGAAAGTGCTTAAATAGATCTCTTATG

>V300080312L3C005R0650456867

GGTCGAGTGCCTAATTTGAGGTTATCCTCTCAAGTTGAAAGGC

>V300080312L3C004R0720312792

CCTAATTTGAGGTTATCCTCTCAAGTTGAAAGGCTTGAGTCGG

>V300080312L3C002R0410667388

TCCTCTCAAGTTGAAAGGCTTGAGTCGGTTACCTCTCATGTTA

>V300080312L3C004R0431002862

GAGTCGGATACCTCTCATGTTAATGTCTAGCTTTAAGGTTGTA

>V300080312L3C004R0500046277

CTCTCATGTTAATGTCTAGCTTTAAGGTTGTAAATCTCAAGCA

>V300080312L3C006R0101294658

TAGCTTTAAGGTTGTAAATCTCAAGCAGACGACTTGAAATCGA

>V300080312L3C005R0480168349

GAACAAAAGCTGCAGCCGATGTGGCGATTGTGCTACATTGACA

>V300080312L3C001R0650824568

AGCTATATCACCTGAACAGAAAGCTGAAAGGCCAAGATGCCCC

>V300080312L3C002R0471121425

TGAGATTCAGTCTCACTCAAAAATTTCAACTTGATTGCGGCAG

>V300080312L3C001R0200375029

AACAGCACCGTTCTGCCCATCCTGTACAACCATCTTTTGTTTT

>V300080312L3C005R0330348435

AACGTAACCAAAGCGACGCCCGCGTGAACGGCGCAATGACACT

>V300080312L3C004R0160899869

AAAGCGACGCCCGCGTGAACGGCGCAATGACACTGACGTTAAG

>V300080312L3C004R0710171349

AAGCGACGCCCGCGTGAACGGCGCAATGACACTGACGTTAAGT

>V300080312L3C001R0020180254

CTTCATCTATCCATCCGGGAGGTATAAGATGCTCGGACCTGGG

>V300080312L3C004R0270909338

GTATAAGATGCTCGGACCTGGGACCCGGCGCTCCACCTGCACC

>V300080312L3C002R0551178786

TCGTCAGGGGGTTCCCGCAACCGGCGGCATCCGAACTGTTCAT

>V300080312L3C005R0520531333

CGTCCTGCGGAACACGCAGTGGGTGATCGGCATTGTTGTATTT

>V300080312L3C002R0620592444

AAGGACTGGGTGTCATGACACTCGCTATCGGAGACGGCGCAAA

>V300080312L3C005R0200225820

AAATGGCTGGAACAAGTGGTTAAAATAGGCGCTTGTTCCCACG

>V300080312L3C001R0340407198

GGGATCCGGAACGGCGTGAGCGGCAGAGCTCGCCCCGATTTCT

>V300080312L3C004R0270226136

TAGAAGAAGGAAAACAAGGAGCGGCAGTGGCCGTGTTTGCCAT

>V300080312L3C001R0101118915

TGCCACAAGCTTCGAAATATCTCGATGATTCATTGTATGATTC

>V300080312L3C006R0580040395

ATTCGGGTATCGCTCTCTGATCTGCTTTTGCTTGTCGAGACGA

>V300080312L3C006R0430603105

TCGCAGTGAGGCAACGGCACGCGGGTGATGCCGAAGATCCGGG

>V300080312L3C006R0250311981

TCGCCGGATACAAAATCAACTACTAACTTCTTGGTTCTCGCGT

>V300080312L3C001R0170057342

TGCAAAGTCCAGTGTCTGTCAAACGATTTGTTCAACGTCGCAA

>V300080312L3C001R0690785333

CCCACTATTTCGTGACTGAGCTGGATCTTCAAGCTTTCTCTTC

>V300080312L3C004R0151234834

AGCAACAGCAAGAACAGCAGCAGTTGGTGCTTGCGGAGCAAGA

>V300080312L3C004R0371221079

TCGTATGCGGCAGGATGCGGAGGAGGAGGTGGCGGTTGTTGCC

>V300080312L3C006R0131021206

AAACCTAGGTCGCCGGATAGACTCTGAAGACCTGGATCGACCT

>V300080312L3C006R0701286376

TTGTTGTTGTTGTTGTTGTCGATGGTCTAATCGAGAAGCTTCC

>V300080312L3C001R0371316689

GATCACGCGCGTGCGCGCGAGTATATTGTCAATGATCCCATGA

>V300080312L3C001R0450477472

CATGCACTCCGAGAGGCTCTGTTGTTACATGTCAAGCACACGC

>V300080312L3C001R0090162400

TGTTACATAAGTGGAGGAGACTTTATTTTTCAAGTATCAAGTA

>V300080312L3C001R0280622050

TATCGCCTCCCACAGGTGGCAGCAAGTCGATCTTGAGGAATGT

>V300080312L3C002R0100078986

GCCTCCCACAGGTGGCAGCAAGTCGATCTTGAGGAATGTGACT

>V300080312L3C006R0520585409

AAGTTTGCTTTCTAGTCTGCTCATTTTTGGAGTGGACGCGTCA

>V300080312L3C003R0350300650

GCTCATAGATAACGAGGACTTAGAAAGACTATCGGAAAAAATG

>V300080312L3C003R0130198086

CGGTAAAGTTCAAATTGGAAGAGTCACTGGTAAAAGAGGCCAA

>V300080312L3C002R0671010287

GCGCGCATTTGAGAATAAAAGAGTGTGGTATACACACATACGT

>V300080312L3C006R0711073148

CTTTCACAGTAACGCGTACTATCTGCATCAGCAAGTGTCCAAA

>V300080312L3C002R0660591436

CCGCGGTATGCGTCTCATCAACTCTCCTAACTGGAACTTTTAC

>V300080312L3C003R0571011679

TCCTTGCGCTTTCACCACCGACCATGGCCGATCGTCAAGAAAG

>V300080312L3C003R0261031851

AACAGATCTTCGATCTAGTTTATGAACGGAATGAAAAGCGACG

>V300080312L3C006R0230508887

CGGGACGTCCACTTAGCAGACTTTCAACGCCTAGAGCTGATAG

>V300080312L3C002R0530153562

GTCGGTGAAACTGGTTCAGGAAAGACTACTCAGTTGACACAGT

>V300080312L3C004R0271328487

CGAGAATACCCTTATTAAATGTAAGCGGCAATGGAAGACGACA

>V300080312L3C004R0600576716

AAGCGTCGCACCAAGGATGAAGCAAGATCTTCGGGCCACTATT

>V300080312L3C005R0700082646

CATAACCATTTGTAGCACTGATTCTACATCGCTGTCTTGCACC

>V300080312L3C002R0271210357

TTCCTCGTTCCAGATGAGCTTGGCCACGATGGCTGATACGCGG

>V300080312L3C002R0670011429

TGTGAAAAGATATGTCCGGAGACCCTCTTCTTGAATCTTTTGT

>V300080312L3C002R0380000356

AGAAGCCAAAGCCTTTGCTGCGCTCATAATGTGATCGCGTGTG

>V300080312L3C003R0070580208

TGATTCTTGCAAGTGAGTCATGAGTAACATATCGCGAGCGGTA

>V300080312L3C003R0210155553

CGTGAAAGCAATATAATACGTTAGACAGTCGACCAGGCAAAAT

>V300080312L3C001R0461390318

AGCTTCAAGAACTTCCTTCAAACGAGTAAATATGTCTTCTTTC

>V300080312L3C001R0180827814

CGAGTAAATATGTCTTCTTTCTTTGTAAAAGCAACAGTCTTTC

>V300080312L3C003R0141237166

TCGATACCAGCCATAATACAATGCTCCTAGACTGCAGAGCTGC

>V300080312L3C001R0040351376

GGCAAATATCGTCGTTTCCACGTCTCATTCAGTGTATGCACGT

>V300080312L3C004R0381278590

CAAGATCATGGCGACATTTTCTTCAAAGCTGGCCAGGCATGTG

>V300080312L3C003R0600383834

TGAGTTCCTCTAAATTGATAATATCACATATTCACAGATGCCC

>V300080312L3C002R0070307166

CAATGCCGGAAGCTCAAAAGCTTGGTGCTAGAGGATATGTACC

>V300080312L3C005R0140739683

GTGATTAAATTCTGCTCAGTTACCCGCATCGCTTTGCATGACA

>V300080312L3C004R0550658365

CCTCCTTTTTATCATCTTTTACAAACATCGAGTTTCCATGAAA

>V300080312L3C006R0430352764

CCCTTCCATGTTGCGACATTTTATGAAGCTGTGCTTGGATGGA

>V300080312L3C006R0590781937

CCTTTTACCGGATCTGTTGCTTCTTTTATCACGCTGCCCACGG

>V300080312L3C004R0110491241

CCATGACATGAAGAGACTCGATGCATCAAGCCAAGGTAAGGAA

>V300080312L3C003R0040748507

GAGCATATCGATCCATTGGAAAGAGAGATCCGCGAGCTTGCAG

>V300080312L3C002R0120866947

TCTTTCTTCCTTGTCTCCAAGCATGTCAAGCTCTGCAGAGAGT

>V300080312L3C005R0531081121

CGATACAGATTGCATCAACTACATCAAGTCCTCCCGAGGCTAC

>V300080312L3C003R0321318795

AGCATTGAAGTAACTACGACTGCGGCTGCAGTCACCAGTTCAG

>V300080312L3C006R0440435001

CCGAGCTACCAGCCGAGTCAACATCCAGCAGTTTGCCAGTACC

>V300080312L3C006R0420437215

GTCAACATCCAGCAGTTTGCCAGTACCGTCATCAAGCGTCGAC

>V300080312L3C004R0660830644

GGCACCGGGGAAGAAGATTTGGACGGATTCTCGTATAGCAAAC

>V300080312L3C005R0040050114

TCTGTCATATTTTACAATACACATTTATCTGTGTTCTGATGTT

>V300080312L3C001R0470775659

AATCTGCGGTCTGCTCGGACGTCGGCCCAGCTGCTTGGTCGAC

>V300080312L3C005R0420450380

GCTTGTAATCCGACGGTGTCCATGTGCTGGAGGCATCCTTATC

>V300080312L3C002R0250980440

TGTAATAGGGATTGCAGATGAAAATGCACTGAAAACAGCCGAA

>V300080312L3C004R0670617584

GTTTCTTTTTGCTTTATATAGGGAAGGATGTCGAATGGGCTCT

>V300080312L3C005R0540179743

GCTTTTCGAGGGCTCTAACCATATTACAGGCAATTGCAATGCT

>V300080312L3C001R0691236537

CCGAAAAAAGGTTAAAAATAATGGGTCACTCAGAATCTTGCAT

>V300080312L3C005R0560375509

ACATTATAGTGCCGTTTAGTTCACGTTGGCATGAAAACATAGC

>V300080312L3C005R0510586758

CCCTTCCAGGTTTGGGAGCGAGGCGGTCTCCTGACGTCAAGAC

>V300080312L3C005R0490727239

GCTGAACTGAGGAATCATAAGCGTAAACAACTCTCCTGTTTTA

>V300080312L3C001R0490487849

CCTAAGACCATGCTTATTGGCAAACGGAGACATCGCCGAGTGA

>V300080312L3C005R0281315698

TTGGAGAACATGAATTGGGAACGACGATTGATGTGAGATGTCA

>V300080312L3C003R0700460702

TGTGAGATGTCAAGAAGATCCATTTACATACATTCGTGATCTG

>V300080312L3C004R0401323324

GGAAGGATAAGTTTGGGAAAGGACGCCAATCGTATAGAGCTAT

>V300080312L3C004R0131198451

TTTTCATTGTGTGCTGTAGAATATGCATAATGAAAATAAGGCT

>V300080312L3C002R0270771730

ACTCGCATGGATCCATTGCAATACGATCCCTACCAATGAACAA

>V300080312L3C003R0261177739

CTAACCCTGAACGGATACCGAACTCGCAACTGTACCCTCCACC

>V300080312L3C001R0270077275

CCTCACAAAGTATATACATCACAGCGATGGATGCCGTCGTAAA

>V300080312L3C004R0180617872

AATTTAGGCTCAGGATCTTGGACTGCATCGTTCAAGTGCAAGC

>V300080312L3C006R0180161853

CGTGCTTGATAATCCATTCTTCTTCGAGATCCGATTCGTCGGC

>V300080312L3C005R0401188917

AAATTCTTTTGGAACGTAGGATTCTTTGCATGGTCTGTTTCCA

>V300080312L3C002R0610128179

CTGTTTCCAATATGTTGGCAAAGAATGTGGCGACCTCCTCCGC

>V300080312L3C001R0040529916

GCCACGGAATCTACATCCATTGCATCTCCGTTGGGTTCCTTGA

>V300080312L3C002R0481378278

TGCATGCTTTCAGTAAAAAGACCAGACAAAACATTAGGCACTG

>V300080312L3C005R0270635432

TACATATCCATTTTCTTACCTAGTGTCTCCCAATTGGGTAAAT

>V300080312L3C004R0280265502

CGAAATCCACGATCACGGGCGTTCTAAAAGAGCAAAGGCAATA

>V300080312L3C002R0410413561

CCCTCCAAACTCCTCTTCGTCCCGCTCCTTATCCTTCACTATC

>V300080312L3C005R0400919698

GGAACACTTGATGATTTCGAGTTTGACGATAGCAACGGTGACT

>V300080312L3C005R0151233846

TAGTGCTCCAAACGATGCAAATGGTCTCTCAATCAGTAAAGCA

>V300080312L3C006R0180614141

ATTATCAATTAAAAAATTGAATATCATACCAGCCATTGATTAA

>V300080312L3C003R0710224710

AGAATCATTTGCGCTGAAGTGGACGATGCTTTGAATGATGACA

>V300080312L3C002R0700078768

CCCCATGGAATATGACTAGGCTTGACACCGCACTTCTTCCAGC

>V300080312L3C001R0630998974

GGATCTTATGATAGGGACACCTTCCTGTTGCTCGGTGGACTCG

>V300080312L3C004R0720362678

TACTTTTTTGACTTTGCTTGCGAAGTGTGGCTGTTTTGCTGTT

>V300080312L3C004R0360271874

ATGGCGGAATCCACGAATGGAGCCTTTTGGGAGGGCACATTCT

>V300080312L3C004R0340535240

GACTTAACCATGTCTATGTGGTCGATGTTAACGATCTATCTTT

>V300080312L3C004R0050912074

ATTCGGTAATTGCTCTCGTATTTGTTTTAATCAAAACAGGTGC

>V300080312L3C006R0281032489

ATTTTTTACCATGAGCGACATCGCGGATCGGATATCAATGCCC

>V300080312L3C004R0651216661

TTACATGACTCAACATCTCCGTGCCGATTTGTCCTACCAATCA

>V300080312L3C003R0370045277

CGTACCATCCTCCTTCATATCATTATTGCTCATTAGCAACAAT

>V300080312L3C004R0360376920

ACACAGACGCAAAGACCTTTGCTGTGAAGCAGTTCCGTAAACG

>V300080312L3C004R0211397747

TCTTTTTTCCATCCCCTCCTACTTTACATCCGCCTCATCATTT

>V300080312L3C006R0370564492

AACGCCGTTGTAACCAGTAAGACGAAGCAACTTCTCCTATATA

>V300080312L3C002R0111277653

CCTATATACACGTGATACAATCTTCTGGTTAGAGCACCAGATC

>V300080312L3C001R0410004557

TACATTTGCTAACAGGCAATTATAAGTGGATATAGTAGTAACA

>V300080312L3C002R0200748348

CATTTGCTAACAGGCAATTATAAGTGGATATAGTAGTAACAGC

>V300080312L3C002R0330757536

AGGAGAAGGGGCCAGCTAGGTGTAGATATACACAGACGATGGC

>V300080312L3C003R0500255601

CGAAGCTGCTGGTACCACACACGGACAACTTTAGAGACAGATG

>V300080312L3C005R0120747551

CTATATAAATAAACGTCAAATCTGGTTACCCTTTCCCTTTTCT

>V300080312L3C003R0420087065

CACTAGCAACGGTGCGGCCCACTACAGCCGGCCTCAGCAACAC

>V300080312L3C005R0430200282

GCAACGGTGCGGCCCACTACAGCCGGCCTCAGCAACACCAGCA

>V300080312L3C001R0500189229

GTCTGCCTCGGCTTCCTCCTGCTCCTCCTCTGATTCTTGCCCG

>V300080312L3C005R0010861633

GATCGCACGCATCTTTTCTATTGTAATACAATGTATATTATGG

>V300080312L3C002R0431045861

CTCTTGGGGAGGCCGAATACAAATTGTTGGCAACAACACCGCT

>V300080312L3C004R0601405261

GCCGAGGAGGCTGCTGCTGTTGCTCTGCATGCTCGTGGATGCT

>V300080312L3C005R0040219367

AGGGTAACTGATTTGAATATCCGTCTCCTTGCATATTTATTTG

>V300080312L3C005R0271159283

TTGTGGCGCATGCTATGTAGCAAAATTACAATGGGTTTATTGC

>V300080312L3C005R0670753341

ACACATATTTCTAAGCTACAGTATACGCTAGATATATTTGGCT

>V300080312L3C006R0110717460

TGAAGCAAGCATATGATCCTTCGGCATCGTTATCGCAGAGTCA

>V300080312L3C006R0580100859

TCGCCCCGCCTTGGCTCTTCTCCAAAGTCGCCCGGACACAGAC

>V300080312L3C005R0241396826

CGGGTGCTATCAAGCCACTGAGTTCAGGGGGCGGATATCCCGA

>V300080312L3C004R0270539306

CTATCATCATCCAAAGATACCTTGTTGAAATTGTGGGACCTCT

>V300080312L3C006R0640540389

GACAAGCGAGGTTTTGCGACTGGCGGTGCTGACAAGGAAGTCA

>V300080312L3C001R0160559243

GCACCCACAACCAAGTGAAACGAGATGGCTTACATTTATTTTC

>V300080312L3C001R0120631647

GACGGCCCTTGTGTAGTGAATAAGCATACTTGAAATATTCGTA

>V300080312L3C001R0301044083

AAAGCCGGCGAGGCTTTAAGGCCCAGGATCATCAGGGCAAACC

>V300080312L3C003R0250142764

ATCTTTGGTAATATCAGATACTGAAGATCGATCAATTTACACC

>V300080312L3C006R0680701051

TCGCAATTCTAATAAATAAATGCACATTTTATCTCAATACGTA

>V300080312L3C005R0160600199

CTGAACTTCTAGTCCTATGGATGGACGATTCATAACTACCCGT

>V300080312L3C001R0100233203

GCTCTTTTGATACAGCGCCATCCTTGGTCTTCTCGTCTTCTGC

>V300080312L3C004R0580419863

CTCAGTCAAAGGCTACGGTGAAGTCGAGTAAGTCTGAGCTACC

>V300080312L3C003R0630129040

TCGCTGCCGGTGAATCGCAAAATCCTCACAAAACTGTACCCGC

>V300080312L3C003R0020619210

TATTGCTGCTAGTTGCTTCGTCGGCTGCTTGGCATTCTTGACC

>V300080312L3C001R0500816145

GCTAGTTGTTTCGTTGGCTGCTTGGCATTCTTGACCAGCTTGT

>V300080312L3C005R0240330756

CGCTCATCATTTGTATATTTCTCTCACCTTCCCCTCCCTTTAG

>V300080312L3C005R0151011956

TATACCTCTTCAACAGAGATTCCCACATTCTCACTAATTTAAA

>V300080312L3C001R0081242226

CGGAGATGGTGCGGTTGGCAAGGTAAAGACAAACCTTACAGTT

>V300080312L3C005R0650491394

TCCGATAGTCGCTTTCCACGATCTTATCGCGGTCGTCAGGACA

>V300080312L3C004R0720352682

GTGAAGGACAATCGGGGCAGAAAGAGTGTTGGAGTTGCTGGTG

>V300080312L3C001R0610822793

AGCTCACATTTAAATACGCTTTAGAATAATCCGTTGTATATCA

>V300080312L3C002R0630204407

CAATGTAGATATTCAAAAAGATCCATAATGCATACTTGTCACA

>V300080312L3C004R0221402470

TGGTAGCTACTGATGAAAGTTATGATTTATATATTTCGAAATC

>V300080312L3C002R0640137316

AGCCTTTTCCAGTATTCTCCAACGATCAGATCAAAACGGTCTT

>V300080312L3C001R0480507706

TAGAACTTCCAGTGCAATCCAAGTCCTGGCGGAAATGGCTCAT

>V300080312L3C004R0581356634

GAAGATAAATTATGCAAAGCTTCTACTGAAGGTTTCGAAGACT

>V300080312L3C001R0530435972

CTGCGATACTTTGTTCCAGCAACTATCGGATGACCTTTACGAT

>V300080312L3C003R0550137087

TGTCTAATCGGGGAGGATGGTATCGATCCAGATAAAAACATTG

>V300080312L3C006R0460371366

GGATGGTATCGATCCAGATAAAAACATTGACGAAACCAAGAGC

>V300080312L3C006R0190516296

TAAAAACATTGACGAAACCAAGAGCAGTTCAGGGTAAGTGACA

>V300080312L3C005R0410155284

TTCGCAAGTAAGTGTCAAGAAAACCCGCTTTTAAATTATATGG

>V300080312L3C003R0180312483

TGCTAAAGGCTCACTGATGACCTCCATTCTCGAAGGACTACAA

>V300080312L3C006R0411365199

TGTAGTGGATCCGGAAGCGCAAGCTGATCGACTTAAAGGAGCA

>V300080312L3C006R0680620993

CGCTAAAGGATGGCTAGTATGGCCTAATAGCTACACATTCTAC

>V300080312L3C003R0581220044

ACACGATGGCGCGTAGCAGGCACTATTCCTTTCACCCGTTCTC

>V300080312L3C002R0431143928

CATGCAAGAAGTTCATGATGATCAAGATCTTCAGCAAATGAGC

>V300080312L3C001R0101231283

CCAAATTTACGGAAGAACAGCTATCTACATTGAATGACGTGCT

>V300080312L3C004R0480194725

AACGTAAAGTGGCATTGTTTTGAGATATAACAAATCTATTTTC

>V300080312L3C003R0580919766

TGACGTTTGCAGATATTTTCAGAAGCCGTCCTGGTGAAGGGTA

>V300080312L3C006R0540113635

GCGATGATGGTGAGCTGCACCTCTAAATCCTTTATGTGGGGCA

>V300080312L3C001R0470457945

AAGCGTGGCGCGACAGACACACGGGCGTTGACGTTAAGATGAG

>V300080312L3C003R0581094692

GCACTTTATAAAAGGTTCTAGTAAGCAGTGGTCAACGAACTTC

>V300080312L3C003R0480593331

CATGTTCTTCTGTATAGAATCAAATCAAAAATCAAATGAAGCA

>V300080312L3C004R0391290018

ACGGAACAACCGCAAAAAAGTGACATGCATTCACAAGGCTAAT

>V300080312L3C005R0190810824

CTGAAAGGATAACTGTAAACTCGACCACAATCCGGGTACGTAC

>V300080312L3C002R0721333156

AGTTGATGAGTGATGGCTAAGAAAACGTAGAAGCAGAAGGAGG

>V300080312L3C001R0020687668

AGTGCCCTTTTTTCCTTCACGTGCTTAAAACGATGATGAACAA

>V300080312L3C001R0011401382

TGCTGCCTGAAGCTACACATGTCCAGCAAGTATACGATGCCAT

>V300080312L3C003R0390562818

TGGATTCACACACCAACCCCGTCCTGGGAACCACGGCTGCACA

>V300080312L3C005R0351034600

TGTCCAAGGGACTCGTAATCGTATTCTTCGGTCATTGTGAGCG

>V300080312L3C001R0701002073

GGCTGAAAACCAATAGTAGTGTGAATATGCGCGCGCTTTACCG

>V300080312L3C006R0711211934

TTATCTTGCGCAATACGAGTTCAGGGCAAGCTATTGAGTCTTC

>V300080312L3C001R0031042010

TACCCTATGAAAACCGGCAATATCTCACATGAGGCAACGTTTC

>V300080312L3C001R0110315602

GATGGCACCCAGCAGAGCCTGCGCAAACACGACGCCGTGGATA

>V300080312L3C001R0010084404

CCTACACTCACAAACATGATGGAGAATGCTGCCTCTGCTGGCA

>V300080312L3C002R0150011262

TTCATCACGTCGCCAAGATTCGCCTTCTGGCGGCATCTTGCGA

>V300080312L3C003R0320017110

TCACGAACTGCCGCGAGCGCGCGCACGCATCCGGGTTACCCTG

>V300080312L3C006R0550561563

ACTAGTCTTTTGCGTATGGGATTTACTTCGGACAGATGGATAG

>V300080312L3C002R0520073482

ATCCTTCCGTTGTTCAAACGCTGCATAAGATACGATTTAATTG

>V300080312L3C004R0580359211

GATCAAGAATAGAAACCACCGCGTGTGAAAACCCGCTGTACAA

>V300080312L3C005R0600675643

GTGTTGAGAGTATGAGACCAGCTTTAATACACGATGCCCTGTT

>V300080312L3C005R0270199639

GGGCGTAGGTTTTGTGCTTGTAGTTGTTGTAGTAGTAATAGTA

>V300080312L3C001R0160195104

AATAAAACTCTGCAGTGGTTAAGGTTTTGTTTTGTGTTCCCTT

>V300080312L3C004R0720350285

ATTCGGGAGCAGCGTAATAAGATCTGAAGCTGCCATTGATCTG

>V300080312L3C002R0410696430

AGTGTTAATAATGGTTCCCCCGGCCTTGAGATGGTTACACACG

>V300080312L3C002R0590746731

CAGCAGCAAGCACCCAGCACACTGCCGCATTTGCCCAAGGGCA

>V300080312L3C003R0320272771

AACGATAATCCGCTTCCAATGCCCGGCAAGCACTTGCAGGATG

>V300080312L3C004R0280978845

CTGCAAGTCGACCAAGGTTATTGCAAACCCCTTCCACGAATCA

>V300080312L3C006R0471006502

CAAGATTTCCGCCTCAGCAGCAAGATGGAGTCTCACAGTGCTC

>V300080312L3C004R0020432594

AACATTTAATTAACTATATTGTAGCCGGCTTCTTGCATGGATA

>V300080312L3C005R0111086536

CAGCAAGAACAGCCGCAGATCTTGCAGCAACAGCAACAGCAGG

>V300080312L3C001R0281286972

AGCCACGAGGGTGAGCGAGGTGGGTATCGGTGTGCCTTCAAAG

>V300080312L3C001R0230032061

TCGAAAAGTGACCCGGTCGCGAGTCCTTTTCCTTTTCTTTGAC

>V300080312L3C003R0690907807

TGGTCAGATCCTTGTCGCCAAGGCGCAGACCTTTGGAATTGCA

>V300080312L3C004R0060371208

CAAACACCGCATTTCAAGGCTTCAGTCCTTAGCTTCCTCAGGT

>V300080312L3C003R0631061055

TCCTCAGGTATTCGAGTGATCAAGGTCGTCCTTAGAAATCTAA

>V300080312L3C002R0070451387

GACCAGGTTGCTTCTGCGGGTTCCGGCTAGCAGTGCTCAATTT

>V300080312L3C005R0111318483

CGGCTAGCAGTGCTCAATTTCCTTCAGCTTTTTCGTCCCACTT

>V300080312L3C004R0100457293

ACTTGTGAGGAGCAGGAGCAGAAACCGGCAAAGAAGGCTGCTA

>V300080312L3C003R0240238339

AAAAGACCTTATACACGTTCTAGCGATCGGATATGTGCAAGGG

>V300080312L3C005R0420290929

TAACGGTCTTAGGTCAAGGTAAAGGATTTCTTCAATCATTTCA

>V300080312L3C001R0500366309

TCACTCAACGATAAATCGCCGAATGACTTGTTAAGCGGCGCGC

>V300080312L3C002R0710574795

GAGAAGGCGTTTTTGATTCCTCGGATCTTCTGGCAGAGGGGGA

>V300080312L3C003R0160592369

CCTGTTCAAAGTAAAATCTTGTTGCCTTTCTTGATGTTACTCA

>V300080312L3C001R0490382412

TCTGCAGATCCTCTGGTATCCGATCATCCAAAAACACTTTGGT

>V300080312L3C005R0311258257

ATGCATGCGACTAGCCCCATCGTTTGAAGGCTAAGACAAATCG

>V300080312L3C005R0580407229

GTGTTATGTATGTAGCGCATGAAATAATCCGTCCATGTTGGGA

>V300080312L3C005R0400505119

TATCTTGTCGCTCTCGTTGCCAGATATTATGCAGCCAGGTAAT

>V300080312L3C002R0450705560

TTCAGATAAATGCATTTTTAAACATGGAAACCAAAATGTATGA

>V300080312L3C004R0450409509

CCTGGCACAGATCAAAAAATATACTTACGACTTTGACGGTATC

>V300080312L3C001R0490413404

CAAAGCCTGCGAAGCACTGTAGACTTGAGGTTACTTTTTGAAA

>V300080312L3C003R0671380586

ATCTTCGTCTCCGAAATATACCATCAGCGGCGGGGCCAATTGA

>V300080312L3C003R0640320242

GTAATATTTGCAAAATGGACGCTACCTGATGTTCCAAGCCGGA

>V300080312L3C002R0200082005

AAACTAAGAATGAATGTTTTGCGGATAGACATACCTGTGGATC

>V300080312L3C002R0570480982

ACTCTATTGCAGAAACAATCGACGCTTTTCCCATTATCCCGTT

>V300080312L3C001R0470363540

CAAGGCCGACGCGTCGGCTTTTGCAGCGAATACGATGCATTGC

>V300080312L3C005R0011009480

TTTGCAGCGAATACGATGCATTGCCTGGGTAAATCGAAAACCA

>V300080312L3C001R0061122391

TCTGGAAGAAAAGGGCTTCAAAATGACGCGCGGCGTCGCAGGA

>V300080312L3C006R0480309564

ACATAACATGAAAACAGTGTAGGGCATGCATGCGGGCATAACC

>V300080312L3C003R0660012279

CGCCCTTCTACTCGTGCTAGACTGGCGCAACTCAAAGTTCTGT

>V300080312L3C002R0630448057

AGAGCGCCTGGGCTGCCTTCAAGAAAGGCACAGCTACTGCATA

>V300080312L3C001R0321395322

GCGAAACGTATAGATGACGCGAAACCAAAAGTGCTCGTAACTG

>V300080312L3C006R0560986097

CATATCATGGCACGGACGGATGATATTATAAACGTTGCTGGCA

>V300080312L3C005R0530585203

GCGGCGTCGTATAATTGCTGCATGTGGGAGGGTGGGACTATCT

>V300080312L3C001R0380400835

TTTCCATACTGGGCCATCGGTTCCAAAGAAAGTATTTAGCGAT

>V300080312L3C003R0350387048

TACACCAACGACGGTAAGATCCGGGACTGCCTGTATCATGGTC

>V300080312L3C006R0100487168

CGTAGCCCCGAGGGCACCTTGTCACTGGTAAACTCTTCGAAAC

>V300080312L3C002R0080681077

CACGGGTATGCTTGTAACTAATTTTCTCTCCCTAAGAGATCGT

>V300080312L3C004R0450545845

GGTGGCTTCTGGTTGTTCTTGATTGTCAACAGCTTCCGAGGTG

>V300080312L3C001R0510423643

TCCCTGAAGATGTCAAAGCAAAGGACCGTCGTTGCATTTTTAA

>V300080312L3C001R0330439008

AACGGCAGCACCCTAAGAAGAAAACGGATAGATGCTGAGCTTC

>V300080312L3C002R0200970314

GGAAAGCTAATTTTAAGGGTCTCAATTGTTTAATGTGGTTTCG

>V300080312L3C006R0350566787

CCACCGTCCGGCAGAAGCGCTTTGGCGAATGCTTGGTGATCAA

>V300080312L3C003R0620899125

ACAGGATTAGTCTATGTCCCTTCTCATTCCATTTCCAACAGCA

>V300080312L3C001R0590092242

AATATTGCCAAGGGGCCGGGAGCATTCTTTGTAGAGGGTGACA

>V300080312L3C001R0081040278

GGTATGCCCAACGTTACCCAAGTCGCTATTCAGCCTGGAGAGG

>V300080312L3C006R0110353725

GAACAACTCGACTATGTCTTTGGGCCCAAATATCGAGGAATGG

>V300080312L3C006R0361356591

CCACGATCTGTCTGCTTTCCTGCTTGCGCGGAAGCATTCACGT

>V300080312L3C002R0140244949

CTTCTCCTACTTGCTCATTCACAATATCGTTGGCTCCATTGCT

>V300080312L3C006R0360361868

CTACACATGTTAGTGAAAGTCGTTGCAAGACCAGGATCCAACA

>V300080312L3C005R0510592538

CAAAAGCCATGGCCGCAGCCTAAAATCCGCACTTCGGTGCTAT

>V300080312L3C005R0250893884

CATTCGAACTTGAAAGGGCTAACAAGAGGAAAAATGTTCGGCA

>V300080312L3C006R0390866286

ATCCTTCTGCGTCCAATCGTCTTATTTTAAGTAACCGCGAAGC

>V300080312L3C001R0531005888

GGGAGAACTGTAGGCTCATGTTCGCTTGTCCGATGGGCTAATC

>V300080312L3C005R0211061652

TTTGTTGTACATGATGAAAAAAGATGGAATTTGATCTACTCTA

>V300080312L3C002R0611334159

ATAGTAAGTGCATTAGGCACTATCTAACTTTTGCTTCTCGATA

>V300080312L3C003R0710918277

TCGTTTCTTGTTACGAGTTCTTGGATGAACTTATTAAGAGCCA

>V300080312L3C004R0400644316

GAGAGTAAAGGATTTATTGATTCAATCGGTTGTGCTATATGTG

>V300080312L3C001R0660750880

ATCTGTCTATCGAAGTTTCTGGTAAGGACTTGTCTGTTATGTG

>V300080312L3C004R0341114070

AATAGGGAGATGTCTAAGAAAACAAGGAATTTATAAACAGGGA

>V300080312L3C005R0150891922

CACTACGCGAAAACCACAGCTTTGAGCATCCCTCCGTTTTATA

>V300080312L3C003R0450016875

GAAGTAAGACTTGCAGCCATTCAGAATTGCAACGAATATGGCA

>V300080312L3C006R0560665528

TAATTATATCTGTAGTAGAAACGCTGCTAATATCTGGTTTTAG

>V300080312L3C005R0020736575

CAACGTCTACCAGTGCTTACTTGACAAGACGAAAACGATAATT

>V300080312L3C001R0191043790

GTCTATAGGACAGTTTATTCGGAAACCTGGCATACTTGGAGAG

>V300080312L3C003R0201237034

GCCATTTTTGAATCTTTTGCAGCCTAGAAAGAGAAGCAGTCAG

>V300080312L3C002R0600822310

ATTGCTTCATCCAAAGCTTTGTCGAGTTTAGATGTCATGCTTG

>V300080312L3C005R0401122859

TTTACAAAATTGCTTGCCCGCGCCCGCTGAGTTTCTTTTTTTG

>V300080312L3C003R0090208572

CAGTGGGGTGGGAAGAGCGTGTTCGGAACCTTATCATCACTGG

>V300080312L3C002R0220960675

ACCTGGGATCCATGTCTACCTGACACAAGCCTGTTGCAGTTGC

>V300080312L3C003R0101109264

GAATTCGATGAACCTACCGAAATCGAGCTTGCTGCTCGTTTCA

>V300080312L3C004R0320570959

CGCGCGCCCAGCAAGTGTTCCCTCCCACCACTCTGAACCCTAC

>V300080312L3C005R0420318097

TCAAGTCCTCAGCAATCTTGACGCAACCAACGCTCACCTTGTG

>V300080312L3C002R0030401246

CTCAACTCGACAATCCATTTGACTTTGTCCCGGAGTTCTTGCA

>V300080312L3C006R0560368767

CTGAAGACGTGCTTCACTTTGTCACTCTCTGCCAACGCCGTGG

>V300080312L3C006R0011311929

GTTCTTGTACCGTGGCCAATTCAACGATTACGAACACACATTC

>V300080312L3C002R0150080270

GACTTGGCTAACTTGCCTGGCACTATCACTCACGGCATGTGGA

>V300080312L3C004R0241346106

CAACTTCAACGTCGAAAACTGGCAATACGTTGCGGCCGGTTCT

>V300080312L3C005R0710179374

TCGCCTGTCCGCTGGATCGAAACTCAAGACCAGCTGTTCAAGA

>V300080312L3C006R0421348212

CATCGAATTGGGTCCTTCGCCTACTCTCTGCGGCATGGCCACT

>V300080312L3C001R0551294847

CATCCAAGCATCTTTGAGCTCTGCCAGAACGAACCTGTCTGGG

>V300080312L3C002R0390743732

GCCACCGACAAGGAAGGTCGCTCTGTTCCTGCACCTGGTGCCG

>V300080312L3C001R0700505294

AAGGCTCGCCGTGCTCAAATCAAGAGCTGGCTGGAGACTGAAT

>V300080312L3C002R0300551987

GAAGGGTGTTGGTGTCGACATTGAATTGGTCTCTGCCGTGAAC

>V300080312L3C002R0690576593

TATCTCTTCTTACGGCAAAATCGCTTCTGAAGGCGCTGCTGCC

>V300080312L3C004R0040125241

TCTGCAAATTGATGAAAAGTCCTGCAAAGCAAAAGCACCAGAG

>V300080312L3C005R0261071835

GCGGATATGCTCGTGTTTGATGGTAGTCGTTCTTACGACGGCG

>V300080312L3C002R0170727267

TGTATTGGATGATGATCGTTCAATCAAGTCTACGAGCGAAACG

>V300080312L3C002R0710423331

AACATCACATTACCTTCTTCTCCATGTTTAATTTTTTTGTTGC

>V300080312L3C005R0570568503

TAGAACCTGGAGAATTCATAATTCGAAATAATATCTTCTATAA

>V300080312L3C003R0441318513

AGGACGGTACTGGACGTGACCTTGTGGTGGTGGAGGGGCAGCA

>V300080312L3C004R0671072415

CAACAATGTTGAACAAAGACACACTAGAGATCGCACAAACTGG

>V300080312L3C004R0070629651

CAGTTAGTATGGGAATGAAAAGGCACACAGAATACGAAAAACC

>V300080312L3C001R0041200470

CCAGATTGAGAGCGATTATTCAAAATATTTGGCTGTATGAAAA

>V300080312L3C005R0060171311

TACAAATTCGAGTCTATTTTGTGCAATACCGTTTTACCCCATT

>V300080312L3C004R0570719031

CTGTTTTCTCATCGACGATATATCCGTCAGTTCCTCACGAATA

>V300080312L3C005R0100727310

TTCATAACCTCTAGGCTAGGCTCAATTTGGCGGCTCTTGGATC

>V300080312L3C003R0390829511

GTTTTCGCTGCGCCAAATGTCACAAATTAGTCGAGTGCTACAG

>V300080312L3C003R0630839785

CCTTCGTGACGGCAGCCCTATCTGTGAAGATTGCTCATACGTC

>V300080312L3C001R0090614334

TTGCCAAAGCTAGCTACGCACGGCTTGTCAAAGCAAGAGACGA

>V300080312L3C001R0190148624

TGCGAGATCTGTGCCGATAAGATTTGGCGTGCGAGCGAACTCA

>V300080312L3C002R0150589842

ATCTGTGCCGATAAGATTTGGCGTGCGAGCGAACTCAAATGTC

>V300080312L3C005R0651006121

TGTGGATATGTTTGTCACTTTAAATGCATGCACAATGTATCGG

>V300080312L3C003R0431065794

CAGGCGCCCAAAAGTAAGCAAACTTGAACCTTTCCGTTTGCAA

>V300080312L3C004R0400447719

GAAATGTATTGAAGCTGTAGAAGCTCGAGGTGAGAATCAGATA

>V300080312L3C002R0230030398

GCAGCTTACCAGAACGACGTAACCAAGGAAAACGGTGGTGGCC

>V300080312L3C002R0530432008

CCGCAAGCTGGAACTGCGCACGAGACAGCTTACGATTTCTTTG

>V300080312L3C003R0380065912

GGTTACTATGGTGTCCGTCTCATGCGTGCTAATGCCTAGTTGC

>V300080312L3C005R0150658744

TCAACCGTCCACTTTGTGCTGTTCGAAACAATCAGGTATGTCC

>V300080312L3C002R0501333976

ATAGATATCCTGAATCTAACGTGGAGGTTGCAGCGTGACGGTT

>V300080312L3C003R0570878512

TAGGTCTCTCAATTGACGAACGCCCCAAGCCGAATCACATCCG

>V300080312L3C002R0140195665

TGACTTTTTGACCAAGGAGGGTGCCTATGTCGATTGCGTCGGT

>V300080312L3C006R0590300310

GTCAGAACCGACAACAATGACGTCACCTTTTTGTACTTTGATG

>V300080312L3C005R0400228319

GGAAGAGATTTCGGTTGATAAGATATTAACGGCGTTGGCGATC

>V300080312L3C002R0121218666

GCGAGCTTCGAGACGCGGTTGATCAAAAATCTGATTTCTGCAG

>V300080312L3C006R0680204572

TCGTGCACGGAGGGCCCGGCTATTTGCACGCCCCCTCCCACCA

>V300080312L3C003R0590208475

CCGTCAAGCGGATATTCAAAGACCTACAGCGTTTTCAAAAATT

>V300080312L3C005R0091342551

TTTAAATTTGTCTTGATTGTCGTGCAATTTGATGCTCCAAACG

>V300080312L3C005R0241111904

CCCGAAGTTTCAAAACTGCCTAATCAACCGGACGTGAGCAGTC

>V300080312L3C001R0320347195

TGATCATTGATGCACTTCTTACCACGTCCTTCATGCATTGCGA

>V300080312L3C004R0110246262

GTTTCCGTAATCAGCAATATTCGTCGACGTAAGAATCTTAACA

>V300080312L3C001R0160340425

CCAACCCTTGAGGGACGACGTATTCCGTCTCTGTTGCTGCCGA

>V300080312L3C003R0190619665

TGCTGCCGATGTTCCGTCATGTGTTCCCGGAATCTCACTTTGC

>V300080312L3C004R0180525925

GCCACAAACGTTTCACATTTTTACCTATTTCTATTCATTCATA

>V300080312L3C001R0070334938

GCAACCGATAGCAACAGTAAGTATTAGGGGGATACAGATTCAA

>V300080312L3C005R0700176938

ATCTCTCTCCTTTCTGAGAGTGTCAAGCAAATTGAATATTGGG

>V300080312L3C003R0690789478

TCCAAGTAAATCAAGTGCTTTGTCATCAAGACCTTATTCTTTC

>V300080312L3C004R0120150920

TTGTAATGGAAGCTTCTTGCCGATCTTAATGATGCTTGGCTTC

>V300080312L3C003R0540807211

TCTTTGTATTCCGGATACAGGAAGAAGACTGGCCATGACAATG

>V300080312L3C001R0360829418

TCTTAGGATCGAGCTGGATATTGGCTTTCTCGCGTACATCCTT

>V300080312L3C002R0641037278

CAGCGAGAACCGGTGCAGCCTAATAATATCACTGTGGAGGAGG

>V300080312L3C004R0040971898

ATTACGTGATAGACAACACGTGGAATAGCATTTCACATTAGAA

>V300080312L3C004R0280335149

TAACTGTAGTAAAATGCTGAAAAGCTCAATATAATGCTGTAAA

>V300080312L3C001R0280950285

AATTATCACTTTATACTCAAGAATATGGAGCCTTTTCGTGGTA

>V300080312L3C001R0450485050

GGTTTGAGATGTTCTATATTCTGATGTCATGACGACGACTGTG

>V300080312L3C002R0240240139

AAACCTTGGTCGCCGATGAACATAATGGGAATAGACTTCTTCC

>V300080312L3C005R0690249474

CCAAGCAGGCGGCGGACAATACATCTGAATATCCTGATACATT

>V300080312L3C003R0210154433

TGTGATGGCTTGACCCAGTATGAGTTGAGACACTGGATACCAG

>V300080312L3C001R0160008400

GGATACCAGAACGTTTGCGAAAAAATGTATGTGGAAATCGGGG

>V300080312L3C002R0700535744

CTTTGCCAAACTTTTGAATAATGCGCTCTTTAGGTTCGGCTGA

>V300080312L3C001R0121275089

CTTGTTAGTTTTGCGTTTGTGCCACTTTGACCACAGTCCCGGC

>V300080312L3C005R0430097026

TTCTGCGAGTTGTTCTTCGACTCTTTTAGCCTTCTTTAATGCT

>V300080312L3C003R0710325291

TATCATACTTCTATCAAATGCTTGAAGCTTATTTCTTCGTCTG

>V300080312L3C006R0620319639

CTGTACTGGGCTGTCGGTGATGGACAACAAACATCCTTCAACC

>V300080312L3C002R0361066550

TATTTTTCCAGTAGTTCCTTACACTAGGAGAAAAGCCCTCCAC

>V300080312L3C003R0281401150

GCCTTCATGATAATATACCCGCATAGCGATGGTGAATGGAACC

>V300080312L3C006R0651195942

AGCAATGCCGTCACCAAAATGTCCCAAATGTGAATAAATAGTT

>V300080312L3C005R0691184868

CAGCAGACAAAAATATGCGACGCGGTGTTACTGTGTCTTCCTT

>V300080312L3C001R0130626261

GTGCGAGTGCCGTCAAGGGCTTCAGACTTGCTGCATTCAGCTG

>V300080312L3C005R0591296303

GGTGCAGCAGTCAGTCGCGTTTTCTTCGCCCAACAAGACTGAT

>V300080312L3C002R0531150360

TGGCTCTATGCACTGCAAAGTGCAACAGGTGTTGAAACTAGGC

>V300080312L3C004R0610170108

GGCGTCTCGAGCTAGTTTCGGCGCCACAAAGAGAGCAACAACG

>V300080312L3C004R0470594796

ATTTTCGGTTTCCTTGGGAACGTATTGGGCGCAGTCCTTTATC

>V300080312L3C005R0010572939

ATGTCCAAGATCAAAAGCAAATCCGGTCGCCACGCGGAATTTT

>V300080312L3C006R0301280511

TTTTGATAACGGGGCAGCTATCAACGATGCCGTGATGCACGCT

>V300080312L3C003R0270070530

ACCAAGGCGAACTTATAACCTGCGATCAGAACGGTGCAGTTAA

>V300080312L3C002R0150886731

ATCGTTGTGTTGTGCGTGGGATGATTGTAATGTATTGCGTGAT

>V300080312L3C003R0520592787

CAATTCACTGTTGCTGTGATGTACTTTGATAAGAGCATCACTG

>V300080312L3C006R0190586855

GGAGGTGTGCCGTATCGTTATCTCGGAAGAGAGCGTTGTTGCA

>V300080312L3C003R0500051295

GGGGTATGCAAGACGAGAAAGAAAAAGAACAAGAGGAAAGAGG

>V300080312L3C001R0330097425

TCGATTTTTAACGTAAAAACTCCCAAAAACAAAAACCTTGCAA

>V300080312L3C002R0530957555

AAAGTATACATATGCAATAATTGACAGCAGCAAAGGTCCCACA

>V300080312L3C005R0310197578

CCAATATTGTCATATATTGATAGAGATATTCACCGCGTCATTA

>V300080312L3C002R0670374031

TGCTCACTGTTTGCTGCTGTTGAAGATGGCTTGTGCTCTTCGC

>V300080312L3C001R0140555605

CCCGATCGAAGTCAGATCTCCAGACAGGCAAGTTGGCCAGGTG

>V300080312L3C001R0400612100

TGCCACTTTCACTTACGATTTTGCGAGCATCCGGGGGCGCATG

>V300080312L3C006R0501197676

AACCACTGAGAAGGCGAGTCTATTACTTCATTAGTACTCATGT

>V300080312L3C006R0380291533

ATGCCAATATCATAAATATCTTCGCCGATCTTTTGAATGGAGA

>V300080312L3C005R0710712370

CCTGATTCTGAGCGCGCTCCAAAATGGCCACAACGACACCGCA

>V300080312L3C001R0670246734

AACATGCCGCGCACAAGCTGGCGGGAACGGAACAACGGCGTCA

>V300080312L3C003R0650504272

GCGAAGGTGCAGCGCTTCCACCTGTAATTAAATGAATAATTTA

>V300080312L3C005R0301226772

CTTCTGCAATGATATCTTTGCTGGGACCGCGGAGAATGATGGA

>V300080312L3C003R0540932370

CAGTCAGTAAAAATTCCAGGTATTACAGGCTTGTATCCGTTTT

>V300080312L3C003R0670120743

GAAAGGATGTCACTGTAACCTGTGACATTCAATGGATATCGAA

>V300080312L3C005R0510934741

TGGCCCTCAGCATCACCTGGCCGAGCCAAGAAGACTTCTGGGT

>V300080312L3C005R0710443841

AGCTAGCAAAAGATACAGTCCGTGTGAAACGCTCATGATCTTT

>V300080312L3C003R0690105864

AAACCCTACGCAAAGTAATGTTTAGAGAGATAGAATCGAAAGG

>V300080312L3C003R0490254678

GCTTCACTTTCTTTCCTTCGACATTTTTTCCCATTCAAGATCA

>V300080312L3C003R0330063231

ATCCTCGAGCAAGCGGTCGTGCAATTAATGCCCTTGGCCCCAC

>V300080312L3C005R0261207685

TATAGGATACCATGCCAGCCAATATCCGCGGTCCGTAGCACCT

>V300080312L3C003R0220777821

CAACAGCAAAGGACAAAATGCCGCTGATTGCGTTTGGAGACGG

>V300080312L3C002R0601031908

TTGCACCTCCCAAAGCCCACGAAGTTCGTATTAAGATTTTGTA

>V300080312L3C001R0300185273

CTGTTCTACCTTCTCCGAGTATACCGGTATGTGTATAATTTTA

>V300080312L3C002R0501158882

GTGAAACAAAGCTATCCTGACAATTACGTCGATAGTACATTCT

>V300080312L3C004R0690297287

GTGATTCCTGCCATGCTTTGTCGAGCGTCGCCTAATGACTATT

>V300080312L3C001R0670394294

TCTTCCTCTTGTTCCTGGTAGACTTTGATCTGATTTTTGAATG

>V300080312L3C006R0480863155

CGGTCACGAGCTACCCGAGAGGTCCTACGCAGAACAATGCGAT

>V300080312L3C004R0290607669

CGAGAGGTCCTACGCAGAACAATGCGATCTGACATGGGATACT

>V300080312L3C001R0010946164

CCTGTCTAATTCTCGATCCTGTGATCTCCACTTTAGACCCTCC

>V300080312L3C001R0551327239

TCTGATAGCCAATGTGCAGTAGCAAACAGCCAACGAGACTAGG

>V300080312L3C003R0700553388

GTTGATGCTTACTGCGAACTTGGATTTTTCGAGCTCATTACGG

>V300080312L3C003R0211299743

TCTCTTGCTGTTGTTTGCATTGATCTTCAAATTCTGCCAATTT

>V300080312L3C003R0250604027

GCGCTCAAGTCGCTGGATATGTTCTTGTTGCGCCCCCTTTTCT

>V300080312L3C005R0201245303

AATAAATATGTCCCTTTGCATCTTCTCGTATATAAATAGGTGG

>V300080312L3C006R0490853858

TCGTGGTTGTGCTCGGTCTCGCTCGGTCAATGGCCGAATACGG

>V300080312L3C005R0350298157

GACTGTGCATTCTTCAATTAGCGTCATGCCTACTCGTTTCTCG

>V300080312L3C005R0370693683

CAATGCTTGCTTACACAAACCCGGCAGCCAGCAAAAATGATGT

>V300080312L3C002R0220456592

CAGAAACCAAAGGAAGAAAAAGGCTATTGGGCTAATTAAAGCC

>V300080312L3C004R0250230779

GAAACCAAAGGAAGAAAAAGGCTATTGGGCTAATTAAAGCCAA

>V300080312L3C004R0360463612

AGCCAACCCCAGCAAAAATGATTCGCGTCACGGGTGCGCTGAA

>V300080312L3C004R0330880282

ACGTTATCGACTCGAGTTGCAATTTCTCGTTATTTCCGGAATA

>V300080312L3C001R0190651678

AAAAGAAAGGAGGTACATCCGTCGCTGATTATTTTGGATTGCC

>V300080312L3C001R0080192135

TAGTCGGCTATCTGCAATTCAAGCGAGAGTTCGCAGATGGTCG

>V300080312L3C004R0190109367

TATCTTATCGATTTACACACTGCAAGCTTTGGAAGAGTTAACT

>V300080312L3C006R0471337715

AATGAGGCTCACCGGTGTTGTTAGAATTCGAGGCAGTTTGGCG

>V300080312L3C006R0430743862

GACAGTCGTTTACGAGCGCGGTGCTTCTGTAATTTCCAAGGGT

>V300080312L3C003R0301121617

GTTCTGAATGGATTCGATTCATTTTCGTTTTTTGATATTTGGA

>V300080312L3C001R0400975113

ACACCTACAGGCAATCCACCAAAGCTCGACATACCAGAATTTA

>V300080312L3C006R0680804283

TCTCATCAGACTAGGTGTTGCAGAATTCTCATATGAGGAATTG

>V300080312L3C006R0720705061

TTTGCAACAGTAACCATGCAAAACCCGAGATAATAGTTTTCCA

>V300080312L3C006R0270398182

CTGTCGCTCTTGGTCCCTTCGCATTCGCAGTCTTTGAAGAAAG

>V300080312L3C001R0030616747

TAAAAACATAGTAATCATACTAAACATAGTGGGTTGGGCTGGT

>V300080312L3C006R0210028565

GTTTTCTGGCTGCTGCAGGTGCAGGAACGAACTTGACGGGCAG

>V300080312L3C005R0421034212

TCATCGCCGGCGAGAAAAACACATCCTTGACCAGATCCCTTTG

>V300080312L3C004R0180712876

TGCACTTTCCACACTGTTCAGTTTTGATCTACTTACTGCTGTT

>V300080312L3C002R0381375663

TGTAAGATGGCGGAACTTGGCAGTATGCTTGAGGATAGTAACC

>V300080312L3C006R0410056078

GACAAGAACGATGAGTGTGTTCATGCTCTGAGCGACGACTACG

>V300080312L3C003R0660026611

TCTGAGCGACGACTACGACGGCGAAGACGAGATTAGCGCGGCG

>V300080312L3C005R0520851849

AGATGACTGTAAAGCTAAATGTGTAGCTTAGAACTTCTTTGCA

>V300080312L3C004R0241106967

GCTGGAGCATCGGTGTTGATACGGAGCGGGACGGCGATTGCGG

>V300080312L3C004R0660241091

TGGTGGAGATGATGTTGTGCTGCTCAACGCTGTTGGTACGGGA

>V300080312L3C005R0250866808

GAGCTGAAAGATACAGAGTCGCGGTTGTTGTTGTGCATATGCG

>V300080312L3C003R0381201910

ACACAGCCGCTCTAGATCCTCAGTGCTATCTTGACAGGATTGA

>V300080312L3C005R0720284610

GATTGAATTTGGAGAGATTTTTGAGCGTGCGATGCTACAGCTT

>V300080312L3C003R0421223954

TTGTTGCTCGAGAGCGTTGCTCAGCTGTTTCTGGAGCTCTGCA

>V300080312L3C002R0231258100

GCGTCTTTTCCAGTTCAGCAATTTTTTCTGCTTGGACTTGTGT

>V300080312L3C001R0090179497

GCACTTGTCAGTTTAGTCTGCAAGTCGCTGACGGAGCAGCTGA

>V300080312L3C002R0111136192

GATGCTCTGCTCCAGGGCAGCCATCTGAGAAGTTTTGCTTTGC

>V300080312L3C005R0130534938

TGACTCGATTTCTTTGCGCGACTCTGCTTCGGTGCTCGCGAGA

>V300080312L3C001R0410525743

TGCGCGACTCTGCTTCGGTGCTCGCGAGACGCTCCTCAAGGGC

>V300080312L3C005R0310855901

CGACGAAGGCGATATTGTAGGTACATATTGGCTCAAGTTCTTG

>V300080312L3C004R0150196096

CGTCGTTGTTGGGGCCGATCCGCGTCGTTCGGTGTTACCATCA

>V300080312L3C004R0640295059

TGTTCTCGAATGTTTTGATCACATCAGACTCGGCATTCTGTGA

>V300080312L3C005R0550609558

TGTTGATCTGCTGATGCTGCTGGTGCTGTCGTTGCGTCTGCCT

>V300080312L3C001R0021066151

ACAAACAATGATGAGCAATTTTTCATATTATTCACATGCGAGT

>V300080312L3C001R0320025786

CGTCATTTTTCTGGGGCCCTTGAAACCAAGAATATTTGGGTCC

>V300080312L3C002R0411079754

CATTCGACTCCTTTTTCATAGGGTTCCTGCCATGCGAGTATAT

>V300080312L3C006R0701015737

ATTTGTCTATCTAACTTGTCTATCTGATGATGACCTACGCTAA

>V300080312L3C006R0440193062

ACGCTAAAAGAACGGAACGGGAGACTGTCTTGTTGCCTTGACG

>V300080312L3C001R0261177499

TCAATAATGCCGACATATTGTGCGACACTTCACGGACATCTGG

>V300080312L3C001R0610728458

TTCGTCGAAAAAAAGTAAAATAGCTGCATATAATATAAATATA

>V300080312L3C004R0340702862

CAAGTACCTGAGCACATTGTGCGCTTCCATTGAGCGATTCTGA

>V300080312L3C004R0540477401

AAAGAAGTAGCGGCTCCAGTTGTGGCTCCTCCCAAATTTAGCA

>V300080312L3C004R0431248204

AAGACTATTAAGATTTATGCAAACCGTCAAAACATTGGTTTCG

>V300080312L3C003R0501028531

CATCCTTGTAAATCGACAGCGAATGGGCACGAGTTGACAAACT

>V300080312L3C004R0371157827

TTGAAAAACACTAATGCGGCGGCGGTATCCATTTAGAAATGAA

>V300080312L3C004R0061220013

CTGATTCGTCTTGACCGCGCGGTCCTGTCAACTCCAGTAGTCG

>V300080312L3C006R0260692301

CCTGCAGCCATGCATCGCAGAATGACTCGAGCCCGGCTGTGCT

>V300080312L3C005R0310516304

CCAAGACGAGGCAGGAACTGCGAAAGCGCATTGTCAGGTTTTA

>V300080312L3C004R0571058978

TATGCGGGGTGGACTCACCTGTATGATGAAAAGTTATTTTGGG

>V300080312L3C001R0181179978

TATCCGGAACTTTAAAGAGACTGGAGCAGCGTACGTGCACAAC

>V300080312L3C005R0160938676

CATGGCCTATTCCAACCCGGAAGTCACACAGCAGGCTCATAAA

>V300080312L3C004R0020682146

GAGCGAAAGCAAACTACCGCGGACAGATTCAGAATATCAAGAA

>V300080312L3C002R0610857336

ATCTGAAAACCCCACATCAACACCCTCAAAGTGCGCGATAATG

>V300080312L3C003R0030683497

AACAGTGCCTCTGCTCCATTGCACCAAGAGGAGAAGCTCTGCT

>V300080312L3C002R0601066441

TATTCATGTTGAAAACAAATATATCCTGACAATACTCATGAAG

>V300080312L3C002R0641273062

CCACCCCTCTCTTTTTCTCTCTCATCTTGCATCTCACATTCTG

>V300080312L3C004R0470230998

CTCTGCATCGCCTGATCCTATGGTTGATGACGACCATCCTTTG

>V300080312L3C005R0311234440

GAGATACTAGAAGCAGCAGCTGTTCTTCTGTCTCTACAACGTC

>V300080312L3C002R0361014395

CCGTTCCGGTTTACGAATGACACCTATGACTGACAGCAGCAGC

>V300080312L3C001R0470622529

TCGTGATATCAAGCCCGAGAATCTGTGCCTGACGACCGGCGGC

>V300080312L3C003R0590413905

TCCAGCCGGCGCAAAGCCAGGCTCGCCCGCTGAAGTGGCTGCC

>V300080312L3C001R0471025059

GGCGCAAAGCCAGGCTCGCCCGCTGAAGTGGCTGCCCGCGCAG

>V300080312L3C001R0360190855

CACTGCCTTCGCGCCCACAAATAGACTCAACACCACCCAACGA

>V300080312L3C004R0330035467

TGCTTGTGCATGTTGGATTATCGATGGTATCTGGGTCCCGTAC

>V300080312L3C005R0421131804

ATTATGGACGCAAATTCGCAGACTGGGAACAACGAGCCATTTT

>V300080312L3C002R0190345004

GGCATATGTACGGATATGAACAAGAGAATCATACAAGGAAATA

>V300080312L3C004R0111245175

AGATGCAAAAGGAAAGGGAAGGAAACAACAGAACGAGTTGGTG

>V300080312L3C001R0621205193

TCATCATCGCTATCCGTCGAGTACCTTCTGGAGCTTTCAATGT

>V300080312L3C002R0471048853

GTCAGATGACTGGCGCAATGTCATTGACGGCTACGAAACGGGC

>V300080312L3C001R0320347996

GGGCCAAAAATTGCTGCTTCTCCACAAACGACCTGCATGAGCG

>V300080312L3C003R0410356771

CATACCGCTGCATGATACGTGAAAAGTCAAACCAAACCATCAT

>V300080312L3C005R0691058938

CACCTCGGAAATATCGAAATCGGCGGTCGCAACGACGCAATGC

>V300080312L3C001R0120403750

GACGAGCACTTGAATCTCTTGCAAAACTCGGAATTCAGTTTCC

>V300080312L3C004R0160330110

GCTCAGGATACCCATCCCGATGGACTTTTGAAGAGTTTGCTGA

>V300080312L3C002R0101390940

ATATAGCCATAAAGAAAGGAGAACAATATCTGAATATTACGAT

>V300080312L3C005R0070596881

TTTTGAAAGGTCTAATTTTGTCACAAACACCGACGACTGAAGC

>V300080312L3C006R0301315803

CGTTGGAGCCGCTGGTCTCGTAGGCTACGGTATCGGTGAATTC

>V300080312L3C005R0540492100

AGCTAATATCTATCACAGCTACTGCTGCTTAAAAGATGGGTGT

>V300080312L3C006R0051353278

GGCGCCTTCTGGCCCAACGCAAACGACCATGTGCCGCTTGGAA

>V300080312L3C003R0450986896

CCTGGAAACTTGCTGCTTTGCTGCTTGACAGTCGCACGGCCTG

>V300080312L3C004R0410823801

CCGCAGAAGATCTGCTGAAGAGTCGAGTTGGCAGGAGGTGACG

>V300080312L3C005R0320341453

ACAGGGAAAGGGACAGCAGGCAACCCACTTTGAGCTTTTATGC

>V300080312L3C003R0340615659

GACAGCAGGCAACCCACTTTGAGCTTTTATGCTTTTTTTCAGT

>V300080312L3C002R0700302176

GTATAACGATATTTTTGAATCGATCCGATCCCTCTTTTTTGCC

>V300080312L3C003R0190845652

CCTTGTATGATTTCTTTGATTCAGCTAATATGAGATCATACAG

>V300080312L3C006R0430199955

TCATCATTTTCTTTGCGCGTGCTATACGTTTTTGCGACAACAG

>V300080312L3C004R0601152999

CGTCTTCCAAAATATTGTTTTCGCTTTGGTTGTTGGAATCATC

>V300080312L3C003R0270304452

GGCCTGCATCATTTTGCGTGCTTCCTCCAAAAGCGCACGATGG

>V300080312L3C004R0630290559

CTGTCGCGACTCACGCACAGGTGTTGGTGGTTCACTGCTGCTG

>V300080312L3C005R0500181307

ACTTAACTCTTTCTGGCAGGCACCATGAAGTCTTATCTTTCTC

>V300080312L3C001R0690525132

TTAATTTCTGTCATGCGCAGAGTCGCGGGGAGCGCTTTTCTTC

>V300080312L3C006R0590406109

AATTTCTGTCATGCGCAGAGTCGCGGGGAGCGCTTTTTCTTCT

>V300080312L3C004R0561015164

GTTGTGGCTGTTTTGGTCTCCAAAGGACTAGCAAGAAAAAATG

>V300080312L3C006R0671120478

ATTATACATACACATTCTCGTCAAGTCCCTGTCAACAGTCATC

>V300080312L3C004R0280289883

CTTGAGAAGTACTTCCAAATTGTCCAAAAGGATTGTTGCTTTG

>V300080312L3C006R0641014588

ACGGATACTGGTTTTGGCAACTGTAGCCGATGCAGCTACGGCA

>V300080312L3C004R0560263570

CAATGAATTGAAGCAAGTCTTGATATCACATCCACCAAACACG

>V300080312L3C004R0171233082

GCGGCTGAAGATACGTTGTTATACGCATTGCTGCGCATAGAAG

>V300080312L3C001R0210199637

AAGATAACATGGGCACGGCAGCATTGCACCAAGCAGTAGGAAA

>V300080312L3C002R0620718702

CGATTTGTACATTTGCCAGTAACGTTCATTTTCCATAGAGTTC

>V300080312L3C005R0700635433

CCGGAACCGATCTTGAGGTGCTTTTAAAAGATGACAAGCAGGA

>V300080312L3C003R0500987128

GATCAGATATGCATATGAAGATATCGCCATATATAGTTTTATA

>V300080312L3C001R0450341976

CTAACATGTGGCCACTTCATCTTGGATGGCTTCAAGAAAATGG

>V300080312L3C006R0330986966

GCAAGTGATGATAGTCAATATCTTGGTGGAACTGTTGCTTACT

>V300080312L3C002R0310288489

TGAAGAAATTCTAGATTTGTTGAATTGACATCCAAATGAGGAA

>V300080312L3C002R0320957174

ACGTATAGTGTCGAATGAATATCCATTGTTTCAAGCAATTGGA

>V300080312L3C001R0381139019

TCCTCCTTTGATAATCACATCAGTAGGATTGGGCAAACAGAGA

>V300080312L3C005R0620053981

ATTAAAATAAAAAAATTCATTGATCTGATCAACGAGAAACTAA

>V300080312L3C004R0620949491

TACCTCCGTCAAGATCCCAGCGTGCCCAAGCAAGAGAAGGATG

>V300080312L3C005R0510590329

ACTATTGTTCTTGGATGAACCTACCTCTGGTCTCGACGCTCAG

>V300080312L3C006R0510460639

GGTCTCGACGCTCAGAGCTCTTACAACATTGTCCGGTTTATTC

>V300080312L3C005R0570596981

TCGAAGGTCTCGCTGTCAACGAGATGTCCAGCTTGCCTGTTAT

>V300080312L3C002R0390372471

CTCTCATTATACAGACAACGGCGCTGTGCTCGTTTCTATTACT

>V300080312L3C002R0141153979

CACTTGACCGGTCTTGTACAAACAGAACGGCAGCGAGCATACG

>V300080312L3C005R0600056404

GGGAATCTGCATCGCTCTGTGCATAACATTTGCACTTCATTGC

>V300080312L3C006R0130692828

ATGATGTGCTCGTGTCCCATGCAATGGAATGGCCGAGCTTAAC

>V300080312L3C005R0071127804

GACAAATCTGCCGCGCGCACGCACGCTTTTAGATATGGACTTG

>V300080312L3C001R0701013519

TCAAGTCACATTATCAGTGCCAGTTTCGATAATAGAATTTGCC

>V300080312L3C001R0210952994

TTGCCATTGGTAAGTATACAAGTTGTATGGAATGAAAATGGGG

>V300080312L3C003R0451243984

AGCGACAGGAGCAGGAGACAAGGTCAGAAGAGAAAGATGAATG

>V300080312L3C006R0710458039

ACCGCACAACGAGGCGGTGCTTGCGACAGCAGGAAGCGATCGA

>V300080312L3C005R0321051684

ACTTTCTTCACAGACTTCGCTGTACTGCTCCATAGAGTGCCTC

>V300080312L3C002R0190022065

CCATGCTTTTAACGATTCCTTTGGTAGCTGTTGTGTCTCGACA

>V300080312L3C005R0230239728

CAACTTCTTCCTTGCACTGCTCTACACTCCCGAGAAATCAGAG

>V300080312L3C004R0521378520

CCCAGCGAACGGATAAATTGTTTAGAGTCCTCACAAACCCGAG

>V300080312L3C006R0430112151

GTTGCCGGTGGAGATGGAGTTTGTTCGCTGATTGTGGAAGCTG

>V300080312L3C003R0040148188

AGAGGAAATCACATAAACAGTGTAATTATGTGACGATCTTTAG

>V300080312L3C006R0100114738

TATGCAATAAGAAAAAAGAAAATCTTGTAGCCACAAATTCTGC

>V300080312L3C004R0090258320

ATGTTGCTTTGGTATCATTGTCATCCTTGGCCTGGCTGTGGCT

>V300080312L3C001R0271183294

TTCCAGTAGGAGGTGGCGATCTTTCTGGGTTGCACATTGGCTC

>V300080312L3C001R0631204450

CCAATTGAGGTAAGGCTTAATTTCGTCATTAAACGGCAAACGT

>V300080312L3C002R0560386830

TAAGGATTTCGCGCTTCGTACGTAACAACCGTACCTTTTAATC

>V300080312L3C004R0540790396

GTATCTTGTTGAGAATAAGTGTTGTCGAGTGAGCGGTTATTAT

>V300080312L3C005R0541038211

CCGCTTACTTCATTAATCTGTTTGCATAATATCCTCAATATGA

>V300080312L3C005R0621267341

TCAAAATCTCTGACTTCATCTGCAACGTTCAAGAGAGCAAAAC

>V300080312L3C003R0511324444

TTGCCTTGGCTGCATCACTCAGCGAGGCAGCTCCCGCTGTGCC

>V300080312L3C004R0051291501

TGAAGATATTCTTCCAGTCGAAAACAAAGCTCCCTCCGCGTTT

>V300080312L3C001R0120393698

CCTCCGGAGATGGAGGTGGCGGCCTACCCATTGTAGGTTCTCT

>V300080312L3C006R0031017289

GATGGAGGTGGCGGCCTACCCATTGTAGGTTCTCTTTTATGCA

>V300080312L3C005R0260695918

CTTTTGCCTAACCTTCCTTTGCCCCTCGATAAGCTTCCTCTTG

>V300080312L3C005R0500181893

CCAAGGATTCCAGATGGCGACGACGATATTTGCGAGCACGGTC

>V300080312L3C005R0090181988

TCCAGATGGCGACGACGATATTTGCGAGCATGGTCCATACGAA

>V300080312L3C001R0051291155

TGCGATGAAATCACCTTGAAGAAGCGTCAGGCACCTGCACCTC

>V300080312L3C004R0170045753

ACAACATTTCATATCCTTAGCAATCGTTTATTTGTTTATTTTG

>V300080312L3C005R0250415155

CATTTTCGCAGCTGTAAACGCAAGTTGCCAAGTAAGTAATACA

>V300080312L3C002R0480189556

AGCATGCAAATATATTTTGCATATAGCCGCCGAATATCTGGTA

>V300080312L3C006R0070530302

CGTATCGCGATTTTCGAAACTTGATCGCACGGTTGAGAAGCGA

>V300080312L3C002R0440185172

ACCACGGAGCTTGTTTCTATATAGTACGGTTATCAGTATACAA

>V300080312L3C005R0041281275

CTTTTCGGCTTCATCGGCGCAGACAACAGAGAAGGAACCGACA

>V300080312L3C004R0630817686

CCGGAAAATTTGCCGTTTTCGTCAAGGAAAACTTACCCATAGG

>V300080312L3C002R0200481488

CGTACACTATTATCATATCTACGTATACTTTTATGGCAAATTA

>V300080312L3C004R0380657627

GGTTTCCATGCTTCTCCGTCGTTGTCAACGTATCTATCTGACA

>V300080312L3C002R0621056776

TGAGCAAGAAATGATCGCGATGCTTGGTAACTCTCATCCTTTC

>V300080312L3C004R0661193360

ATTCGGTTCCTGTGCCAAAAGGCACGCGATATCTTCATGTCTC

>V300080312L3C003R0010394459

GAGGGCAGCCATGGTGCGCAAAACTTGAAGGATAGGCTTGTTC

>V300080312L3C005R0390949449

TTCAGGGTTCATAAAGTCGGCTGGAGAAGTGGCAGGGCACTTG

>V300080312L3C004R0100031334

CATGCTATAACCGTGGCCACCGCAGGCACGACGGCAGTCCTCG

>V300080312L3C003R0580267958

AGCCATGGTGGTGGTCAAGCTCTTGAGACCAGAGGAACTGGCG

>V300080312L3C003R0171385058

TGGTTCAAGTCGTACAGACGGAGCATTTCACGGCCAGTGAAGA

>V300080312L3C005R0440110047

AAGGAGGAGAGTGAGAGGGGGAAGAAAGGAAGGCCTGGAGAGA

>V300080312L3C005R0130557847

TCTCAAGCGGGTTTACATTCAGATCGAGAATTTGGCTTACGGC

>V300080312L3C005R0501039710

ATGTCACGTTGGAGTTGGATGGCGTTCTATACTTTAGGATCAT

>V300080312L3C002R0530083809

CAAGCAACGATTTTAGCATCTGAGGCAGACAAGGCTGAGCAGA

>V300080312L3C006R0500314422

GGGAGGGTTCTTGAGAAAATGCGGGCCTTCCCACGCACGCACC

>V300080312L3C005R0530294415

ATTCGATATACAGTAACCGGTCCATTTATCAGTGCAGTGACGA

>V300080312L3C001R0350258946

CACGCTCTCCCACTCTCTCTGCCATTCTGTGTGCAACTCTTTC

>V300080312L3C006R0200423337

GTGTACCCAAGTACAGCAGTAGTGAGAAAGTTGCCAGTATATA

>V300080312L3C004R0250470153

TGTCGGCACGTATGTGGACGACGGACATTTTCTAATGGATACG

>V300080312L3C003R0620085934

CATGGCAGCCAACGGCTCCAACATAATGCTCGATCATCTATTA

>V300080312L3C003R0450628914

TTTCGATAGACATCATATGCAGATCTTTCGAAGTTTTCTCTTG

>V300080312L3C004R0210202423

CTTTCAAAGTTTTCTCTTGAACTAAGCAAAATTGCAGGTTGGC

>V300080312L3C005R0120868893

AGCTTTTGCAACCTGTTAAGCACTAAACTGCCTGAATTTTTTC

>V300080312L3C001R0210636429

ACTGTGGACAACAGACTGGATGAAAAAGGAAAAGCCCGATTGG

>V300080312L3C001R0091255634

CCGGGACAAGCCAATTTTAGAAAAGCCGGTAATCCGACGACGA

>V300080312L3C006R0481293694

GAGATAGGAAAGAAGAAGAAGACAAGTTACTATTGTACAGGCC

>V300080312L3C006R0150773844

ATTTTATAAGATTGGAGACTCTTACTCGCTCTCATGTGCTGTG

>V300080312L3C005R0671235769

GCATCAACTGATGTGCAAGCCTGTAATTATCGTTGTTGCCAGA

>V300080312L3C002R0070526498

ATATCATTTCTAACGAAGTCTGCGTTGTATACTCAATCGTGTA

>V300080312L3C006R0230957517

AGAGAGAACGAGAGACAGGGAAAACCACAAGTGCCCATGTTGG

>V300080312L3C003R0270680146

TCAATGTTCCTTATCATGATAGTAATCTGACGCAGCTCTTGCA

>V300080312L3C006R0670924128

GCGAAACTGTGCGAGATTTGAAACTCCAATTGGCTGAAGCGCA

>V300080312L3C005R0511158683

TGAAACGGTGAGAAATCGAGTGATTGGTGTTACCTATATTGTT

>V300080312L3C001R0301100270

CGAGGCGCGCCAAGTGGATGAAGTGCGCCGGTCCTATGAAACC

>V300080312L3C005R0310010492

ACTGGAAAAGAAACAACTGCAGAAAACCATCCGCACCGAAAAT

>V300080312L3C005R0081401037

CTGAAAAAGAAGACGGATGACCTTGCCACAACCACGGCGCAGT

>V300080312L3C004R0610896295

TTTTTTCCATAAGATCATCGTTAAACCAGCGCGTCAGATTATC

>V300080312L3C003R0500493272

TCCTTGAAACCAGTGATGATGATAACGGGTTAATCATTTATCT

>V300080312L3C004R0591291645

GAAGTCCAGACTGATGGGTGACAATAATGGCTTCCAGAGTATT

>V300080312L3C005R0430107020

ATGAACCATATATAAACGCAAACATCAGTGTGATGACTTGCAA

>V300080312L3C002R0280438331

AAAATTTAGGCTATTCTCTAAAAGATACTTATAGGAAGTTCTC

>V300080312L3C003R0481366299

TCTTGATAGTAATGTTTAAAGGTATAATAGTTGCGAAACATGG

>V300080312L3C004R0271320305

ACGACGAGCTGATAGTTAGAGATTATCTAACTTTCAAGCTAGG

>V300080312L3C003R0371123571

CAATCCAAATTCAATTGCAGCTTTCTAGATAAAGTGGAATTTC

>V300080312L3C004R0080007193

GCTTGTGCTCGTGCAGAGAATGACGTGCCATTACACGTATTTG

>V300080312L3C005R0660376003

TATAGATCGAATGGAAGATGCTAACGTTGTTCTTATACTAGAT

>V300080312L3C002R0571309353

CGCAGGAACATGTGGATTGACTTGACTGGCCTGCAGTGGTGGG

>V300080312L3C001R0251192412

TTCATTTTCTACATCTTTCTTGGCTGGAGAATTAGGCTTATTC

>V300080312L3C002R0060849103

GGAGAATTAGGCTTATTCTTGCCCTTCTTTTGGTTCTTCTTTT

>V300080312L3C001R0350573885

AAGAAAGAAAAGAAAAAGGATATGTAGCGAGAGATCGGGGGTA

>V300080312L3C003R0031056569

TTCAAAGGTGATAGCCGCTGCGCTGACACGTGCCCTGAATTCC

>V300080312L3C001R0090129489

TAACCGAAACGTGAATTCTTCCTGCATCGTATTGTTATCTTCC

>V300080312L3C004R0430288427

CGATATAAGTGTACCGTCAGATCCATCAGCGGTTTTATCCTTG

>V300080312L3C001R0111257096

AATTGGTGCTTAGGAAGAACAACAAATAACGCGATTCGCGAGT

>V300080312L3C003R0090543763

TATCACGGATAATCTTGATTGCCTCACCACCACCGCTGCTGTC

>V300080312L3C004R0550648594

TGGCAGTGTGTAGCAACTGAATTTGGTTGCCGCGATACCGCGG

>V300080312L3C002R0380236493

TGGCAACAAGCAGCACAGAGCTGGACGAGGATCGCCCGTTTAC

>V300080312L3C005R0270934762

TTTCCACACCGTCCTCGACGAAGACGTGAAATGCAGGCCATTA

>V300080312L3C006R0610652685

CCTTACATGGACGGCTATTTCTGGAAGAATAACGGCAACACTG

>V300080312L3C006R0450483058

AGATATATTGATCGTATATCCATGATTCCTGTTTCTGATTTGG

>V300080312L3C005R0571178062

CGCTGGTGTCGAGGGCCTATCCGGTTTTGTCCATGTAAGTTGG

>V300080312L3C006R0160813158

CGAGTACAGGATGTCCCTATCGGTGAAATCATCGAAGGCACTG

>V300080312L3C005R0300099311

AGGTCAAGGGTCGTGTTTTGTCCATTGATCCTGTCAAGAACCG

>V300080312L3C002R0080777368

CAAAAACTCATTGTATCGCTGACGAACTTTAAGAAGGAGAAGC

>V300080312L3C001R0391013343

ACGCCATTGTGAGACTGGACAAGAAGCGCAAGCAGGTCGATCT

>V300080312L3C005R0240533478

ATATCTCTCCCTTTCCACGCACAGCGATGATTTTCTTTTCTTT

>V300080312L3C004R0450916534

CATGTCCGGCAGCCGTCAACCGATTCAAAATTGGAGTACCTGC

>V300080312L3C001R0600492543

GATCTTGGCAGACCTTATCCAGTCGCTCAACAACATGCCCGGT

>V300080312L3C003R0400939983

TCAATGAAGGCTAGCGACGAGATCACAGAGGAGCAAGCACGCC

>V300080312L3C006R0701019460

GGCAGGAACAGAATAAAAGGAGGTAAACTATGGAAAAGGACCA

>V300080312L3C006R0600348935

ATGTTATGTACAAAGAGTAATGCAAGGGAGAAAAAAAGACAGC

>V300080312L3C005R0270726640

AAACTCTGGGCTCGGCATGGATGTTGATTGCCAGTCTGTCTCC

>V300080312L3C002R0200460753

CGCGTCCTTCCATTTTCATGAGTTCAGCGATGAATCCAATGTT

>V300080312L3C006R0690160606

CCCAAAAAGAGATGCCCGTTTGCTTTTGCTGCCTGCTGCGATC

>V300080312L3C002R0270457792

TGGTTTGTCGTTGCCGTCGTTTCTGGCACGTTTTGTCGGCTGC

>V300080312L3C006R0280946964

CAAATCCAAACAAACATGCCATACATTCGTTGTACCTGGATGT

>V300080312L3C005R0481229846

CACAGCGCCGCATATTTCATCTTCTACATCAAACTGCTCGCCG

>V300080312L3C005R0011014213

TATCCCAGTGACTCACCTCCCATACCGGGCGCACACCTTGCTT

>V300080312L3C002R0190900472

CTAGGTCCCATCCCCTATTGGAGGAGTGCAGGTGCAACGCAGG

>V300080312L3C001R0110513591

ATTATTTTCTCTTTCTTTGAATCTCCTTACAAAGCTTTTCTTT

>V300080312L3C002R0500851551

ATGCCTTGCCCGTTTCTATGCAGACTGATGAACCAGACACCAG

>V300080312L3C006R0170877348

GCACCAGGCAGTATAAAGTTTCGCTGTTCCCTTCTTGATCAAA

>V300080312L3C001R0650800873

TGACTTTTAACAAAATGCGACATCAATTTGTTCACTGGGGGGG

>V300080312L3C004R0340144392

GCCCCTTTGTAATATTGCGGGGCAGCATGTTTCACGAAGCTCC

>V300080312L3C004R0690918291

CGGCAGAAACTGAACAAAAGTTTGGACGATCAGAATTCACTTT

>V300080312L3C005R0570088972

GTGGAGATGGCTGGATTCAGAGTGGCAGCTGAATTTTGACGGC

>V300080312L3C001R0611238601

GCAGAAGTACTACTGTAGCACCTGCAACTCCACCGATTGTATC

>V300080312L3C002R0270485661

GCTGCCTTCTCGCTCTGCTCCTTTGTGTTTGGACTATACAACT

>V300080312L3C006R0190130532

CAATCATACTCTTGCTCCTTCTCGTCTTTCTTACCTACCTGGT

>V300080312L3C005R0450951718

CCTTGTCGGTGTCGTAGGATGGGTAAGTTCTAGCCGTATGGTA

>V300080312L3C001R0460956291

ATCTTATAGAGTTAGAAAGATGTCCCTGTGACAACGTATAAGG

>V300080312L3C002R0330152035

TGCTGTTGTGTGCTAGTAAGTCGATTTTACTTTGCAGATATTG

>V300080312L3C004R0550023511

AGCTACAGATCTGTGATATTATATTCTACATATTTCTGCCATG

>V300080312L3C003R0301332858

TCCTTTGCATGCAGCGTAGTTGACCTTGATAGCCATAGATTGC

>V300080312L3C004R0210496452

GCAGCTATTGAAAAAATGACCATAAGTACTGTAAAGTGCAAGG

>V300080312L3C004R0370374078

AGTATCAAACGTACGGACATGCACGGCTGCTGTCAGTTCCTTT

>V300080312L3C006R0520565666

AGTATCAAACGTACGGACATGCACGGCTGCTGTCAGTTCCTTT

>V300080312L3C001R0590669888

AATTTAACATCTTCTCACGTGATTAGCAGGCGCATTTTCCTTT

>V300080312L3C005R0520961421

GACGAAATTTTTAATGCAATGTGGCACTGATCAATTGTTTACG

>V300080312L3C001R0440122065

CTATTCAAATAATTCGTTGACAAACCAAACTTGACATGATAAG

>V300080312L3C006R0531131635

AGTAGCTGCATATGAATAAACTAGGATGCAACTACCGTTTACG

>V300080312L3C005R0480558583

CGGGTATGTCAGGCATTTGTCCGGTCAATTGCAACGAAAAAGG

>V300080312L3C003R0240880852

CGCTTGCTGCCAACGGTAAAGAAGGATTTTACAAGGGCCGTAT

>V300080312L3C005R0590548287

CGGCTGATTATCTCCACATAATCGTCGAAGTATTGCGTCTTGC

>V300080312L3C003R0391216418

CCATCATCCCATCCATGATCACTCGCAAAGCCCAAGACGGCAA

>V300080312L3C001R0700836484

TAAAGATTCTACTGGATAACAACGGGCAGTTCGCCTTACAATG

>V300080312L3C002R0350806559

CATCGTCAGGATTTGGTTGCAGCAGTAAAGTCATGATAGCTTC

>V300080312L3C002R0080414984

AAGCATATCCACACAGATAGAGCCATCGTCGTCGATATTTGGG

>V300080312L3C001R0380295016

AGCCATGTTTTGTTTTTTCAAGTGCAATAATGAATCGATATTC

>V300080312L3C001R0140563308

AGTGCAGCCTGAGATTTCCACAGTCACCAGCCGAGAAAAGAAA

>V300080312L3C003R0550256186

CCAGCTTAACTGTAATCTACCTCCGCGCCTGCACCTTCCGGGC

>V300080312L3C001R0191347547

GCACGTAATATCACACCATACAAGCCTGGTCATACCCATGCAC

>V300080312L3C005R0380597160

CCATACAAGCCTGGTCATACCCATGCACCAGAGCTGTTACAAC

>V300080312L3C001R0551064445

CCTGGAAATACGTGACAAATAGAGGTTTATGTAGGCCAAGCGA

>V300080312L3C003R0010860253

GGCGGGAGAAAAGACGAAGGAGAGGCGCGAGACGGTTGATATT

>V300080312L3C002R0460513319

CTCATCTACATCATATTACATATAAATTGTACAGAAAAGATCT

>V300080312L3C001R0610574559

CAGGATTTTGCTTGAAAATAACGAGACTTGAAAGTCGACGTCG

>V300080312L3C004R0550537986

CGCGCGAGAGATTCACGCACAACATATGCGGCCTTTTGTCCAG

>V300080312L3C001R0280106890

CACCTATTCGAAGGACATGTGAGAAACTGAAGGAGAATAGACA

>V300080312L3C006R0550236818

GAACCCAAACGGTAGCCAGTGGCGACTTTTGTAGCAGTAGCAG

>V300080312L3C001R0100449498

AACAATGCTCATCCAAGAGAAGCAGCAGAAGTTTCCAGTCTAG

>V300080312L3C003R0471379371

TTTTGTCATGTACTGGTGTACACAGTCATGAGTATATAGGGAC

>V300080312L3C004R0391107123

TCCCAGACAATAAATACTGAACAACAAACGAACCGTTGGATTT

>V300080312L3C005R0560923107

TATCCAACTCCAACAGCCTACGTAATAACTATCCTATGTATAT

>V300080312L3C005R0650247882

GCAACTATTAAGTGGATCTTCTACCATTCTCGTGGGCGTAAAT

>V300080312L3C002R0270183705

ACTATTAAGTGGATCTTCTACCATTCTCGTGGGCGTAAATTAC

>V300080312L3C002R0270969184

ACTCTTGCCAATTCTCCTTTGCTTTTATCGATTTGAGTCTTGC

>V300080312L3C002R0540401855

CATTCATCAGCTAGCAGATCCGGCACTTTAAACTGCGGCCACA

>V300080312L3C004R0010867922

AGTTTTCCACCATCCAAACGTCGCGTCCAGCTGCCATCCGTGG

>V300080312L3C001R0490539751

AGTTCTTGCTGCTGGCAGCATTTCTTGCATCATACAATCCACC

>V300080312L3C003R0270553764

AAAATACTAATTGCAAAGGCAATCAAGTATATATATATACTGA

>V300080312L3C006R0431335907

AAGCTTGACTTCGCTGCGGCTGCTTATTCGTGCCAACAATATG

>V300080312L3C005R0550569514

TGGGTTAGGAGCAGGTACGGATGACTGATTACCGACTGCAGGA

>V300080312L3C005R0490776358

TGTCTGTTGTGATTGGAGATCACTGGATCCATTCTCTTCGCGC

>V300080312L3C001R0460478065

CCGTTTACAGTCGAATGGGTGCAGTTGCTCGAACAATTCTACA

>V300080312L3C005R0441001341

CAAGCCGAGAAGAAGACGAGTGTCCTGTAGAGATACCAGAATT

>V300080312L3C004R0291056255

TTTGCATGCGATGCATTCTACTGGCATGATGCTCTAGATAGGC

>V300080312L3C002R0070339337

GAAGGGTCTTGACTTGGCTGTAAGTAGTAATCCAACAACACAG

>V300080312L3C003R0470573364

TATTTAACCTCTGTAGCCGCTGATCTCATGGTCGCAGAATACA

>V300080312L3C001R0130679711

GGAATACGTGGACGTTACTTCGCCGAAATATCAAAAACCGCAA

>V300080312L3C006R0130444484

TATAATCTCGATTTTACGGTGCCGAAATCAAAAAAACCCCAAA

>V300080312L3C002R0201327574

ATCGGCGGCGACATTGACTCTGCACAATAGTCAAATTTATTCC

>V300080312L3C006R0591254605

CCTAGCGCTCAAGTTTCAAGTGATTCCGATGATTCTGACGATG

>V300080312L3C002R0390740619

TACCACTATATCTTGATCCCGTTCTGCCGTGATTTTTCCCTAC

>V300080312L3C001R0500655234

GACTCTTGTAACTTTCTAATACATTCTTGACCAAGTTCAGCTG

>V300080312L3C006R0640314162

TTGAGCTTATCGTGTCCGTGCACTTCTCGATCCATTTCTTCCA

>V300080312L3C005R0191066384

TATCAGCAATCATCTTGTCGCGTGCTAGGCCCATTCTTTGGAT

>V300080312L3C003R0421127251

GCGGTTCTTTCAGCAGTACGTACGCTGCACGTTTGGGTAATTT

>V300080312L3C002R0230788654

GGAGAGTCTCCATTGTATAAAGCAGGTCGGAAAGCGAGGTTGG

>V300080312L3C003R0721383182

TCAGTTGTCAGTATTCTCGGAACATTTTGACACTTTCAAGTAT

>V300080312L3C005R0210850263

ACCGAAAATGCGTCACTCTTCAGTGTGTCCATAACCACGGACA

>V300080312L3C004R0350623852

CAAGGGGCGGATGCTTCGGACAGCTCGGGCCTGCCCCTGTTTC

>V300080312L3C006R0410714227

AGTCTAGCAAACTTTTACATTATTTGAGTTAGTCGGGAATAGA

>V300080312L3C005R0221137417

CAAGCACCTCGATTCACAATGGACATTCCTAGTCCTAATCGAA

>V300080312L3C002R0511359397

ACTAGATGGAAATTGTCAAGCTGGATGAAGAGGAAGATAGACT

>V300080312L3C002R0650855552

GGTCCCTTATCCAGGTAAAGGCGCATGGATAATATAGGGATAG

>V300080312L3C002R0380827453

ACCGATAGTTCTAGACGTCCATCCGCTGACGGCACCGATCGAT

>V300080312L3C005R0331208239

GGCGTCCATCCGCTGACGGCACCGATCGATTGCAACGAAGATC

>V300080312L3C006R0050981104

TGTCACAGAATTGATTACCAACAGGTAACAAGAAAGAGAGAGA

>V300080312L3C006R0140811260

TACAGGGTGAATGATGCTATAATATACAGGAGTGCGAGGAATT

>V300080312L3C005R0490771995

GACTAGAGCGAGCTTTCTGCAGGTAAAAATGTGATGCAAGCTC

>V300080312L3C003R0670816000

AGATTACCTGAAGGAGCAGCGGCCGTCACTTGCCATGAAAGGA

>V300080312L3C001R0430462952

GAAGGAGCAGCGGCCGTCACTTGCCATGATAGGAACCCAAAGG

>V300080312L3C005R0171148500

AAGAGAGTGTGTTACCGTGTGTATATGTGCCTCCTTTCGTTCG

>V300080312L3C003R0261346310

GATTCCCCCGCACTCCCTGCTTTGCTCACGGGACATCGTCATT

>V300080312L3C006R0031222227

GCGCCCGTCACCTTCATCAACGGATATGATGCTAGAAGAACCT

>V300080312L3C002R0280255537

CCCATCCTCCGCCTTCGTGGGCGTAAACAACTATATTTTCTTG

>V300080312L3C003R0450129113

GGACTACACCGCCGCTTCAAACGAATACCCTCATCCCTCCACC

>V300080312L3C001R0680358354

ACTGGCTGCCATTCTCGCATATGGCGAATCACTGTCCAATGCC

>V300080312L3C005R0691056380

TATAGCGCTATATGCTACCCCATTATGTATTGAATAAATAGTA

>V300080312L3C005R0160715379

CTCAATGTAACTTGCATGATTTATATGCTCGTTTGACGTAGCC

>V300080312L3C005R0680965699

TCAAGTTGCTTCTACGCGCAAACATACGGCTGAGAAATGCTCG

>V300080312L3C003R0190319236

AGTTCATGAACGAAGAAGGTACCAAACTTGCCACTGCTACGCA

>V300080312L3C004R0411142972

AGGCAAGTACTTTTGTACGGGCATGGATCTCTCAGCTTCAGGC

>V300080312L3C006R0540480466

TTGACAGAAGTAAAACGCGGCATCATTCCAGCCATTATTTCGC

>V300080312L3C003R0620247493

TGGAACATTTTGTTGACGGCATTGCTGACTGTATGGTATCCTA

>V300080312L3C004R0660116526

CTGACTACAGAGATGAGAATTATGTATGCAAATATTTACGTTT

>V300080312L3C004R0530848531

ATTGTTGTATGCATCGGTATCTCCTTGCAAAAGAAGAGACCCA

>V300080312L3C005R0110046169

GACGTAGGCTAACTCGCAGGGACGTTTGACGCGATCGAGCTGC

>V300080312L3C002R0120810970

CGATCGAGCTGCTGACTAAAATTGCAGCAAGGGTTTGCTTAAT

>V300080312L3C001R0330309054

TGGATTCCTAACAATACCAGTTCCATTCACGATCGCTATGCGG

>V300080312L3C001R0200842983

AAACTTCTGGTATTCCGTGTGCCAGCTCATCCTACGCTTACCT

>V300080312L3C003R0270425082

GCTTCGTTCTCGGCTCTCCATCCAGATATTCGATATCCATTGA

>V300080312L3C001R0620873499

ATCTTGGATTCTGAATATTATCGAAGTACTAATATTGAGACTG

>V300080312L3C003R0721010876

CAACTGTTTATACGCTTTTACAACATTCTTCCCAAGTACAAAT

>V300080312L3C005R0560485109

CCATGGGAACATTGTCGCTGAACAATGGTGGTTTGGAGGATTA

>V300080312L3C003R0590462808

CGGTCGCGGTGGTGGACATGGCAATACAGGCGGTCGTACACCT

>V300080312L3C001R0701225262

CTCTTTCTCTTTCTCTCGACTTTACAACATCTTTTTTGTTGCG

>V300080312L3C001R0310726031

GCTTTCGGTCTGATCAACCGAGAGTGACGAAGGGTAGCTCCCT

>V300080312L3C002R0640706717

ACTGACGGGCATACCCTGAAGGCCTCTCTCTTGCCTAGCGATT

>V300080312L3C005R0070042880

TTTAAAGCTCGATCCAAGCAGCAAGCTTTACTATCCAGCTCGC

>V300080312L3C003R0640034122

AGTCTGCAGATAAGAGTGAGAAAATAACGAACATAGGAGACTG

>V300080312L3C005R0161317427

TCTCATATGCCCGCTCCTCCTATGGGAGTGCCACCTATGCATG

>V300080312L3C002R0270434073

ACCATTTTACTTCCTTTAGCCTACTATTTCCATCAATATTGTA

>V300080312L3C002R0570025107

AATCCTAATTGTTCCTTTTCCACAGCGTGCCATGGAATTCATC

>V300080312L3C003R0451318873

GATCGCAGTTCTCCTAGCAGCTGAGTCATTGCATCGTTTAAGC

>V300080312L3C002R0080988691

CACGATCAAATATCTCAACGTACCGGAAGAAATCGTGGATATG

>V300080312L3C002R0561234279

CTCTATTTTCAAAACAAGCTTATTCAAGTTATCACCAGTATTT

>V300080312L3C005R0270057371

CTCTGGTAAACAGGCATGCCAAAGTATTCACCCATGATGGCGC

>V300080312L3C006R0301317397

GTGCAAAGCAAGGATAGTCACTGCAGTGGCGCGAGGAGTTGGG

>V300080312L3C004R0060557985

CACCAGGCAGGTTTTACCAAATATGTTAATCCAGCGTATCTCG

>V300080312L3C005R0450129817

TTCCTCCCTCTCTCCCCCCAAAAAATCCTCTTCAATGTTCCGC

>V300080312L3C003R0020122013

CCACTATACTTTCTTTGCGATAAATCAGGATACATTACATTCG

>V300080312L3C003R0400190556

ACATCCGCGTTGTAAGAATTGTAACAGGCAATGTAGATGCAGG

>V300080312L3C001R0541275514

GTATGAAATTGATAACTGCTAAATGACGGTACCACTTTCTTCA

>V300080312L3C006R0081384006

CCAAAACTAACGTTGAAAACAGCAAGCGGATTGACTGATGACT

>V300080312L3C004R0380251547

AAAGATCGACGCTGGCGCTGATCTTGTAGTTACCCAACTCTTC

>V300080312L3C002R0130310654

ACTTGACTATCGAATTTATCCGCAAGTTCAGAGAAGCAGGCAT

>V300080312L3C002R0450287857

CAAGTAACTTACCATGCTGTCAACAAGCAGGGCGACTTTAAGA

>V300080312L3C005R0340343934

CTGGATATCATGAACAACTGGTACCTGATCAACATTGTTCACA

>V300080312L3C002R0280832527

ATGTAACCTTTATATCTCTTGTCATCCTCCCGTTCTTTCTTTC

>V300080312L3C003R0720852227

TACCCATTCTTCTGGAGAAGAAGTAATCCTTGACAAAGAATGG

>V300080312L3C005R0310008470

GTGCCACAGGTTTAGTCATGTCGAGAAGTGAAAGAGAGCTGCA

>V300080312L3C005R0350243537

TCTACAAAATGTATATCACGCAAATGAAACCGGATGATCTCCA

>V300080312L3C003R0131382131

CCAATCTTTTCTCACGATAGCACCCAGGTTGTCAGCATTATCA

>V300080312L3C004R0590273965

CTTTATTGTTGCTAAGATTCCGGAGGTCGCACCAGTTATCGTG

>V300080312L3C004R0360947554

GAGAGATAAGAGACAAAATGACGCGATATCTTTGCGTCGTCTT

>V300080312L3C002R0510397654

CTTTGTGCTGACAAGATCTATCTTTTAGGAAAAACGAACATGG

>V300080312L3C005R0210365758

GCTTACACGGATAAACCGGAAAAGACCCGTCATCTGCTTCGCT

>V300080312L3C003R0510695166

AGCGCATAGCAATTATCTTCCATGAAAGCCATGTATCCAGCTA

>V300080312L3C005R0320850830

TTGTGCACAGACACAGTCAAGTGTAACTTTTGCCTGAACCGCT

>V300080312L3C005R0170383995

ACACGTAATTTCGCTGTAGCCTTATTCATAGATTTGTAAGCGA

>V300080312L3C004R0090702241

GAGAACGATCCGGATGTGAGAACAGAACGATGCATAAAGTCAA

>V300080312L3C003R0300309743

CTTTTGCTTTAATGTTAGCCTTCGTGACCCAAACGTTGATACG

>V300080312L3C005R0250015547

CAGTGTAGTTATTCACGCTTAAGCAAAGTACCATCAGATAGCA

>V300080312L3C004R0231211036

GCATGCTTTCCACCCTGTCCCTTGGTACTCGAGGACAAGGACG

>V300080312L3C002R0460735756

CGTTGTGCTTTCTCTTTGGCATGGCGAGGTCCTCTGGAAGACG

>V300080312L3C006R0400918584

GATGCTGTAAGTGAAAATCGTTCCAAGATCGCATATACCAGTA

>V300080312L3C001R0441220801

TACTGCAGCGTTTTTGCGGGAGGAACGTGCGACTTTTCGTTCT

>V300080312L3C005R0360079526

CGACATTTCCTCGTTTGCAGTCTCTAAAGTTTCTAACCTGTTT

>V300080312L3C006R0080618666

AAGCGAGACAAGATCCTTCAAACCTGATCGAATATAGTTGACC

>V300080312L3C003R0720835856

AGCCTCGCCTAGCATAGTAGGTGACATGTACTTTTAGCATACC

>V300080312L3C002R0200730924

CCTTTGCTCTGTTCTCTCAAACATCCTGGTACGTTGGCTCCTC

>V300080312L3C001R0110721261

GATTACGCAGCAGTAGCGCAAATTACTAACGTAATGCTCTTAA

>V300080312L3C005R0581346293

CGGGGTCGAGTTTCCCGATCTTCTTCTTCGTTTCCCTTGGTTA

>V300080312L3C003R0160341960

CAATGCTTCGATGGCGTGCTGTTAATGCCAACGACATCTCTTC

>V300080312L3C006R0720128055

TCTGAGACGTGCAGGGTTAATATCGGCGGGTTGTTTCCGGGCT

>V300080312L3C003R0181187523

GCATTAGATACATCATGTAGTTGGCATTACTAGCTGGCTTCCA

>V300080312L3C001R0421065231

TCCTCCACATCTTGACCAATAAACGATAGAACCAATTTCACGC

>V300080312L3C003R0181286672

GAAAACAATAACATTGGCGGTTGCTTCCATCTGTTCACAGTTT

>V300080312L3C003R0191047561

TAAAGTGTTTGGGCTGGCGGTCGTTATTGGCTCATTGCAGCAC

>V300080312L3C001R0180253266

ATACGCTGACTGGCGAAAGCGAAGAAGAGTCGCAAGTAGCTAG

>V300080312L3C001R0581146417

GATACTGCTATCTGCTATGCAAATTACCGCTGATCTCTCACAA

>V300080312L3C003R0510926934

GGCTTTTGCACAGATTCAGTTGGAGGATGCACCTCAGCCGACA

>V300080312L3C003R0320785237

GAAAACGATGTGAGTTCTGCTGCTACGGAACATGACGATCGAA

>V300080312L3C002R0451193695

TGTACACCTGGGTGATTATCAGGCTGCAGTCGTTTGTGCACGC

>V300080312L3C001R0650701958

CTCGCAAAACGACTTGAAAAGCACGAATTACTAGAATTTCGCC

>V300080312L3C003R0471125188

CCGGAGCAGCTATATGCTTTGACAAGTAATGGAACGGTAATTG

>V300080312L3C003R0080926243

AACATTCGGTGACTCACGCTTTGATTATACTTGGTGACGTCTT

>V300080312L3C002R0160505076

CAATAAATATGGTTCAACAACGAGGCGAGCGGATTCTACAGTG

>V300080312L3C002R0150042429

AAGCCACGGCGTCCGCATGAAAATAAGAAGCATTCTATGTACT

>V300080312L3C001R0530837527

GCCTTTGTGATGGCGAATCCGAGGCGCGTCAGCGTCTGGAATG

>V300080312L3C006R0280631251

ACTATCAAATCTGGATCTGTCTCCATTTTGCAAAGCTCTCTTG

>V300080312L3C001R0540268403

GCGTGCAACTTTGGATTTCTGTATTAATGCAAGCATTCCGTAT

>V300080312L3C004R0050775257

GTATCCGATCCGGTCTCACCTAGGATTGCTTCTTGCTCCAGTT

>V300080312L3C004R0690723425

CTTGCTCCAGTTCCTCTTCCAGATCTTCCTCTTCGTCTTCATC

>V300080312L3C002R0311121835

AAGCTGTACAGATATCGTGCTGATTGAATGTTCTGGTTCTCTG

>V300080312L3C005R0360479443

TTTCATAACCATAGACTGCGACGGATATACAGGAGGGGGACTC

>V300080312L3C001R0601380370

CACCGAGCTCCTTTGCGAAATTGCGCTCTTCGATTTCGAGTTT

>V300080312L3C003R0290455971

GTTCCACAGGACACACGAGTAAGTCGTCACTTTGTCGTGGTTG

>V300080312L3C005R0620698932

AAGTTAAAATGGCTTCAGAATTCACAGGTTTAATTTCATGAAT

>V300080312L3C004R0110282193

CCAAGACCTTGTTGACGAGACCAAGTTGGAGACCTTCATCTGC

>V300080312L3C003R0671353495

CAACATTGCATATCTACAGTATAGAGCACTTGCAAAGTTACCT

>V300080312L3C004R0170258261

AGGCGGTTGTGGATTCCGGAGCACCGCATCCAGTGCATTGTGA

>V300080312L3C004R0210787817

CTTAGCGCTCTTTTCCTCTGCGGTCAATGGTCGTTGGCGAATA

>V300080312L3C006R0450335418

TACATCAAGACTGATTCTACAGGTGCGTCGTGGTGCATTGTCA

>V300080312L3C003R0461152838

TCGAACCGTGCGGTCGTCTGAGGCGGTTAATATGTGTGTTTTG

>V300080312L3C003R0450612157

CGTATTAATCCACAATGTGGATTTGCACATTTATGCAACGCAA

>V300080312L3C003R0201193024

CTTGCAACGAAAAACAGGTTAGTAAAAGAACAAACAAAAGGAA

>V300080312L3C003R0350451597

AACAGGTTAATAAAAGAACAAACAAAAGGAAAATGCAAGGTCC

>V300080312L3C004R0510705848

TGGTCAAACAGGAGAATGGTATTTATATTCGTTTACTAAGCAT

>V300080312L3C006R0661090071

CAACAGAATCGACTCGATCTCAGCCTTGCCGACGGTGACGTCA

>V300080312L3C002R0720118458

GAGAAACGTCGTGTCCATCGTAGCAGTCGCAAGTTCCAAACGG

>V300080312L3C002R0610617264

CCAGATAGACGATCAGCGGGATGAAAACGCGGGCACCGAAGAG

>V300080312L3C003R0380924055

CCTCTGATGCTAGGCAATATCGGAAGGAAATCAAGTGCTTTCC

>V300080312L3C005R0600188748

CAAAACAAGATGGGCGAATGGTACGACAAGGTTGTCAGATACT

>V300080312L3C005R0591115544

CTGTAAGTTACCCCATTGCTTTCATATTAGAGCAATCAACTTA

>V300080312L3C004R0230856000

TGACTCGAGATTTAGGTAACAACAGTAGCGAGAATGCTATCAT

>V300080312L3C002R0590030439

CTGCGCATGGTAACCACGGGTGCCAGCAGGCGAGTCGCAGACG

>V300080312L3C005R0510269568

GCCACACATCGATTCTGTATCGCTACGCGCGCACAGGATGCTC

>V300080312L3C002R0100801683

GCGAAGAGGAAGCGCTGGCGCCGCTAACGCTGCTGCTTGCACC

>V300080312L3C006R0390484852

CTTACTTTACAATCGTCTTTGTACCATTGACATGCTGCTGGAC

>V300080312L3C002R0631251546

CAAGGATGTACACTGTTACACCGGCGCCTTGACGCGTAGGGAA

>V300080312L3C002R0460623352

TTGGAGTTTGGGTTGCACGTTATTTTTGAGATTAATGGTTGTG

>V300080312L3C002R0331098009

GAAAAAATCGATTTTCCAAGGCAAAATCGAACAAGAAAGGAAA

>V300080312L3C005R0590104075

AACATTCAATTCAGCATACCAATGGAAAGCTCCATCACCTCAT

>V300080312L3C001R0640349665

CGGGCGTTAACTAACCGCATATAACTTCGTGTAAACGTTGAAC

>V300080312L3C006R0310155977

GGTAAAAAAGTTAGAACCGACATAGATACGCAAACTCTTTAAA

>V300080312L3C004R0370608988

GAACCGTGTACATGAAAGATCATAAATAATGTTCTTTAAGACA

>V300080312L3C006R0040146256

GTAAGTACAAGATCCTACTAATTGCTTTGTATACTACTACTTG

>V300080312L3C002R0180409538

GCATACGGCCATGCTGCCACATCTCAGTGACGCACAAGCCGAT

>V300080312L3C005R0650201238

GCAACGGCATTCGAAGTCTTTTGATAAGACCTTCCATGATTTC

>V300080312L3C003R0720473877

TTTCGTAGTTCAATAAAGCCCTTGCGGGCAATGCGACGGCGGA

>V300080312L3C001R0090022410

TATCGGATGCGTCCTTGTTATCCCAGTGCTTGGATGATACGAG

>V300080312L3C004R0420563682

CACTAAAAACTGTCCAAATGTAAGATGGTGAGTAGTATTACCT

>V300080312L3C005R0170333359

CTCCAACTTGAAAACATGCTGGTTGAACTGTAAGCAGAGCAGT

>V300080312L3C001R0310476975

ACTCTGCAACATCGTCGACGTTTGGAATCGTGCCTGTTCCACT

>V300080312L3C006R0210410601

CCTATTATTTATTCTTGTACATCTATTCTTGTTCTAATCCTTT

>V300080312L3C006R0630997242

TCCTGTTTTCTGCCACTCAGGTCCTTCGCGGACGTCGATAATG

>V300080312L3C004R0610447040

AGTTGATGCCTGTATGTCACGACGATACATAGTTACATATTGG

>V300080312L3C001R0200742293

CAATTGTATGCGGCGATGGTGTAGCAGCAGTCATGCAAAGCAA

>V300080312L3C003R0230243187

CCATACGTAAGTCTGGATAAACCGATTGCGTTGCTGCCCATTT

>V300080312L3C005R0141181764

GCAAGTCTGTGTTTGTCAAGTTCCACTGGAAACCTCTCCAGGG

>V300080312L3C006R0140451367

AATTTCAATAGGCCTGACTTTCATTGTTACCATTCCCGTAACT

>V300080312L3C001R0070665943

GTCTCCAACGTAACACGCCTTCGATGGTCGACCCTGACCAAGG

>V300080312L3C004R0350989066

GCACCCGCACGGTCGCTATTCTGACAGCTCCAGGTACCAATAC

>V300080312L3C002R0681231848

TTGGCTAATAACAAAAGAGTAGAATAAATTATCCTAGGTAAGC

>V300080312L3C004R0490572786

TATAATAGCTGCTTTAGCGCCCAAAATTTATTGGAGCAACCCC

>V300080312L3C005R0310238531

ACCAGCTTTGTAAATGGCGACGACAGCACCACAGAGTTTCGCA

>V300080312L3C002R0290165450

CTTGCAGCGCCCTACACCAGAAATGGAGGAGACGGCAATGTCG

>V300080312L3C004R0161147690

TTGGTCGGAAAGTACAGCGGCAAGGATTCCATCGCTACAGCCC

>V300080312L3C001R0720425404

CCGTGGACAGGTCGACACCACCGGTCGCGTATCAGCATATCTA

>V300080312L3C003R0220544483

ATCTACAGAAGGTTACTCTTGACCGAAACCCATGGTTTCTTCT

>V300080312L3C004R0541073542

AGAAAGGATTGAACATTTCATGTGACAGATAAAAGAAAAAGTC

>V300080312L3C005R0130797154

CAGAACGTTTCAGCATAAACGTCGTTTATTCTGCAATTTATGC

>V300080312L3C005R0691090395

TTCATATAATCGGCATCGACGACCCAACTCGCGTTCCCAGTAT

>V300080312L3C006R0691082254

GAGGATACGGCTACAACGCTGCTTACATTGTGCACGTTAGGGA

>V300080312L3C006R0370301288

ATATCGAACGATGGAAAAGCGAGCTCTTGAGCGGCATCGTACT

>V300080312L3C006R0600368489

GTGCGAATCTTTCTACGAATTTGTTCATTTGGGGGAGAAGAAG

>V300080312L3C005R0360298400

GCGGCTGGCTTCCTTTCGCGTAGAACAAAGAAACGGCAACAAA

>V300080312L3C005R0240104768

CTAGGGTATGGAATGTTAAAATCGTACAACATGAAAAGAGATA

>V300080312L3C001R0471133540

AGCTTCTGGAGGAAGTCAAAGATCAACTCAGTGATATGCTTCC

>V300080312L3C006R0280903361

AACGTGATCAAAGCGTGCAAGCTCTAAAACTTGGCTAGCGGGA

>V300080312L3C001R0640316002

CGAACGGATCCACCATCTTCGACTTACTTTCCAGCAAAGATAA

>V300080312L3C002R0630719896

GCAAAGATAATTCGCTGCTGGTCAGGAGGGATACCTTTTTGTC

>V300080312L3C003R0511400597

CTTTCGTAAATACAAATACTTTAACGTTTCGGACATGATGATC

>V300080312L3C005R0710486355

AACATCGTTAGGTGACTTTTTCATAGACTTTTCAATGCATGTT

>V300080312L3C003R0610133076

TGACAATGAAGACTTCGTTCTTGTATGCACTTTCTAAATCAGA

>V300080312L3C001R0050830023

GAAGTAATCGGATGACCTCTTTATATATTGTTCATCGTGACAC

>V300080312L3C002R0390057650

TATTTTCGGGAAAAGAACCAATCGCATCTGCATCAACCATCGT

>V300080312L3C003R0690335803

GTGACTCTTTGGATTGGTCATTGCGGTAATATATTTTACCATC

>V300080312L3C005R0440672686

GCTTCTTGGCATTACAAATGCATGTGTAGAATGTTTCATAATG

>V300080312L3C003R0100150171

CAGGAGACATCAAGTTTGCCAAGTAGGCATCCTCACTGGCAGC

>V300080312L3C005R0380081290

CCCTCTCGTGTCCTTTCTTTTTCCCTTCCATCAGGATGAGACG

>V300080312L3C005R0380558497

AAGGTCCACCTCCGACCTGTCACGACACCTCATGGAAGATGGA

>V300080312L3C003R0470193388

CCTTGTATCTGTCCTTGTATGTGAGATCGAGATCTGTGGTGAT

>V300080312L3C005R0140943305

AAAAGATGCGATTCTTTTGACCGTTTTTGAGTCCCCTTTTCTG

>V300080312L3C001R0710289954

ACCGATAATCGTCACAGCACCGTGCATCTCGTCATGCACTTGC

>V300080312L3C003R0270677791

TGAGTGGCCTCTCCCAGAAGACTGTATTCACAAGCATGGCATT

>V300080312L3C006R0450038730

GGCATTGTAACAACTGGCGTCGGCATTATCCATTCAAGGAACC

>V300080312L3C004R0550551355

GAGTACCCACTACACTCATTACGATATTTTCCAGTTCGCAATC

>V300080312L3C005R0580424520

GATCGGTCTTATCCAGTAACAATGTGTTGCTGCCAAGCAGCGG

>V300080312L3C002R0430175750

GATAAAATCCTTTTGAACGTCCTCGTACCATGCATCTTTCAAG

>V300080312L3C004R0200520659

GACGAGTTTCTGATAGAGCCACGCCAGGCGTAGCAGAAAATCA

>V300080312L3C001R0470193995

GAAACGCACCGCTGTAGAGCTAATCGCCACACTTCGACGTTGT

>V300080312L3C001R0280687339

GGCTGATATTAAATAATTGGATGTAACGTCACTTACTTCCTTC

>V300080312L3C006R0370896421

AAGTCCCCCGACGTGTTGCGCGAGGCGTTGCATACTCAACACG

>V300080312L3C001R0541157180

CCAACGTTCGTGCTAGCGTATTGTGGAACTGTAGTATCATGTA

>V300080312L3C005R0500769286

CAAAAGTCCAGAGGATTTGCATGGTGACCGCCATACACAAGCA

>V300080312L3C002R0450750113

TGATGTCCGGCACCACATATATCACGACAAAGATTGCGATAGA

>V300080312L3C006R0410431897

GCTGCATGAGGTGAATTATATCAGAAGACATACCGATGCCAGC

>V300080312L3C003R0250642178

CCGCTTAATCCGACTTGCCCGACAACCAAAAAGGGGAATCTTG

>V300080312L3C002R0560146677

ATGCAGAAGATGAAGGCGAGGTCACCAGTGGAGTATCTAATCT

>V300080312L3C004R0670622240

CACGCCGCTGTGCGATTCGTGACCAACTTGCCCATCGGCACTC

>V300080312L3C003R0390850817

TGATCATCCAGCCCAAGTTTTTGACACATATTCCATTCACGTC

>V300080312L3C006R0681164031

AGGGTTGATTGCAGGCTATGGCATTCAACTGAAAAGGAAACTC

>V300080312L3C006R0310579792

AATGTTGAACAGAGTAGTGGTTTATTCAGTTTTTCTTTCCTTC

>V300080312L3C005R0130270832

CATACATAATGAGCTGTGCATTGTAACATACCAAAGCATTGGC

>V300080312L3C002R0500741700

TCCGGATTCGACTTTCTTTCATCTTCTGTAATGGTATTCTTGT

>V300080312L3C003R0250525260

GATTTGGATTGTTGGTGACAGTAGGCACAGGTGGCAGCACGGA

>V300080312L3C001R0210359921

GATGTTCCGATGGCTCCATGACTAGGGGAGGTAAAGATATGGG

>V300080312L3C005R0631166237

AGACAAACCACGATCCGGAGAAGGCCCCACCTTCGAACGCGGG

>V300080312L3C001R0500264359

CTTTCGACTTTTAGAGTACGTTGCTTTTTTGTGTGGGCATTTT

>V300080312L3C006R0481008104

GCTACGCCATCGTTTACGGCCTGTATCTAGCGGCATCGACCAT

>V300080312L3C003R0500777748

CCTTCGCGTCGTTCATCTGTAGCAACGTCCGGCCGATATTATG

>V300080312L3C006R0450984938

AGAATATGTTAGGATGGGAGATATCTTTGGTTCCTTTAAAGGT

>V300080312L3C004R0620429949

AGGTATATATTTTTCGTTTTTATATACACTTACGCCTGGGTCA

>V300080312L3C001R0600250587

GATATGAGGTGAATCGGGAGCATTTTAAGATGAAAACTTTAGA

>V300080312L3C005R0650918930

CATCTTTAGGCGTGAGCTCTGTGATCTTCTTCTTGATTGTGAT

>V300080312L3C004R0561025066

ACATCTTACTCTCGTGTGCAAATGATATCCGCTCCGAGCACTC

>V300080312L3C001R0250615608

ACATATCAAACTTTTCTCCTGAAGCAAACATGCATCTTGTTTC

>V300080312L3C004R0401082907

TGCCGAGAAACTCAAAATAAACCTGACCAAGGTCCATCTTGTC

>V300080312L3C001R0650892311

GCCGTGGTCATTACCACTTTGCCTCCCCTGGTAATGATGCGTG

>V300080312L3C006R0251003593

CACTTCCAGAAATACTTTGCAAAGAGGTCCTCTTGCCACAAGC

>V300080312L3C005R0440539577

CCCCATTAGTTTGCAGATAAACAAAACAAACCGGTGCCACTGT

>V300080312L3C004R0160404906

GTAGTGGCATAACAATGTATCGAGATTGCACGCTCCTCTAGGC

>V300080312L3C004R0020064607

GATTTACAAACAGGGCAAGTTGTTTTTGATTTGTGGTTAATGG

>V300080312L3C004R0040878455

GCACAAATGCCTTTTTGAGAAAGGATGTTGATTTCTTTTCCTT

>V300080312L3C001R0231055022

CAATGCACAGGACGAGCTTATGAACATTGTTAGCTTGCCACCA

>V300080312L3C003R0030474046

GCCTCACAGCTGTAGGAGATGTGGCTGGCAGGTGGCTGCAACT

>V300080312L3C001R0430673227

TCCCTTTCTTCTCTCCTACAACTTGTCCTTTGCTACAAATGTC

>V300080312L3C006R0431117395

GCAAACTACAATCGATACCAAGACAATGGATACGAACCACGTT

>V300080312L3C005R0500230628

GTTAGTTCTCTTTGGTGCTACAGGTTTCACTGGAGCCTTGACG

>V300080312L3C003R0420686861

GGATTTGACTCTGTACCTAGTGATATGGGTACGTTCATGTAAG

>V300080312L3C006R0320602531

TCTGCAATCTGCTTCTCGCACGCAATGACGTGTCGAAACTTAC

>V300080312L3C002R0220374173

CAACAACTCTGCTGGCAATGTGCAGCCTGTCTAGATAATGCAT

>V300080312L3C003R0160957655

CTGCCAGCAATGTTCCCCGTCGATTGAATTTGCAGTTGAGAAT

>V300080312L3C002R0480795864

ACCACCAGTCGACGTCGAGGTAGCGGAGGATGCTTCTGATGGT

>V300080312L3C004R0380146224

GTCGTCCTGCTCATCTGCTTCCTTGTCGTCTTGTATCACTTCA

>V300080312L3C002R0550420387

GTGTGCTTCAGCGTGGTCATTTGTCTGTTTCACGGATTGCGCC

>V300080312L3C003R0270469727

TGAATGTGGACAAACGCTTGTAGAAAGCATCGCGATCCAGATT

>V300080312L3C001R0621120949

TGCACAGGGTGACTCTCAAGGCTCTAGCAATAATCTGGCCATT

>V300080312L3C006R0260280412

CCAAGCTGAAAATATCACCGTCAAGCAATTTGTATCAAGATTG

>V300080312L3C002R0431150678

GGTGGAGAGTAACAGTATCGAAGAATATCAGAAGACAATGATG

>V300080312L3C005R0500111899

GCCTAGCTATAGCGATGGAGAAGAATCCATTTGAGGAATGGGA

>V300080312L3C001R0570996872

CAACATTTTTTCGATGTCACTGTATCTTCAGAAATCGACAGCG

>V300080312L3C003R0450894942

AATTGTCCTTTCCGTCTCGTGTCTAGGGAGGAAATTTTACAGG

>V300080312L3C006R0711152979

CACCAACACGCATACCACCTGCCAGTTCAGCATATCGCTCATT

>V300080312L3C002R0151028460

CCGAAAAGATCCATCATTTATGGACCTTGATAACGAAGACTCT

>V300080312L3C005R0280880761

CTGTCTATACCCGTCCTGGCTCCACCTGCATTAAATAGAAGCA

>V300080312L3C002R0160532666

TCGGCAGTGATATTACATTCGCGTCAGCTGGCGTATCTGTGGC

>V300080312L3C006R0160332972

GTGAACACGTTGGGAAAAAGTTTCCATGGATGCAGCTTATCTG

>V300080312L3C003R0400218956

GAAGAAGACGATGACGAGGAAATCGAAGACGAGAATGAAGAAA

>V300080312L3C003R0110981713

AGCGTAAGATTACCCGACAGGTGCGAATTGTAAAGAATAAGGA

>V300080312L3C003R0251209004

TAACCACGTTTACTTAGCTTTGGCTTGATTGGATAGGCACCAG

>V300080312L3C006R0300583856

CGAAAGAATTCCTGATGGACGTGCGCCAGCACCTCTTGCACAA

>V300080312L3C005R0471234739

AACGCGCAGAGTATATGACACCTCGGATTCTCGTGCGCTGAGG

>V300080312L3C002R0560762610

GACAAGAGCACATTCACTGAATCCTTTACAATTTGTGTCAAAG

>V300080312L3C005R0650299995

CGCACGGAACAAGCTGAAAGTGTGAAGTGCCGTTGGATCCACT

>V300080312L3C006R0430726980

GATAACCTTTTCGTACGAACTAGGGGAATGATCCATAAACATG

>V300080312L3C004R0430515547

TGTGGAGACTGACAGCAATGTTCTTGAAATCCTTTAACAGACC

>V300080312L3C002R0700099251

AGGCAATCCAGAAGAAGGCGAGATAACAAACGGAGCATCTTCT

>V300080312L3C005R0600263759

TTGATCGCTGCCGCAGTGGCGGCTGTACGATTCACGGGTCTCG

>V300080312L3C005R0111248561

TCCCAATCCATACTGTCGGTCTCGTATTTCTCCTTTCGTTCGA

>V300080312L3C001R0491125340

TCAGCTCTTACTATGACGTACTTTGCAGCCGATGGATTGTCTT

>V300080312L3C006R0650610121

AACGCCAAGAGACAGGACAGTGCCGTATTTCGCTGCTTCGAAG

>V300080312L3C002R0170222250

TCCTAGGAAATCCGGCAAATTTTCTGGCGCAATGTACTGCAAC

>V300080312L3C001R0060015939

TGGATTTTTTCAAGCGTCTAAACCGAATACTTGTGTAAGTTGG

>V300080312L3C004R0240162401

TGAGATGATGCAAACGAGCACAGCATGCTGGTAGTACAATTCT

>V300080312L3C003R0191270895

AAGCAGCCCATTTTTCTCTTGTGCGCGGCTCCGAGACCTAGCC

>V300080312L3C004R0691140673

CTCCATTCATGTCTATCACCACCTTGATGACTGGAAGTCTTTC

>V300080312L3C006R0220614509

ACGATATCGTTGTCTGGTGTAAATGCGTTGCGTTTTTCCGACA

>V300080312L3C005R0680879166

CATCGTCAATATACACTGCTTTTTTCGGCAACAAACAGGGGCT

>V300080312L3C006R0480393265

GCAAAAACGCCATCAAGTCAAAATCTTGGGTAAAGGCGGTAAA

>V300080312L3C004R0151114800

TTTCCCAGGCACGACCGTTGAAGTTTGGATGCAAAATACCCCG

>V300080312L3C003R0661133997

AATTGAAAAAAAATTACTCATTCGTGGACAATACAAGTTCAGG

>V300080312L3C001R0381399487

TACTAGAATCGCTTTGGAAACATCTTTGATCTCTATCATGTGC

>V300080312L3C001R0431324023

AATAAAAGGAAAAATGCTGATGCAATTCAGCAATTACAACGTA

>V300080312L3C003R0050207791

CCTCGCACCAATGTCACGTTCGAGTCCATCAATAAGCTCAAGC

>V300080312L3C001R0320515990

GGTGCTATTGCTGTTGGTCATCCACTCGGTGCGTCGGGCGCAC

>V300080312L3C006R0680215508

GGAGTTGCCGTAGTTCTAGAAAGAGTTTAGAGGAAGCATATAA

>V300080312L3C006R0270596568

GCAGTTGCATGGAGAGATAAATCGACCATGCTCGTCTTCTGTC

>V300080312L3C006R0530590556

ACTACCAGACCGGAAGCTGCCTTTTGCAGCGTGTATAAAAAAC

>V300080312L3C002R0290148601

CATCTTGAGTTTGGGCACGATACAGCACCCTTGACTCTGACGA

>V300080312L3C002R0021207161

GAAGCAATATCCGCATGTCTTTGTTGTATTGTATTCATCGATG

>V300080312L3C004R0180597508

AATTGTTGGAGCAAGGTAGAGGGATATTTTGGCCTTTCAGTTG

>V300080312L3C004R0460605890

TTTGCGGTAGATCGTTCTTGTCATCTCGTCTGGGCACGAATGG

>V300080312L3C006R0460019734

CGTAGGAAATTGATAATGCGTGCCACCTCTTTTAGTTGGTCTT

>V300080312L3C006R0020541911

GACTTTCTTTTCTTCCTTTTTTCATGTCAAACAATGTTTCTTA

>V300080312L3C004R0250023521

GGAGCATAGAACAATGAATTGTAAACAAGTAGCGAAAATCGGT

>V300080312L3C005R0640481056

TCCTTATAATTGACGTCAAGGATGCAAGAGCAATGACTTGATC

>V300080312L3C004R0540816596

AATAATATAAATAAACATGTCGTGTAGTTATTCTATTTGCACG

>V300080312L3C003R0350242553

GGTACCGATTACACCCGATCCGATTCCATCGACATCGCTGAGC

>V300080312L3C006R0470994627

AGACAATATCATACAAATTATCGTTGAACTATGTACACGGCGA

>V300080312L3C003R0641052504

TTTGGGAAGAAGATGCGCACGAGAAGGTCGAAACGCAGCAACT

>V300080312L3C005R0561009878

TGCTAATTCGTTTTCATCTTCCGTTGCCTGTGGTTGATGCTGT

>V300080312L3C004R0600605229

TAACTTTTGCTCAGTCATGTCTAGTGGCAGATTTGAAATGTAT

>V300080312L3C003R0560616301

TACGTAACAGTACTTACCTCTTTGTGCTTCGCCAAGAACGCAA

>V300080312L3C004R0140528823

CAAAGCAAATGGTCCCAAAAAACAGTGATGCCGTCATCCTTTC

>V300080312L3C001R0310903065

TCACATAGGACGGGTCTAGTGTCTCGAGGGTCAATAATTTCGG

>V300080312L3C006R0231062058

CGAGATTATCAAATGTACCGTTGCTTGTATGTAGCAAAGCACC

>V300080312L3C002R0250816249

TGTAGCAAAGCACCGCCTAGCGACTCCTTTGTAACGGTCTCGA

>V300080312L3C003R0170658047

GACTTTCTCGAGTCAATAAGCGTCTGTGACGTTATTGGTTCTA

>V300080312L3C005R0550054457

GCTCAATAGAGCCGTCACCTCGCATCGATGCGAGCGAATATAG

>V300080312L3C006R0650701894

TGGTTATCACGAGCCCTCGTCGTCCAATAATATCAGCCAAGGG

>V300080312L3C001R0300534853

TACGCGCTGACGGTTGATTTTATTCTCGACGTTTTGGAAAGCA

>V300080312L3C001R0490180928

CGCAGAGGCAGTGAAAAATGCATTCTTACATGCTTGGAATGGA

>V300080312L3C005R0260109980

TATGCAAACAGGAGAACTGGTAATAGAGTACAAGTGAGCAGAA

>V300080312L3C003R0250680286

AATCTTTTACTTGGAGCTCGTTTCCTGAAAAACCGTGATATGG

>V300080312L3C002R0100418881

CAAGTCTCAGGCCAAGATGGCTAAGCGTGCCGCTGCTGCTGCT

>V300080312L3C001R0390567287

ATATTAGTACAATGCGCTTCTCCACCGAGTTTACTACGAACCC

>V300080312L3C005R0610697911

AGTGTCTCACAGTGTTTGTTAGTTATACGAAGACACCGAGCAA

>V300080312L3C004R0271139012

CCGAGCAACTGAAACTCCTTTCGATTTGCCACAAATGTCTCAC

>V300080312L3C004R0631055436

GCAGCAGGATTGTATCGAACCATCGTCATCGATTCAGGGGTTC

>V300080312L3C004R0451296838

TGTCACTCCAAAAATGCCTAGCACAGTGTGAACTGCAAATTCT

>V300080312L3C001R0020256207

CTTCTACGCTTGATATAGAAGCACCAACAGCTCGTCGCATACC

>V300080312L3C003R0260922431

ATCACAGTCCTCTGTGTCTCCTGACGACAGTGTTGTGGGCCCT

>V300080312L3C003R0420163215

TTCTTGTTATGTATCAACGTTATCATTGCAGTCAAACTGGAGA

>V300080312L3C001R0621030830

TTATAAACAATTCATTTGCATCGAGCTTTCGGTCTAACACTCC

>V300080312L3C002R0151287797

TCGAGCGATTAGCCCATGTTCTCAAAAACCCGCCTAGCCTTGC

>V300080312L3C001R0711065091

AGGGAGTGAGCCACATCGATCAGGCGACCGGTGCATACGAGGG

>V300080312L3C004R0290872750

GGCGCAGTATTTTGGAAGCTAACATTAAGTTGGAGACGAAATG

>V300080312L3C006R0651271096

AGCAGACTGAGAATATGCTCAGCATATTTGCAACTCGCTTGGA

>V300080312L3C002R0690296966

GATCGGTTGATTCGGTTTAAGTCGCAGTCGGTGAGGAGGCATG

>V300080312L3C004R0501103772

CTCCCTCAGAGAGGATTCCTCCGAGGATAGAAAAATATGCTCC

>V300080312L3C002R0710682610

GCCGTCTTCGGTATTGAAAATCGACCGGAAATTCGTCACGAAA

>V300080312L3C001R0101174129

TCCAGCCTAATTCGCCACGCGGAAAGTGAAGAACGTAATGAAG

>V300080312L3C005R0061059187

AGGAAAATGCGAATACACTGTTATAGAGCCTGATTTTGTTTCG

>V300080312L3C004R0630107934

CATTGAAGAAGCTTTTGTGAATCATATCCATGCACCACATCTA

>V300080312L3C006R0180270356

CCTCGCTCGCATCCCACAGCTCCTCCTCCCTATTCACATCCTA

>V300080312L3C004R0120150773

TTGTACATGATCTCATCCAAATTCAAAATAAATAAAAAAGGAG

>V300080312L3C003R0230993813

CCGACACTTTCCTGCTCCAATTCTGCAAATGCTACGATCTAGC

>V300080312L3C004R0631379991

CTGGCCCTGATGTTAGCCTCTGGAGCGACCAGGTTGCCAGTTG

>V300080312L3C005R0280165661

GGGCCGATGTAAAATTCACTTTGTGGAAGGTTGCACCGCAATG

>V300080312L3C006R0361379140

AATCGTACTACGCTTGATCAACAACTTTTCCAGGAACACCAGA

>V300080312L3C005R0230410323

GGTCGATCGCGACACGGAAAACGAGACAGATCAAGCTGTGATT

>V300080312L3C005R0050048115

TGATTTTGCACGATAGAACTGAAGATGTGATTTAGCCACGAAG

>V300080312L3C001R0100608264

CAATGCCCCTGGGGATGCAGCTTGCAGAGTTTCCAATCGATCC

>V300080312L3C006R0100408308

GCTTACACGATACCTTCGTCGGTTCAATATCCCTATTGTCAGT

>V300080312L3C004R0201083063

CTGTTCTCTTCAACCGAAATCCACAGTGGGTCGTATTTCACGA

>V300080312L3C004R0460826535

CCAGGTTTATGATTGCACGTAAACGGTAGGTGATTCTGAGAGG

>V300080312L3C006R0550634838

GTCGACAAGTCGGATTGGCGCAAAGTGTCGAATTTGCAGCGTT

>V300080312L3C006R0041041771

TGTATCACTATACCCGCACCAAGTTTTACTACTAGCTCTACCC

>V300080312L3C004R0661008409

TGGAGGGTACCCAGCTAGCATCTCGTAGATAAGAATACCGAGA

>V300080312L3C001R0420384899

GTTTAGATGATGATAACCCCCAAACCTGCAGCATATTTATATG

>V300080312L3C003R0230255883

AATTTGAGTCGTAAGGCAATCGACGATAAAGACTGGGAAAGGC

>V300080312L3C006R0680426663

ATATCTTTTGGCAGAGCTTCAGCTGCGCAAGGTCATTGTAATC

>V300080312L3C001R0071020295

CTGCCATTTCCTCGACGAAACCGGTTGCATACACGTACGGTTG

>V300080312L3C005R0650492475

TTCGCACGGATCAATCCCGCCGTGCTTTCTGAGTAGCATCCGG

>V300080312L3C003R0650974373

GTGCTTTCTGAGTAGCATCCGGCGAACCTTTGACGTTTCTTTA

>V300080312L3C001R0391088974

GGCATTTCACTTTGGTAAGCACGTCTTCAAACGCAATCTGAGC

>V300080312L3C005R0260687337

AACATCGTCGATGAAACCAGCGATCTTTGCAAAGTCTTGTTCC

>V300080312L3C005R0381162570

GTCGGTTGATCTGACTGAGACCAAGGAATCGAGGCCGCCGTGT

>V300080312L3C005R0260630668

TATCTCCTGTCAATGCAAGATTGTTGGACATCTCGATAGTTTA

>V300080312L3C004R0450709514

TGTCGATACCAAGCTGGACTGCTGGCGAGATTTGATTTTGCCA

>V300080312L3C002R0391062235

TTATGGCTGCGCCCTCGTTGGTGTCGACCAGCTTCGTTTGATA

>V300080312L3C006R0570510146

GTGTATGCCAACTTGATCTGCTTGCCGGTTTTGGCATCTTTGG

>V300080312L3C005R0490132077

CAGACGAGGCATTCATTGGTTCTTGCCATTGTGGGCAAAGTTC

>V300080312L3C006R0470921467

AGCTGTAGCCAATCCACCGTGGATACGCTTGTTGTCTTCTGCC

>V300080312L3C001R0610657445

GCCCGCACCTTGCTCATCTAAAGCTAGACCGTGAACATTCATA

>V300080312L3C001R0100199943

TGCAATAGATGCAAAGATCCAGGCATCGAGGTGAGATTATTGC

>V300080312L3C001R0450763815

ACTATAAGCACAACCATGTAATACGATATCATAAAAGCCTTTG

>V300080312L3C003R0331159869

TGTGAAAGGCAATTCAACTCTCGCGGCGCTTTACACTTCCATC

>V300080312L3C001R0370802024

GAAAGGCAATTCAACTCTCGCGGCGCTTTACACTTCCATCTTA

>V300080312L3C002R0160382658

GACATGCGAAATACTACCCAGTAACTGCCTGTCAGCATACGCA

>V300080312L3C006R0710458398

CGTCGATATTTTCCTGCACCACCTTGTACATTTTTCTAACTAT

>V300080312L3C005R0610738444

CGATCTAGTCCCAAAAGTCAAACGCGTTGCATATTCTACGTGT

>V300080312L3C006R0311353064

TCAAGGACACGCGACCTGACGATCTGTGGCACCGGTTTAGTTT

>V300080312L3C006R0550097671

TAGATGCTTCGTCCTCAGCTTCCTTTACTTTGACACCTAGTCT

>V300080312L3C001R0320432317

GATGCTTCGTCCTCAGCTTCCTTTACTTTGACACCTAGTCTAC

>V300080312L3C005R0720285627

TCTATCTGCGATGCTGCTAGCTGGTAATCGGAACATGCAATGA

>V300080312L3C004R0150220703

CACTTGAGAAAGCGTATAGCGTCGTTTCGGGTCGACAACAAGC

>V300080312L3C003R0660377862

GCTTCACCCACTCAAGCGCTCGTTTCACAAGCAGATCACCCAG

>V300080312L3C004R0350390852

TGCATATTAGTCTCTCCACGGACTCCAAATACCATACGAATAG

>V300080312L3C004R0111033551

TGTTTCTTCTGGCATACATGCTTGCTCAGCAACAGCCACGGTA

>V300080312L3C006R0220356785

AAAAATGTGTGTTTGAAAAGGGAAAAGAAGGAAGCATTTGCCC

>V300080312L3C005R0100850734

TCCCCAAATGATATCTATCATTCGGAATGAGAGTACGTCCAAT

>V300080312L3C002R0321334462

ACAGCCGGCTAAAAGTCTATCATCTGCCTTTTCATATTTCTGT

>V300080312L3C006R0400092259

TATTTACTACGCGTTCAAAAGGTATACTGAGAATATCATATCT

>V300080312L3C001R0720269022

AGTGCCTTGGGCGAAGCGTGGGCAAGCGTAGCCGCCTCTGCAC

>V300080312L3C002R0510701158

CGTTACACAGATCAAGAGTATCCATTACCGCAGAAGACCGCTC

>V300080312L3C003R0270018965

TGGTAACAAAGATTTCCCCGGGCTTGAAATTACTGCAGATGCA

>V300080312L3C001R0460803951

GGGCGCCTCGCCTGAAACGAAACAAGATAGGGCATTGTTTACA

>V300080312L3C005R0350895032

GAAAGCCGAAGGGAAACCCACGGCCACCGAAGGTAGCCTGGTC

>V300080312L3C002R0440726755

CCCCCTACAGATTCCCCTTTATTTATCGCAGCCCTATTGATCT

>V300080312L3C006R0660054560

CCGTGGTTTAGGATTCTGGGGAGGTGTGCGCGCATTAAAGTAC

>V300080312L3C006R0511135166

AAGTACGAGGAGGCCTTGCAATGGTGGCAATTCGTGCCTTGGT

>V300080312L3C004R0630175186

CCAAAGCAGGTTGTAGTCGTAGCTCATTACCACTGGACCTCGA

>V300080312L3C004R0600123360

ATGTTTACACAACGATCCGGGAGAAAGAATAAACGAGCTGACT

>V300080312L3C001R0310111965

CTTGAGACGTTTGCCCGAGGGTAGATCTATATAACATTTCACG

>V300080312L3C004R0661223555

GTGGCTGCTGTGGGAACGTCGGCGAGGCAGCAGCCGTGGGAGG

>V300080312L3C004R0200874496

TGTTCGCACTCTCAGTGCTGACGGAAGCATCAACACTGACGGC

>V300080312L3C002R0531166845

TAATCTTTGAACTCCTCCGTCAAAGATTTGAATTGCTGCAATA

>V300080312L3C001R0430671469

CCAGAATCATTGGTTTGCTGATCTTCATAAAACTCACTGAATA

>V300080312L3C004R0190886298

TTGGGTGAGAATGGTACCAGCCTACCACATCGTATCCTTTTTC

>V300080312L3C006R0560720895

AGAAAAATTACATGTTTCACCTGATCATGAGTGTTGTCGTTTT

>V300080312L3C003R0541288672

ATCCTGCGGCGGCTTGGTGATGGTTTCTACAGTCGATGCTGTA

>V300080312L3C003R0061101644

ATGTCGATAATCTCTTCTTGATCGTCCATGCTGTGCGATCAAG

>V300080312L3C002R0461063085

ATTGCTATTCCTGTAAACATTGTAATTCATATCCATCGAAATT

>V300080312L3C001R0180937448

TTTCTTTTGTTTCTTTTGAATTCTTTCGGGAATTCCCAATGGA

>V300080312L3C004R0570533107

TTTCCAGTATATGATGAGATTCGCCGGGCGAGAACGCGGCGGT

>V300080312L3C004R0040450761

AAAGCGACCGCATCGGGAACATCAGTGATGGGAACGGCTTCAC

>V300080312L3C005R0060224379

TGAAGACGATGTTGACGAGTTTGCTCGCCGTGGTCTGCGTTCT

>V300080312L3C002R0470572944

CGTTCGCATGGCTTCTTCTTTATGGAGCGCCCGTCCATCCTTT

>V300080312L3C001R0400178472

TACCAGCTAGCAGCTTGCCACGGTACAATTGACCGGTCTTGAG

>V300080312L3C006R0180889598

GGATCTATCGAAGGAGGTCGAGTCGAACACGATTGCGGCACCG

>V300080312L3C001R0430047316

CAAACGTATTCGAGGCATTGCCTACTGTACACGAGTATCACCA

>V300080312L3C002R0640184808

GCCACGATTGGTAGGATTTTGTCACTGTTTCTGTGAGAAAGGG

>V300080312L3C006R0441123719

ATGAAGTCAATACATATTGTTCGAGCCCGGATGTAGTAAAAAT

>V300080312L3C006R0110476973

CTGATGTAATACCCAATATATGTACCTGATCATTCTCTAAGAA

>V300080312L3C006R0610515752

GCGATTCAGCACGCGTTGTATGCAGCAATCGACACGCAAGATG

>V300080312L3C004R0320119792

AACTCTGTTAACGACGTTGACGACGCTATGCAAACAAGCATTG

>V300080312L3C002R0511005661

TCTCGGTCGCACACGCCATCAGAGTTCGCAGGCCATTGCGGGG

>V300080312L3C006R0280524993

CACCCGCGTCACACCAAGGAAACTGCAAAGGGCAACGCCGGCG

>V300080312L3C002R0610921644

CTCAAAGTCTATTTCGTCTTCGTGTCCAACACTACCGTTGACT

>V300080312L3C002R0200188753

TGTCTGCTTTCTCGTGGGACTTCATGTTAACATCATTTCCAAA

>V300080312L3C004R0230205387

TACCCAATGCAATCCCTTCTAGTATCCGTTCTATTACAAGAGT

>V300080312L3C003R0100870372

TCAGCCAAGCTCCTCCTCTTGCGTTGCTGCTCTTCGTATTGTT

>V300080312L3C005R0370252429

TCCCTCGTGATCATGAACAAACAACAATGGTAATACGTTATTA

>V300080312L3C006R0600445651

CTGGCTGGCCAGGTGCAGTGAGATTGACAAACTCGCCTGTGGC

>V300080312L3C006R0551076772

AAACAAAATTGCTCGTTGCATTGATTCCTCGCCCTTCGCCGCC

>V300080312L3C005R0651000393

TACGTGTAGAGTGTATTGTTGAGCACAAAAGTAGCTGCGCCGA

>V300080312L3C005R0300086937

GCAAGCGCAATGTGATCGATACTTTCATACAAAGCTTCAAAGC

>V300080312L3C004R0300620653

GGAGAGGTCGATGCCTTGATTTCGGAATACAACCTTTTGCTGT

>V300080312L3C003R0631159377

CAAAGAAGAAGAAACCATCTTCATCAAGTCTTAGTAGATCACC

>V300080312L3C001R0030452701

TCAACACAAACTAAGTCTGTTTTGCGTATGAGGACCTACTGTG

>V300080312L3C003R0600136039

AATACCTTTGTAAATTGTATGCAACCAAACCACCATCGCACTT

>V300080312L3C001R0690369229

CACTGGGAGTCAATGTGGGCGCAATCGACAAAGGCGGGAGTTC

>V300080312L3C005R0550220774

ATAGTCAACAATCCCTCTAACGACAAAAATTACCGGCGTTAAG

>V300080312L3C001R0170655936

ACCCCAATTCACATGGCTCCACCAGCCATCTTCATTCACTTTC

>V300080312L3C003R0721317384

GACTGATGATCGCTTCTTTTCTTTGGAAACTACCTTGTGTTCC

>V300080312L3C003R0070448270

GGCACAACAGAGTAGTGATGTCGGACGCCTGTAATTCCATCCG

>V300080312L3C003R0630641318

ATATTAAGACTCAAGTTCTCGGGGCTCAATCCTCCCGTATTGA

>V300080312L3C004R0300178282

ACAGTGCTCTGTGGTGGCGAGAAAGGCGCCTGCGAAGGAGGGG

>V300080312L3C006R0600858008

TAATACCAGACTAAAAATAACACTTTAAGTTTGTCATTTACAC

>V300080312L3C002R0570470218

CGCTTGTTCCCCCGCAAACCCGACCTACACCGCACGCGAACTT

>V300080312L3C004R0060152433

GGACACTGCGTTTGAGGCTGCCGCTGCTGTCGGCTTGCCAAAG

>V300080312L3C006R0680023061

CAAACGCGAGTAACTAAGCAATGGCGTGCTTCTTCCCCAGGTT

>V300080312L3C004R0680187909

TTTGAGGATTAATGGCTGTAATTGTCGTTAAACATGCATGAGG

>V300080312L3C002R0410014317

GGTTGACAACGGTTCTGTCAGCGGGGTTTCGGCAAATAGCACG

>V300080312L3C006R0010734287

ACAAAGCACATGTCAATGTTGATCGCTATCATGGCTTTGAGTT

>V300080312L3C006R0560874990

CGTGGTCTGTACAAGTCTGATGATGATAGCCAAGTATATCACG

>V300080312L3C005R0210449866

CAGCTTTACCAATTGCTGATTGAGAGCCACATTTCCAAGAATG

>V300080312L3C002R0310944500

GCTTGTCGCAATCTTTTTAAAAAGAAGCGAGGCAACCTTGCTG

>V300080312L3C001R0370719951

GCAATCTTTTTAAAAAGAAGCGAGACAACCTTGCTGCTGCTGG

>V300080312L3C005R0170872116

GGACCTTTCGTTGACCTGCCCGGTGGTGCCCAAAAAGTCTACA

>V300080312L3C006R0010950038

CAGGAATGAATTACTACTCTTTTTCATCTTTCCAACAAAAATC

>V300080312L3C002R0720648733

AAAGGTAAAGTACAAATCCATTCAACTTGAGAAGAAGGTGTAT

>V300080312L3C003R0030390540

AATTTGACATTCTTGCCCGCTAACAAGGTGCCAGATCTCGTTG

>V300080312L3C002R0590850379

CAACGCATGGCGATTGCTGCTGGCCTGGTATCCATTGGCGTTG

>V300080312L3C001R0530161830

TTTGGCGTTCATGAACATACATTCTTCCCAGAAGCTAATTTCC

>V300080312L3C002R0520112429

CACTTCCCACGCAACTGATCAATAGTGTCCGTTATCAAGTCCT

>V300080312L3C006R0710555078

TGGAGCCTTTTAGATAACGCTTCTAGGAGAGGCCATTGGCTCA

>V300080312L3C002R0671161561

ATTTATGCATATACTGCATGTATCGATTTATGATATAGCTGTA

>V300080312L3C002R0091290784

GAAACTTATTTCCTTTGCATTGATCGTGTCCATGGCCAAGCTT

>V300080312L3C005R0090487414

CAAACGATGATATCTCTGGCGCAAGCTATTCATTAATTAAATC

>V300080312L3C001R0050049908

AAGCGGTCCGTTCATGGTGCTTCTTTATGCATGAATACCAAGT

>V300080312L3C004R0480151100

TGTATAGGGCCGGGCGAGCCGTACAAGGCCGTGACTATATGGC

>V300080312L3C006R0370624712

TACATATATCTGTGCAGACCATGAATCGGCAAGACCGCCCAAA

>V300080312L3C001R0460683924

TTAGCTGATAATAGGCATGGCTGAATCCTGAACATATTTTGGA

>V300080312L3C006R0260545071

GGAGACGAACGAAGAACGCTCAAGGTTATAATAACTGGTAATC

>V300080312L3C004R0470199339

ATTAGGCTGTTATGTCTTAATTCGTTGCTGCTGCATGAATTTG

>V300080312L3C002R0610919981

TCGAAAAGGAGCACGGCATCCACTGCAACCTCACGCTTCTTTT

>V300080312L3C002R0121249347

ATTTTCTTTTCCAGCCTAGGAAACGCCCGCGTACAGAAAGCTA

>V300080312L3C006R0220387875

GTTCAAGAGACACAATCGAAAATCTCGTTTGCAGCAACGTGTC

>V300080312L3C002R0550551464

AACCAGAAACTTGCTGGAGGCAGGCGAAGCCCAGATGTCGATC

>V300080312L3C005R0350617028

GCCCAGATGTCGATCTAGTACGAAAATTCCTCGCCCAGATACC

>V300080312L3C002R0350135094

GTTAATCCAAACTCTCTCGATCATTTGCGACAAATTTACGCAC

>V300080312L3C005R0140148568

GCCCACACTGCAAGCGAAACGAGAATTGCTCGAAGTACGAAAG

>V300080312L3C003R0450054146

AGTACGCTGGATATCATCATCAGCAAAGTATTGCATGCTTACC

>V300080312L3C005R0720379783

ACCCAAGGACGGCGATTTGGGCACTGTTACCATCCAGCGAGTC

>V300080312L3C003R0200009645

CTGTTTTGCCTGAAAAGACTCCTCTGGAACAATACGGCGGGCC

>V300080312L3C005R0690270599

TCTGGAACAATACGGCGGGCCATTCCCAAATGATTTACCTGTC

>V300080312L3C004R0191027565

TACTGAAAAACCCCATCTAGGGTTTGGTCCTCAATACGAGGTA

>V300080312L3C006R0430761868

GGTAATAATGTTGATCCTTTATCATGCCCTTGTTCGTTCTACC

>V300080312L3C006R0170644056

TCTGATTGATGCTATGGGAGTCTTTGGACATGAAGGTCAGCAG

>V300080312L3C005R0190756284

ACAGTCTTTGAGACGTTTGCGCACGACCCGATCACCGAATGGC

>V300080312L3C001R0310737844

CCAACAACTACAATCTAGCACAAATGTTTATCGGGTGGGCGCC

>V300080312L3C002R0071402583

CAACTACAATCTAGCACAAATGTTTATCGGGTGGGCGCCATAT

>V300080312L3C002R0591340189

GCGTCGCAAATGTCGCTCTCGATTCCTGTGAATATCGCAGCCA

>V300080312L3C003R0040861931

AGTATCTGTGACAATATTTGCTTTGACATCTTCCACGGTCAAA

>V300080312L3C001R0130622174

TCATGTGCGGATACTGGTCCATTTCGTCCATTGTAGCCTTCAG

>V300080312L3C003R0080290711

AAGTGTTTGCAAAGTATGGTACGCCTGCGCAAAAGGAAAAATG

>V300080312L3C001R0680784970

GTCGCGAGGGCAACGAGTATGTTATCAACGGCCGTAAATGGTG

>V300080312L3C001R0400152997

CTTTGGAGGCTCGTCGCACCCTGAGCTGACAAAAGCTGTCTGT

>V300080312L3C004R0581064841

GAAGTGCACGAGTCAGTCCGTGAACAAGACGTCTTTATTATCC

>V300080312L3C002R0230580713

CTTCTTTGATTGTCCGTTCGATAATCTCGTCAGTATGCCATTG

>V300080312L3C001R0620596978

AGCGCCAAGTTTTACAGTAGAAATACTATGCATCCAGCATGGT

>V300080312L3C001R0530890400

CGTCCTTGTCGTGCAGAACAGCGCACTTATCCTCATCATGAGA

>V300080312L3C004R0220405847

TATTTCGAAAAGATTTTAAAGGCACCGCCCAAACCGGCACAGG

>V300080312L3C006R0670804788

GGACCCGGTGGCCAACGCTCAAAGTCAGCTTTGGATCCGCAAC

>V300080312L3C005R0160891374

CGAGTGGAAAGGCTCGGGTCCCGTCATGCCATCGATCAATCCT

>V300080312L3C002R0500851981

ACGAAGAGTCAGACAACATTTTCATTTCGTATACCCGGGCATT

>V300080312L3C003R0050342662

AACCGTTCTTTTATACTTTTGAGACAGATGTAAGTCTTCATCG

>V300080312L3C003R0510152587

GATGACGGCGAAGGCGAAGCATTTTACAAGAAGGTTCTCAATC

>V300080312L3C002R0471117864

GAATATCATGGAAAGTGCTCGATAGAAGTCAAGCGTGCACTTG

>V300080312L3C002R0230507342

CATAAAAATTCGACTCTATACGCATCCAGCCGAGGAGGCCGTA

>V300080312L3C005R0221080217

TACCTGAGGATATCAGGAGGCACCGTCAACGCCGATCTACGTG

>V300080312L3C005R0490052604

TGTGAAAAACGCATTGATCGATATCATTTCTGCCATTTTTACT

>V300080312L3C003R0650621110

TATATTAATTGATGTTTTATATCTATCTTGTTAGAACGCAATC

>V300080312L3C006R0420625183

TAGTTCATTGCAGAATGGCATGCAAATTTCATGTGGTATGGAT

>V300080312L3C005R0050714891

TGAACAAGAAGCGGAGTGGATAGGACCACTGGTACAAAATCTG

>V300080312L3C002R0181003503

CGCTACATTACCCGCAACGAGACTTTGCCTGCGCGTGTCCGCC

>V300080312L3C003R0431013282

GGTTTCTTCTGCTTCTTCATCATCGTCCTCTACGTCCCGGCCA

>V300080312L3C004R0021336427

TGATATCATATTCATCCCTTCTTATCGTAATTTTTCAAGTACG

>V300080312L3C006R0260925493

GCAATTTCTGCCAGCTTAGAATTCTGTGTTGTGTGCGTGTGTG

>V300080312L3C001R0100333201

CAGTGCCAGCCTGGTCAGGCTGTACCTGTCTTTGAGCAATTGC

>V300080312L3C004R0211150391

GGGACGTGACATATGTGTCAGAGCAGTATCGTAAATTTCTGTT

>V300080312L3C003R0430599908

TGACGCTACCTGTGGGTGAGTTACCGGCTCCAAAGGGAACAAA

>V300080312L3C006R0520422462

GAACCATCATTGATGAAGTTTAGAGATGTTGAAACTCTGTTCC

>V300080312L3C003R0720657052

GAACGGTCCACAGGTGCTCGCCTTCATCAAGAAGAGCGTCCGA

>V300080312L3C006R0571012767

CCATACATATCAAGTACAACAGCAGCCGCCCTCGAAACAGCGT

>V300080312L3C003R0220541316

ACACGAGCTAATGATGTCGTGTCGCTTCGACTCAGAAAAAGCG

>V300080312L3C003R0381152477

TGGAATAAGACTGTCCATGCCATTTGGTACAAGTACTTTGGGG

>V300080312L3C004R0240644291

TTTGCAAATGCGTCTGGGAATAGGCGGATCCATGTATCATCAC

>V300080312L3C003R0601242104

TCAAGGAAACTTGGAATGGTACCTGGCGTTTCCAACATATACA

>V300080312L3C005R0371106928

TTTTGCAAACAATAAACATACATTATATGGATCATACAAGGCA

>V300080312L3C003R0261361710

TCTTCTGAGGATGGTCTGTGGTCCACTGTCTGCACACTGGAGG

>V300080312L3C003R0330213495

CATTTGCAGTTCCTGCCAAGCGCGCGTAAGCTTGGCTGCTTGA

>V300080312L3C004R0481003110

GTATACATATGCTAATACGTATAAGCAACAACTTAATGTACTT

>V300080312L3C006R0710371272

TCTGCAAGTCTTTAAAGAAGCTTGTCAGCATTTGTAGTAACGA

>V300080312L3C001R0210513597

TATGAAGATCAAGCCCATGTCGATCGAGTGGTGGACAAGCTGG

>V300080312L3C003R0150941119

TGTCACACGAATCACTATTGCTGGAATACGAGTCAAGCATGAC

>V300080312L3C001R0080533585

CGCTGATCACTCGCTTCGGTGCAAACAATGCTGAGATGCATCT

>V300080312L3C001R0401154482

ATGCAACTACCACAGCACATTGAAGCCGTAAGGAAAGCAGGAC

>V300080312L3C004R0271249980

TGCAACAGCAAACTGAACGGTGTGCACTTTGAGCTGACAGGCG

>V300080312L3C003R0680365655

AAAATCAATCACGACTGCAAAAGTATATCTTTTTATTCTACGC

>V300080312L3C001R0110525994

GGTCCAATGTGTGTACCATTGTAGTGGCGTATGACTTGCTCTG

>V300080312L3C002R0371177844

GCAGTAGAACATCGACTTTCTGATCCGACAGGGTCAAGATCGC

>V300080312L3C005R0161363342

CGAATTTCCAAGCTCAGGTTTCCAAAGTCAAACTGTGAAAAGA

>V300080312L3C003R0490622758

TCTTTATTCCGCGATTTTTTATCTAAACAGCGGCCGGAAGATC

>V300080312L3C005R0150997532

AACTTTTTCGCGGATCAAGTCGGGCGGTTTGTTTTCAAATGCC

>V300080312L3C006R0611152299

ACAGCTATGGGAATGCCCATGGTTATGGGACAAAAGCCCATTC

>V300080312L3C002R0480618527

TAATTGGAGACCATATATTGATATTGTTTGTCAAGTGCAAATA

>V300080312L3C004R0350874299

CTGAAGATCAAGCTGAAGAAAAGATTGTCGTGAAGGAGAGCGC

>V300080312L3C005R0651106413

TGGTCAAATTCAAGCAAGAAGCCATCGTGCCCATCGGGTGATT

>V300080312L3C006R0320931042

TTGTGTCCATCGTTGTGTATCAAAAGATTTGCTTCGCGGGGAG

>V300080312L3C005R0130467455

TAGGTTACATTCACGCATATTTCCCACGGGAGGTGACAATGTC

>V300080312L3C004R0220527716

GCAGCATACGATGAAGAATTTAGTAGGATCGAAAGCTTTCTTT

>V300080312L3C006R0441220387

CGCGAACGTTTCTGAAGAACACTTGCTGAAACTCTACGATGCT

>V300080312L3C003R0650050729

TGAACGAGAGAGAAAAATGCCCATCTATGTGAGAAAAAGAGAC

>V300080312L3C006R0250966877

GCAACTGATCAAATTGTGCAATATCGATGTTGCTTGTCTTGGG

>V300080312L3C003R0040371609

CCAAATTGATCCAGCAGTAACATTGGTATATACGGCTCGATAT

>V300080312L3C006R0390207743

GGAAATTTTCAGGCACATTGACACCCAATTGCTGCATAATAAA

>V300080312L3C005R0230769787

TCTGGGTTTATAATAGCTAGCAGAATCTTACAGAGCGCACGCG

>V300080312L3C005R0490081504

TACTGCCGACTCAACATTGGAGAAGTTGACAAAAGCACACTCT

>V300080312L3C005R0561114325

CCCAGTTCATATTGACCAAGACTTGGTGGCGGTGGCTCAACCG

>V300080312L3C005R0160607038

ACCAGCAACAGTGTATGCACGGTTCCGAGAGACAGTCTCTGTA

>V300080312L3C003R0261326620

CCAGTGGTAGGACTTTCGCTAGCACATCTAAGTATGAAAGCAC

>V300080312L3C004R0610217948

TAGTTGCTAATTCGATGTTTGTTCAGCATGTCTGCCGAGGAAG

>V300080312L3C001R0540754227

AGGCTTTGGACAGGTAAATTGTCAATGACCCGGTAATTGATTG

>V300080312L3C001R0310657236

GATAACATCCACGGACAGCACTTATGCAGCTCGCTTTTCTGCA

>V300080312L3C005R0570322673

GGTACTGGTCCATGCGAGGAAGGTGTGGGACCGACTTGCGAGC

>V300080312L3C004R0500111409

TGTGAATTATTTTGGATCTGATAGCGATTTTGTACTTTCGAGG

>V300080312L3C006R0480239378

AATTGCAAGCGACAGATCGAGCTCTTCGCCAGTTGCAATGCAC

>V300080312L3C001R0310013413

TGAATTTGTAGCTGCCTCGTCAACCGCAGCTATCTTTTCCTTC

>V300080312L3C006R0210272661

CGAATTTCTCCAGGATACGAGAAGTCGGTGTACTTCCACATGT

>V300080312L3C005R0190257897

TAGCATTGGGTTAGGCTGCGTACACTACTTTTGTAAGATCTCC

>V300080312L3C006R0431039343

CCATTCCTGGGTTCCAGCTTGGAATATCAATGCAATATGGAAA

>V300080312L3C002R0450197411

AGGATATTCCAGACACGTATCTTTGGATCTTCTCCGGCCACAA

>V300080312L3C003R0691064860

AAGATTTGATGTTCTAGCTTACCATATCATTATTTCTGTAAAA

>V300080312L3C001R0580481804

TACTACCGCATAGATATCGGCACTGCCCATCAAGCCATCTTGC

>V300080312L3C005R0480296875

AAATGCTTTCAAAACATTTTCAACTTTTTACCCGTTTACATTC

>V300080312L3C005R0280194348

AGCGATTGCCCGCGCTAAGCGGGATTCCGATATTTGGTGTTGC

>V300080312L3C001R0540043503

TCAACCTGATCACGAATACTTCTTGCAACAAGCGGTACAAAAG

>V300080312L3C004R0320747905

ATAATCACGCGGTCTCTAAGCGAGCGTCCTCGATAGAATGGGT

>V300080312L3C002R0700452898

CCGCATATGACGATGGTGCATTTCTACGTGGAAGTTGACAATC

>V300080312L3C002R0520936434

CGCCGGACTTCTCGATGGCGAAGCACTGTACAACGCGCTGCGT

>V300080312L3C001R0610050576

CCGCCATCAAGGATCCACACAAAATCCGACAGTGTTGTCTGGG

>V300080312L3C001R0610597368

CCACTCCGGCATTTCTATTGGCCTCGCGCATCTCGTTTTTAAG

>V300080312L3C003R0341041812

CCAACGAGGTCTTTGGTATTTGCAATGGTTCACCCACTCGCAT

>V300080312L3C005R0320595596

GTTATTCTCCTCAACAAGCATGAGCTGGTCAGTTCTGATGATC

>V300080312L3C003R0060311655

ACACTGACGGCTGCTCATCGTCACTAGAAGAAAAGACAAGATT

>V300080312L3C004R0310289572

TTCCTTGGCAGTTACGTGGATACCGTTGCGGGCAATGACAGGG

>V300080312L3C005R0391055076

TACGGCATTACGGATTACAAGTCCATTGTGCGACTTGCAAGCA

>V300080312L3C004R0601397343

GTCGCCCTTCCTGCTGCATTGTGCAAGGATAAAACAACGCTAT

>V300080312L3C003R0340840074

ACTATTGCATAAACTGTATGGATAATCCGCTGTGCTCATTGAT

>V300080312L3C004R0160796645

CAGACGACAGGAAAACTTTGAACAATTTGACGTCAGTAACTAT

>V300080312L3C003R0360711598

TCATGCATACATTGATGGATATGGAAAACAAAACTTTGAAATT

>V300080312L3C004R0040661477

CTCCTCTTCACGAGAAGATTTTCGGCGACTAAGTGCTGGGAAC

>V300080312L3C001R0070499902

CCTCTTCACGAGAAGATTTTCGGCGACTAAGTGCTGGGAACTC

>V300080312L3C003R0351231339

CACGCAGTCGTTTTCTTACTTTGTAGTCACCGATGGAACACCC

>V300080312L3C001R0291362888

ACCAGGTTGGTCCGTTCATTGTGACACCAATAATAGCTTACGG

>V300080312L3C003R0410415856

CCACCAACTTTTTGCACCAGCAGAAAGATAAAGATTGACTATC

>V300080312L3C002R0070042870

AATTTGGCGACGTTTAATAGGACTAGCTAATCCATCAGACAAT

>V300080312L3C004R0680081778

ACTATCGACCATTGCGTTAGATCGCACCCTTTAGTATGCCAGA

>V300080312L3C005R0290270439

ACCCTTTCCAAGCAAGCTTTTGCATTTACAGGTACTTACTCCT

>V300080312L3C003R0550533649

ACGCGAGTGAGCCAGCACGTCAGTGTAAATATTTCATTTTCAT

>V300080312L3C006R0080930084

CGCGTGATGACGCATGAAGCTACGTGAAGACCGCTGACGTGTT

>V300080312L3C005R0580354226

TGAATTTCTTCAAAACTTGAACATACCTTGAAAGCTCTGAATT

>V300080312L3C001R0700774687

GCAAAAATTTGATTCAAGCATGTCTGAACGCGCCTGATCCAGT

>V300080312L3C005R0650517096

GCTCCGCAGACCCAGCAGCAGTATGGTCACATGCCTGCTCCGT

>V300080312L3C005R0200331091

ACCAAGGAACAATTTTTCTCTGTATATTTGTTCCCAGCAATAA

>V300080312L3C001R0620320428

GATAGCGGCAATATGACGGGGACAGAGGATACCAGGCCCACTG

>V300080312L3C002R0230789205

CTGAGGATGGTCTCTTGGACGATCACTACCGTGCCGCTGCCGA

>V300080312L3C002R0500009135

AAAATCCGGCCGATGAGCATCATGGAAAGGTTGGCGTTTGACG

>V300080312L3C003R0340089947

AGGAAACAAGAGATTGAATAAATGCTGTCCAATCCGCTGGTTT

>V300080312L3C002R0010035670

TACGCCGATAATCATGCCGCGGAGCGAGGGATCCGCCAGCGGA

>V300080312L3C006R0340679022

CTTTACGGTATCCAAGAGTCCATCTGCAAGCTGCGTAAAAATC

>V300080312L3C004R0241123419

TGCTGCTCTCTGCAGCCGTTGCAACTTCTCCCGCACGCACGTA

>V300080312L3C006R0670156255

TAAACTCTTGGTCGCTCAAGTCGTGCAGAACCGCAAACTGTGT

>V300080312L3C001R0040855518

TGAAAAGAACAAGCTGCGGCTGTCGGGCACACCAGTCGAGTTA

>V300080312L3C004R0250442404

CCAGCCGTCGCATTCAAAGCATCAACGCAGAACCAGCGCAAAA

>V300080312L3C001R0530826440

AGATAAAAACATCAAACTGCTCGACTGCCCCGGTATCGTTTTT

>V300080312L3C005R0210459050

GGAATTCCTGACGTGCAACTGGTAGCGCGCAGTGTCCTGCAGG

>V300080312L3C001R0370487265

ATTATTTACATTTTTGCGGTAATCTTCTCCAAGTAATTCCATG

>V300080312L3C004R0410686889

GGCTCCAGCTCCAGGACCTTGAGCCGCGACGTTTTGTACGCTA

>V300080312L3C001R0030670983

TAACTTGATATACTTTGCGTTCTTCAGGCAAGTCCTGGGTGAA

>V300080312L3C004R0580527188

AAGTTTTGAGGCGCAGAGGACGACGTGATCGCAAAGAGTTGCT

>V300080312L3C006R0420733181

CTCGTACAGGTGGCCTCGCAGAGAGGATGTCTTGTAGTTTGCA

>V300080312L3C002R0170345230

TGTGATGTATGTATGGTATTTATCAAACTTCCCGCAAAATTCC

>V300080312L3C002R0400737087

GGTGCATCCCAGCTTTCTAAAAGAGACGGTCGTACGTTCGGCA

>V300080312L3C006R0680609070

ACGCCCTACTACGTCGGTCTCTTGCTGCGAGCTCGTCGTCCCT

>V300080312L3C001R0281043157

TCTTTTTGTTGCAACGATCGTTTGCCAAATAATTCTGCGTAAA

>V300080312L3C006R0220812155

AACGGCCCAGGAGGGTGTTTCCCCAGTAAGCAATTCAGCGTCT

>V300080312L3C001R0390898894

TGCATTGGAAAGATTCTGCGAAGCTTCTTGGGTATCCTTAATC

>V300080312L3C005R0550206075

TCCATACCGAACATTATTGTGGTCCTGGAACCATCGAATATGA

>V300080312L3C006R0230009657

ATCGAAAGGATCATTCAATTCAGTGTAAGCATTGCAGACTTCC

>V300080312L3C002R0430015244

GTCACCAGGAGGGAACTTGACGTTCAACTTTTCTTCAAGGGTG

>V300080312L3C004R0380303200

AACTTGTCGCGGACATTGTTGTTCATGATAAGATCAAGGTATC

>V300080312L3C006R0361016234

ATTCAAAGTATTGCTACAATAAGGCGTTAGCGACTGATTGTCG

>V300080312L3C004R0201243820

ATCACGTTTACAGTAGCAGTACTGATGCTCAAGGTTTACGGTA

>V300080312L3C001R0350482741

GCACCAGCGAAACATTGTTTCTCTGTCAGGAGCATGTTAGGTA

>V300080312L3C002R0251142473

CTCTCGCAAATCTAAATATATCTACAAAGTACCCAGTAATGTG

>V300080312L3C003R0221356451

CCGATCTCCTTTCAGTGGGGCGACTGCGGATGACATGTACACG

>V300080312L3C005R0620978123

AGCCAACCACGGTCGACGTTGACAACAGCAACTAGCATTCCCT

>V300080312L3C001R0350157513

GGAGGAAGAGGTGATCAAACTATTCTCTTCTGAACAGCGCCTT

>V300080312L3C005R0531157477

TATGCATTGATACTTTCACCAACTCGCGATCTATGCGTACAAA

>V300080312L3C003R0330771694

TTGTTGTCCTCGATGAAGCTAGCGTACTCCTGGGTGCCGGATT

>V300080312L3C004R0710062112

GCCAAGAGACGGAGAAACTAAGCCGTAAATTGATACAGCCTAT

>V300080312L3C001R0010411689

AGGTATCTAATAAGCATCGAATTATGGTTTCGAATGCAAAGCC

>V300080312L3C004R0630485988

TCTTGCCAACTGAACGAGTAGCTCCTTTCTGCTTTCGAGAGCT

>V300080312L3C002R0560672321

AATTCTTCCTTCTCCAGGTCATACAGACTTCGCTCTGGCTGAG

>V300080312L3C001R0110461753

CAAACCATTCGGATCCTGCGAACGTTGCTGTGAATTGGGTGGA

>V300080312L3C004R0530610720

TAATGCGCTTCCGCCTAGTTTCTCATTATAGGCTTATTTAGAT

>V300080312L3C001R0530173976

CAATGCATTGCTTCGCCTCGTAATTAGAACGGTCATATCTTTC

>V300080312L3C004R0180217775

CAGACAGACGCTAAATTTAATCTACCGTACAACCCCACTCGAG

>V300080312L3C006R0050429535

AAATTAACATTGCGTTTCTCGGCTTGATTACCGGCTGGATCAG

>V300080312L3C003R0591031971

AAAGGTATCTCGTTTTCTGGTTGCGCGATTCAGTGTCTTTTGC

>V300080312L3C005R0190902117

CGTCGCTGGTTCGCCTTGAGTTCAAAGGCTTCGATCCAATTGG

>V300080312L3C002R0090419689

GAACGTTCATATCATCTGATACGTTACTGAACGCAATGCATGC

>V300080312L3C003R0500843141

GAATGAGCTGTATAAGGATAGTTGGTGTTAAATGGGTTGCTGG

>V300080312L3C003R0350606738

GACCTTCCCCGCTGACGCGCCCCACTGGGCATCGGCAGGGGAC

>V300080312L3C005R0580472254

GATGATAGCATCCTCGGCGTCTGTCACTTCTGGTATCGGCACT

>V300080312L3C001R0260719925

GTGACACCAGACGCCGCCTACTTGCCACATAATATATTCGACA

>V300080312L3C002R0650548399

GAAACGACGACGACGAACCAAGAGAAAACGGTGCTGTGTATTC

>V300080312L3C004R0250462036

TCTTGCTTCGTTCCCAATTCAGCTTCGATGTCACACAGTGTTC

>V300080312L3C004R0240789138

TCCACTTGGCGTTCTTGAAGCATTTTCTTCAGTACAGGATCTT

>V300080312L3C004R0421236076

TGTGGTAGATGGCTTTGCGGGTGCAGTTGGAGTGACTTTTGGA

>V300080312L3C005R0220534124

CTTTCTGTTGAGTTGCACCACCAAATGAGAAAGTAGGTGCTGC

>V300080312L3C003R0390802558

ACTAGTCGCAGCAGTTGTAGCTGATGGTTTTGCAGTTTCCGTT

>V300080312L3C003R0020471292

AAGAAGTGGTACCAAAGGTTGGAGTAGTTCCGGCAGGAGCAGC

>V300080312L3C002R0051146905

GATCATGGATCCTTCAGGTAACAGCCATGTTGCCCATTCTCCA

>V300080312L3C002R0340930875

GTAAGGGTCGTGGAAGTACATGTCGTCCTCGCTTTCGCGGATT

>V300080312L3C002R0290065783

AGTAGAGTAGAGCGAAGGCGGGCTTCAACGCGTTCCCTTCAAT

>V300080312L3C005R0190113521

AATAGTGTTCGAAAGTTTGTGATTTATCAATTGCAATATAGTC

>V300080312L3C006R0431083721

GGTTGACAAAGAGGACGTACAAATGGAAGGGCGGGGAGCGGTC

>V300080312L3C004R0360370965

CACTGTTAAACCTATCATCCAACCACTTGTTTTCTTGTAACAG

>V300080312L3C006R0410373019

AGCAATTCAGGCAGCTATTGCGAGTCAAAACACCTCGCAGGGC

>V300080312L3C004R0101205448

GGGTTTGTTCGGCACCAATAGCAGTAGTAACAATGCACAGCTT

>V300080312L3C002R0260807515

CACTGAACCTTTATTGCTTATTATTCAGCGTGATAAGAGAAGT

>V300080312L3C001R0170490716

CCATCCTTGAGAGCCAGACGAGTCAGTCCCATTCGTCGTCGGT

>V300080312L3C001R0300983225

CCTTGAGAGCCAGACGAGTCAGTCCCATTCGTCGTCGGTAAGC

>V300080312L3C004R0080765602

GACACTTCGGTGCTGTGACTGGTCTCCACTTCCATCCCCTGTT

>V300080312L3C002R0131104118

TTTGGCGTGCAAAGGTAAGAAATAATGGGCAAGAGAGCGGGTG

>V300080312L3C002R0700370049

AAAATGGTCGCCATCGCATCCAGCACTTTTCGGATCAGTTGAC

>V300080312L3C002R0110222428

GTTGCGGCAAAGCGACGCGGAGCATAGCATCCCGTGCTTAAAG

>V300080312L3C003R0180999026

AAAATGATGCTATCCGCCAATGCGGATTACGATCACGGAAAAC

>V300080312L3C003R0231352930

GGATGGTGCGTCCCATCGGATTGAGCATGTAAAATTCTGCTGA

>V300080312L3C003R0540493798

TTTGTGTGTTACCTTGGCATATTGGAATTTGAATTCCTGCGTA

>V300080312L3C002R0261165305

TGGAAAAATCCATTCTGATCATAAGTTTTGCTAGAAAAAGTGT

>V300080312L3C004R0421025271

GCGTCAGCTTCGATCTGGAGTTATCCATTTCCACTTTGAGTTG

>V300080312L3C005R0390611522

GGGTGCTGGTAATACTTCGCTTGTCAAAATGCGCATTCGAATT

>V300080312L3C001R0040403529

TTGGCCGTAACAGCGGCTTCCAACAGGATCCGCAGTGTAATTG

>V300080312L3C002R0330508867

CAGTTAAAATTATGGAGGACGATGTAGCATGCGATATCATCAA

>V300080312L3C006R0620624124

TGTAACAACTGCGCCAAAGAGAAGAAAGTGTGCGCCAAGTGCC

>V300080312L3C001R0110224145

TAACCAAGTCTATCGCATAAGATATTTCCAACGTGATGCCAAC

>V300080312L3C006R0220471100

TCAGAGTTGCGTGCTGTTAACGAGAACTGTATCCATATGAGTA

>V300080312L3C003R0441130095

GAGATGGTGCTTTTGATCTGCGGAAGATCCATTTCTTCTTGAA

>V300080312L3C004R0300373893

AAATCCTTCTTTCGTTGCTCTGCTTCTCTCTTCACGTCATCTG

>V300080312L3C004R0700268093

GCTTTTTCCATTGCAGCAAGAATCAGAAGAAGCGGCAAAAGTA

>V300080312L3C001R0160471015

CGACATCATCTAAACCGCCTGCACCAAAGCCTAATTACAAGCC

>V300080312L3C006R0270506876

AAGTGATGATTCGTTAGAAATTATGTCATGGGTTCAAAGGAGG

>V300080312L3C005R0650129982

CGCACAGAGACACGGCAGCAGCAGCAGAGTTATTATCAAGGTT

>V300080312L3C004R0270295765

GCGCATGATGGCTGGATACGGATCGATCGAATCGGCGGGAGCC

>V300080312L3C003R0561365172

CGCGATACGAAGCAACGGCAGCTGCAGCCAGGAGGCATCAGTT

>V300080312L3C003R0250913103

AGGTAAGTTTGGTCCTGCCTGCGTTTCTCGGATTTTTGAAAAC

>V300080312L3C006R0570451894

CTGACTGCGTTTCCGGATTTTTGAAAACATAACAATCGTACCC

>V300080312L3C002R0030213641

CTTCCAGTGCGGTATCCTCCTTCCAATGCTATCCCTTCATGCA

>V300080312L3C005R0060590949

CATCAATTAGACTTTTGCACGTACTATTGAATCTGTCAATGTT

>V300080312L3C005R0510348060

AAATGTTGGAGAAATCAGGTGATTATGTTTGTTTCGCATAAAT

>V300080312L3C006R0390229017

TGCAGGTCAAGTTCTCGAAGTCCAGGGCAAGAAAGCCATCGTT

>V300080312L3C003R0681357487

ATGATCCTACGCAAGTATATCCTTTGTCATCACGCTGCTTATA

>V300080312L3C001R0140669500

TCATTACTCCACGTTTGGCATTGACCACTGCCGAGTACTATGC

>V300080312L3C003R0060158605

TTCTTGGAGAAGTTTGAGAGAACCTTCATTGCGCAGGGACCCT

>V300080312L3C006R0680159123

AGGGAGTTTTTGATACCGCCGATGAAGAGCAGGATGAGTAGAT

>V300080312L3C003R0211229347

GTTGTAGTGTAGCATGTGCGCATGTTGTGTTTGTATCCCCTTT

>V300080312L3C001R0650946890

AGGGAGCAGCGTTAAGGAGAGAGAGGTGCGCTTGTCTTTATTT

>V300080312L3C006R0391283293

AGCAAGGTCGGCAGTCTAGATAACATCCACTATAAGCGGCGTT

>V300080312L3C002R0540126923

TACGAATGACGGTGCTGAGGAGAAAGAAGAAAAAGAAAAGATC

>V300080312L3C005R0440561652

CGATGATTTTGTAGATATTCCCTGAGTTCACTGTACCATGCAT

>V300080312L3C004R0160600372

CGTCGTGCCCGTATCGACAATGCCCTCGCCACCAACTTGGATG

>V300080312L3C002R0071043540

TTGGATGGATGCGTTTTGTATAGTGATCGTGTCCATGCTAATT

>V300080312L3C005R0281303941

TTGAACCTTGACCTTGACAGTCTCGGATGCATCGAAAAATGAG

>V300080312L3C001R0140143804

CTCCTTTGCACCGCCTATTACATAACGCCTTGGATCCTCATTT

>V300080312L3C004R0041371302

AAACCACGTATGTAGAAATGCGTGTACAGAGCACGTAAGAATG

>V300080312L3C001R0120732814

ACGTCCTTCTTCCACGAAATCATGCTTCCGAATTTGGCGTCAA

>V300080312L3C002R0060893128

TCGTCTTTCATGGGTATCCAGGCGTGCTGCTCATCAAGCATGG

>V300080312L3C003R0410285869

TCCAGGCGTGCTGCTCATCAAGCATGGCAGCTTCACGCCAGGG

>V300080312L3C006R0470576513

GCAGAGACGGAATTCGATTTGCGTTTCTCGGTGGTAACAGATA

>V300080312L3C001R0591196196

AGCATCAAGAACCAGGGACAAAGGGGAGGAGAAGGTGAGGGAA

>V300080312L3C005R0291119802

GGAGGAGAAGGTGAGGGAATGGCGGCGGAAGGGGAGGGGCGGG

>V300080312L3C003R0051103863

AAAAGGCGAGGATGTCGACTCGCGCTTTTTGAGCATGTGAACA

>V300080312L3C002R0350148149

CAACAACGACCTCATCTGCAGAGCCATTGAAACAAGTCGCATA

>V300080312L3C005R0571403973

TTCTATCATCATGTCCTTGATTTCTTCAGGCGTCAGGTTTTCC

>V300080312L3C003R0630754500

GAGAACTCGTTAAAGTCGATCACTCCGTCCTTGTCCTTGTCAG

>V300080312L3C002R0300956455

TCCTTGTCAGCCTCTTGGATCATTCGATTCGCTTCTTCTTTGG

>V300080312L3C004R0550352172

CTTGTCAGCCTCTTGGATCATTCGATTCGCTTCTTCTTTGGAA

>V300080312L3C003R0290866386

GAGATTGCTTAATTGACCTATATTATAAAAACGTCGATCGGGT

>V300080312L3C005R0100435809

CGTTCCGTGGTTATCGATGTGCCTATCTTCCATCGTCCTCTCC

>V300080312L3C003R0570073683

ATGGAAGTCTGAAAATTTGGAGCTAATTGAATATGCAAGGAAG

>V300080312L3C001R0150015739

TTTTGGTTAATGCATATGTTGCCGATAAGTTTGCCTGTCGTCC

>V300080312L3C002R0381252073

TTTGCAGCATAGTGCCAGCGATGTTCCCATATTCTTTTGTGCC

>V300080312L3C001R0630326375

TTCCGAAAAGTTCAAGCAGTTGCTATCGGCAACGACTTTGCCA

>V300080312L3C001R0620686982

TCCAATATATGCAAAGGCATAAGTGTGGGAACCATGATAGAAG

>V300080312L3C005R0140564541

CAATTCGCGCAAAGACCAACTATTCTGCATCGATTGATTTTGG

>V300080312L3C004R0241226877

GCGCAAAGACCAACTATTCTGCATCGATTGATTTTGGAGGATA

>V300080312L3C002R0720936336

TTCTTCAGCGAGCACGTCGGATACGCTGCCTTGGAGAATGCAT

>V300080312L3C005R0600740547

CATTGTTGTTGACCGGACTCTTTGATGAATCCGGGATTCCTCG

>V300080312L3C006R0420900106

GCCAAAGATGGCCAGTCTTATGGATCTCGTTATAAAGGGAACG

>V300080312L3C001R0041342920

CAAGGATCCTGCGTACCCAGAGTAAGGACCAGTTCACTGGATG

>V300080312L3C001R0280845203

CAACTGAGCCGGCGCGCTCTTCAGCTACCCGGCAAGCTAGATC

>V300080312L3C005R0620407946

GTTTTCTAGTTCTCGGTAAATTACTCCCACACTATTGGTATTT

>V300080312L3C006R0231071067

TATTTCCGTAGCATCATGAAATTGGTGATACTCACATTGATTC

>V300080312L3C006R0701392654

AGTGTACGTTGCAAATCCAAGAAGATCTTTTAGCTGACAACAA

>V300080312L3C001R0160231769

CCTCAGCTCGCGGAACACAGACCTCAACTTTCTGCGATGCTTC

>V300080312L3C003R0341005509

ATACTATTGATTTTACTGACTCCGTCACAGATGTGGAAACTCT

>V300080312L3C002R0020242062

CCAAAATGTTGAAATCGGTCGATTTTCAAAAGCATTGCAAGAT

>V300080312L3C006R0360334026

TACGTAATATCAAATACATCTATGCATATATTGCTAACTTATA

>V300080312L3C004R0600204545

CAGGTATTGTCCAGTCAAATAGTATTCCTGAAGAAAATTTCAG

>V300080312L3C002R0670290171

TGATGCTATCACCGATGTTGTGGAGAAAACTGCCACGATCGGT

>V300080312L3C005R0011354869

TCCGGACATTAAGCGGTCACGAAGGACCCGTAGATATCGTCAG

>V300080312L3C002R0281346689

AACTGCGGCCGTTGCTCCAAAGGCATATCCAGCCACTGCAACG

>V300080312L3C003R0300647467

CACATGATCACTGCAGATTTTCGCCCTTGGTTCCTGCAGGTTG

>V300080312L3C006R0320540866

CTGAAGTTCAGGACCGCAGCGAGATCTCTGTTTTATAACCTCC

>V300080312L3C002R0700071278

GTTATCGAGAGAATACAGACCGAAGCAGACCATGACATATCAC

>V300080312L3C003R0570114480

AATTAGGACAGAGAATCGATGTTTTAAATTCATCGATTAAATA

>V300080312L3C004R0580808307

CATTATGGCGCTGACCTCGCATCTAGTTTTCATGTAAAGCAGA

>V300080312L3C001R0390557271

CATCTTGTCCAAATCGTGAATTTATGTGAAATCACTCTTCGTA

>V300080312L3C003R0120042694

ATATCATTCATTTAAAACTTTAAAAAATACTATATTTCTTTCC

>V300080312L3C003R0201012230

ACGCGTAGAGTCAAGTCGATGCGAGCCTCGTAACGCTGCAACA

>V300080312L3C002R0160123859

GGTTTGAGCAATAAGTTGCTAACGCTGGCAATTTTGCTTTGCC

>V300080312L3C005R0300363226

CGAGCGACGGGTCGCTAACACGCGATGCACCATCGAGAAGGCG

>V300080312L3C001R0540464778

CATCATGCGGTTGAGTAGACCGTGGATGACTTCGAAGCCGGAT

>V300080312L3C004R0580860284

TTCTGTGAAACCGGCAAGTGCAAGTTCGCGTCTAGCATGATCT

>V300080312L3C006R0360223831

GGACAATGTTGGCCTTATGTTCGTCAGTAGCGGTGATTTCGAT

>V300080312L3C006R0210722370

AGCTTATCCTGCAAGTCAATGAAGCTGTCGTAGCTCTCCTGCG

>V300080312L3C001R0430860068

TCGGGTTTCACGATAACCTGCTCCAGCTTGCCGCTGGCAGGCT

>V300080312L3C006R0240386936

TGGAATTGATGACAATCGGGTAAGCAAAACCGCATATACCACT

>V300080312L3C001R0670592345

CCTTTGCAATTCTTACGCCAACTCTATTTTTGCCTATGCGCAA

>V300080312L3C003R0361346361

TTACATATCTGGTGACTGTCTTTGCATATCTTACTATGAGCGT

>V300080312L3C002R0190601584

GCTAAATCCGATCAACTTGACAGGCGAGCTTTGGTTACTTGGA

>V300080312L3C003R0360061558

CATTTTCCCGTTGGCCTGCTATCGCCGACTGTTCGGTCAATCG

>V300080312L3C001R0420891023

CAGGCCTTGGCTGAACGCGAGCGAATGCTTGAAGAGAAAGAAC

>V300080312L3C002R0050737167

AGAATATCAAGAGGAGGAAGACGCTTTCGATGAGGGCGATGTC

>V300080312L3C004R0140509114

AGAAAGGTACGACTTTTACAATGTAATAAAAACAATGTGAGCT

>V300080312L3C006R0110932135

GTAGCACGGGCTCGTGCACGCGCCTCCAAATTTCACATCATCA

>V300080312L3C002R0390177066

GGAACTATGGTAACCACGTGGCATCGAACTGATTAGCTTCGCT

>V300080312L3C004R0010484913

GAGAGTTCACATAGACCAAAGCGGCAATGTGAACAGCAAACTG

>V300080312L3C002R0611071414

CCTTCACAAGTTCGTCGTGAGGAACACTTGGTCTTTGCGTTGC

>V300080312L3C004R0340728371

AAAATTGTGCCAATCGTTACAAAGGTCCATTCCTCAGATCCCA

>V300080312L3C001R0500319972

ACCCGATGAACTCCTAACGGATTTTCTTCGTCGTATTCTCTGT

>V300080312L3C005R0370896776

CTCTTGTTTGATAAACAATTGATAAGTGTACATACTCAGGTTG

>V300080312L3C005R0101339548

CGCCCATTCGCTGACGCAAAAAAGGCAACAACAAAGTGACAAA

>V300080312L3C003R0380640409

ATGATCTCGGCAATTCAATGGATCAAGAAGGGCGTTGCCGCCC

>V300080312L3C005R0650150776

GAAAATGAAAAGAAACAAGGTATGCTCAGTTTGGCTCCTGATA

>V300080312L3C001R0300397497

TTGCCAAGAACTTTATCAAGGATGCTTGGTTCAAGGCTCTCAT

>V300080312L3C005R0510564811

GCTCTGATATTATCCACTACGAAAAGAACTACAAGCTCAAGCG

>V300080312L3C004R0271334714

TCAGAAGCATCTTTACAAATCTTAATGGTTTCAATATATTTTC

>V300080312L3C006R0070688607

CATGTTGGTAAAACTCTTCAGCTAGAAGCAACCCCTTTGGTTT

>V300080312L3C003R0420458384

AAAGTCCCTAGAAACCCGTTGTTCAGCACTAGTCTTGCCAGCT

>V300080312L3C003R0510093830

ATATTGCAAAAGCAATAGCACCTGATCAAGGCAATTAGACGCC

>V300080312L3C002R0271324447

TCAAGGATTTGCAATTGAGGGCAGCTTCCAGCGATTGTCAGCA

>V300080312L3C002R0100234124

ATTGTGGTATCGTAAATTTTCAATGAACTTTGACGATTGACAT

>V300080312L3C004R0601378818

ACCGAGGCGTTCCTGCGATTCCACGGAAAGCCGACGATTATCT

>V300080312L3C001R0721359361

TGAATACATGTTCTCATTCTTGCAAATCTTGACTGCCATGGTC

>V300080312L3C001R0681292159

TCGCTCCTTCCGTCGCTTCACCACTGCCGTCTCTGAGGTCTCT

>V300080312L3C003R0340611507

AACATTGTCAGTTTGCTTGCGACTGGTGCCAACGCGTACAACC

>V300080312L3C001R0430859168

GCGACGTTTGTGGAATGAATGATCTCGCGCAAGGTGTGAAAAC

>V300080312L3C003R0420507726

CATTTTCATCTGGTCCGATCACCCTTGGCTATGCAGCATTTGC

>V300080312L3C005R0220601299

TTTGCCACAGGCGATCCAAGAATAAAATAATAGCATATCCTTG

>V300080312L3C003R0661243755

AAGGTCTGCAGTGGCGACATCGTAGATATATGTTGCCCGGTTG

>V300080312L3C002R0020788711

CGAAATAGTTCGAGCCAGGACCGATGCTGGGTGGAGGCGGATG

>V300080312L3C002R0280274078

ATAAAAGAAGCAAGGGCCGCCGGTCCTCGCCTCTTCTCCATCC

>V300080312L3C005R0611361740

AGTGGGTGCTGAAATATGTTGTCACTCGTCATATCTTAACACG

>V300080312L3C002R0211274893

TGAGATTGCAGATCAAGAGAGGTCATACACAGCGGAAACAAGG

>V300080312L3C003R0231391270

AGCTGAAAAAAGATGATGAACGATTTCATGAAAAGGGCCTCGG

>V300080312L3C005R0510183183

AAATGGTCATGGTGCCAGGACAAAGTTATTACTGTACCATCTA

>V300080312L3C005R0060709250

TCCTTTTTCAGCGCCTAATCTGTCACTCTGAGCTGGGCACGTG

>V300080312L3C001R0120222069

TTGGCCCACTGTTTCCAACCATTCTGTCAGTAGCTCGACAGTG

>V300080312L3C005R0211304997

CTAGCAACAGCAATGGCTTGCACCATTTTCATGGGCTGGATGT

>V300080312L3C002R0160233445

GTTTCGCTTCAGGATATGAACGCGTATCCTTATAGCTACTTAC

>V300080312L3C002R0021400816

TTAGCAAAGATATAACTTGTGGTGGACGAAGCAAGCCATACCT

>V300080312L3C002R0460654604

CATTCCGGTGCTGACTTTGAAGGAAAGATCTTTCAACTGTGCA

>V300080312L3C005R0100478962

TCAAGCGACCAACGGCTTGCGACAAGTTGATGAGGTAGCTTGC

>V300080312L3C002R0331023710

CTACTTCCTTCTCTCGTTCACCGCCACCATAGAACGCGATTTC

>V300080312L3C005R0701085655

AGTAATGATGCGAGACTAAGTTGCAACGTTAGAGGTCACGATC

>V300080312L3C002R0050652425

GCCTCGTTACCGCTATATCAATTCCATCAGTTTCTGGCCATCG

>V300080312L3C004R0090606081

TGGCTCTAGCAATAAAAGCTTCACGGTTTCTTGAAACGATTGC

>V300080312L3C001R0360170991

CAGCTGGAATGTATATGCTTTATACTTGCAGCAAACATACCTG

>V300080312L3C005R0651037487

CCTCTTCCGCTGTCGACAAGGTAATAGACGCATACATCCATCC

>V300080312L3C005R0440074671

TACTGCTAGCTCGAGGGGATGCAATAGATGAAGAAGTTGCTGT

>V300080312L3C006R0600918142

CGTTATCCTTGTCTTTGTCTAAATAATCTTCAACGGCAACGAT

>V300080312L3C003R0690439716

TATAACACGATTCAGCAGTGAGCTTTCCATGTTGATAAAGAAG

>V300080312L3C006R0510890753

GACAACGATGAAGAGGTGCCGTATGTACAATATCTTCCTTTCC

>V300080312L3C002R0010758996

ATTTTCGTCTGATTCGGCCTTGTTAAACACAAGCGGCAAAGCT

>V300080312L3C003R0270144374

GAGGCATTTTTCACCACTTCCGGCAAGGAAGATTGCGGTTGTT

>V300080312L3C001R0640185587

AGATGATGTATTAAATTACAAAGGTTTAAGGAGAAGAGGCATA

>V300080312L3C003R0290944263

CCGATGCACTGACGGTTGCCTGCCAAAAATGGAGCATACTCGT

>V300080312L3C001R0461097979

GGGTATGTTGGCAGTTGAGATATTTGAATGGTCCAAAGGATCT

>V300080312L3C006R0270010952

CCCGGCTGCTAGGAACGTCAAGCATTGATTCTGGAGATCCTTG

>V300080312L3C001R0090569676

AGACCGCGCATGTGAGGACAAGCAGGAAGTGGATTGGGGGAAG

>V300080312L3C006R0561112071

GGCTCGCCGCCCACGAGCCAAGGGTCGTCGTCGGAGTACATGA

>V300080312L3C004R0370565064

CAATGGCCGCGGCGGCAAGGGTTCAGTCTCCAACTCTGTCTTG

>V300080312L3C006R0020503383

AAGTTGCTTTGTAAGGAGCCTGTCAAGGAGACATTGTAATACC

>V300080312L3C001R0710518779

GCTTGCAGAGCGCGGAATTTCAACCCGAATTCTAACAACTTTT

>V300080312L3C003R0121377651

AGCTGCAGACGTTTCTCATCTCGGCAGCTCGTCCTCCAGATAA

>V300080312L3C006R0620650652

TACGTCGTCTCATATGTTTTAGTGCAATTGCAAAGCGCTCTGA

>V300080312L3C001R0540206488

ACTACAAACCGAGGTTAGAACGGGCGCTTTGTCTCCCCGTTAC

>V300080312L3C003R0330968860

TTACCCCAATGCGAGCGCCCTTAAGTTTGAAACGCAAATAACC

>V300080312L3C006R0160240686

AGCTGGTATAGATCCATAGCTTTTGAGTGATCAACAACGACAA

>V300080312L3C003R0311258368

CTTTGAAGAGTCCGTATGCATTGCGCATTTCTTGTCGTTGTTG

>V300080312L3C004R0410112846

CATTGCAGCGGTTGGGTCAGGTAAAAGGGCCATAGTCGAGGGA

>V300080312L3C003R0010818727

AAGTTTCTGAGTTGCGAAAACCCTTTATCGGTATCGAGAACCG

>V300080312L3C004R0451328783

AGATGTCCTTGCATATACGATAGCTTGTCGCAATATTCTGTGG

>V300080312L3C005R0550950332

CAACGCAGACAGATGGCCAGTCTTGGCAAAACTGCGATCGCCG

>V300080312L3C003R0210624352

TGTTTTTTGAGCCCTGCCTGCTTTCAAGCCATTCGCCCCGCAA

>V300080312L3C004R0070924172

GTTGTGCCGAGCTAGATTTAAGCATGAGCAAGCGAATTTAAGC

>V300080312L3C004R0451114385

AATAGTAACGAGCACTTGTGGCGAGAACTTGATGACGCACGAC

>V300080312L3C005R0020511956

GCAAAAATGTCAAAGAGGGATGAAGCTGCTGCTGCACATCCAT

>V300080312L3C005R0250229111

AATAGTAAGCCCAGACCTTGAAATCTTTGAAAAAGGTATGTTC

>V300080312L3C006R0490164662

TGCAAATGTTGCCTTCCTCATCGCAAAGACCGTCGCGCTGCTT

>V300080312L3C001R0680369313

GATACAGAAGGCAAGGAGGAGGAGTAGTGTAATGGGTTGCATT

>V300080312L3C002R0270018658

ACATTCCACGGAAGGCTCATCATTCCTCCTCGGACATTCTTCG

>V300080312L3C005R0051016739

AGGGCTTTGTGCAGAAATCGTCAATGCTGATAAATTAATAGCT

>V300080312L3C006R0421070263

GTCACATGTAAATCTTTCGAGATCTGTAAATATTGTTAAAATA

>V300080312L3C002R0590966000

TTTTCCGTTCTCTTGGGCTGCCATAATCTGGTAATTTCTTTTC

>V300080312L3C004R0680385976

CTCAGAAAATCATCTATCTAGTAGCTTTCCTTCTAAATCTCGA

>V300080312L3C005R0070396974

ATTATGGCGCTGACCTCGTATCTAGTCTTCATGTAGAGCAGAA

>V300080312L3C002R0271076551

GTCAAAGAGAGCCAGACTTCAGCGAAGAAATCCGTTTCCAGCC

>V300080312L3C002R0520804641

TCTTCATCAAATGCCAGCTGTGACACCTTCCAAATCTGCTATT

>V300080312L3C003R0090216712

CAGAAAAATAGACCGGTCCTATGAAGATATTTTCTCCCACTTA

>V300080312L3C002R0511142536

AGATATTTTCTCCCACTTAGTGGTAAGTAATACTACGCTTATT

>V300080312L3C003R0140064746

CACTTATGAATGTTTATGTATCTCAAAAAATCCACAAATATCT

>V300080312L3C001R0510905242

GAGCCTTCTCTATAGAAATACTCTCGGCACGCTTATGCATTCA

>V300080312L3C003R0360185909

TAATGCCTATCATGCATTGATGGTGTTGTACGTTGACCAAAGA

>V300080312L3C002R0720569897

TCCAAACAATTCGCTCTTGACTTTCTTCTTGATGTGTCTGTCC

>V300080312L3C001R0630054574

AACCTGTCAGGATCTTGCATATATACCGTTTGGAAATATTTCG

>V300080312L3C003R0161290853

TCAGGCAATTTCTTCCTGCTCTGCCATTTGCCTCCTCTTCTGT

>V300080312L3C005R0590267584

GCATTCTCGAGTCAAACAGTTTCAAGTTCGGATAATTATACCT

>V300080312L3C005R0190404027

TAGAGATGTATTATAAGGTTCTAACTTGACATATTCAGACCTG

>V300080312L3C005R0060714946

GAGATGTATTATAAGGTTCTAACTTGACATATTCAGACCTGCA

>V300080312L3C001R0660324896

AACACTGCTGGTCGGTGCCACATTGCCTCGAGCTGATGCAATC

>V300080312L3C002R0590379610

CAGACTTCCCATTAGCAAGACTCGTCGACAACATGACCACGGA

>V300080312L3C005R0160294736

TGAATGGTGCCGCTCAAGTCAATGAGCAACGCTTTCACTTGAC

>V300080312L3C003R0550143705

TAGTTGCGGCAGGAAATCGTGTGGCTGTATGCAGATCATGAAA

>V300080312L3C006R0711077231

TGCACTGATGGTGCATCGATCCATTGATTTTATTGATACTTTG

>V300080312L3C005R0030614524

CCTTCACGGCATCCTCGTGACTTGCTTGGGCTTTTCGCAGTGC

>V300080312L3C006R0291297477

CATAATCTGAACGCATGTTATCTAGCCTTGCTTCATGCTGCAT

>V300080312L3C006R0050039979

CTTTCAGCACAGAGCGAGGAGGCTTTTGACCAAATTTATCTTC

>V300080312L3C006R0611325323

GTTTCGAAACAAGAAAAGAAAGAAAAAGAGAGAGGCCTGTACC

>V300080312L3C004R0280588445

TTATGCTCGGACCCGGATTCGTTGCAGCAACCCCGTGATTTGA

>V300080312L3C002R0620610304

CAAACCGTTCTTCGCATTCTTCAATAATCTAATGCGGGATATC

>V300080312L3C005R0021404644

ATGTCAGCAAGCAAAATTTTATAGAGAGCTATGTAGCTTCAGC

>V300080312L3C005R0160861080

AATTGTTTACGACGCGGACGCACAAACTTTTCAAGACAATGGA

>V300080312L3C002R0640847361

AACTCCATTCACAAGAATGGACTCGTTACAACTAGTATGGGGT

>V300080312L3C003R0630548367

CTGCAATTAAGAATGAAGACGATGCTCGCGCTGAATGCTGTGC

>V300080312L3C006R0720934414

TACGAAAGCAAGGTAAGACATGGCGCGAAGCGGCAGAAGTCTT

>V300080312L3C004R0110905318

GCTCCAATATCGGAATACCTTCGTTTAGTCACAGAAGGCTGCA

>V300080312L3C003R0680206100

CATGTCAAAAACTGGCTATATACCTTTACATTGCTGTATAAGG

>V300080312L3C002R0340048885

GGATAACGCATTAGCCGTTGTGTTGTGACCAGCAAGGAAAAGA

>V300080312L3C001R0401025346

TCAATACGCACCGCTTCCTTTTTGTACAAGGCAAAAATCAACA

>V300080312L3C005R0391048761

TCCTTCTTCAGTTCGCTTTCTCTTTTCAGCCTGTAGATTTGAA

>V300080312L3C002R0300940483

CCTCCTTCAAATGTTTCCGCATATTCCCGGTAGAGATAATAAA

>V300080312L3C003R0260978385

CAGTCAAGTCGGTGGAAGAAGAGAACAAGGAACAGGGATATAC

>V300080312L3C001R0371231133

CTCGGGTATCGGCTCGTATGGATGACAAGGTATTTTCCAGTTC

>V300080312L3C005R0020817803

TGTTGTTGCGGTTGTACAGCTGCTGAAGAGTCACCGAGACCCA

>V300080312L3C004R0250314131

TTTGCCTTTTCACCTAGCACCATACATCAGACTTGTATGTTTC

>V300080312L3C005R0490379389

ATCTGGATTTATCGTATCGCTTCCCCCCGCAGATCCCGTAGCT

>V300080312L3C005R0450468088

CAGGCTATCAATTTCAGAGCTATGCTAAATTCTCTGTCTGTCA

>V300080312L3C004R0320246572

CGTAAGTCTACAGCTAAGAATGGTACAGAAGGAAAGCGATTGC

>V300080312L3C002R0110667093

TAACAAGTCATGCGTCATCGCACTTGTGGAATCTAGCTTATCG

>V300080312L3C002R0370924425

GGAAAATGGGATCCTTCTTCATCGTTCTGCGTATTCAAAGTAA

>V300080312L3C006R0470421338

AACGCTTAGATATGGCTTTGTGAGAGGCATACGCACGTTTGCA

>V300080312L3C004R0591246200

GTATCCAGATTGCTATTTCTCCATTAAGTTACTCGTCTTCAAC

>V300080312L3C003R0431300408

GATTGCTATTTCTCCATTAAGTTACTCGTCTTCAACTAGAATA

>V300080312L3C005R0200872362

CGTTGTATAATTCAAAATGTGCAGACAGCATTGCACACTGATG

>V300080312L3C001R0151163815

ATATATCTTTATCGTATGCCGAAGTATCGCATACAACGAAGGG

>V300080312L3C006R0180147947

GCTAATGTCGATTAATGAGAAATCTCAACATTCACAGTAAATC

>V300080312L3C002R0620287089

TATGCTGGTCTCTATTCAGCATTATTATGGAGTAGGCCAAAAG

>V300080312L3C005R0610590280

AGATGTGGTCGCATGGTATCCAGCACGTTAGAAATTACACGCA

>V300080312L3C002R0011135441

GGTGTGTCAACGTATACCCATTCTTCTTCCAACGATTTTAATC

>V300080312L3C004R0150819781

TAAAATTCACTTTAGTTTTATTTTTGATCGATAGCTTGCATAT

>V300080312L3C003R0540257949

TACAATATGAGCTTTGTGAGCTATCCTTTCTTAACGTTGCAGC

>V300080312L3C006R0161202574

GCATTACCGCTGTCGGATGAACTGGCGGTTGTCATTGATGAGG

>V300080312L3C003R0551109587

ATAAAGCCGAAAGGGTTTAATAGAAGGTCAAAGTTCTGAGAGC

>V300080312L3C005R0200318751

AGTTTCTTTGATGACGAGTCGGTAGAGATGGTAAGAGGTTTAT

>V300080312L3C003R0370670114

GCGGCGGTGGCAGGAGAAACTTGTGTGCTTGGTCGAGGAACGG

>V300080312L3C005R0340592368

GGAGAAACTTGTGTGCTTGGTCGAGGAACGGGTTGCTCCTGGG

>V300080312L3C002R0440505960

CTGCCGCTGTTGTTGTTGCTGCTGCTGTTGGCTAGCAGAATCA

>V300080312L3C001R0131035130

TGGTCTCGCGTTTGCCGCCAGTTTTAGAAAACCCACCTACTCT

>V300080312L3C004R0380324342

GCTTGTTGAGGAAGCCTGCGACGTCCTTATAAAGAACAATATT

>V300080312L3C006R0711326244

CCGGTCTATGACAAGAGTCCAACCGACTATGTTGGAATGTTTG

>V300080312L3C002R0240104780

AGGAATTCGCGTGCGGTACACACAGAGGTAAGAAACTTACGCA

>V300080312L3C005R0420430991

AACTTATGGAATCCGATATGGATCTGCCGCTGGCAGGATTACC

>V300080312L3C001R0200092132

CCTGAAAACGAGTCTGCAAAGCAACAGTATATAAAAACACTCA

>V300080312L3C006R0721034016

CATCAGGTTGATTTTGCGGGGCGGCTTTCTGGAACTCGGGCAA

>V300080312L3C003R0170122311

TTGGCCATGCACTCCACTCAGGATACAGTTGTTCGAGATATTC

>V300080312L3C001R0650832228

GTTCTCCTTTCAGCAAATTGATAGGTACCTGATCGTAATCGAT

>V300080312L3C001R0370665573

TACATATCGCCATCCTGAAGCTGTTGAACGTGGTACTATCAGG

>V300080312L3C006R0410560324

TCGAAGTTTCCCTCGGTGACTTGTCCAAGGACGAAGCCCGTGC

>V300080312L3C002R0291102914

TGCTTGATGACCGACGATGATCCAGAACAGGAACGCAGCGAGC

>V300080312L3C002R0630603517

AAAAAGGTTTCCATCTGGTAGCTGGCGATGGCTAGATTTGCAG

>V300080312L3C001R0191308334

CAGATTTTTGAAGTATTCCAAGAGTATCGTAGCCATCCAGCAG

>V300080312L3C002R0300264601

TGAGGGGGATGGTCTTCCGTCTGCATGTATACATTAAGCATCT

>V300080312L3C003R0510897875

CAGAAATTTGCGGATGCTACCATGTTCCCTTATTAAGACTAAA

>V300080312L3C002R0091165011

GAGTATGCCGCAGACCCAGGACGACGAGGTGAGTTTGTTCCCC

>V300080312L3C005R0230831917

ATCATGCAGGTGGAGACGGCGAAGCAAGCAATGAAGGTAAAAG

>V300080312L3C005R0510295420

GCAATGAAGGTAAAAGGGGTCGAGCATGTGAAAGAAAGCGAGG

>V300080312L3C004R0361226909

TACAGAGCAGAGTACTCCAGTAAGCAGTTGTCAGCAGCATGGA

>V300080312L3C002R0181209518

CTTCAGAGATAATACCCCGCTCCACCACCTAAGGACGATAACA

>V300080312L3C002R0420290525

CATTTCGGAGACCTATCCCAATTAGAAGGGACTCGTTAAGATC

>V300080312L3C004R0251270119

TTCCTTACACAGCACATTCCACGGCTGGTTATCCATTGCAAAG

>V300080312L3C001R0030800503

AACACAAAAGGAATTTTTCGCTGTTCTGTGGAGAACAAAGATA

>V300080312L3C006R0471062938

CCTAGTGACATCTTGCTTACATTAAATTCTCAGTACGCACAAT

>V300080312L3C004R0710933347

CTGCAGCAAATTATCATTACAGGGTGGGGGAATAAGCATTTAC

>V300080312L3C003R0381076176

GACATCGTGGTAACTCGGTAATTTGTCATATAAAGATGCATCC

>V300080312L3C002R0481163834

CGTTCCAACTTTGATGGGAAAGCTGAGAGACATCCGCCGAGCC

>V300080312L3C005R0440187461

ATGCGATCAGCGAGTGAGAGGTCCCTAGACCTGGGATGAAAGA

>V300080312L3C003R0560443731

CAGTATCATCGACTAGAGTTTTTCGCCGACGAAATCTTTGTGA

>V300080312L3C003R0530374409

TGATCGTGAGGAGGGCTACGAAGTGGCATGGAACAGCATGCAG

>V300080312L3C006R0441002081

CTTAAAGTCTGTCCGACATCCCAACATTATCGCTTTCCATGAC

>V300080312L3C005R0431339956

AGTCAGCAGTTTGGAGAGGATTCTCTAGTGTTATTCTTCTCTC

>V300080312L3C001R0320994479

CAAAGAGGCTTCAAAAAATGGATTAAAAAAGCTTCACACTGCA

>V300080312L3C002R0170714792

CACAGCGAGGTGGGTGCCACTGCTTTTCTGATCCAAGTGCTTA

>V300080312L3C005R0171063415

ACAGTCGCGCTGACCATCCTTGCAAGCCCAGAAACGGCCTGCC

>V300080312L3C001R0010474077

GGCAAAAGATGCAGTAAAAAAAGATATAGAAATAAATGACAAG

>V300080312L3C002R0480757731

CTTCAGCGCGGAGGTCAGCGATTTTTTGCTTGAGGGTTTCAGC

>V300080312L3C003R0240120355

GGTCAGCTTCGGCGTTGGCTTCGGCTTGCTGGATCATTTGCTC

>V300080312L3C001R0200119943

TGCTCAGACCAGAGGAGGCGGCAATGGTCATGGACTGATCACG

>V300080312L3C004R0040634639

GGCGGCAATAGTCATGGACTGATCACGGCCAGTAGCCTTATCC

>V300080312L3C001R0420436959

CATGTGAGCAGCCAACTTACCGGGATGTTTGAGAGAACAGAGC

>V300080312L3C002R0550944534

GTCACGTCTTAACCAAGATGCAAACCTATGGTGCACAGTTTTG

>V300080312L3C006R0660663169

TGCAAACCTATGGTGCACAGTTTTGACATACAGCTATCAATTG

>V300080312L3C001R0610835430

AACTCTAGTCTATCAAAAGATACAACCTTCTACAATTACAATG

>V300080312L3C005R0261148972

ATCCCCGACATCAGCACCGCAAAGTGCCTGAAAATTATGGATC

>V300080312L3C004R0721233446

GTCGCATCTCGACTAGTCTAACGTTAGGATTATCTTTTTTCGC

>V300080312L3C003R0350347217

GGATTCTCTTTTTTCGCTGCATTTATATCACGCCAAGATATCA

>V300080312L3C003R0300793060

CAGACGGAGACCAGCTTGCCCGATGATTGGGACCTTCAGGACC

>V300080312L3C006R0621137216

TGCTACGATCGTTCGAAATCGTTCACCCGGTCTTTGCACCGAT

>V300080312L3C006R0380997877

AGAGATAAACACACCTTTTTCTGCACTTCTTGAAACAGTCGTG

>V300080312L3C005R0030952909

AGAAGTAAAAGAGTATTGATTTGGCAAACATTAAACAAATCAC

>V300080312L3C003R0671280636

TGGATTGGAAGTCGAGATCATTTGCAATGAAAGAGATCGCTGC

>V300080312L3C001R0671219631

GGATTGGAAGTCGGGATCATTTGCAATGAAAGAGATCGCTGCA

>V300080312L3C005R0340557690

CGTGGTTCTCGCAGACTTCGCGAAGCTTGGCTTCAAGCTCCTT

>V300080312L3C004R0110441571

GCTTTTTCGGTCTCCAGTATGCTCAATAACAATAACGAACACG

>V300080312L3C002R0630128608

ACCCGATGACTTCGTCTTTGTCTCTGTCATCGACTTGCACAGC

>V300080312L3C002R0141356135

ACACACTCTACTCACTCTGCAACAACCAACCCACAAAACAACC

>V300080312L3C002R0640539547

AACCACGCGATATATTTCTTGACAGCTGGAGTCGAATAACTTG

>V300080312L3C005R0221319906

TAATGGGAGCTCAGGAATACCCTTGAAGCCAAAGTACTCGCGG

>V300080312L3C002R0090293510

ATTCTTTTGTCAAATATTACAAGTCAAGAGGGGGATATGCCAG

>V300080312L3C001R0260739180

AGATGACAGATTGGCTTCAAGAACTTTACACATCGTCGGAAAC

>V300080312L3C003R0280602688

GTCCTTTGACAAGGAGCTCGCCTGGCTGGTCAATGCCAAGCCC

>V300080312L3C001R0530952535

AATTGATACGTGATGGCAGGCGAGACTTCAGTCAAGCCGTAAC

>V300080312L3C005R0330865978

AACGCGTATGATCCCAAACAGCCGCCTGTATAGAATAGCAAGC

>V300080312L3C005R0531001100

ATAAGAGGGTCATTTTAGAGCACCTTGACAAAAATCCTTCGGC

>V300080312L3C004R0110690330

CTGCAAGTCTCATCGATCGCAAGTATAATGGTCCTGAAAGTCG

>V300080312L3C001R0330307597

AGCAGCTTTTGAACACAACAATCCATCAATGTGCAAATTCATC

>V300080312L3C006R0691218930

TATGTCAATACGAATGATTCTACAGAGTCTTTAGATACCAGTC

>V300080312L3C002R0611136135

GTCGGAGGTGTCTCAAGCGAACGATGCTTCAAACGCATTTTCC

>V300080312L3C001R0380641797

ACGAAGGGATTGTATCTGTACAAATCCACCAGTGTCCCAAAAC

>V300080312L3C002R0720326830

GCGTGAATTTTGAATTAAATGTCTCCTTTGAGACTTGCTCCCA

>V300080312L3C002R0710157861

TGATGATTACTGGCGGTAGTGAAAGTAATCTTTTCGTCTGGAG

>V300080312L3C005R0170826711

ATTGTTTCTTTACCTGGCACTGCTACTTGGGCAGCTGATTCCT

>V300080312L3C003R0010845795

GCTGTCAAGAGAGTACACATTTATATCGTTGTAATATGTATTT

>V300080312L3C002R0710098997

GATACTACAAGGAACGAGAGGAAGGATCAGCGATCGATTCTAA

>V300080312L3C001R0640268226

GAGAAACAATGCTGGATAATCTAATTTTGCTACAGTGCTCACC

>V300080312L3C005R0551084241

GAAATCGCTGCTGTTATTGATAGGCACTTTGCGGGCTCGACTG

>V300080312L3C002R0671201674

GTCAATAAGACTTCAGAGAATAGTACCTGCTTAGAGATTTGGT

>V300080312L3C005R0660608825

GTAATAGAGACTATATTGTTTATGGTCCATGTTCTTTCAGGTC

>V300080312L3C001R0440402759

GATCGTTCCATCAGCTGTTGCCTTGAATCAGGAGGTCGACCAT

>V300080312L3C005R0551216810

GAAAACTTGAATTTCTATCGACCGCGTTGTAGAGAAAATGTCA

>V300080312L3C003R0400182029

GAAAACTCTCCAGATTCTTGGACTTTCTCACCCCGTTTCTTCT

>V300080312L3C003R0640545609

AGAGGAGTATCTAAGCGAGTGAGTGAGTGAATGTGTGAAAGGA

>V300080312L3C005R0640687816

ATCTAAGCGAGTGAGTGAGTGAATGTGTGAAAGGAAGGAAGTT

>V300080312L3C003R0720793136

ACAGTAATAACTTCCTTTTCTCCTCAATGGATCCGGAACTACG

>V300080312L3C001R0360117009

CACAGGAATATAAACCGCTTCTCCTTTGCTGGGGATCAACAGC

>V300080312L3C005R0231194943

ATCCCTCCCCTCCCCTCACATTCCAAGCTATGCTCGTCGAATA

>V300080312L3C002R0440268587

CATCTTCGAGAGTCATGGACGACGATAACGACCTTTTACAGAC

>V300080312L3C001R0390398880

CACTCACGGCTCGGCCTGATCCATATGCACTGCTCATTTTACA

>V300080312L3C002R0410412600

CCTTCATCGAGCTCTTCTCTGCCGTACTGGTCTCGTGGGGGAT

>V300080312L3C006R0381183743

CAAACTGTTTTCAATCTTGACAAATACCCAACAGTCGACAGTG

>V300080312L3C002R0710337855

AGTTTGGGCTAAATGGCAAGTGTGCACGAACTCTAATCCGAGG

>V300080312L3C002R0280036998

CCCTTTGTGCGTTAGTCGTCACTTTATCAGCCCCGATGCATTA

>V300080312L3C002R0201000875

CGACAACAGGTTTTTCTGCTCTCGTCTCTTTTCCTTCGTCTGA

>V300080312L3C005R0671303660

GAATTTAGAACGCGTCTCTGACGAGGATGAAGAAGATATTGAT

>V300080312L3C003R0241178381

TAAGCTTTTGTATCCTTCAAGAGACTGAGTAATGCTTTATGCA

>V300080312L3C006R0670853902

TATGTTGTTTTCCCGTCCATGCCCGCCCGAGCGCAACCCTTGA

>V300080312L3C002R0550945063

CTAATGTCTTCAAAGATTTTTCCAGCGCAACAGGATATATGCA

>V300080312L3C005R0321308065

CACTCCTTGTGAGCGTTTTCGAGCTCTTTAATAGCCTTATCAA

>V300080312L3C005R0490942206

TTATGTTGAAATGTAGCAATAAGGAATGGTAGCTGGTGGAGGG

>V300080312L3C004R0200455961

GCAAGATGACACCTTCGTCTGCCCGCTCGGACGTCTCGTCGCC

>V300080312L3C004R0651373577

GGCGAATCATTGGAGAAATACAGGGACCATCGTCTGTCATTCC

>V300080312L3C001R0720657710

CGCTGTCAATCATTCGTGAGGGAGAATAAAATGGGCGGCGGTG

>V300080312L3C003R0180684909

TCCCTTTCTACATACTTTGCAATGCCGCTGACAAGTTACGATG

>V300080312L3C005R0700320113

CAATTGCAACGGCAAGAAACAGCACCGCCACTACCACCACCAC

>V300080312L3C003R0300516484

CACTACTACTAGAAGAGTAAAGTATCGGGAATGAAAAAAAAAA

>V300080312L3C001R0640890300

TTCAAGCTGCTGGAGATACGATGCATTCTGTTGCCGTCGTGCC

>V300080312L3C002R0430240976

AATGGAACCCGGCGGTGGTCATTCTAGAAGTTTATCGGTGCCG

>V300080312L3C003R0660603335

CGGTTCCATTCGAAAGGACCTTAGATCGTCATCCATCGCAAGT

>V300080312L3C001R0441224491

TGTAAACCTACTGCGCTGGTCGCTCGGAACGATACAAAAAAGA

>V300080312L3C005R0691038860

GGAACGATACAAAAAAGATGAAGACCATCATTGTTCTCTTGTC

>V300080312L3C003R0330584745

CAAGATTGTAGCCCTGGTACGTAGAAAGATTCCTCAATTTTGC

>V300080312L3C005R0411364828

GAAGGAGTAAAAATGAATAGATCGTTCAAGAATACTCTTTTAG

>V300080312L3C002R0390408392

GACTGTCTGGATTTGACACCTGCAGTTTTCAACGAACTCGCTG

>V300080312L3C003R0380201704

CTTTTGGTGTCTAAAAGACAAGTATCAATAACATGCATTTCTG

>V300080312L3C004R0380973860

GACAACGTTCCCAATTTTCTTCAAATCAGAAATTGTCCAAGAC

>V300080312L3C003R0190263143

GACCCAATTATACCAAGGTACCAGGTGAGTATACTGTATAGTA

>V300080312L3C005R0630517249

GAGCGCAACAACCATCCCGTGCCTATCGGCATCGGAAAAACTC

>V300080312L3C002R0480784535

TAATCCCAACGATATGTTACTATGCTAAACCAGCTTATGACGA

>V300080312L3C004R0690663392

GACGCATTTCCAATGGAAAACGGCGTGCTCTTCAAACACGACC

>V300080312L3C002R0370224543

CAAGGCATTTCATAAACTGAGAGTGAGTGCCAGTGATATTTTT

>V300080312L3C003R0061114144

GTTAGCATACAGACAGCAGAATTACAAGTGGTCTGGGTCCATT

>V300080312L3C003R0341071313

CTTGGATAAATTTGCGAGATACTTGTATGATGCATGTGTTGGT

>V300080312L3C003R0560572282

AGATCAAACTGTACTTGGAAATACTTCTTTTGCACTTCCTTTG

>V300080312L3C005R0451189481

CCTGGACAGTTTAGTCAGCGCGAAACTGTGCAAGAGCTCGATC

>V300080312L3C006R0230660027

AAAAGTTCTTCAAAAAGCCAGTGATGCATGCAGAATTCGAGTA

>V300080312L3C006R0031172611

CAGATGGAAGGCTTCCTCAAAAGTCGGGTGGAGTTCTTTGAAC

>V300080312L3C004R0281067206

GCTTAGACCAGATTGATGCAACTCTAAACATTATCTACCGGCG

>V300080312L3C001R0450435856

GAGAAAAGAAGGAATATGAAGTCCGTGCTGTCGAACTAGCACC

>V300080312L3C002R0441015240

CCTTCTTCTACAAACATTGCAAACTTTATAAATAAAAAGGTCC

>V300080312L3C002R0080750411

CATTCTCCATTTCTTCTCTGAGCAGTCGGGTCTCATTTGAATT

>V300080312L3C001R0090836309

GGACATGAAACCTGGATCACGTGTCATTGAATCAGGTATGGTT

>V300080312L3C005R0410005080

GCATGGATTGGACGATATCATAGAGATTCGACAAAGGGACGTG

>V300080312L3C004R0510929421

GTTGAAATTGTGGAAACTATACAAACGACGGAGAGATTTCTGC

>V300080312L3C006R0680339527

TCAAATTATTTCGATTCGCGACATTCAAAGATACCATTATGAT

>V300080312L3C002R0470920340

CTAAGCTTACACTTAGGGTGCAAGATGCTTACGCAGATGCAGG

>V300080312L3C001R0620780718

ACAGAATCTACAACACAATCGATCGCGTGCCTGATATCGATCC

>V300080312L3C005R0681239824

CCTACTCGTCCCGATCTCGTTATCCTCAAGGATCTTTCTCTCA

>V300080312L3C002R0380644243

CAAAAGCAACGTATTGCCATTGCACGTGCTATATTGAAGAACC

>V300080312L3C002R0210075917

TTATTTATCTCTAGGCCACCTCTGCCCTTGACACCCAGTCAGA

>V300080312L3C002R0570165617

CTGAGAAGGAGCCAGTTATCACGATGAGACGGAAGAGTTCTGT

>V300080312L3C001R0720929351

CACGGTGTCAGCAACTTTACGCTTTCCAATTTGCGCAAACACT

>V300080312L3C002R0210248190

GAACGGGAATTGGCTGCACTTGTCGCCTCAAAGGTATGCACAG

>V300080312L3C003R0480592289

GAAGGTCCGGTCAATATTATCAGGCGAAGAAACTATTAGACTC

>V300080312L3C005R0520962573

GAGCAAAGAAGTCCGAGAAAGAGAAGGATCAAATGGATGTCGA

>V300080312L3C006R0600520631

AAAGAACCACCAAAGGAGGAGAAGGCCGATTCCTCCAAGGAAG

>V300080312L3C003R0240246248

CAAAGCGCAAGGTACAGGAGGAACTTGGCGTTGCTGTTAAACG

>V300080312L3C006R0050766892

ATCATTCATTTGCTCTTGATCCGTTGTGCTTTTCGTTTATATT

>V300080312L3C006R0640842508

TCATAGTACTGTCCAGACATGCTGAAGAGAGCGGCACGACTGC

>V300080312L3C004R0121009105

CAACCCAATGTCTGCACAGACAAGGACGGTACGATTGACCGTT

>V300080312L3C006R0620557600

ATGGCACCAATACCCTCGTTCCTGGTACTCACAATGCCTCCAG

>V300080312L3C004R0421208920

CTACCACGTGGGTCGATCCACGACGACAGCAATATATTAGCAC

>V300080312L3C004R0230346207

GATCACAGCCAGCACTGCGTCCTGTGCCTGGTCAATGCCACAT

>V300080312L3C006R0110173946

CACTGAAGAGAACAAGAAGGAATACGTAAAGTGAGTTGAAGCA

>V300080312L3C004R0690957010

GGTTACACAGCTTCCAAAGGCACACACTTGGTACGGACAAAGC

>V300080312L3C002R0630469451

GCCTCCCTAAACCGCGGCACAAAACGATATTTTTTTGTCAGTT

>V300080312L3C002R0471280987

TCGTGTTGCATTAAGCGAAAAAGCTTCCCGATTTGTAGTCTTT

>V300080312L3C004R0640820407

TCTTTAAAGTATGATCCATGCTGCCGGTGACGAGCTTTGTATC

>V300080312L3C001R0691278333

AGTCCCAGACAGTGGCAGTCTTGTCTTGAGCGCCGGTAATCAA

>V300080312L3C003R0451037093

TGTCGTGTGCTCCAGCTTTTCTGTGCTCTGTTCTTCTTCCTCT

>V300080312L3C005R0360551425

GTATGACTGCTTTGGACCGTCAGCAATTCCAGCCCTCCAAATT

>V300080312L3C005R0131395399

GTTTGCCAAACAAAGCCGAAGACGCTCAAATGGATAATAAATC

>V300080312L3C003R0681190016

CGAAGACGCTCAAATGGATAATAAATCCAGTCTCGGAGAACTT

>V300080312L3C006R0191224090

GACAATACCGGCAATGACCAAAGACCTTTTGAAAGTATCATGG

>V300080312L3C003R0111222325

TGCAGTGGCTTCATTGAACTTTAGTCCTAAAAAGTTGACGACC

>V300080312L3C005R0130235920

GACGACTTCCAAAGACGATAGCAGATCCCAGTCGCGCTCCTTG

>V300080312L3C001R0300779300

CCCAGATAACTTGATTAATGTCTGCACCAGATCAACAACAGTG

>V300080312L3C002R0700410270

TCCGGAGCTCTCGACATGGACGCCTCTGTCAAAGCAGCGCGGT

>V300080312L3C005R0110802926

TGCAAGAAGAATATATTCAGAAATTCGCCACGCCGCTCGCGGC

>V300080312L3C001R0660564834

AATAATAATAATAATAAAAGGAGAAAGAAGGATTATTCTGCCG

>V300080312L3C006R0160728671

ATTGGGATACCCTTTCTGACCGTGAAACGAAACAGCAACATCT

>V300080312L3C001R0630909517

CGAACCCTTCCGTAACTGGTCGGGGAAATAAGAATTGGTACTG

>V300080312L3C003R0490310504

CCATCAACGCGGGTGTCAGAAGCGAACACCGAGGTTGCTCCAG

>V300080312L3C002R0650960836

TGCACCAGCGCCTAAAAGGCACGTGTCCAAACGGCGAAAATCT

>V300080312L3C001R0130942454

CAAAGAACCTAGCAACTACTACCTCTACCACAACATCCAGCAC

>V300080312L3C005R0010160612

CAGGGACGGCCACTCTGCAATGCTTGCGGTCTTTTCTTTAAAC

>V300080312L3C003R0630413896

TCTTTATGCAGCGATAACTATGGTTCTCACCGCAGAGCAGTTG

>V300080312L3C006R0640237171

AGCCGTCGGCTTTTGGTTTGCTCTCGAAGATTGCACTGACGAG

>V300080312L3C001R0580491344

TGATCTCGGCAAATATGGTATTAATCTAGGAATTGGTTTCTTG

>V300080312L3C001R0450070939

TCGCAGCCGATTGGCAAGGACGTTTCTGGGCTCGTGAATCCTG

>V300080312L3C002R0480918506

CCCACGGAATAGTTTCGCAACAGTCCACCAAGGGATTTTGTTC

>V300080312L3C001R0040618694

GCCTGCCATGAGTAGGTAGGTAACATGAGCAAAGCGAATATGT

>V300080312L3C006R0270283660

CCATAAGTTCAAGATCACCCGAGCACAGAACAAGCATGTCAAG

>V300080312L3C003R0301401714

TAGATACATCCCGCAACTACTATCCTGTGGAGGACATCCTCGG

>V300080312L3C002R0261004010

CCGCATTGTGCTTGAATTGGATATGCCAGCACATACTGCAAGC

>V300080312L3C004R0520533149

ACTGGCGGTGACGAGATCAATACCGACTGCTATGCGCTCGACA

>V300080312L3C005R0390626361

AACGCCGTCTTGATACTGGAAGAATCGAATGTACACCCATCCA

>V300080312L3C001R0581034740

CCGAGCAATGCGACGCGGTGTTCTGGTATCAGATGCTTAAATG

>V300080312L3C003R0380278381

GAAAAGACGCCGCTCATGCCGTTAATCTGTATGCCATATCTAC

>V300080312L3C006R0421094443

TTCAGAATGAAGTTAAAAAGCTGCAAGCGGAAGATTCCCAATA

>V300080312L3C006R0071264521

CGTCCGCTCGCTGTATTCATATGTCTCTGCGAACGAAAGTGGG

>V300080312L3C002R0130338916

CAATGAGAGTTGACCCGTCATAGAAAACTATCGAATAATAATG

>V300080312L3C005R0450778545

ATAAACCAGCGCGGGCGTTGCTGGAACCGTAGCACATATCTTG

>V300080312L3C004R0640258017

GTCTCAGACCTGTGGACTGCTCACTAACCCTATGGAGCATTAC

>V300080312L3C003R0540747850

ACCCCCCAATCTGGCAATCTGCCCGTGCATCCCGATTGTTGGG

>V300080312L3C001R0341072489

ACCAGAGAAATATCTGTTTGTGGAAACAAAAGAGCAAGATAGA

>V300080312L3C003R0231220224

CTCTGAATAAGCTAAACTGGTAAGATTTTACATGTGTATTCCA

>V300080312L3C005R0210572768

GGCGTGTGCACGAAACTTTTGCTTTTTCTTTCGCATAATTCGT

>V300080312L3C006R0561008173

GGAGACAGCCAGCTGCATATGCGTGTATAGATATATCCAGTAC

>V300080312L3C005R0570354037

GAAAGAGGGAAAGATTCAAGTTGGTGCGATGGAAGGGGCGGCC

>V300080312L3C003R0400511024

TCGACTCTAATTCTTGTGCGCATGTCCATTTTAAGACTAGTCT

>V300080312L3C006R0131147843

TCGTGAGCTAGTTTGTTTGCCCGCACACACACACACAAAGAGA

>V300080312L3C002R0540931176

CTTCTGCTTCATCTTCTGCCCCTGTCACAACACCTGTGCCTCA

>V300080312L3C001R0050057541

CGCATGATTATTAAATCTAGGCCTTTTTTTGGATTGCTCTAGG

>V300080312L3C006R0220390002

GGGCTCTTACCTATGTATCAATGGTACGTATCATTTTTGGTGA

>V300080312L3C006R0720505556

GTGGCAGCTGTTGTCGGCACTAACACGATATTGTTCATTCCAA

>V300080312L3C005R0200392025

CTTGAGTAAAGTATTAGCGACAGGATCCGGTGACCATGCCTCT

>V300080312L3C002R0420499989

GCTAATTGTGTGATGATAAATGGCTGAAGATCAGGATGCATGA

>V300080312L3C005R0621337258

TTTCTCATGAATTCCAAAGCCAAAAAACTCTGAAAATGAAACG

>V300080312L3C001R0520249236

TATCTAGATATTTATTGCAATGGTAGAGTTTACTTATTTGACA

>V300080312L3C001R0090455595

GTTTGGTCTGTTTCACGTGCAATGCTCTCCAAACTAGTCATCA

>V300080312L3C001R0571067136

GCTGTGTGCATTATATAATTGAAGTCGTTCTTGCACTGCTTTC

>V300080312L3C006R0461207167

TCTCACGTATATCATCGCCCATGCTCTATACGAGTATCTGTAA

>V300080312L3C005R0500915781

TGTCTGATATCTTCAAGCATCCATGTCTTGCCAATAGAGAAGA

>V300080312L3C006R0171225226

GGTGCTTGCTTGCTTGACATCTTCAAAGACTTTGAGATGAATG

>V300080312L3C002R0340861804

ACAGGTACACCTCCACCCATGAGCCCGACATCTCAGACAAACA

>V300080312L3C005R0290368646

TGGTGTTACAGCAGGAACGGATCATCAACGACGTAAAAGAATG

>V300080312L3C006R0300722141

TACAACATGCGATGGAGAACGACGCATCCGGGTAATCACTTTG

>V300080312L3C003R0300791141

TAAACCCAAACTTCTCAATAGTAAGCTCCGTTTACTTTTATTT

>V300080312L3C002R0701191315

TCCTTCGATCCAGACACGATCCATCGTCCGTCAGGTGTGCATG

>V300080312L3C003R0040117504

AGACACGATCCATCGTCCGTCAGGTGTGCATGCTACTGAGAGT

>V300080312L3C005R0291119393

AGTCTGTACGTCCCAGACCCGAACCATCTTGTCTAAGCTGCCT

>V300080312L3C005R0171156746

AGGGAATATATGTCTTGCTCATGGCCTATAAAACATTGTCGTA

>V300080312L3C002R0420655972

TTTCGGATACACTGCGAATCGTACTCACCAGATTTGGTGACCG

>V300080312L3C001R0230452024

GCCAAAGCGCCCGGAGGAACAGCAGGAGGAGGACCTCCCATCT

>V300080312L3C001R0551153693

CATAGCTATGGAAGGGCATTAAAATTGGACATTGCAGCCCCAG

>V300080312L3C004R0200534404

ACTCTGCTGGAAGTTATTCATTTCTTGAATCTGAGCATTCACT

>V300080312L3C004R0150951352

GGTAACCCCGATTCTTGCTTGCTGCACCTGAAAAACTCAAAAA

>V300080312L3C004R0421201285

CTTGCTTGCTGCACCTGAAAAACTCAAAAAAGTCACACTAGAA

>V300080312L3C005R0370672830

GGTTTGAGGGGGGGATGTGGCGGATTGAAGGGGGGAATTCAAG

>V300080312L3C003R0540838487

AATCGACGACTATGTCATGAATTTGATGATAGAAGGCTACTTG

>V300080312L3C004R0200846774

ACACACTCTACGCACGAAGAAGCACAAGTCGAATCTAATAATT

>V300080312L3C005R0140528569

GACACTTCCAGAAACCCCGCGGACGAGCAACTTTGATCATCCG

>V300080312L3C004R0531124635

CCCCGCGGACTAGCAACTTTGATCATCCGTATCTCCAAAACTA

>V300080312L3C005R0320728174

CAGAATGATTTTCCGTATGTTACCAACTAGCAAATCCAGTCTA

>V300080312L3C001R0230056475

GTCGTAGGTCTTGTATTCAACAACTCCAAGGTAATCAATAATC

>V300080312L3C005R0570054113

GCTAATCTGCCTTTTTTCCATTACCTTTTATGGAATCCTTCTT

>V300080312L3C006R0661034101

CACTTTTGGAGTTTGGCAGATAGAACAGACACCAATCTGAGGA

>V300080312L3C006R0511333209

AGGAGGATTACACCACGTCTACTGATAAAAATCCATGATCATT

>V300080312L3C004R0440388860

CACCTCGTTATTTTCTAATATCTACGCTCAGAGGGAAGTAGAC

>V300080312L3C004R0320411112

GTCGAATAAATGCCGCAAACGCGATAGGTAACTGGACTGCATC

>V300080312L3C001R0491127975

GAAAAGTTTCCTCAAAGATCGATTTATAAGAGCAACTGTCTTT

>V300080312L3C002R0670914744

GGGCTCGGCCCTCTTGGGTTTTGCGAAGAGGACCCGTTGGGGT

>V300080312L3C005R0251283590

TATGGACTCTTCCCACCGCATGCTCTAGCAAGTACGAAACATG

>V300080312L3C005R0680823965

CCTTAGTAGGTCGCTGCGTCTGGTAGACACAAATACTCTGCAA

>V300080312L3C002R0650543655

AGAACCGGGGCAGTGGGTTCGATCCCCTTTTTATCGCGAAGTA

>V300080312L3C003R0240561226

GCATGAGTGCGTGTAGGCCGGTTGGTGCGTGCGTGCATGTGTA

>V300080312L3C005R0190143740

CGAGCAACAGCATCCAGATAATGGAGACCCCTTATTCAGAAGA

>V300080312L3C006R0701177988

TAAACTCGTCGCAAGATCCTCCGCTTGCATATCTCAATAAAGG

>V300080312L3C006R0101298381

ACTGGACTCTACATTCGATCGGCAGATCATCATCTTCTTCGTC

>V300080312L3C002R0261221160

AAAGACAATGACTCGTCGTCTTCATCTTCATCATTGTCGCTGC

>V300080312L3C005R0211030106

CCATGCACTTCTTGACCTTTGAATGTATGAGATTGGAGCTCTT

>V300080312L3C005R0180762119

ATTGATATTGGACTTTTCACGATTTTTGTTTTCCTGCAATTTA

>V300080312L3C001R0021035126

ATGTGCTTGATTATCTGACGCTCTGCTATGTTTTTGGTAGATC

>V300080312L3C003R0350129339

AAGGTGGGATTACCGGTCCATTCGAGTGTAGCTGTCGTGATTT

>V300080312L3C002R0401008443

TCATTCGCCACCAAACAGTACAAAAGACTCGCTTCGTCATTTT

>V300080312L3C002R0181173645

CCCCATCACGGACGCCTACTCCACCGTTATCCACGGTCTCATC

>V300080312L3C002R0480605109

TAGACAATTCTGATATAAGGAACAACTAGTTTATTATTTTCTT

>V300080312L3C004R0210222404

CGAACTAGTTCCGATTGATAATTGAATTCGAGCGGCAAACGTA

>V300080312L3C006R0431202533

CGCCATTACCTCGGGCGTTTTCAGATGCGCTGCCATCCATCCC

>V300080312L3C005R0270357820

CAAACTTGCGCCCATCATTGCTTCGGTCGCGTATTTGGTGCTG

>V300080312L3C006R0720651426

CTCGACATGCTGCAAGCTGAGAGAGTATTAGTTCTTGCCCTGG

>V300080312L3C006R0661091591

ACCGATCTGATCCAGACAGCCAGCAAAGATATCAATAGTGTGA

>V300080312L3C005R0360767917

CAATTCATGGACTTGAGTCTTTAAATATCAATTAGCATTGTGC

>V300080312L3C001R0500960349

GATAGGTGGGATTTGTGCCACGCGGTTCAAGTTATATTCAATA

>V300080312L3C002R0620121054

TAAGTGAGGGCCGTGGCGAGCATAGTGGTGTCCACATGTCGGT

>V300080312L3C003R0181103207

CTGCATGAGCCTTGCCATAGTGACGTAAGAGACGGTTATAGCG

>V300080312L3C001R0620569930

CAACCAAGAGTTCCAGATGACCGATGAATATCGATGCTTCGCA

>V300080312L3C006R0480447942

CAGATGACCGATGAATATCGATGCTTCGCAGTACTCAGAGCCG

>V300080312L3C003R0141167174

AACATCGTTAATGTCACCCTGCATGTAGAATGCGGATTCAGGC

>V300080312L3C002R0371131143

TTTGCAATTGATTTCTAAACACGTTCTATGAGGCCAGCTGCTA

>V300080312L3C002R0340109685

CAAAGAAACAATGAGGAGGCAAAGATGAAGGTTTTGGCTTGCG

>V300080312L3C003R0560362997

TGCTTCTGCGAGTTCTTTTTCTTGCTCTGTGGCCGGGAATTTC

>V300080312L3C006R0380520770

CGTGGATCGAGAGCCTTGTATGATCCCCACAGAGATTTCCTTT

>V300080312L3C004R0491126122

GCACCGCCAGTATATTAGAACGTGCGCCGCCGTTTGCATCCGA

>V300080312L3C002R0330176173

GAAAACAGCACCTTATTGCATGGAGTACGGAAACAAACAGCCT

>V300080312L3C004R0240711893

CCTTATTGCATGGAGTACGGAAACAAACAGCCTGTGCGCTGCA

>V300080312L3C001R0660126404

TCTATGATTACGATGCTATCTCGCTGCCGTCTTTCCGCGGATG

>V300080312L3C002R0480331661

TACCAGCGGTTGGCTCAGCGAATAGGCGTATCTGCATGATCTC

>V300080312L3C003R0030644567

AACGCGACAAACATACGGATTTAGTTCTCTGCTATTCTTGTTT

>V300080312L3C005R0480039158

TTGCAGTCGTATGCTGGTTGTTTCCATGCCTTGCTGTCACGTA

>V300080312L3C002R0231193164

ATCCCGTTCACGTAGGTCAGGGCAAATATAAACAAATAGAATG

>V300080312L3C003R0470287915

ACATACGAATAACGTCCCAAAGATATTTTGGTGCCCGGCCAGG

>V300080312L3C001R0210219348

CTAAACGGCGAGCGGCTGAACGATTTTGCGGAATTAGCCGAGG

>V300080312L3C004R0491352314

TAGTCGAAAGAAGGGATTCGAACCCGCACACTCATTGATCACC

>V300080312L3C006R0341343974

CCGAGCGTGTAGCCTTTAACGTGTTTGACAAGAAGGTAAATTG

>V300080312L3C003R0520833572

TGCTTTAGCTTGGAGCTGAGGATCCACGAACAAAGGAGAGTGA

>V300080312L3C006R0410281231

TGCTCGACTTTTCAGTGGCGTTATAGTATTAACAGCATTGAGA

>V300080312L3C004R0271150019

GTGCGAATTGGCATTGTCTGATTTACTATCTTCGTCGTACTCG

>V300080312L3C004R0310951001

TCGTCGTACTCGTATTGATCGCTGTCGTCTGAACCGCTCACGT

>V300080312L3C005R0690048308

GGCCGCAAATTCGAAGTGTCGATCAATGGTGACATCCTCTTTC

>V300080312L3C005R0640369670

GAAAAAGCCTGTAAAGGATGCTGGCAATTTTAGAGATGTCATC

>V300080312L3C002R0490011991

TGGCTACTCACTTCTTTAACCTTCCTGTACCTCTTGGATCGTT

>V300080312L3C001R0330740422

GACCCAAAAAGCCTTGAACATCGAATCCTGTAGCAAGACACAA

>V300080312L3C004R0500295360

GTTCTCAGAGACTATCAAACGTTTGCAGCCAGGTGGATAGTCG

>V300080312L3C005R0351298207

GTGCACGATAGGCCCGTCAAAACTCTCGAACTCCCTTGGTATA

>V300080312L3C004R0460725288

CAAAAGATGACACATTGGAAATACATGTCAAAAAGATTTTAGA

>V300080312L3C006R0180973042

TGCACGTTGCATACTTAGTATCACTTCTTGAGTATCCAAGAAT

>V300080312L3C004R0061092087

ATTCCGGATGATTTGAGCATGCCGCAGCAACCTGTTCTGGATG

>V300080312L3C006R0170129441

CGTTTCCATACTGCCATAAGGCATTCGACATACAGAAATCTGT

>V300080312L3C004R0600250497

GATTGCAACAATACTCCATGTTCATACTCGAATTCTGAGTGTG

>V300080312L3C005R0020803469

AGTGACGGAGGTGTTAGGTGTCGATTTATTCTTTTGTCTATGG

>V300080312L3C005R0111308440

GGATACTTTCAGTCTTCCACCCATATCTCTTGAGCATTGGTTC

>V300080312L3C005R0571322621

GACCACATTGTTGTCCGTGTCCCACAAAACCTGCTGCGAGTCT

>V300080312L3C001R0700655282

CATGTCGAATTCGATATACTGACAAGCCATGGGCCATAGATCA

>V300080312L3C002R0650762283

AAGAATTAACTATCCCTACAGAGTGCAGGCTGCCATTTTTTTT

>V300080312L3C003R0171265054

GGTTTGCAATGGACCTGTATCTGATGGAGATCACCATTTCTCG

>V300080312L3C005R0560578658

AGGAATCATACTGTAAAACATTTTTACCAACTTCACTCTTTTC

>V300080312L3C002R0360707748

TTTCACAGCAAGATGTAACAGCCGTACCAATCTTACAGATGGT

>V300080312L3C004R0340160215

CAAAAGTCTACTTTTACTGAACAGCTTTTATATATTGCGCAAA

>V300080312L3C004R0361254403

GTTACTGTTCCCTGCCTGACACAATATGCAAACATTATTGCCC

>V300080312L3C004R0540793066

GCCACGGCTTATTTACTGAAACCAACGATACAACGACAATGCG

>V300080312L3C005R0381304690

AGCGATATTGTGCAAAGAGGTAAAATTAAACGTCGCACTTACA

>V300080312L3C005R0311136747

GAGTCATATTAATATTATACTAAAGAATCTCTCTTCTTATCAC

>V300080312L3C004R0191218843

CCGTTGACCGAGGTTTGTCAAGTAACGGTCAAGGATGTTGGCC

>V300080312L3C001R0160633660

TACGCATTCCTACGTAGGTGTGAATCGCGCGAGAGGAAATAGA

>V300080312L3C003R0330975735

CCTAGCCGATAACGAGCTGCGAGATAGACAGGAGTAGAGTTGT

>V300080312L3C006R0470444852

TTTAGGGATTAAGGGTTTATATTACGGAAATCCCCAGTGTCTT

>V300080312L3C004R0100674612

GCGCTCTTCCCTTTCTTCTTCAACAGAATATTCTTGTTATTGA

>V300080312L3C002R0560477663

AGTTTATTCTCGATTGCCTTCAAAACAACCGGATAATTCCTCC

>V300080312L3C002R0360560462

TCTCGATTGCCTTCAAAACAACCGGATAATTCCTCCAATGTCC

>V300080312L3C002R0361391908

TATTTCCGGTTTACTCTTTTCAACCTCGACATGTCACGCTGGA

>V300080312L3C002R0140762859

TATACACTGCTTCAACTTTTAGAAGAGTGGTTTCTAAGCATAA

>V300080312L3C001R0670055190

TATTTGACCTTGTACTCTTGACTTTTCAATGCAAGATTCAGGG

>V300080312L3C001R0171139348

TGTTCTGTCATTCTGCGTCGTTAACTCGCTCGTAACATAATAG

>V300080312L3C002R0191028744

CGCGGTGTCTCGTGGCAACAGAAGCCGGATCTGACCGATCTCC

>V300080312L3C004R0530793646

CAGCTAGTGTGACCCTGGCGATTGCAACGAGACACCAGCTCTA

>V300080312L3C005R0160506520

GTCCCGAGGACACATGGTAAGTAAAAGACTTGAGATAACTAAG

>V300080312L3C003R0571404166

CCTTTGATTGGTCCTAACGAATAGTCGGCCGGATTCAGTTGAT

>V300080312L3C004R0561139994

TATTTAAGATAGTCTTACGTATGGCTCCAAAAAAGCAAACTAT

>V300080312L3C001R0140230811

AACAGGACATTCTAATAGAGGCAATCGAACCAGCTGTGCCACA

>V300080312L3C004R0370209283

ACCACTGCGCCACTGTGCGCAATGATGCGAATGCCGGATCAAA

>V300080312L3C004R0310358046

TGCGCAATGATGCGAATGCCGGATCAAAAAATAGCAACGGTCG

>V300080312L3C002R0360851194

GACCCGTGAAATGCCGGGTACCGAAAAGCATTATAAAAGGAGG

>V300080312L3C002R0550149492

AAGAAAAAAGCTAATATATTTAGACAACAAAATTCGGTTATCG

>V300080312L3C001R0510426495

TGCGAGTGCTCCGCATCGAGAGCAAGATCGAAGCGGTTCTGCG

>V300080312L3C003R0180616538

TCGTCCTGCGTTTCCAAGCTGGGTGCAACAACAGAGCAAAGTT

>V300080312L3C003R0420772131

GAAAAGAAGGAAGAGAATCTAGTTGCTACAAGTAACGACTGTG

>V300080312L3C004R0591028392

CGTTTCCCTTAGCGCCATCTTGCAAGTGAATGTTTTAGAAGTC

>V300080312L3C002R0300280003

AGTGTCCAGTTTAGGAAGTATGGAAGAAGATCCTTTTCTTACG

>V300080312L3C003R0090996687

CGCTAAATGCTGCACAAGCGAATCCTCACAAGTTCAACTTGTC

>V300080312L3C002R0300913659

TATTTTAGATGACCTAGGAGATCGAGTCGATCGAACAGAGAGC

>V300080312L3C001R0280515612

CAGAGAACCTAGGTCAGTTGAAGAACAGATGCGCATGGATGAA

>V300080312L3C001R0640260507

CACTGGTGTCTCTTTCTTCGGGGACTGACAATGAGTATAGAGG

>V300080312L3C005R0040873966

GCTTCCTATCATCAAGGAACGTATGATAGAAGCAGGAAATGGT

>V300080312L3C003R0560604715

CAACGCAGTCTGCCGTGGCTGTTGCCGCTGCTGCGGCCGTCCA

>V300080312L3C002R0641078087

GGATTTCTCGACAAACGTCAAATGGCTGCAAATTCAGCACATC

>V300080312L3C005R0080793978

GTTATTGTTGCTGTTATCGTTGTTGTTGCTAGAGGGAGTAGGA

>V300080312L3C003R0070842468

GTGCACTGACATGATGGATAAGGAAGAGAGCGGCAATCAAGTA

>V300080312L3C003R0540158908

AATGTTCGCGTCGGGGTCTCGTGGCAGCGCAAAGCAGTGGTGG

>V300080312L3C001R0491030179

ACCCCGACCGCTAATAGCGGCAAAAAGGAGAGTCGGATGTGCA

>V300080312L3C003R0210191502

GATCTGTCAAATGGACTAAACGCACAATGTCCTATTTGGACAA

>V300080312L3C004R0010883167

ACAGCGGACGGTACTATCTTCGTTCAAGCAGGTGATGGTGGTA

>V300080312L3C003R0401152810

TTATGGTCATACAGCAAATTTACTGTAAGGAATATCTGAGGCT

>V300080312L3C004R0120617682

AAACTGCAAATTTTGACTTTATAACTAACAATGAAAACTGAAC

>V300080312L3C006R0231013846

CAACAGCTCGGATGGAGGAGACGATGGTCTTCCATACGATCCG

>V300080312L3C006R0430671370

GGCAAATGGTCATGTTTGGTAATGAGCGCGCAATCCATGAATC

>V300080312L3C006R0211053398

GGCATTCGTATGTTAATAGGTCAACACTCTGCATTGTATCGTG

>V300080312L3C006R0330535694

CATGACCAATTGATGTTTATTGTAAAACAACGGTTAGTTACGG

>V300080312L3C001R0130478003

AAGCAACTATAGGAGGTAGTGTTTACCCGCAATTACATGGAAG

>V300080312L3C003R0670524420

AAAGGAATGCCAAGCTGCAGCCAGAACGTACTCTTTGCACGGT

>V300080312L3C004R0251246153

ACCTACTCCCCTTAAAGTATTGTTCTTCAGTGATTCTGTTAAA

>V300080312L3C005R0580545334

CGAGATCTCAGAACAGGAAGCAGCTTCAGATCGACGGTTACGC

>V300080312L3C006R0481011743

CGTCGTGAACTTCCTGAAGATGTGCGTCTGAAGATGGACCTCG

>V300080312L3C004R0571402698

TGGACGGACGAAGTGGACCCATCTTGCCAATGAAGACACTTCA

>V300080312L3C005R0681355247

TATATTACCCTATAGTCAACTCGCGGCCAGAATAGAAGCGTTT

>V300080312L3C003R0170125562

AAAAGGGGAGTTGTAGGAAAGAGGAGAATAGAAGCTCGGACCC

>V300080312L3C006R0521365211

TGTAAGGGAAAGCAGGGAAGTTTGCTCCTGCGCGGTGGACATG

>V300080312L3C005R0311155414

GTCGATAATGCAGAATGTATTACTTACCAGGTTCCGGCCTGTT

>V300080312L3C003R0690878013

GAGGAATGTGTTGATGACTGAGGGTAGGTGCGGAATCCGTGCG

>V300080312L3C002R0440705528

GGCAGAACAGCAAAAGCGCGGCAATGCATAGTAGATACGGCCG

>V300080312L3C003R0721035387

TACAGAATTCGGCTCCAGAGCTATTTATACTGAATGTAAAGTA

>V300080312L3C005R0360420904

CACTGCGAGTTATGGTTCGTCTCAGAATTCCATCTGAAAAGTT

>V300080312L3C006R0270883870

CACAACAAATATTTTGTCGATGACTTCCTCGGAAGAGAGTCTA

>V300080312L3C001R0690060563

ATCCTCTCAGACTCAGCGTGAAACTTAAGTTTTCCGTCAAAAG

>V300080312L3C005R0141189760

ATCATGCCATTGGCTACGTGGGGAAACACGCCACTGGAAGGTA

>V300080312L3C004R0120346230

ATCTCCATTTTTAGATTCATTCGAATATACCCCAGGCATTATC

>V300080312L3C005R0430145290

AGCAGTAATTCCCCGCTTAATTAGCCCCTTACATGTGGTTCAT

>V300080312L3C003R0670366192

AAACGTTGTCGAAGAGAAGACAGGCTACGCAGCTACATTGGCT

>V300080312L3C005R0650269859

TACCACAAAAAGTCCAACACCGCCCTGTAAACTATGCTCATAT

>V300080312L3C002R0560949230

TGTCGCCTGTCTAAGGAGGCCGGCAGATAATCCAGCATAAAGA

>V300080312L3C003R0510819108

TCAACTTTATCGTTTGGAGATTACACGGATGCTGGTGGTCTCC

>V300080312L3C006R0300326387

TTTGCCTCCACTCTTTTATCAGTGAAGCTCGCAGTATAGACTA

>V300080312L3C001R0081045629

ACCGATGCCTTATCGACCTCCACATCCGCAGCAACCGCATGGC

>V300080312L3C001R0380710833

CCACATATAGAATCTCTCAGTTCATACGATGATTCCGCAGGCA

>V300080312L3C002R0090243447

CATCTGCGAAACATTATGATGCAGCTGCGTAAATGCTGTATCC

>V300080312L3C005R0240596533

TGATGCAGCTGCGTAAATGCTGTATCCATCCTTATCTACTTGA

>V300080312L3C005R0040882882

TCAGATTCTGGAGCGCCGTACTACAGTCATCCGTCACGAAGGA

>V300080312L3C002R0080015253

TGAGGATAACAACCTTATCGTTTATGAACCTCGCAGGCGTCGT

>V300080312L3C004R0050961725

AAATTCAATGGACTCGCTTCAAGGAAATTGCTGGCTTGGAGAA

>V300080312L3C004R0531239521

TCCTGCGAAGAAATTATGAAGCTTCATCAGCAAGAACAGGCTG

>V300080312L3C005R0611199993

AAATGGATGCTGATTACTCTGACGACGGTGAAGACACCGATAC

>V300080312L3C004R0640994487

TAGGAAGTTGCATACAAACTTGACAAACCGGGAAAAATACCAA

>V300080312L3C002R0461107066

AGACGCTGGCCTATTATCTTGGACAGCGCCATCAAGGATGTCC

>V300080312L3C004R0161189996

CATGGTTTGTCTCGGACGTGATGCCTGGCGATGTTCCTATTAT

>V300080312L3C006R0280487331

ACAAACAAGGCTGACACCGTGGTTGGTTTAGCGCCAGGTGTCA

>V300080312L3C001R0250674390

AGTATCCCTGCATTTCTAAAGCGATTACCAGCATTAAATTGCG

>V300080312L3C005R0010032356

AAAAGAAACAGTGGAACGCCGTCCATCTTTGACGAATTCCTTC

>V300080312L3C005R0391153372

GAAACAGTGGAACGCCGTCCATCTTTGACGAATTCCTCCAAGG

>V300080312L3C003R0420122064

CGATCGGGTAACAAGGTTGACTTTATACAAGTTTTAGAGGATA

>V300080312L3C001R0330420789

AAATACACCAACGACGACGACAGCAAAGAGGAGAGCAGCAATA

>V300080312L3C004R0721239984

CAATGAGGATCTGCTTTGTAGCTTTGAACTCTTGACACCAACA

>V300080312L3C002R0400280831

TAGCTTTGAACTCTTGACACCAACAGAGCATCCGCCTGCAAGG

>V300080312L3C004R0091103160

AACTCTTGACACCAACAGAGCATCCGCCTGCAAGGATTGCAGC

>V300080312L3C003R0680865981

ACCGACTGAAGAGTATGCATGTTGATTCTCATAACTTTAAGAC

>V300080312L3C003R0401384747

AGTTAGCAACTTTTTACGATAAGCTGCATTGACTTTAGCTACC

>V300080312L3C002R0050474619

CAATACTTGGACAAGCGAGCTTACGTTATTGTCAATGGAGGAG

>V300080312L3C006R0170018209

GCTTACGCTATTGTCAATGGAGGAGCTGAAGAAACAACAAAGC

>V300080312L3C003R0271397070

CCCATCGTCTCATCTGGGGCAAACTTTTCAATACCGGTCAAGT

>V300080312L3C002R0130086137

TTAAAAAGGAACTTTAAAAATATTCATGCATTAACACGTAATA

>V300080312L3C004R0591196755

TACTGTGCTTACGAGAAGATATTATTTTATGGAAAAACAGAGT

>V300080312L3C002R0071017453

TCTTTTGGAGAATCTTCGTTTTTCTCTTTTTGCTCCTCTTGCT

>V300080312L3C004R0110380186

GGATATCCTCGTCATCATCTTGTCCATCCACACCGCTAATAAC

>V300080312L3C004R0340844538

CATTCGAGCAAAAGCCGCAAGTTCCATATTGCCACCATATATA

>V300080312L3C006R0021326091

TCCGCTCCAGTTTGCAGCAATGTATTACGTTGTTGAAAACGAG

>V300080312L3C001R0220564443

CTTTTTTACTTACACAACTCTCTTGTAGTTATTGCTCTGCGAA

>V300080312L3C003R0340605787

TCGTTTGCCCTTGCTAGAGCCTGCATCCGTTGCAGTATCCACA

>V300080312L3C001R0190452881

CCTTCTCCACCAAATGCCACAAATCACACACTGCATCTTTGAT

>V300080312L3C004R0611195763

GAATGATTAATGCGTGCATTAAACTATCAGGTTTCCCAAAGTT

>V300080312L3C003R0121125263

CAAAACATGTCAGTATGAACTTACTGGGTGAGCATGGCAGGTA

>V300080312L3C005R0260853818

ACCGAAAGTGGCATCACCAATGCAGCGGACGTTGGGGACGTTG

>V300080312L3C003R0020905148

GAACTTGCCGGTACCCTTGACATAGTCGACGCCGTATTTCTTG

>V300080312L3C001R0500638560

TTTGTAGTTGCTTTCTAAACTGAGAGATTTGCTGTCATTTGGA

>V300080312L3C006R0040534799

GCACTGGATATGTTTATTGAGAATATTCGTGAAGCTACCTATG

>V300080312L3C004R0440239006

TGCGAATGCTGAGCCTCAGATATCACTGCGCTTGCCATGGACT

>V300080312L3C004R0631017601

CATGAAAATAGTTATATCGGAGCTGCTGTTGGTTGCTCTCCGG

>V300080312L3C006R0290419601

TCCGGATACACACTCAGGTAACAATGGCTCAATCAAAATAAAA

>V300080312L3C001R0350900433

AGGTCAGATCTAGGTTATCGGTACCAAATATGTATGTCAACAT

>V300080312L3C006R0250306963

TGAGTAAACATGAATGGAAGGAGCCGGTTGAGAGGTTGCTCTG

>V300080312L3C003R0070692099

CATTTCATATTTCATGTCAGAATGTGCTTGCGTCAAGGAACAC

>V300080312L3C005R0430277692

ACTACTATGGATATCCTTGAACTTATCAACATCGAAGGACAGC

>V300080312L3C003R0260620877

CCGGTGATAGGACGTCGCTCCGATCTCGTTAGACACGTCCGCA

>V300080312L3C004R0340674554

CCACATGTCCGCCTCCTCTCCCTTTGCCCTCCACCCCCGGAAG

>V300080312L3C005R0520895926

ACCTTCGCCAATCCCATCGCCTTACACCCTTTCCAATGAATCG

>V300080312L3C002R0250780917

TAGCTTTGTTTTCTCGATCGCAGCAGCTCTATTTCGTATAACT

>V300080312L3C001R0420145760

GCTTTGTTTTCTCGATCGCAGCAGCTCTATTTCGTATAACTCA

>V300080312L3C004R0180159578

GTAATCGCTGGCGACCGCCGGGTTAAAAAGTTACCTCCACTCA

>V300080312L3C006R0040488265

GAGCCATTGCTTTTTGCGTTGTCTGCATTTTAGAGCAGCTACA

>V300080312L3C003R0300705918

GGGGGCCGGCCTCGACGACGTTTCAGCAGAGGGTTACCGTCCG

>V300080312L3C002R0230233666

TGACTTTGCCACTTTCCCCCAAAGACAACGCTTCATCGATGGC

>V300080312L3C006R0670996855

TGACGCTAATAAACGACATTAGTTGACGCATTTCACAAACCTG

>V300080312L3C001R0250195828

TCGTGAGAGGTACCGGCCCTTCATGTGCGGATACCAGTCCATC

>V300080312L3C005R0640956525

GGATTGGAGGATAGGAGTATTAGACTTGATGAACACACTCGGG

>V300080312L3C001R0181344543

GACTAAGAAACGAATGAAACTGTCGAACTGGATAGTGATAATC

>V300080312L3C001R0670791290

TTTGAGAAATAAAAGCAAGCCTCATTTTTCTTTGCTTTCTTAT

>V300080312L3C004R0411139968

GCCAAAGCTTTTCAAGGAAGCATTCGAAGACCTGGACGAGAAG

>V300080312L3C002R0540632720

ATATGGGGTGAGTTTATTATCAACGCATGTCCCAAGAATTTCA

>V300080312L3C003R0130997959

TGTCCCAATTAAGCCATTTAGTTTGTTTATATTCCGTGAGGGT

>V300080312L3C002R0630046067

ACAATATTAAGGGAATTTCAGTTTAATTTTAATGTACGGGGAA

>V300080312L3C004R0490811471

ATTTCCCTCTCCCGTCCTTCTTCACATTTATCTCTCTTTTCTA

>V300080312L3C003R0530263014

GACATGCAGACCATCTGATAGGCGATCTCAGCTTCGCGTGCCA

>V300080312L3C005R0610202907

GCAATGTTGGCACGTACAGGAACCTCGGTCGGCGTCAAGTAAT

>V300080312L3C006R0700775577

ATCGACTTACAGTTTCAGGATGAACTTCCTCGACAAACTCGAG

>V300080312L3C001R0481263234

AAGATTCCGAGACTACCTGGTAACACATTAATCTAGATGAAGA

>V300080312L3C004R0210710212

ATCCAGACTTATTGCATCTGGCTACATCGACGTTCCAACGATA

>V300080312L3C005R0101015592

TGGACTCTCCAAAGCGTAGGCTTAATGGATCGCCGCGCTGCAT

>V300080312L3C003R0701294640

AGTCGCGATGATTGGCTTCTTTATCCTTTTCATTACCTCATTT

>V300080312L3C004R0301041244

GCTTAAGAATCGCAAGAGCCTAGAGGATCAGTTTATGACATTG

>V300080312L3C004R0581352232

GACGACTGCTACAGCGACACCGCTGACTGCTGCAGCAGCACTG

>V300080312L3C001R0691234290

GCTCGTCAAGATGCATTGTTGCAAACCAGAATTTCTCGTCGAT

>V300080312L3C002R0380553994

CTGTGGCAACCAACCAATAATTTCAAGGAACTTTTCCGCCAGG

>V300080312L3C002R0640117382

TTGCCACGTTGGAAGAAGGTTCGCCGAATCTTTTGATCTCCTC

>V300080312L3C002R0250755797

TTGTAAAAGGATAAGCAGCTGCGTCAATGTAGCGACATCAATG

>V300080312L3C001R0670249917

TCGCGAGAGCCCACGTTGTCGTCGACCCATATTGTCTCAATTT

>V300080312L3C004R0031010256

AATACTCAATCATTTTGTGCACAGGCTTACGTAGTCGTGTACC

>V300080312L3C005R0511065329

ACCAGTAATGGTTAGGAAATATTCTCGATTGATACCACCATAA

>V300080312L3C001R0710758400

GTGGGACAACCCAGCAGCGGATTAGCTCGCTTTTGCATATGCA

>V300080312L3C002R0650550889

TGCATTCTGATTCCGGATCCCGGTCATTTTTTGGTTGTAAGTC

>V300080312L3C006R0161228272

CCTGATGAATTCGCGCCAAGCAGTAAACCTTTCTGCGGATAGC

>V300080312L3C003R0210719704

TTATGACAGAAGCGCTCGACCCTGCTGGGACAGTGATCTCTCA

>V300080312L3C006R0200955602

GCAAAGGCATCAACGTAAAAGGTTATCAATCGTTTTACGGATC

>V300080312L3C001R0050008021

TGTTAATAGCTGCACAGGGTGATGCACACATATCAGCTTTAAT

>V300080312L3C006R0580678998

AGAACTCTTTGCTGCAACAGACAGATTCAATCGAATCATGAAT

>V300080312L3C003R0540180232

TATTCAATTGATCCAAATGGTAGCAATTTTCGCATCTATAACA

>V300080312L3C002R0100708547

GCTGACACGCACAGGAGCCTCACACTTTAATCGACTTCATCAA

>V300080312L3C006R0560685832

CTTGATCGTCGTTAAGTCATTTTAAGGTCTCGCGGCCATCACA

>V300080312L3C003R0180203022

GTAGACAACCGGTGGAGAAATGAAAAGCGGAAGCTCTCTCTCT

>V300080312L3C006R0250095948

AAAGGACGAGACGGCTGCCAACCACGAGGAACCGAGACCTCGA

>V300080312L3C005R0521325161

GATTTTGACAATCTGCCAGGACGAGGCAAACCAATACCCAAAG

>V300080312L3C004R0430360580

TTGCCCTCAGGAGGATCAATTGGAGAAAAATATGGTTTTTTGG

>V300080312L3C005R0190982379

CCTCAAACTGAATGGACATATTTTTAAGGAAGATCGCAAAGGA

>V300080312L3C001R0710960986

CTTGCAGAATGTATGGTTACATTTTCGATTTTTATTTGCATGC

>V300080312L3C004R0060564750

TCTGAAATGTGCGGTGAGGCGAAAACGGGTAGTACCGGGTGTT

>V300080312L3C006R0370718100

GCTGTCTGTCATATGAATATTCTACAACAAAGGATGATGAGAG

>V300080312L3C001R0620217853

TGTCAGGATGGATGTGCCCAGCGGTAAGATATTGAGGAAGCTC

>V300080312L3C002R0381404163

CGGTTTCAGGATCCTCAACTGGCGAGCCAAACGTATGCTTGTG

>V300080312L3C004R0020914291

TACGATCAGTTAACAAGTAATCCTGGAGAAAGTGGTACATACG

>V300080312L3C001R0281161158

CGACTGAAAGTTCATCGCATGTGAGTATTCGTTTGTGTGTTCC

>V300080312L3C004R0671304541

TCCCACCAAGTCGCAACTGGTTTTTGCCAATGGCTCTGAATTC

>V300080312L3C006R0151309267

ACTGAAAATGACAGATCGGGAAACTTGGTGCGTCCCTACACCG

>V300080312L3C006R0681299033

TCGCTTATGGCAACCTCAGGCGACAACAAGCAGATCAATGTCT

>V300080312L3C006R0501326339

AAGAAAGGATCTTTGAGTGTGCGTATTTCTCGTGAACTAAGTA

>V300080312L3C005R0481039123

GCGGTGAAGAAGCTCGCAATGCAGGACGGTCAGCTTCGTGTTG

>V300080312L3C003R0640013807

ACTTACGCAAACATGGCAATGAATGTCAGACTTAACAGAGATG

>V300080312L3C003R0190587506

CCTTTTCCTTGTCCCTCCATGAGCGTGCAGGCTGCCGTTGCGT

>V300080312L3C005R0280911512

GTCCTTGTTTTTCTTCTTCCAAAACATGCTTGCAAAGTTGGCA

>V300080312L3C004R0040310712

CAAAAGCGAGCATGAAAAGGTAAATTTTGTGAAACAATACCCC

>V300080312L3C001R0720976533

TTCTTTCTTCTGAATGCCGCGCAAAGATAACACAAACGATACA

>V300080312L3C005R0480683801

TAGATCGCCCGAAAGCAGCAGTAGAATCCGAGCGCAGAGTCTC

>V300080312L3C004R0590881400

GAGGACAAAGGCGACCGAAAACTACGAGGATACGCAAAAGACA

>V300080312L3C002R0150435525

TCAGAGCTTTCGTCTGCTGCTGCGTCAGCTGCCTCCTTTGTTG

>V300080312L3C001R0660126610

ACTGTCGGAAGGACCGTGGAAACGAACATAGAATTCCTGCATG

>V300080312L3C004R0641210199

GCAAGTAATCCATACAAGAGCTTTTTAGTAGAGGACATGAATA

>V300080312L3C003R0441070367

CTACATATGCAGACTGAAATTTTTCGATTTCTACGAGCTTGCA

>V300080312L3C005R0391023775

AAGTTCTGTAAGTATCTGTTCGATGGACATTAATGTCTATAGA

>V300080312L3C001R0600980070

CATTGTGCCGTTGCCGCTTGTGTACTATGCTCTAGATTACTCT

>V300080312L3C001R0471099912

CCAACGTACATCTGCTATTATTCCCTTTTTTGGAACTAAATTG

>V300080312L3C002R0681248930

AAAATGGCTAAGCCCGGTGCCCTTCTCGGACCCATTTTTTCCC

>V300080312L3C004R0241279749

TAACCGCGGTCTCGGTCTCATTGTTGCATCGAATCCCCGCAAC

>V300080312L3C006R0191232814

TCGCGTGACAAACAAACTCACACACAATTTTCTTAATTTAGCT

>V300080312L3C003R0090509885

TGTTTGAATCTCCTTGCCATCACTTCGTCCGTCTCAGCTTTGG

>V300080312L3C005R0071329591

CTGCTTGTCTTGTATACATGAATGAGAGTTCAATTTTTTCCGT

>V300080312L3C003R0050124043

AAACGCAGACACAAGAACCCTATTAGGAAATTTTGCTGCATTG

>V300080312L3C006R0320477670

CATCCATACCTGTTCGTGTGTTGTTACACCTCGCATCACAAGG

>V300080312L3C002R0470676636

GGTATGAATGTGAGCGTGTGTATGATTGTGCATTGTTGGTTAA

>V300080312L3C006R0670959850

CTGCACAGGAATTCCATGATAGCAGGGGATAAAAGATGCTGCT

>V300080312L3C003R0400406743

CTTGAAAAGTGGATGAGCTTCTCTTCGCGCGCTCTCAATTGGA

>V300080312L3C003R0441077184

CATCAAAAGGCATGACGACAAAGCGGGAAGGCTCAGGGCAGTG

>V300080312L3C001R0260588557

GTGTGGGAGAAGGTATTTCACTAATCATCGCAGTGCATTCATC

>V300080312L3C005R0110778600

CCCTTAAAATTTTCTTTTTTTTTCCCACCTCTACCCATCCCTG

>V300080312L3C005R0350639287

TTTCGCGACTATCAGCATTATGAACATACCTCTCGCATGGCAC

>V300080312L3C002R0210725056

CAGGCCAGCTTCGCGGCAAACAGCTGCCAAGTCTGCACCGACG

>V300080312L3C002R0400855921

CTTTAGGTGCTTTTAGGCCTGTATATCCATCCAACTTGAGTGT

>V300080312L3C003R0180930161

GGAAAAGGATGGCCAAGCTTTCCCTGTAACCATCTGTTATCCC

>V300080312L3C002R0540843016

GACATTGGATCTTTTCAGAATATTCTTGATGTTTGATGCGCAC

>V300080312L3C006R0540774923

ATCGTCGGATAGACGGCTTGTAAAACGATATCTCTTATTTCTT

>V300080312L3C002R0141072733

TCTTATAAATCTTGGGAAACGTAACGAAATTCCTTTTGATGGA

>V300080312L3C001R0480995244

TCTGAAATTCTTCTGTATCTGGATCGTAGCATGCAAGGAGAAA

>V300080312L3C006R0230264888

GGCTCATACGTAGCGTTTTCATCGTCGTAAAGTTTGACCATCA

>V300080312L3C004R0480199262

CTTACTTCGCCATTCAGATAAAGACAATCAAAAGCATATATGG

>V300080312L3C005R0141031335

TAGCAAGGCAACTGACTACATTTGGCATTAGCATAGATTGGCT

>V300080312L3C001R0410727355

TACACTCTCTATCATGTAGAAACATTAGTTAGTACAAAGATAT

>V300080312L3C002R0510099857

TTTTGATATCTGGATGGGTAATATGCCGCGGTGCTTTTCCTTC

>V300080312L3C003R0720313222

AGAGTCGCTGAACCAATCCAGTTAGCATACAATATGTAAAATA

>V300080312L3C003R0140370924

TCAACGCTTTGGCTTTCTCATTCCAGATAGCTGGCACAGTTAG

>V300080312L3C005R0080150571

CTTTCTCATTCCAGATAGCTGGCACAGTTAGACACTATTAAAT

>V300080312L3C005R0441263373

AGTCCAATAATGGGATTATCACGCATGTAGGTGCAAGCACCTC

>V300080312L3C003R0270801567

CCAACTTTACCAGCACGTCCGTCCGACTATCAGCGCTCGACAG

>V300080312L3C005R0710955156

GATTTCCTCATCGTTACAATATAGATATATTTACTCATTAATT

>V300080312L3C005R0220846082

ACTGAGCGTTGCGTCCACGGACACACATACAAGGACTTGTACA

>V300080312L3C005R0701090591

TGGTTTTCTTCATCATAACAACTATGTAGTTTTGTACGGCGTG

>V300080312L3C004R0181112797

ATGTATTCTTGATCAACGCAACATACAGGACTAGCGATCATGG

>V300080312L3C005R0661235674

TTGTAAACTACGACGAGATGTGACGATTGATTGAAATCTGAGC

>V300080312L3C005R0520914842

TCCAGCTTGTCAACAGGGTTGAAAAAGCCTGACAGTCGGTCGG

>V300080312L3C006R0541228867

GATGGCCTCCCTTGATGCGATGAAAATCAGCAGGCATCACCGA

>V300080312L3C001R0120589665

ATCTTGACTTAATGTGGTGGCCCTCGACTTGCGGATAGGACAG

>V300080312L3C005R0070750696

AGAAAGTTCTTGGAAGTCAAGTAACTGTTAGGGGCGATAATAC

>V300080312L3C002R0630115106

GTCAAGTAACTGTTAGGGGCGATAATACCGGGAACATACAGCG

>V300080312L3C006R0340163706

CAGCATTGCACCGTTTTTGATGTAAAACGCACGATACAGTGCG

>V300080312L3C001R0581079921

ACTCAAATTTTCGGTTTGATCTTCTCAATGATTCATTCTCTAC

>V300080312L3C005R0641195986

TAACAATGATGGTAATAATAAAGTAGTCATCGTTTTCTTACTG

>V300080312L3C004R0420561973

ATTGAAATATGAGGAAGTATACTGGGCTTGAACAGTGCCAGCC

>V300080312L3C006R0101112185

CGTAGACAATAAACCATTAATAGATATGAGGGCGATTTTAACG

>V300080312L3C005R0020841367

TAGAGTATAGATGCTTAATGACTTATGAACGGAGCATAAAGAC

>V300080312L3C003R0130598146

ATAATCGAAGAGATAAAACGTTAATTCCAACGCAAACCTACTG

>V300080312L3C004R0600519298

CTGAAATTTGAAACAAAACATACAGCCGCAATTCATGTTGAAA

>V300080312L3C002R0270500834

TGTGGAAAGCACTCTCAGCAATCTCTTCAATCGTCCTGATTTC

>V300080312L3C003R0291040728

CCTTGGTCGATGAGCTACTAAAGCTATACACGGCAGTCAATAT

>V300080312L3C002R0600552635

TATTGAGCTCTTGGACCAAATATATCGAAGCCAGGAATGCCCT

>V300080312L3C006R0550927686

GCAGAGGAGCGCCACTTTGGGCGAAGAACCTTTCGTCCAACCA

>V300080312L3C001R0100535306

AACTCTTGCCAAACTTCATAGCGCACCATCATTTTCTATCCCT

>V300080312L3C004R0560903800

ATCATTTTCTATCCCTTTATTTGGAGGTCGTCTAGGATTAAAG

>V300080312L3C003R0050771490

ATATGTACCGTACACTGATGTAGAATTAAAGGCTCATTACATT

>V300080312L3C006R0480352180

CGATTTATGACCAACTGGTAATGCACAGGGCTTGCATTTGACT

>V300080312L3C003R0690896396

CCAATACTCACGTATCCAGACTCCTGTTATTGAAGTCATGAAA

>V300080312L3C006R0600689162

CATGAAGGGGGAGCACGATGAAGTAAAGAATTAAAGCGGAAGG

>V300080312L3C005R0360741690

CGAAGATATTCATCGTCGGCAAATAACAGTTCATTGCCTTGTT

>V300080312L3C001R0120617885

GTGTTCGCGAGCGTGTTTCCGGGTTTGTTTCTTGTGGCAGTAG

>V300080312L3C003R0101084159

ACCTCCAATTACCCAAGCTCCTTCAACTCTATGACCTCTATTA

>V300080312L3C004R0660047819

TTCTAATGGGATGGTCCTGGAAGTAACTAGAATATGCTCAATC

>V300080312L3C003R0080289293

TCTCTGGTTTGGACAGTAGAATAAGTTTTGGATATATTTGGGT

>V300080312L3C001R0410261577

AAACAAAGGTTTGTGGATGTGCACCTACGGTAGGTGATCTGAG

>V300080312L3C005R0280057454

GTAAGAAGCACCACAAGAAGGATAACGCGGTGCAAGAGGGTGG

>V300080312L3C001R0440301953

CCCACGAATCTCTGCGGCAATTTTGCGAAGGCTACGAAGCTCT

>V300080312L3C001R0670827337

TGCTCCTTGCCTGGACTTTGCTCTAGCGTGCCAACCATTTCTA

>V300080312L3C004R0360620540

CCTTTCTGCAAGCTCGGGTGTTAAGATCGCTTGCATTCCCTTG

>V300080312L3C004R0230797476

TTCATGGTCGTCACTATTTTATCGATGTTTCCTCGACCATTGG

>V300080312L3C001R0611313539

TGTCCAGATCACCTCCAGATAGGATATATATATGGCACTCTGT

>V300080312L3C005R0651252856

CTGCGTTGGAATTTCGTTGACTTGACTGCGATTCTTCTGGTTC

>V300080312L3C001R0541233153

ATGTTGCCACAGAACAACCTTTTAAGATGCCCAAAGGTCAGGG

>V300080312L3C001R0171321961

CAATCCATGCTCGTCTGTTCCTGTGCACAGATATGCATCGAAT

>V300080312L3C006R0340312394

AGATCCAGCTGCTTTCAACATTTAGTTTGAAAAGGGGACGCTC

>V300080312L3C004R0520611185

GTCCGACAAATCCAGTCTGTGCAAGGTGCGACAGCGTTCAAGT

>V300080312L3C005R0541305658

GAGCTGTCGCATATCATGTCTCCGGCTGACAACCATCAGGCGT

>V300080312L3C003R0240672218

CAGACTCTGTAAAAGCTACCGGAGAGATTATCTTGTCAGCACC

>V300080312L3C005R0410572159

AGGAATGCCTTCCTCCGTTGTTGTTTCACCTGCTTCCAGTATT

>V300080312L3C005R0140859009

TCTTTCTTTTCTATCTCTTTCACACAGAATATTTGACATCAAA

>V300080312L3C006R0011058376

TATTTGGGCTCCTCGTACCATGCATCAAAGTGGGCAGAAATCA

>V300080312L3C004R0651100636

TTCCTCGGCTACAAGCTCTATCTCGAGTTTGGTTGGCACATTT

>V300080312L3C001R0250604382

TCATTCTTGCATACTACGGCGTGAGTTTGTTGTAAACCTAGGT

>V300080312L3C003R0571184402

CAGACGGCCGTCGCCTCTGGGCATTCAATGACACCTATTGGTC

>V300080312L3C001R0691071026

AGGACTAAATCCTTTTTTGAGAGAGTTTGTTCGTAGTACGTAC

>V300080312L3C003R0290576547

GATAACAGCACCGGTGTTGCTATGGAGCAAAGATGTCAGCGTG

>V300080312L3C005R0151321660

CCCGCATCGGTGTTTTGCATTTCTAACGATTTCCGCCGTGCTC

>V300080312L3C003R0090747663

TGATCATCGTTGCTAGCGCTGACTGCTCTCCTCATCGCTGCTT

>V300080312L3C003R0520615540

TGCATGTTTTTGTAGGTATTCTTCGACGTTTGGAATCCATACC

>V300080312L3C002R0520756334

ACAGCTGAGCAACGTACTGCCACCCTACTGCATTCGGATCCGT

>V300080312L3C004R0030100641

GGGTTAGTAGGGTTAGTAGTGTCTGGAGGAGTAGATTGGTTGG

>V300080312L3C001R0581115737

TAGATTTTGCATGATAGGAGGAGCTTGTTGCTGCTGAGCGACG

>V300080312L3C002R0680732086

ATTTTCAATGGGGAGCATCGAGACGAGACGGCCCAGCTCGTCT

>V300080312L3C004R0631406730

TTAAAGTAAGACGTATCCAAGCTCGCGCGACGTTGGAGCTTGT

>V300080312L3C006R0160440975

TCGACCTTGATTCCAGCCATTGGACCGGCCGATGTTCCGGTTG

>V300080312L3C001R0031346154

AGTTTCCTGATGGTGAAATTGCTGTGAGACATAGGCGGGATTG

>V300080312L3C005R0260625603

CCCATCCGCGGCTCGCTGCTGCTCTCCTGGCGGTAGCTACCAT

>V300080312L3C002R0210348805

GCTTACGGGTGATGCAGAATTCTCTCGACCTATATTCTGTCGG

>V300080312L3C003R0100341155

ACATCCGCTTGTAAAATTGATGACGTCCTGACAACAACAATTA

>V300080312L3C001R0180519650

AACTCGCTTGCTAACTGCTCTGCTTCGTCGCGCGAGGACACTG

>V300080312L3C005R0400090625

TGCTATTCTTTTCGAGCTCTCCTTCAAGTGGCAAACTCGACAT

>V300080312L3C004R0081038475

GTGTTATTAGACTTTTCAGGGTCTTGCATTGCTTCTTCCTGTG

>V300080312L3C002R0690698895

GAGCCTGTACGTTTGCTACCTGTCCATTAAGTGTTGACAGCTG

>V300080312L3C004R0030041485

GTTGTTGGACAAAGGTTGATCAATGCATTGTCAGCACCCAATA

>V300080312L3C005R0461328342

CAAATGTTTCGTTCTTGTTGTATTTTCTCCTTCCCCTCACTTT

>V300080312L3C005R0120995592

GTTTCGTTCTTGTTGTATTTTCTCCTTCCCCTCACTTTCATAC

>V300080312L3C003R0070857950

TACTTCGTCCTATGCATGCAACCAAACGTAAAGATATTTTATT

>V300080312L3C005R0560806575

GGCATGCTACGCCGAGTGTCCTGAAGATCCAAAGATGTTCGGT

>V300080312L3C003R0350175060

AATCCCAGAACCTATCCATGTCCAGTCGGCTCCTCCTGCTTTG

>V300080312L3C003R0051073855

AGGCTGCAGTTAATTAGACCTGAGGCAGAGATCGCGCCCAAAA

>V300080312L3C001R0390060832

GCGCTTCATGTTGACATGGAAGGCAGACTCATCTACGAAGACA

>V300080312L3C006R0330002366

CCCGCTGTTCCTGTCTACAGGCTGAAACCGGATCTTCTTATGT

>V300080312L3C002R0591372811

CATGAGAACATCCACATCCTTTTTTACGTCATCAGTCTGCATG

>V300080312L3C005R0400691302

CAGTCTGCATGGCGTCTGATGGCCTCTTGCGAGTTCGATTATG

>V300080312L3C006R0030585241

GAAAAAATGTACATCCGGAAACGTTGTCAAGGTAAAATGGTAT

>V300080312L3C003R0431110716

GGAGCAATGCGATCTGCTTCCAAAATCAGAGCGCATTTGGCAG

>V300080312L3C004R0070055793

GGAATTCTGATTATTGCTGCACGGAACAAAATGGAGTACAAAT

>V300080312L3C002R0160715373

GGTTATTATCGCCTGCAATTTGATCGTCGATTTCATTTATCTA

>V300080312L3C004R0060291708

GATACACCAGCGCCTGGCACCTCTCCAAAGCGAAGAGAGTTCC

>V300080312L3C006R0701199396

GTGCTTTTTACTTTGACTCATGGGGTTTCTACACAAGGGAGAG

>V300080312L3C003R0100347151

TGCTGCTTCTTCAATTTTCCGTTGTCCATCGGTCGGCGGAATT

>V300080312L3C006R0680711339

CGGTGTATAATCGAACAACTTTTGTTCCCATTTTGTAACAACA

>V300080312L3C002R0150576856

AACAAACTGCAGAAACCGATGTGTGTGTGCCCTGTCACGCGTT

>V300080312L3C002R0690274913

GCATCGCCGCTGTTATTACTGTCGCCGGAGGCATCGCGAAACT

>V300080312L3C003R0711256018

ACAGCCATAGCAACCATCCTTCGCCGCTGTCGCGACAGCAGGA

>V300080312L3C006R0181161615

TCCACCGTCCTAGCTGGTCTAGAAAAGGAAATTCTCAAATTTG

>V300080312L3C004R0261251099

AAGGATACAAGGCCGGCCTTGATACAAAGTGTAAGTATTTCGC

>V300080312L3C006R0470940985

TCATGTTCCACGTTGCGCCGCTGATGCCATACAAGAATCACGA

>V300080312L3C006R0380290543

CATACAAGAATCACGACAAGCAGCAGGTCCATCGGAAACGTTA

>V300080312L3C004R0400063257

GTCCATCCGGAGCAAGTGAATGGCAAAGAGGCATGGAGGTAAT

>V300080312L3C001R0550183255

CGAGGCAGTATGAGAAGTCTGCGGTCAGCTCTGATAAGTGATC

>V300080312L3C001R0221341635

ACAATTCGAATCGACGGCATCAAAAGCAGCAGCGGGAGGAGGA

>V300080312L3C002R0231221675

ATTTTGTTTAAAAAGAGGATAATCGAAGTTTTGATGAACCAGC

>V300080312L3C004R0110057231

CAGCTGCACGAAAGCTGTTCTTTAATGAGATGACGCTGTTCTG

>V300080312L3C006R0620994847

TAATGAGATGACGCTGTTCTGCGCAAAGCTGAAAAAGTACTCC

>V300080312L3C005R0630756136

TGAGTATTATTCGCGTTCATTCATAACAATTTTAGACATACCG

>V300080312L3C001R0391189038

CAACTGCACCGTCTCTTTTGAGTTTGCATATACGGCTTGCATA

>V300080312L3C002R0660591987

TCCAGAGTGCTAAGAGCAAAACCATAGCGACCAATGTCTAAAT

>V300080312L3C006R0480173989

GTACGCCGCGATCCAAGTCTATTCAGGGTATCTTTTGCAGCTG

>V300080312L3C005R0550756942

ATCGACTCTAGGATCTGCGATACTTGGACGTCCATATCTAACG

>V300080312L3C005R0120568245

GCCGGATTATTGTTCTCTTTTACCTAGATAATCGTATGATGGC

>V300080312L3C001R0130041749

ATTTTTATTGATGCATGATACGACGCGATGTGATATAAGGTTA

>V300080312L3C001R0190553705

AGTTGTGGACATTGGCCCTCGTGAGTTTATCGACGAGCACGGC

>V300080312L3C003R0341287520

AATGCTTTGCAGCGTTGACTTGATCGATCGTCTCCAATTCTCC

>V300080312L3C006R0171077667

TGAAGGTATGCCACGACAGATCAAACTGATACGAAACAAGCTG

>V300080312L3C005R0250702498

ATTTTTGACCCACTCCAAAAATGGTCTTTTAGGTGTAAAGGAG

>V300080312L3C006R0570521227

AACAAAGGTCCAAATAATCCATCCTAAAAGTAAGGCGCCGAGA

>V300080312L3C002R0690794262

GGAAAAAAGTCAAATAATAAATCAAGCTCGGCTAACTTTGACT

>V300080312L3C002R0151392104

GCACTCTCAGGAAATTATCGCTGTCATTCATTCGTACCCATTC

>V300080312L3C001R0690690154

ACTGCTTGGCTGTTGCCGCAAAGAAGCCTGCTTTTTCTTGTCT

>V300080312L3C001R0520624525

TTTCTGAATGGCAATCACGATCTTTGGTTGCGGGCTGAGCGTC

>V300080312L3C004R0650076057

GAGAAAGTGGTTTATAATTACCCAGACCGAGTAACCACAGTGA

>V300080312L3C006R0521383467

CCAGTCTCTCAAGGATGGCTCCTATGTGGCTGTAAAAATTCTC

>V300080312L3C003R0720379972

TTCTCTTCATCGCGTCATTCAAAATCAAGACTTCATCTTTATG

>V300080312L3C006R0130400438

GGACCTTGATGGTCTTTTCCTGGCCATCTTTAGTCTTGATAGT

>V300080312L3C003R0611043120

GGAAAAGATCAACCTAATCGTTGCGAAATCTTCTCTGTTGCAA

>V300080312L3C004R0370833296

GCAGCAACAACAGAATATAACGGATTTGGAATTGGTCAGTTGT

>V300080312L3C001R0441274634

GCCAAAGTTACCAAGAAGACTTATTGGTGCTTGATATATGGAA

>V300080312L3C005R0380388702

CCCGATCCTGCAAACGACTACCACGATGCCGATGAAGATTTGA

>V300080312L3C001R0610477955

ATCCAAGGTAAAGCGATGCTAACTTCCCTTAAACGTATGAAAT

>V300080312L3C005R0471163894

GAGCTGTTGGACTAACGGAGTTGCTGCTGTAGCTTGAACCTGC

>V300080312L3C006R0400528541

TGTCGAGTATAGTCGAAGACATTTTTGCATATGCGGTAGGATA

>V300080312L3C003R0080516742

CGGTAGGATAAGCATAAAAAAAACATGTAAATGATGTGGCAAT

>V300080312L3C001R0141031952

CACTGTACGGCCAGATTGTAAATGGCTTGCAGCACGTTGCTGG

>V300080312L3C005R0480558400

AGCTTGCTTCGTTGTCTTTTCCAATGCCGAGAAGAAAGGCTCC

>V300080312L3C006R0540786042

TCTCGCTTTCAGACGTTTACAATGCTATTTTGAGCTGGAACCC

>V300080312L3C002R0220938550

ACGAGTCTCCAGCTGCTGAATAACTCGCGTCAGAATCCCCACA

>V300080312L3C001R0150199187

CCACACGCTTTGCTTGCGGCGAGTCGTCAGCTCTCACTCAGCA

>V300080312L3C002R0190411052

CAATTACATTGTAATTACTCTCGTTGGCCACTGCGCTCTGTCT

>V300080312L3C004R0700283137

AGCATACGGGAGAAGAGGTCTGAGCCCTACGGCTTCTTGAACC

>V300080312L3C006R0220946697

AGCAAAGAATTAAAAGACTTATTGATTGCAGCGCAGATCGATT

>V300080312L3C004R0530956455

GACGGACGTAGCACTGGGAATCTGCGCGCATAATGAAACTGTA

>V300080312L3C002R0671345830

GGCAAGGGCGTGCATAGCGGGCGGCATTGACTCGTCTGTGTTT

>V300080312L3C004R0221402144

AGTGGTCATATTGATAACGATAACAAATGATTTACAAATTGTA

>V300080312L3C002R0301291655

CATCCACGACTTGACTTTTTCGATATCTTCGTGATCGTCTTGC

>V300080312L3C004R0121177415

CCTCTTCTCTTCTAATTTTACAATGAGCAGAAACGAAAAGAAG

>V300080312L3C004R0510526264

GCCAACTTTACCGCTTATCAAGAAATGAAGAAGTACGCCCGAC

>V300080312L3C005R0030733196

ATTCCATGGTTTGGTTTATTCTTAGAATATCGTCCAAATGGTT

>V300080312L3C005R0060962044

GCAAAACCAAGAGAGACAACCTGTACGATGATGATATCGCCGG

>V300080312L3C001R0520252614

AAAGGATTATCCGAGCTGCAGTGGACAAAGGTCGTAGCCTTGG

>V300080312L3C005R0450708366

TCTTACAAGCGTTTCCATCAAGGTCAAGAGCTTGATCGTTACA

>V300080312L3C005R0661155758

GTAAAGCCCATCCAGCGCTGGAATTTCTTTTGGAACCATCGTA

>V300080312L3C006R0121148766

CCTGGTCCAGACTTCAGCACTGCTTTGCATGATTTTTTTGAGA

>V300080312L3C005R0060862381

TGAGATATAGCTGTACACTGATCAAATCTTGATCGGTATCACA

>V300080312L3C001R0460825166

CCGCCTCTCCTTACATTATTGACGTCGCTGCTGCCACAGGTGG

>V300080312L3C006R0370360958

TCAATTCAACCATCTTCAGCAACAGCAGCAGCACTTTGGCATG

>V300080312L3C003R0031109382

GCAACTTTTACCACAGCAAGCGCAACAAATGTACCCCAACATG

>V300080312L3C005R0690825850

ATCCGATGTATCAGCCATCTGGCAAGCCGGGTGGCGCATCTGC

>V300080312L3C005R0270502419

CCCGGCGCGACCGGTTACGATGACATGTCGCCTTTAGGAGGGC

>V300080312L3C005R0051347895

ACTAAGTCCTCTGTCGGCCCTTTCGCGATTAACCTTGCACTCT

>V300080312L3C003R0401034579

GGCATATCGAGATTTGGCGGGTTAACATCGCTGCCGAGTCTAG

>V300080312L3C006R0520329877

GGTGAAGGCTCAGTAGACTTTGCTGATTTACTGCTGCTTGCTG

>V300080312L3C006R0200039188

CAAGAATAAGCTCCGGATCTTATCGCTTTAACACGGTGTGATA

>V300080312L3C006R0320203967

CACTCCATTGTGGCGACCTCTCACCCCTCCTCGCGATTCATTA

>V300080312L3C001R0591043774

AGTCGGTGATAGCTCCATTTGCCAAAGCGGACAGTCAAGAATA

>V300080312L3C004R0290964237

CATCTCTATCCCTTCGCTTTCTATCGTCGTACCGACTGCTGCT

>V300080312L3C006R0430428354

GCCTCGCTTTTTATGCTTCAGTTTGGGATGGATCTCGGCTGGA

>V300080312L3C006R0361361657

CCACAGACGCTCTTCATGCCCGAACTTGCACGTTTTACACCAC

>V300080312L3C006R0330820883

ATCTACTACACTGTCCAGTGCTTATACCTTCTGTCACTGCGGT

>V300080312L3C003R0190733588

CATCATCTTTAAAACGAGAAACAGCTCGCCGTTCGAGATGTAC

>V300080312L3C006R0350497685

CAAAAAGTTGATGTAGCCCGGAGAACCGGACAAGACAGTGACG

>V300080312L3C004R0620982193

TGATGAAAAGGGAAATAAAAGGAACGACCAAAAGTAGACATAA

>V300080312L3C001R0090128476

ATGTCGTAGCCGTGGATCACAAGAAGTCGCGGGGCGTCACTGG

>V300080312L3C006R0691345287

GACTTAAACTCACTAGATCGAGCGTTTTCATCATCAGACACGT

>V300080312L3C006R0670224302

TTAGCTTCACGTTCCTTGAGCGCTTTCTCCTTTCGCTTTTCTT

>V300080312L3C006R0340527181

CCATCAACGTTAGCAGCTGAGCTCGGCAAAACGACGTCAGGTC

>V300080312L3C006R0280415939

TCGTCGTTATCCTCCTCCTCCATATCCCCCAACATTTCCACGT

>V300080312L3C006R0160340713

GGCACTGGATATTGAAGCAACTCACCGTGGATAAAAAAGCAAG

>V300080312L3C005R0190632653

TATGTGTCAATGTGGAGCTTTGGGCAACTAAGGCAAATATAGC

>V300080312L3C005R0290470940

CCCAAGTTCCGCCACACCTCTTCTGCACCCTCCTCTGCAGATA

>V300080312L3C004R0561359977

GTGATCTTTGAAAAATGCTATTCCTGGCTCTGGCAAAACGAGG

>V300080312L3C004R0450949789

ACTGTGCGAGGAATTGACATCACCGTTCTCCTTACCCAAACCA

>V300080312L3C005R0690696905

GATCTAATGTCTCATTGTGGACTGCAGCAGACTAAACATGTTG

>V300080312L3C006R0510716304

GCACGGCAGCAGCTGAGCAGCAAGAACGTCTCCAGCATCGGCT

>V300080312L3C001R0270229289

GCTTGTCTGGAGATCTCCAATCGAGGTCAGCCGCTCCAGGAAG

>V300080312L3C001R0720849186

CACAGGATCAATGTTGTTCCAGATACCAGCCACGAAATCTGAC

>V300080312L3C006R0330099662

TACACGGCCTTGCGCTACAACGCATGCTACAAATTTTTGCCGT

>V300080312L3C005R0470286689

ACCAAGCACAACTCGAATTACTACTTACGCGGCGTCTCAACCA

>V300080312L3C005R0320054692

AGCGATTGCTCAATCAAGAAGAAGAAGTCGCCCCAATCTAAGT

>V300080312L3C005R0100830131

GGGTTGCTGGAACTCGGAACACTGTACTCACCCAAAGTAAGAT

>V300080312L3C005R0141097211

ATTCATGGTCCCAGACTCTTTCTTTCTGGCTTTCTCGCTTTCG

>V300080312L3C006R0620171772

ATTCATGCAAACTATCGCGAAACACAATACCCAGCACTTTGCT

>V300080312L3C006R0040769000

TCGTTTTCCCGGCCGCCGCTTTCTTATGTTGGGAAACACTTGC

>V300080312L3C005R0340742478

TACGCGAAATATACTGCGAAGTCTACTTCGATCAAATACACAG

>V300080312L3C005R0670119518

AAAAGAGCCCTTATTCCCTGACTTATAAAATGTGGCTATAGCC

>V300080312L3C005R0580147222

TATGGGCATTAAAGATCTTTGGGAACTTATTGCACCTGCCGAA

>V300080312L3C002R0610977546

AGTATCTAAGCGATTGCAGAGAGTTGACAAGATTCTTGGACTG

>V300080312L3C003R0200898457

TGGCCAAAACATATGAAGAAGCAAAAAGGTCAAGACTGACCAA

>V300080312L3C006R0320876732

ATACCGTCGGAAAAACAAGACAAATGTCAATGCTAGATTTCTT

>V300080312L3C005R0351043350

AGAGCCGGTACCACCACCCAAGGAATGTGCGAGCTGGAAACCT

>V300080312L3C004R0590775553

AAAACGTTGATTCTTTCGAGTTGCAGATCGTTGTCACCGCAGT

>V300080312L3C002R0320094790

TAACAGCCTTTCCTTTCCATATCACGGCATACATACTGATGAT

>V300080312L3C002R0531097312

GCAGTACAAATTTACAGGAACATACCAGCACGAATGAAGTACC

>V300080312L3C002R0130338058

GATAGAGTATCGAACGAGAAAGCGTGACCAAATCTTTCTTTGC

>V300080312L3C006R0330987667

GACTTAGTTGACCTGAGATGAGCTGAGCGGCGGGGCTGTGTCG

>V300080312L3C003R0510924864

AGCTTCCGAACGAGCAGATAACCTGAGCACCATCACGTACTCC

>V300080312L3C006R0201239387

ATCGTCTGCGTCTTCCTTTACGTACGGCGTCAGCTTCAACGAT

>V300080312L3C003R0530445189

TGGTGTATTCTTGACGATATTTGCCGGAAAAGCGTCAGGCACA

>V300080312L3C006R0430112947

GACATGGTCGGCGGCCGGAACACGTCCTTTTTCGGTGCATCGC

>V300080312L3C004R0120829096

TCAAACAAAAAGAGAAAGGAGAATGCAAGCAAGACAGACTCTT

>V300080312L3C001R0101063526

CCCATCCACGGCCTCTATATCTTCGACGCTCTCATCGGTTTCC

>V300080312L3C003R0220229819

TGGAACGTGATATCAAAAACAGCCGCATGCTTACCGAGATCAG

>V300080312L3C001R0401144656

CCCCAACAAAAACACGTCAATGCAATAGGCAGCTAAAACAGAT

>V300080312L3C001R0450429743

ATATCGAAGATAAAGCTGTAGGCGATTTTGGCTCAGGTTGTGG

>V300080312L3C003R0580354985

AGCATTTGATGATGCTAATAGGGCTGAAGTTACTCTGTCGGTT

>V300080312L3C002R0641300576

AGCATTCAAGACGCTTATGGATTCATACAAGGAGCAGATCGCG

>V300080312L3C002R0200628640

TCTTGGATAGTCAGCTGCGACGCCGTTAAGTAAGCAAAAAGAG

>V300080312L3C003R0280394452

ATCCGCTTCCGAAGTTACAAACGGAGGGCAAATGACTATCTGG

>V300080312L3C005R0550464514

GCTGGGGCTACTATCGGCTATCGAGGATGTGCGGGCCCTATAA

>V300080312L3C005R0250929617

CTGCCTATGAGTGCAGAGTGGACGATCGCTTTTGTTCGAGATG

>V300080312L3C002R0590306626

ATATATGCATATATGCAGCAAAAGAATAACAGCACTGAAACTA

>V300080312L3C004R0241035771

CCCTTCAACGTTTCTAGCTCTAAAGATGCACGATGCGTTCCAA

>V300080312L3C006R0420121838

CCTTCAACGTTTCTAGCTCTAAAGATGCACGATGCGTTCCAAG

>V300080312L3C003R0570102968

GACCAATTGGTAGAGATGCAAGAATATCAAGAAGCAGTGAGCT

>V300080312L3C002R0500242742

CGATCAGTCAAAGCGAAACAAAGGAGGACGATGATTTATTGCG

>V300080312L3C004R0410405080

CCAAAGGAGAGAAGATGTACCTGACACTTTTGAGAGTGTATCT

>V300080312L3C005R0510503069

TTGAAAAATCAATCCGAGCTGCTAACAAGGAGCGCAACATGGA

>V300080312L3C004R0560903876

CAATTGACTTACTACCGATCGCGAGCTGTGAAAATTACGGAGG

>V300080312L3C001R0531125247

GACTCAACGTGCCAATTGGCGATAGTTCGCTTGTCGGCAGGTC

>V300080312L3C002R0500885063

ACCTGGATAAGGACGACCTTGTCGCGCCATTCTGAATAATCTT

>V300080312L3C006R0690140065

CGAGGCTGCTACTACTTGCACTTCCGTTGCTCGCCACCGAATT

>V300080312L3C006R0030826837

GGTGAACTCGTAGCAACACAGGAGCGGGAAGAAAACAGCAAAC

>V300080312L3C003R0251001601

AGCTTCCAGAATAGCATCAAGCTGGCTCTTTTTCACTCCAGGA

>V300080312L3C005R0241124481

TACATCGTTATCTTCTGCAAAGTAGATATCATTCGCATTCACC

>V300080312L3C002R0401192154

ATATTTCTGAGGATATACTCTTGAACAAACAGGCTGTATGTGA

>V300080312L3C004R0171035785

CTCACTCTATCTAGCAGACGCACCGCCTCATCTGCTTCGTAGC

>V300080312L3C005R0040360356

CAATCTTGTCCCAGTTTCGAAGACTGTACCATCCTATATATTC

>V300080312L3C005R0131339766

CTGCAGCTTATCTTTTCACCGACGACATGTGCTGCATTGGTGC

>V300080312L3C005R0010643575

GGGCACGATACTTTTCCGTCTTGGGCACTATACTTTCTAGGAA

>V300080312L3C004R0281082969

GAGGTAGTCTTGATAGACGCAACGTACAAGACCAACGATCGTG

>V300080312L3C003R0720560416

CCTGCAAAGTTTGCGAACCACTGCTTCCTCGATCATTTCTCGA

>V300080312L3C003R0720049755

CTATTTCAAACCGTAAGTTCCACATTGCAACAAGAGCATTTTG

>V300080312L3C005R0280883781

GAGATTGCCGCTTAATTGGTTGACATCGAGTTTGAGGTTTTGC

>V300080312L3C005R0691319245

TAGCCAGCGCTGCCCTTGAAAAGACAAGCACAGCCGAATTACA

>V300080312L3C002R0540386832

TTCCGATCCATACGCCGTTGTCAGAGTAAAGTATGGCTTGTCT

>V300080312L3C003R0551014855

CACGGCATTACGATGAACATCTATAAATTACTACCACCACGCT

>V300080312L3C004R0120498662

CAGTCTACTCTAAGCAGCATCTGGAGAGCATTCTTGCCCTTGC

>V300080312L3C002R0120704152

TTTTGATTCTATTCATATGCAAGTGATTAGTAACCAATCAATT

>V300080312L3C001R0340278680

ATAAAACCAACCCTGACTTGCTATGGACGCCACTTGATAAGGA

>V300080312L3C002R0080610218

ATCTCGGCGGCAACAAGATATAGCCTTGTTAGCGAAGCGGTCT

>V300080312L3C003R0431163012

TCGACGGGAAACGGGGCGACGCAGAAAGATACAATGTCTCCTC

>V300080312L3C005R0481338540

GCAACAAAGAACGACGTTGATAGACGTTCGAGTAGTCTGAGCC

>V300080312L3C004R0020416508

TGTTCGCGAGATTGAGCAGACCGATATCCCGTCCAACGACGGT

>V300080312L3C004R0431101023

ACTAGCCCATTTTCCAGAGTTCTCCTTGCTGAACAGTAATTAT

>V300080312L3C003R0100863647

CGCCAGGATCTCTACAGCATCCTGCAAGACTTGTCAGATTACG

>V300080312L3C003R0491084890

ATCACGTTGCACTGCTTATCCGCCACTTGCTAAAGTCGACGAA

>V300080312L3C005R0010914881

AAGATTGCAAAAAGAGAGTACAGCCTCGTGCTGACAAAAAGCA

>V300080312L3C004R0651181972

TATTCACGGTACTATGGTTAGAATAGGAGACAAGATTTATCTC

>V300080312L3C001R0030837835

AGATTAATCTCTTTGTACCCTTGTACATACTATTGCCACACGT

>V300080312L3C001R0080278797

GTCAGAAGGAAAGCTCGAAAAGATGGAAAAAGACTTTAGTCCA

>V300080312L3C003R0311078343

TCATCAAATTGTGCCGTCAAGCGCAGGACTGGAAGCTTTTGAA

>V300080312L3C001R0480222328

ATGAATTCTGCTAGATCTACGTTGAAGTCGAAAGAGCTCGTGT

>V300080312L3C006R0620226450

TCTGCTAGATCTACGTTGAAGTCGAAAGAGCTCGTGTGACACG

>V300080312L3C001R0540360025

CAAACTCGTGGTTTCTAAGACCATCTACGCACGTATTGATCGC

>V300080312L3C005R0010620963

GAGCTCGCTCTCGGCATGCTTTACGAGCTTCGGTCATCATGGA

>V300080312L3C001R0150250502

TGACGTTCGGCTTCGCATGTAAATATCGACAGTTAAATATTGG

>V300080312L3C004R0270337171

AGCGACTGGTGAGAATGGGTTCATAGAACCGGTTTGATTAATC

>V300080312L3C004R0201257881

GACTGGTGAGAATGGGTTCATAGAACCGGTTTGATTAATCCTG

>V300080312L3C001R0140761577

CATTTAAAGTACCGACAGATAGTGCAACTGACCAATCTACCAA

>V300080312L3C005R0210234075

CAAGTAACGTGCAAGTGTCACAGCATGTCGGCCAGCTCCCCTA

>V300080312L3C005R0040784651

TGGTTGACGGTTGCGTATATCGATGCATCGATAATCTGCAAGA

>V300080312L3C005R0410716805

TATTATTCGACGTGTGGACAGATCTTCCCTTTAATTCCAATTC

>V300080312L3C001R0700338702

ATCTTCCCTTTAATTCCAATTCCCTTTTCTTTCTCGTCTCTCA

>V300080312L3C002R0481270526

CCGAGGAGGTCTACCGCGAGTAAGTACAAGTGTATAATGACCC

>V300080312L3C002R0120745028

TGCTTCCCGTCCAGAAACAGACTTTGAACAGGATCCTGCGCTT

>V300080312L3C001R0180585915

AAAATCGTCAAGGAGAAGTACCACACCGACTTTTACATTCTCG

>V300080312L3C004R0110910047

TGCTCTCAATGGGTTTCATCTTTTCCAATACAACTCGTAATTC

>V300080312L3C003R0640541627

GACAATCAGCTTTTGCAGATCTCCGGGTACGGCTATAATTATA

>V300080312L3C005R0710614165

CCGGGTACGGCTATAATTATATGAGCAATGAAGAAAGGCCATA

>V300080312L3C003R0360984541

GGAAGGACCGATGTACGAAGTTCGCAGAGCCACATGGTTCATG

>V300080312L3C004R0590933329

GGCGATGGTTCAAAGTGGATTCCCTGCGAAGAAAATCTTGCCG

>V300080312L3C001R0150005113

CGACACGAAATATCGTTTGGCATGGCCAGCGATGATGATGATA

>V300080312L3C001R0081394965

TTTTATTTCCGGTACAAAACTTTTTTGCCGTGGGTTCTCCTCT

>V300080312L3C006R0690975195

AGTTACCATATGCTACCCCGCTGTTGAAAACCTATACAACATC

>V300080312L3C004R0700714354

TGAACTTTGCACACAGATCCCAATCATAAATGTCAACAACAAT

>V300080312L3C004R0460457102

TCTACGGGCTCAACTGCATTGTTGCAAGCACGCAACGCTGTTG

>V300080312L3C004R0041012019

AGGAATCCGTATTCCCAATTCCGTGATATTTATACCCTCGATC

>V300080312L3C006R0090720861

TCTTATGATAATAAAGCTCGATGCTATGGAATGTCTTTTGACA

>V300080312L3C003R0331383283

AAATATAAAACATATTTTACTTTCGAGTCTCCAAGTACACGAC

>V300080312L3C005R0390161671

GAACATCGAAAAATACAATCATAAACATTCTAACGAACTCAGT

>V300080312L3C005R0150990147

CTGAATCATGTGCATTTTCGGCAAGACGTATCAAGATGCCAGG

>V300080312L3C004R0670277651

CCTGTTTTGCAATGGGCGCGAACCATGTCCCGACCGCTTGTCC

>V300080312L3C006R0550081264

CATAATGTCCATGATATGGTGAATTCAGTAGCTTTTATTACTT

>V300080312L3C002R0121325619

ACAAATGCATTAATGCTGCTGAAAAGATAGAATATTTGGAGGA

>V300080312L3C002R0200835312

ACTTCCTACTTCCATACAATGTCACATGAAAAGGCGTTTCTAG

>V300080312L3C005R0350315074

GCTGATAAAAGCGTGCAAGTCAAGGAAATCCAGCAATCTCAAG

>V300080312L3C003R0271120973

CTTATTTTGATGAATGGACCTGCATCGATCGATATTGTCGCGA

>V300080312L3C004R0131042097

TTCAGTTATATATTTTATTATACATCTAGCTCATTGTTGCATA

>V300080312L3C001R0210030560

TAGTTAAATGGAAAGAAGGAAGACTAAACATAATGCGAATACT

>V300080312L3C002R0440162127

GGGAACGTGAGATCAACCAGATAGCAAAATCAATCCATCAATT

>V300080312L3C002R0570770336

CGTTTATCACCAGGGTGCACGATATCAAAGCAAGACTCGACGT

>V300080312L3C004R0411315737

CCAACTACACCAACATCTTCAAGGTGGACTTTCACGTGACAAC

>V300080312L3C001R0010623615

CAAGGCATACATTGAACCGAAACCCTGGCACTAACTGGGATGT

>V300080312L3C003R0700411623

AAATTATTTTTTGCGTTTGGGTCCAAATTATTTAACGGATCCC

>V300080312L3C006R0250781383

CCCTCAAATTCGAAAAAATCGCTGCGGAAGCACTTTTTCCCTG

>V300080312L3C004R0561236285

AATGACGTTGCAACCATTGTGGTGAAACCGGTCATGTTCGATG

>V300080312L3C006R0220010399

CTTATGTCGACTTCAGTCTCGACATTGGTTGGGCCGTGCGCGG

>V300080312L3C002R0400693044

GATAATAAGGAGAGTGACTTTGATCCTCATCGCTCGCCGTCTC

>V300080312L3C001R0340334341

TCGAAAACAGGATCCTGAGACGATCCAAAGTCTAAACTTTGCT

>V300080312L3C005R0680600226

TGATGTTTAGCAAGGCCGATGCGAGCTTAAACTGTAACGTAAA

>V300080312L3C001R0421147575

GTTGGGTGGTGTGACTGGTGGACGAGGAAAGGCGAACCTGTCG

>V300080312L3C005R0590931630

GCTGAGCGGCGCGTCTCTTTCCGTTATTTGCTGTTGCTGTCTT

>V300080312L3C004R0130047285

TGAGCGGCGCGTCTCTTTCCGTTATTTGCTGTTGCTGTCTTAA

>V300080312L3C002R0241318328

TAAACTGGATACGTTCAAAAGTGACGATGCTTTGGTTAGCACC

>V300080312L3C005R0110696862

TTGCAATGACCGATGGATACGGCTGCGTATGAAATGTGTATGA

>V300080312L3C001R0170600474

CATAGTGATGATCTGCTGATTGGTATTGCTGGGTTGTTGGCGG

>V300080312L3C001R0610747729

GTAGAGAGAGAAAGAGAAGGAAGGAGAGAAAACCCATCTAAAA

>V300080312L3C003R0540123822

ACGAAGCAGAGTTGTCGCTTTTTCAGGTTATGGAAGAAGATCG

>V300080312L3C002R0300374855

GCGTTGCTTACTTGGGTGCCTATCACTAATTTTGTCCCATCAG

>V300080312L3C004R0200281617

ACTTGGGTGCCTATCACCAATTTTGTCCCATCAGTCTTTTGCA

>V300080312L3C002R0280370120

TGAGAAATTTTATCGAGAGTAGAGGCTACAAATGTGTATATCT

>V300080312L3C005R0550456731

CCATTCGGCTGGGTTTGCTGCTGACCGCGCGGGACACTGCCTT

>V300080312L3C002R0620792356

TGGGTTTGCTGCTGACCGCGCGGGACACTGCCTTCAATCAATG

>V300080312L3C006R0571219970

CCCAGCGCAACGTCCAGATCGTTCTTCGGATCAGCCACGTAAG

>V300080312L3C001R0320810977

CGTTTCGTTGGCCGTCAAAAATACCCTGACTAGCATTCAGCAT

>V300080312L3C003R0181366254

TTCCTTCACTTTTCGTGCAATTTCCACATTCTTCCAACCATCC

>V300080312L3C003R0021360953

GGCAAACATGAGCTGCTGCTTTTCCACAGACATCGTCGTGAGT

>V300080312L3C006R0020404286

CTTGCTTTCTTTTCTTGGCACCAGTGCTCGATATCTGCACCGC

>V300080312L3C002R0651224912

ATCTTTATCGAGTTTGCCTAGCATATCGTTGTCGATGATACTG

>V300080312L3C004R0290092768

CAATATAAGGAGATCTCTCCGCTTTGCATTTATAACTTGGCGG

>V300080312L3C004R0480919808

CGAGCCTTATTTCGGATAAAGTCTTGTACTTCAGCATCTGTCT

>V300080312L3C002R0310593276

CACGCCGGAAAGTTTCGAGTTGCCGAGCAGTCTCTTCAGCATT

>V300080312L3C006R0640328222

GATATGCCTCTTCAACTTGAGCTTTCCGTTCGGTGTCTGGAGC

>V300080312L3C004R0631203759

AGTTGTTGTCTTTGTCAGGCGTACTTGCTGTACAGTGAATGTC

>V300080312L3C005R0690499982

GCAACAAGTATAGCCGGCTTCCTGCCATCCTAGCACTTTCAAA

>V300080312L3C003R0431195222

AAAACGACAATTGCAGCGACATTAATTTTTGAAAAAAAGTGTA

>V300080312L3C003R0720665328

TTATATTTTAGAAGCCGTTGGAAACTTCAGTGCATTTTCATTT

>V300080312L3C004R0690056731

TTACAATGTAGCAATCCCAGATGTTTATAAACAAATAAAAGGC

>V300080312L3C004R0060178269

AAAAAGGAGTTGCTTCCATTGACTACGATGATCGTTTACATTA

>V300080312L3C003R0351210679

TAAACAGCATGTACGCGATGGATTTCGAATCGAAAACATGATG

>V300080312L3C002R0410306133

CCACAAATCATAGCAACAACTAGACGAAAATTTTTCATCGACG

>V300080312L3C003R0240853797

TGGCCAGCGACCGAGTCTGCCTTCTCGTCCTGCTCCTCGTTGC

>V300080312L3C002R0150451550

AAAGAAAGAAAGGAGAAAACAACTCGAATCCAAGCGCCCTTTT

>V300080312L3C005R0360753827

CAAGAACTTCTCGGTTACATCCATGCATACAGAATCAAGGATA

>V300080312L3C002R0480492044

TAACTTCAAAGTCAACATCCTCGCTTTACCTGCCCACCAGATC

>V300080312L3C003R0261317734

TTTGGTGCTCACAGCTATCGTTAAACGTTTTCATAATTCCTTT

>V300080312L3C001R0430155742

TAACCATTAAGCTACACCAAGCGAATACGTGTCTTGTTTGGAA

>V300080312L3C001R0151234060

GGAACCACAACAATATTTTACCGATGCACGCTATAATTGCGGA

>V300080312L3C005R0120319977

AATATCGTATTTCTGCAAGTTGTCAATGTAGCAGCATGTCGGT

>V300080312L3C006R0591097628

CAAGTTGTCAATGTAGCAGCATGTCGGTCTTTTTTGTATTGTA

>V300080312L3C004R0300855215

TCTGAAGAAGGAATTCTACGCGTTGTCCAAGCTGAGGCTAACC

>V300080312L3C005R0520483946

CCCATAACAGGATTGGCGGATCATAATAGTTACACCCGAGAGC

>V300080312L3C004R0610407244

GAAAAATTCACCGCCGATCCAAGATTAGCAACACAAACCAAAC

>V300080312L3C001R0491288232

TGCTCTTCTTGATCAGCATTGCGGCTCTTGTGAACGCTTTGCC

>V300080312L3C005R0121152300

CAGAAGGCACTGCTGTCCCGTTCTGCACTCAGTCTAACCTAGC

>V300080312L3C002R0050871524

ATGTTGTTTGCATTGTAACATAAAAGCGATGAAATAAATGCTC

>V300080312L3C005R0560781004

CAACCGCTTTTGCATTCGATGCTGCTCCAACAAGTCGGACTGC

>V300080312L3C002R0040345693

GCTCCATGCGTTTGCTCGAGACGTTCTCTTTCCTCGTAGCTGC

>V300080312L3C005R0370402247

TTAAGTGATTATTGGATATACTTGCGAATTGGCACTTGCATTT

>V300080312L3C003R0440388868

CCCCCCTGAAGCGCATAGTAATATTCTTGAATGAATAATGCAC

>V300080312L3C002R0620122540

CTGGAAATGCAGTAGACGGATCACTCAATTCCACTACTTGAGC

>V300080312L3C003R0721171462

AACGAGGTAATGTTTGGATAGAACGATAGAGTCTGTATCCGTG

>V300080312L3C005R0050885023

TATCTTGGGTAGATGGCGACGAACGCTTTCTTTCAATAACGAC

>V300080312L3C005R0461173120

CAAGCTTGTCAACAGCTTTGATTTGTTCTTGAGGGATAACATT

>V300080312L3C002R0481128698

GTGAGTAGCGTAGGCCCGCCGAAGGTATGCCTTTTCCATGGAA

>V300080312L3C003R0261138840

CTTGCGCGGCTTGACGTTAATGTTCATAAGAACGGCGACAAGG

>V300080312L3C006R0700474536

CGTTCTTTTTGAGCAATAAAGAGGGATGGAAGGATGGCTCTTG

>V300080312L3C006R0500351665

TGCGCTTTCGCTACCTTGCTAAACGCGCGTTTCCTTCGACTTA

>V300080312L3C002R0340835029

TTGGTGCGCCTTGGGCAGTAGTGCTTTGCGGATTTGCGTAAGA

>V300080312L3C005R0660927365

TCTGCCATATCTGACGGAGACGACTTGCTCTTCATAATTGAAT

>V300080312L3C005R0140822751

AATATCCTCCTTAACTTTGCTTTATACTAACATTTCCGAAACA

>V300080312L3C006R0591084826

CAGGCCAACCAGCATCCAGCGCTATCTTTACAAATTCCACGTC

>V300080312L3C002R0171354428

TCCCATATCGCTTTGTTCGCTGACTGACAAGTGAGCGCCCGCC

>V300080312L3C003R0381249062

CATATTTGTAATGGAAGTAAATTGATCTTTCAAAGTCGCACTG

>V300080312L3C005R0710471393

GGGTCATGGAATCGTATCAAGCGTGCGAGTGATTTGGCCAAGT

>V300080312L3C002R0680587224

TGGTCAAAACTCTCTGATCCAGTGGTTTCGCCGACGAAAAACA

>V300080312L3C004R0451060452

ATCGTGCTCTGCGGTATAATATCGTAGTCGGGAGCAACAGCTT

>V300080312L3C005R0081252543

ATTCTTGTCAGTGAACAAAACTCGTTTTAGTTTTGATTTTTGT

>V300080312L3C003R0340425870

CGAGGATCTACTCGAGGTGATTTTTGATAAAAATAAACGATTT

>V300080312L3C004R0450557508

CTTTCACGAAGAGCCATCAACTCTTCGTCCATGCTTCGTTCCA

>V300080312L3C003R0300657445

TGCATGAGTCGCTTCGACTTGTCGAATCATGTGTGCAAGCTTG

>V300080312L3C004R0441104374

AGGCAGGCCAATTCCAAGACCACCAACCAGTGCCCAGGTTACC

>V300080312L3C003R0200800370

CACTGTCGGCCGTGTGTTTGGCGACTTCCTCGACGGTAAATTT

>V300080312L3C003R0670441984

CTGGTAATGAAACATTTGAACATCGACAAATGTTCACAACTTG

>V300080312L3C003R0190214820

ATGAGGTTATTGATTCCATCAAGAGCAGCACAAGCAATCCACA

>V300080312L3C002R0220030870

AATGCTGATGAATTTCTAGCTTTCATCAACAGTGTTATGGACA

>V300080312L3C004R0100187790

GAATACGTAGAATACCATCAAGTCATCAAGACAAAAATCCGCC

>V300080312L3C005R0471014303

TATCTTTATCCGCCTGTCACAAGAGATTAGTGTAAATCTCCGG

>V300080312L3C002R0050492860

CCTGTCACAAGAGATTAGTGTAAATCTCCCGGCGACTTACGGC

>V300080312L3C004R0630727138

ATCGGATCTGCCGGATCCCTATCTCCTCATACAAACTCCTATA

>V300080312L3C005R0260644043

AAAGGTAAAACCGTGCCCATTTCAGAATATTGTCGTCTTCTCC

>V300080312L3C002R0470560489

CCTCGTCCTCTTCTCTGCCCATTGCGTTTACTTGTACAGGAAA

>V300080312L3C004R0100385693

AACACCAACTCCGCGTTGATACGGACACTTCCGCTGATGCACA

>V300080312L3C003R0370928536

GAATAAGAGAGTAAGAGATTTGGTTGGATGCGAGCTGAATGCC

>V300080312L3C006R0400721264

CATCGCGAACTTCTCACGGCTTGCTGGTTTCTTTTAACGTTGT

>V300080312L3C002R0710364989

TGGAGCGTAACCTCCTTCGTTTGCATGATTATCAAAACAATGT

>V300080312L3C001R0200339726

TTCCCATCTCGTGCTTCGTGTAGTCTTGAGCTCTATCGACGAA

>V300080312L3C003R0131235728

CTTGTTGTTGAGGATGGCGCATAGGGAATATATCTGCAACTAC

>V300080312L3C001R0470477188

GCCGATACGCTTATTCCGGCGCGACCACTTCCCATAAGGAAGC

>V300080312L3C003R0160005331

CCAGTAGGGAGTCTTGATTCAACTGTCTCTCCTTCAAAGGTTG

>V300080312L3C001R0670457928

ACCCGTTATGCGACGAATGTGAGGCCAAGTTGGACAGATTTGC

>V300080312L3C005R0181071916

CCGTGCACACCATGAAGCGATCGATAGCGTGCCTTTACATGCA

>V300080312L3C006R0580236548

AACGATTGCGTTACCGAAGGAACCCTTTATACAGAGTCCAACC

>V300080312L3C005R0291021097

GGCATACAGGGCAGGATCGTTCAAGACGATATCAACGATGCGA

>V300080312L3C001R0570005018

TTCACTTTTCCCGGCCTCTCTCGAATATTATTTGATTTTCATG

>V300080312L3C004R0020190466

AAGGTTTGAACGTTGACCTTCTAGTCTGCGCAAGACAATTGGG

>V300080312L3C005R0250873886

GGAACACTCTGACACGGAAGAGATTCAGGAGCCAAAAAACCGA

>V300080312L3C006R0590218061

TCGAAGAGCTCGCATTTCAAAACAGAATAGCTGCTATGGCACC

>V300080312L3C001R0431117481

CTTGAGTGGCGTATGACACGGATGCTGCGCGTTCAACTTGAGT

>V300080312L3C006R0110034707

TTGTTCGCTTTTCTTCCCTGGATTGTCGCAGTGGCTGCAACTC

>V300080312L3C005R0451317003

CTACTGTTTTCACTATCTGTGATTCCGGCCACAAGCATGCTGC

>V300080312L3C006R0351220744

TGGCATGATGAATAGAGACCAGTTGCTTATGCCTTACCTTTAC

>V300080312L3C003R0080625988

CACACCGAGAGGTACCATTGCCACGAGAAGGGCTATCACGATT

>V300080312L3C005R0161311016

TATAAATCTATGTAATTATACTTGCTCTAAAATACAAAATGTT

>V300080312L3C005R0400837747

GGTACTGTTCATAGGCTGCTTCATTGGGTGTCTGTTCTCGCTC

>V300080312L3C006R0050252145

TGCTTCCTCTTCTTGGCTGGCGGTTGTGGCAGTCTCAGGCTGC

>V300080312L3C006R0630164859

GCAGGCTCTAGCCGAGGAGATGTATCCGGCAATCAAGGCAACA

>V300080312L3C001R0160974560

TGTATCCGGCAATCAAGGCAACAAGCGAGAAACTGAGCTCGCA

>V300080312L3C004R0130948063

CTGGTACCTGTAATTCACGAAGTCTTGATACGACTTCTTCTGT

>V300080312L3C005R0200496425

TAGCATTGTTGAATTGTATGTGCATCGGCTTTTCAAAGGTAGA

>V300080312L3C001R0521043803

CACAGCGGATCCGATACAGCCAATAATTGATTTCCTTGAGCCC

>V300080312L3C001R0051208997

TCTGTTGATAACGACAATCATTTTTAACTTTCAAATCTTGCTG

>V300080312L3C006R0680498063

AAAAATGGAGCATTCTACGGACGTAAAAGTAGCAGATTATAGG

>V300080312L3C005R0461020847

GATAGCCGTCTCTATGTATTCTGGGTTCCGTTGTTGTTAGGTA

>V300080312L3C003R0300009330

CAAATTTTTTTCCTCTGCGCCAATCAATCTTTACCCTTTATTA

>V300080312L3C003R0121027281

GATCGCTGACAAAGAGCGTATAATCAATGAGGTTGAGAAAGGC

>V300080312L3C004R0350804917

ATCACATTATCCTTCTAAACTGCAGCGGTGCAATCATCCGTCT

>V300080312L3C001R0131005588

TATGCAGCTTGTCCCAGAAAGCATACAAGGACTATGCTATGCA

>V300080312L3C005R0260769297

GGCCGATACCATACTCGGCATCGCAGATACGATTGAGCGAACA

>V300080312L3C002R0631234994

CATTCAAGTTTTGAACAGGCAGTTCAAGCAGCAAAGCAAGGTG

>V300080312L3C001R0060523695

TACTTCTCACGTATCCTTTCTTTGGCAATGTTACAAGCATAGT

>V300080312L3C002R0440844435

ATGCGTTGCAATACGGGTACATTTATTTTGTCGTATGCTTCTA

>V300080312L3C003R0170296567

GATGATTCGATTTTGTTTGTCAGCATGTATGGATATACAGAGG

>V300080312L3C006R0400471986

CATCTTCGCGAAAGTAGTACTTCTGGTGGTCCAAGGTAATGTT

>V300080312L3C006R0420778952

ATATCTCAAGAAACCGCCTTGCTTCGCTGCCATCCGATATCGC

>V300080312L3C003R0260491781

CACGAGCTATCCTTCCGCGCACGCCAGCTAAACGTACGAGGCT

>V300080312L3C002R0600360976

ACAGATATTGAGATTCCAAAACCAGAACGAACCTATCCTGACA

>V300080312L3C006R0021337149

CCCAAAGACGGCGCTTTTCGTCCGAGGATCAAGTACAAGCCAA

>V300080312L3C006R0340546982

GAGTGTTGGGTAATAGACTATTGATGATCCGCTTTATCTCCCC

>V300080312L3C002R0300537124

TTTTGCATGCACGGCGGTCTTTCGCCTGATTTACAGAGCATGG

>V300080312L3C001R0720795481

AGTATCCTTTACATTTGGACCAGACGTTGTCACCAAATTTTTG

>V300080312L3C002R0431101497

TTACATTTGGACCAGACGTTGTCACCAAATTTTTGCAAAAGCA

>V300080312L3C002R0220376544

TTTTTGCAAAAGCATGATATGGATCTTATATGCCGTGCTCACC

>V300080312L3C003R0580731893

TGCCGTGCTCACCAAGTGGTTGAAGACGGTTATGAATTTTTTG

>V300080312L3C001R0610985145

CACCTTATCTGAATTAGATCTTGAAACCGGCAGAAAAGAGATC

>V300080312L3C003R0231318278

TGGATAGCATGCTTGACAATGCGCTGGTCCAGATCGTACCAGT

>V300080312L3C005R0020956763

AACTTTGCTACTATTCTGGACAGTGTAAGTTAAAATGTTCCGC

>V300080312L3C001R0621390318

TACCATACATTAGCATATCGCCTGCATACCCCTCTTTTGATGT

>V300080312L3C002R0470236921

CCCCTCTTTTGATGTCTTCTACTGCAAAAAGCAGCTATACGTT

>V300080312L3C001R0610479020

TCAGCGAACGTGGCAAAACAATAGAACGCCCAAGCTCGGCATC

>V300080312L3C003R0510448070

GTAAGTGTATTGTCTCCACGCCTAACCAAACATTGCATGATAT

>V300080312L3C003R0041307819

GGACGTTTTACCAGATCGAAAGTCCCACCCAGCAAGATTGGAT

>V300080312L3C005R0521275596

TGATACAATCTCGGACTTGCTCTCATCAAACTCGAGAAGACAG

>V300080312L3C003R0330708634

ATTAATGCAAATTACACTGCATAGATTGCACAATCCATTGTTT

>V300080312L3C001R0720680196

GGAGATGCAAAATGAGCTGTACAATGCCTGGTCGAAGTAGCAG

>V300080312L3C004R0600556964

TGGGTGGGAAACAAGAATTGGAATAGGATTATACGAACCTTGC

>V300080312L3C002R0450896169

TATACGAACCTTGCTAAAGTGGAAAGAACGAAAGAAAACCGCG

>V300080312L3C006R0511176140

TCACCAGTACCCTCCTCCTTCGTAGCAGCCTCCTTCCGGTGAC

>V300080312L3C003R0210690162

CTCGTGAACATCATGTTTTCTGTAGCATAACAATAAATTATTA

>V300080312L3C001R0450891544

CAGCTCTTTAAGGAAAAAGGTGGAAAAAAAGGGTAGACACGGC

>V300080312L3C002R0431325991

CTCACGATAATCGTCAACTGTGCATAGATTTTCGGTGGGTATC

>V300080312L3C004R0631352298

CCAGTTTTAATCTTGTTATTCTTGATATTTGCGAGTGTTTCCA

>V300080312L3C002R0521322708

AACAGATCAGAGTGAAAACTGAAACATTTTTTGAATTTTCATC

>V300080312L3C005R0330840971

TATAAAGTGTACACGTTTCTGTATTAATAAACATGATTGGCTG

>V300080312L3C003R0030379593

TGAACAAGCCGCCTGCCAATCTTGCCGAGTTTGAGGAAAAACT

>V300080312L3C001R0241260854

TGGAAAATGCGCGTTGGGGAAACCAACCACCTTGCTGAGATAA

>V300080312L3C003R0500453588

AAAGACTTGCTTGGATTATTGGGATCTGTGATCGTTACAGTGG

>V300080312L3C006R0020977161

TTTGGTTGGCATCCGATTGGAAGTCCGCATCCCTTTTCCCGGT

>V300080312L3C001R0660260106

TGCTTCTGGCTTTGTGAAGTCCGCAAGTCAAAAGAGCGGTCAG

>V300080312L3C005R0330246145

CGTAGAGGCCATTGAACGAACGCCCGAGTATGAAGAATTCATG

>V300080312L3C001R0500569960

GAGAATATTGCACCGCAGATCATGGTAGCCAATCCCTCTGATC

>V300080312L3C005R0470374629

AACTCATCTTACGGAATATCAAATACGCTCCCAAGAAAGTACC

>V300080312L3C004R0721073930

ATTGAACGGCGTCAGTAATTCTTTGCTGTAGAAAGATTGCTGT

>V300080312L3C005R0260494892

AGTAGCAGCCGGTCGAGTGACTACAAGTTAACGAGTCAGAATG

>V300080312L3C006R0460810013

AAACCTTGAAAACTAATGGATGGAAGTACTGAAATTGCCGCTG

>V300080312L3C003R0300720464

AACGCAATATCGGTAGTGAGAATTGCGAAGCGAATCTTGCGCA

>V300080312L3C004R0120742539

GCTAGCAAGCGACTATTGAAGACACACAAGCAATTACTCACAG

>V300080312L3C002R0170461337

TCTGCTGAAAGCACACTCTTTATATGACAAGGTACCTATCTAT

>V300080312L3C004R0251098842

GACGCTGGAGACGGTTATAGCCGTGATTTTTTTAGAGTATGCG

>V300080312L3C005R0430461213

GTCAGCAACAATTACAACTGCATGATTTCCGTCTCTCGAAGCA

>V300080312L3C001R0300215082

AGAAGGAAAGAAAAAGAGGTAAGGGAAAATGTTTTATACAAGG

>V300080312L3C005R0710248862

CAGCGATTTTGTAATATCTTATTTACAGTATCGATCTGATTTG

>V300080312L3C002R0200586673

CGGAACGTCTCGCAGAATCCAATCTCTTCAGAGTCGGCACGTA

>V300080312L3C005R0541063337

AAGCTTGACTCGCGCAATCTTGGCATCGTCGTATACTTGCATT

>V300080312L3C001R0400868636

GTTTCGATATTACACAGCCCGGCTCTTCCGTGGCTACTAGCTT

>V300080312L3C002R0470153620

ATCAGACGACTATGTGAAGCGGCGAGCGACAGCAGACAAGGAG

>V300080312L3C003R0680696193

CACATTTCCGAAAGCAACGAGTGCAGTAATCGTCAAAGTCACT

>V300080312L3C002R0140249838

GATCACTCTTCCCTATTTATAAATGCAGAATTGAGATTTAAAA

>V300080312L3C002R0180619829

CCGATATCGTGTTACAAGATGAAGATTACTAGTCTCGCTGACA

>V300080312L3C003R0290443862

ACAGCCTCCTTTTAAATCTCGAGCAGTGGACAGGTATTACAAA

>V300080312L3C002R0040109599

AACACAACGCAGCATTATATTTTTACACCTCCATCTTGTGATT

>V300080312L3C006R0601307501

ATGCAGCTCGTTATCGCATCTTTTTTTTTTCGACATCCGTAAG

>V300080312L3C005R0710894809

TCTTCTTTCTCAAACGTTTTATGTCTATTTTTGACAACCTGCG

>V300080312L3C005R0110955161

ACCCGGCTAATACGGTGGCATCAGTGCCAGTAATTTTCTCACA

>V300080312L3C001R0110488283

TACTTCGAATTTTGCTTGCATTGTAATGAATCTCAATAACGAA

>V300080312L3C001R0621296307

AGACTAGTGACCAATGTGGCAAAGAGCAGGATTGGAAGGATAG

>V300080312L3C004R0390538221

CCCACCAGTCAAAACTGGTCATCCTCTCAGATTGCCGGTCGTA

>V300080312L3C003R0451327890

TCGGAAACAACAATCTCCTATCCCGATCCTTTCGCCAGCACTC

>V300080312L3C006R0210671030

CGTTGATTAACATTAGCGAGTTCCAAGCGCTGTTGACAAGGGA

>V300080312L3C004R0271039156

CACGCAGGAGTATGCGAGGAGCCAATCTGATTTTAAGAACATG

>V300080312L3C004R0290711500

TTGGTCGCCCCTGTTGTAGCTTGGGGCATCTTCGTATGACTCC

>V300080312L3C003R0201357571

CCACAAGTGCTATGCAACCAACCCACGCACCTTCTCATAATCC

>V300080312L3C002R0431135239

AAAAATTATATTGTGTTAATTTCCCCGAGGACACTATTGATTT

>V300080312L3C001R0220000873

TTTATTGACGAGAGAGTGGACAAGCACTTGCGTTGCATCGGCC

>V300080312L3C003R0510338529

GATCTGCGGATCTTCGGTGACGGCTTTTACCCTTTGCAGATCC

>V300080312L3C003R0571104098

GATCTTGCAAGGAGCTACTGCATGGCTGCATACGCCTGCAGCA

>V300080312L3C003R0190569298

CGAAAATGCCGGTAGCTGCAAGCGTCGTACTACTTGCGATAAC

>V300080312L3C001R0220210736

GTGCTAATAAACAATACAGAATTAAGTAACGAAACGAAAAGGT

>V300080312L3C002R0461295354

CTTACATGAACGATTCTCGTAACAGCAGTAATTTTGGGTCTGG

>V300080312L3C001R0251142674

AATAAAATAGATTTTTCTTTACTATTTGGAAAGGAATGAGATG

>V300080312L3C005R0471402040

TGCCTTTTTTAGAACATCCGTCCAATCAATACACTTCTCTGCC

>V300080312L3C003R0530192694

TGGTTCGTCTTGAAGCATACCAGATCAAATGAAAAAAGGGCTC

>V300080312L3C005R0210207524

CATCCGACATGGAACTACGCATCGAGATTTACCAATATTCATG

>V300080312L3C004R0561003950

TTTTCCCTCCTTCCTTCATCATCCCACTGACATCCGAGGAATG

>V300080312L3C001R0080066320

TCTCTCCTTGGTTTTTTATTTTTATTTTTGGACTATAATAATA

>V300080312L3C005R0620896921

TCAGTACTGCAGCCGGGCCACATGATGGTTGCGGCGCCTCTGC

>V300080312L3C005R0200747039

TGCAGGCGCAAATGTATGGAAAGTCATCATGTAAATCATTTCG

>V300080312L3C006R0441083668

GGTGATGCAAAGATATCAGCTAAAATGGAACTTGACATTCTTG

>V300080312L3C003R0221360958

CATTGATCTCTTGTCGCATATATCGCAAAGCTTCTTCCCTAAC

>V300080312L3C002R0371280488

GCGAGGTCTTTATGAATCCCACTGCAAGGAGCTCGAGTCAGGC

>V300080312L3C006R0310610287

TATGCCTTTGCCGTGCACTTTGGGTACATGTCTCCTGCAACAT

>V300080312L3C002R0071139186

AAGAAGGTAAGTTCTTTAGGTTGCCCTTTTTCTCGACAATCGA

>V300080312L3C002R0351039767

CAGGAAAAACTGATCCAGGAACTGCGAACACAAAATGAGAACT

>V300080312L3C004R0261272740

CTTCTGGTGCATTGCAATACTCGTTGAAGTTATGTGTATCTCT

>V300080312L3C001R0510976756

TCAGTTTCGGGTCTGTCACAGCCATCATGCGGTATCCAGCCTT

>V300080312L3C002R0550781186

CACACCAGCTGCTCCTGTCAGGATGCCAAAGCTGAGGGCATGA

>V300080312L3C003R0610383470

ATGTTTGCTTGCAAACAGAATGCTGCGCCTGGACCGTGCTGGG

>V300080312L3C004R0260933532

CGCAGGGCATGAAATAGTTTTGGATTTCTTCTTCTTTGAAATC

>V300080312L3C006R0520631980

TCGCCACAGTGCTTGCAAATAACTGTGGGTGAAATATATCAGT

>V300080312L3C002R0470490562

GTTACGATCAGAACTCAGGATCGAAAGTCGTTCTACTAGAGTA

>V300080312L3C002R0540041707

AACCGCTTTCGGATGCTTATCTGCAAATCCGGACTGGTCGATC

>V300080312L3C005R0491353968

TGCTTATCTGCAAATCCGGACTGGTCGATCTCTATCCGCCAGC

>V300080312L3C006R0571168469

TCTATCCGCCAGCACGTTTTTGGGATAATCTTTTCAAGTTGCA

>V300080312L3C004R0160793261

AATTTGAATTCAAAAACCTGCAGCTTTGGGCATTGCTCAAATG

>V300080312L3C002R0700942046

CCCAACCCGGAAAGGAATGAAAGAGGCCTCTAGCGAAGCCAGC

>V300080312L3C001R0571214776

ATGATGTGAATTTAGGGCAATGAATTGAGCCTGTATATTACGG

>V300080312L3C006R0430329065

GCAAAAAGTTTTCTGCTTTTCTCTTCAAAGGATGTGTAACTTA

>V300080312L3C001R0700042503

CGATATCTTCCGCATCTGTGCTGCCGAGCACATCAGACTCTTC

>V300080312L3C002R0640539935

CTTGCAGGCCACGACCATCGGCCGCCAGGCGTTTATCCAATGG

>V300080312L3C001R0480033077

TCAACCATACGGATGATACGTTGCTTTGCACCGCTGTTCAAGT

>V300080312L3C004R0300723233

CACTGGGACGATAAATGAGATACTCCGCGTGCTCATTGGACTA

>V300080312L3C005R0070083941

TGCTCATTGGACTAATCACCTTGCTTACCATCGTAACATCCGT

>V300080312L3C002R0410106370

AAGAAGTCTCTTGACAGCAGCCCTTCTCGTTTAGCGTACCTCG

>V300080312L3C001R0720040848

AATCCCCAGGAACCTTTCGGCGCAGGTTGTACCCTGATTGATG

>V300080312L3C005R0681056874

CCACTGCTAGTCCAATGCCTAATCCTTTGATAAAGCTTTCCTT

>V300080312L3C004R0700140770

TGCACGCCACTTGCAGAGTCCCGATTTCGTTCCCTTGCATATC

>V300080312L3C003R0531312026

CAAGGCGCTCTTGCTCCGTAGGACTATCAAACTCCTCTTCTTC

>V300080312L3C004R0720286821

CTTCTGCGCCGGCGCCGATGAACAGTGGAGCGTCGGCTGTTAG

>V300080312L3C006R0340325426

CCTTGGCTATCGGTATGCCTGCTGACGTCTTGAGTTTCAGAGG

>V300080312L3C005R0680612550

TGATTCTGGCATCAGTATCCAAGCTACCAAGCAACGCGTTTTT

>V300080312L3C006R0411176685

CAGGAAACGCGTTTCTTTATATTTGCAAAGCTTGCTCATGTTC

>V300080312L3C003R0690635161

AACTATGCAACACGTAGCAATTTCTACACTTTTTCCACAGCCG

>V300080312L3C006R0190512946

TACAGCCAGGCAAATGTTTTCTCAACTCGTCGAAACCGTCCTT

>V300080312L3C001R0401121220

GTGTTGTAAATTTGCAACGAATGACTGGAATGGATAGCTGGCG

>V300080312L3C002R0660628327

GGTGGCGGTCGACGCAGCATCCGGTTCCGTCGGAGACGGTGGC

>V300080312L3C004R0660177067

AGTCATTCAGCTCAAACGATATGCTCGCTAATGTATTTAAAGA

>V300080312L3C001R0130756934

TGGCGGAGCACGTGTTGACAATTGCTGGAAGGACAGGTCCACG

>V300080312L3C002R0390931387

GGACTGAACATCCAAACAATACGCCACTGCATGATTATTTTCA

>V300080312L3C003R0510180061

ACCATAAGTATGGCTACCGATTTGATGACCTTGGTCATAGACA

>V300080312L3C002R0060655495

TAAGTATGGCTACCGATTTGATGACCTTGGTCATAGACATGCT

>V300080312L3C002R0100913056

TGATATACATTCATCAATCTGTGCATTAGAGACTCTGCCGGAA

>V300080312L3C001R0681216994

GCCTTTCTTACAAACTTGTCTTCGCTTACAACTCTCTTTCCCC

>V300080312L3C001R0700498219

CCTTTCCTGAAACGGTTTCTCTTTGAAAGACGAAAATGAATTC

>V300080312L3C003R0680923722

GATTCTGTGCTGATGTGCGGCGTGGTGGTAATTATGCTCTATT

>V300080312L3C004R0061076117

ACCTGCGAAATACCTCTTTGGATACCAGCAGAGCGAAGATATC

>V300080312L3C001R0150284616

TGGCCACTACTCTCTCTTGTTCTTCATTTTCTTTTTAGGATTC

>V300080312L3C003R0620770323

AACAAATGACCGTTGACACGCCAGAGCTACACATCCAGATCAG

>V300080312L3C002R0430125245

CTGACACCTGTTAGCTTCCATTGGGCATGCAAAGCAGAGGTAC

>V300080312L3C003R0530004701

CTGGTCCTCCCAAGATCCGGCAGCGTAGAGCCCACGAGGGGCA

>V300080312L3C005R0460124651

TGGCTGCTGCGGCTTTTCCTGAATGCTTGATAAGCGAACCTTA

>V300080312L3C002R0180306744

CTGTTTGAAAAGTCCATCGTTGGACACACCCGCCTTTTCTTCG

>V300080312L3C003R0330362652

GTTGGATCCAGTTGAGAAGCACGGCGATGCTGTCTACGAGATG

>V300080312L3C005R0181344313

TGCCCGAAAAGTACAAGCAACTCAATAGTCACCTTCCTCACGC

>V300080312L3C003R0680889529

GGAGAAAGGAAAGCGGGAATTTGAGGCAAGGTGCAACAAGACC

>V300080312L3C001R0410400897

GGTCCGATACCAGCTCGCATTGAGCCAAGTTTAATGTGCGAAG

>V300080312L3C001R0330473034

CGTGCGATCGAGAAAGAGTTTGGTAAGGTTGACTAAACCTGCA

>V300080312L3C002R0171148233

TTATAGATACTGGAGGAAGGAGCAATTTGAATGAGAGTTAGCA

>V300080312L3C003R0420963439

GCCATGTATGGCTTCGTTCATTTCCGATTGGGTGAGGGAAAGG

>V300080312L3C002R0160068233

AAGCTTACCTTCCTCCTACATATTATCCTAGTCTGTTTCAAGA

>V300080312L3C003R0491047643

AAGTGCCAGAGAGCACAGAGATGACAGTGAAAATCTTATTGTC

>V300080312L3C002R0261199933

CCAGAGAGCACAGAGATGACAGTGAAAATCTTATTGTCCTTTT

>V300080312L3C004R0300916253

ACTCTCAACTTTGAAGGAAGGAAGATGAGTCATCCGGCTACGT

>V300080312L3C001R0631032905

TCGCCTTTGGAGACAGCAGCTGATGCCTGGGACACCAATGTCG

>V300080312L3C002R0721363414

GCCTTTGGAGACAGCAGCTGATGCCTGGGACACCAATGTCGCC

>V300080312L3C002R0180732558

GATTCTTGAAGATTACGAACAAACCTTACCTGTCCACCATGCT

>V300080312L3C006R0140705927

TGTCGTCATCTACTGCCACCGTCGATGCGTCATCATCCTTTTT

>V300080312L3C006R0050775790

CACGGATATGTACAAGTCGTACGCAGCACCGTCCGGGTTGATA

>V300080312L3C005R0520965287

GAAATATCTCTGTGGGCAGTGATGTCAAGATCCTGTCCGCATA

>V300080312L3C003R0010284481

AACTTGCGATCTTCTATCGGTTTGCCTTGCCAATACAGCTCCT

>V300080312L3C002R0351222380

GAAAACATGTTCAAGACCGAATCCACATCGGGAACGTCAGATA

>V300080312L3C004R0301380195

GGCGGGCCAGGAAGCCGGGAGGGACGAGTGTCTTTAAACATTG

>V300080312L3C002R0590791165

TTGGAAAAGAAACCCGCCAGGCTCGTATGGTAAGCAAAGCTAT

>V300080312L3C006R0060541312

ACGTGTCACAAACTTTTGAACTTTAATGGAAGATAAATAGCAT

>V300080312L3C001R0020345660

GCTCCGACCGCGAAGGCAGGCGCCGAGCCGTGGCCCATCGCGG

>V300080312L3C004R0660664146

CCAGGAAAAGCAGCCGTTTCTTCGAGTACAAAAGCCACGACCA

>V300080312L3C003R0261139068

ACAAACTTATCCAACGCCAATGATTCTTTTCTAACGTCCCAGC

>V300080312L3C004R0511130184

AATGGAAAGATGGCCTGCGTAAATCTTCATATGAACAACGATT

>V300080312L3C004R0520370699

CAATATGAACGATAACATTTCGTTTTTTAGAGGATCTTCTCAC

>V300080312L3C001R0610596017

TGCATGTGCAGGCCCAGCAGCTACAGTTTCTCCATGCAGCTGA

>V300080312L3C005R0231262421

GGATTAAAATAAAAATTGCTTGCTTTTTTATGCGGAGGCCGGA

>V300080312L3C006R0400104997

CTTACAATTGTTTAAAGAGGCTCTTCTACGGCCAATCTCTCTG

>V300080312L3C003R0271017654

AGGTAAAGACCAAAGAGAAGTCTCTTATCTACATGGACATTGA

>V300080312L3C006R0240236751

CAAAAGTTGGTACAGCAGGCCGCCGAGATTGAGCAGAAGAAGG

>V300080312L3C006R0100244170

GCCTGAAGGAGATATCTTACGTGAACGTTATGTCAGTTTCCAG

>V300080312L3C005R0310957789

TCCCTGTGTTGGGCATGCTGCTTGGTAACTCTATGGGTACCAT

>V300080312L3C006R0400244045

CATTCTCTTTCTAAAGCTAATGCATAATTTCTCACTTTAGGTT

>V300080312L3C001R0590921236

ACCAAGCATTTCGTTTCTTCCTTTCTCACCTTTCTCTTTCTAC

>V300080312L3C004R0290662522

GGCAACATAACAATTACAGTGCGTTCGATGAAACACTCAATTT

>V300080312L3C006R0040973645

TATATCCATTCTCGCCCTCCAGCGCATCAACGCAGCACCCACC

>V300080312L3C001R0720648517

TGAAATAGGAGCATGTGTCAGCGAATGGAATGCGCTCTTGTAC

>V300080312L3C004R0610056012

TGCGAGATCAGATCAACTATCTGTTCCAAGCACGGCGACAATT

>V300080312L3C003R0460273847

GGATTTCGGCTTTACATCGAAGCGTCATCCACTCAACAATCAC

>V300080312L3C003R0410472890

CCATATCATCACCGTCGCTATCGACATTATCGAGACCGATATC

>V300080312L3C005R0030838189

CACCTCAGCAACAGTGGATTAATTAACTGCTATCCATGCTGCG

>V300080312L3C006R0230881614

TATTCGAGTTCTTGTCGGACAAGGATTCGAATCTAGAGTCGGT

>V300080312L3C006R0430185401

ACAGCAGCAACAGCAGCATATCAAGATTTCCAAAACGCAGCTG

>V300080312L3C005R0670012075

TGCTGATCAGTGCGATCGATATCTTGGTCATGACCGAGAACAA

>V300080312L3C002R0150873925

GTGTAGTCAATCCATGGACTCGATATTTAAGTTCTTGGAACTA

>V300080312L3C001R0130654764

AGCGCAGTTGGCGGAAGATATTGTCAAGAAGGAACGCTATTTC

>V300080312L3C002R0051180901

CAGCGAATATTTCCGGGTTGCATTCTACGGCCGAGGCTTTCCA

>V300080312L3C002R0170755028

GGAGCGGATACAGAATCAGCATCCGAACGCCCAGCTATTATCT

>V300080312L3C003R0470249036

CCATGATCAGGTAAGCTGAGAGAGGCATTACAATACGAATCTC

>V300080312L3C005R0621255661

GAAAGTCTGCCTAGAACAAAATTGGAAACTCGCTTGGACCATG

>V300080312L3C002R0400293552

AACCTGCCGATAGTCACTCACCAGCTGGACGAGGAAAAAGGTG

>V300080312L3C004R0690182507

TGGGAGACTGTGCTACTGTCGAGAACCCAAAGCTGCTGGAGCA

>V300080312L3C006R0630471212

GCCAGGGGGTGCATAAGCTGGAACAATGAAAATTTGTTGGCCG

>V300080312L3C004R0510557921

GATCGCACAAGTCGACACTGTATCTGGAGTGGATCGATTTTGT

>V300080312L3C005R0401264901

TGCCTAGGACCTGTTGTGCGACCCAGAGCAACCATCCTAAAGG

>V300080312L3C001R0490394607

CCTTTGATTTATATATTTGATTGTCCCTATTCGCAATCACCGC

>V300080312L3C004R0480128521

GGACAAGAGGTGCGTTATTGTTGCGAGATATATCTGTTTGTAC

>V300080312L3C005R0570447591

CTGACGGCTACAATACCCTTACTACCCTTTCCTCCTCTTCCCC

>V300080312L3C005R0290962643

AAAAAGGTCATTACTAATTTACTTCTAGATAACCTGGCACATT

>V300080312L3C005R0570472141

TGGAGGAATTTGAAGATCAGGGCCGAATGGGACTATCAAGGGA

>V300080312L3C006R0230318842

TGTCTTCAAAGTATGAAAAACCAGCATCTCAATGCAGACCGCT

>V300080312L3C006R0020388384

TGATAATCGTCGTCATCATACCGGCCGCTCAGATTGAATTGTT

>V300080312L3C003R0290587846

AAATCTACCGCCAGTTAGCATCAGGACAAGGTTATCCCGTTGA

>V300080312L3C001R0241374435

TGTATATGCACAATCATCTTTACATCTTTTTGTTTCTTACACA

>V300080312L3C003R0530712326

GCGAACCGGTGGGAGGAGAGAGTGTTCGACAGCGACGACACCG

>V300080312L3C006R0010807984

ATGTGAATGTGATAAACAGATCAGGTTTGCCAATAGCCCAGAC

>V300080312L3C001R0540515324

GGCATAGCTACGGCATCGATCACTCGATCTATCCCGCCGATAA

>V300080312L3C006R0320402470

CCTCGGAAAGGAGTACTAGATCTCCCATTGGATATAGGTCGTC

>V300080312L3C003R0131148653

TGCCACGCAAGCGTATAAACTCTCTGAGGATCAACTTTGCATC

>V300080312L3C005R0391162574

GTTGCGCTGCGACGTTGATCGTTATAAATACATCGGATTCTAT

>V300080312L3C005R0030577932

TCATTTAAACTTTAATTTATCCATATGTTACCTCTTCATTGGT

>V300080312L3C004R0380552337

GCTACCGGAATGTACCTGCACAGTAATCTTTAATGAACGCGTG

>V300080312L3C002R0380872369

AGAGACAATGACCGAACACATTGTTATACATACGCGAATGCAT

>V300080312L3C005R0410014103

ACATTGTTATACATACGCGAATGCATATAAATGGAACAAAATT

>V300080312L3C001R0150977022

TAATCATACAGGTTTTCGAGCGTGCGAATTTTGAGAACATACA

>V300080312L3C004R0321005046

GTGCGAATTTTGAGAACATACACAGAAAAGCAAAGGAGAAATA

>V300080312L3C006R0541007464

CAATTTGGCTGCAGGTGATTGTCGTAATCCAATTTGAATAAGA

>V300080312L3C001R0460139758

TTATATTCGTTGAGAAATGTGAAATAAAGCAAAGGAATGGGGG

>V300080312L3C006R0560678078

TGTTAGTTTCAAGTATACATCGAATACACAATAGACTTACACA

>V300080312L3C001R0621273610

AATCTCGAAGGTGCCAGTCAAAGCACAACTTACTGTGCAAACC

>V300080312L3C004R0181305739

TGCATGAATTACCCGTCTTGTCTTGAAATGTTGACCTGAGCTA

>V300080312L3C001R0461257114

GGCTTTAGGTCGTTGATCGTATCACAAACAGGACATCGGAATC

>V300080312L3C001R0500249170

AGCAAGGTTTCCAAAAAATTCAACTCGTATCACGTGACCCTGA

>V300080312L3C004R0431321442

CTGTGCAAAAAACGCTGACTGCATCTGCGTTTGGAAACTTGTA

>V300080312L3C004R0390582752

CTTCTAGATATTGGCAATGGCATTTGAGGTATCAGTCTGACCG

>V300080312L3C003R0700526704

TCTATAAACCAGAGCGCGCTTAGCACTTGGATCCCTCTTTCCA

>V300080312L3C006R0380078637

CGATGTAAGTGAATCATTTCATTGCAGGAGGAAGGATGCTGCG

>V300080312L3C003R0070336122

TTTTCAGTGAAGAGAGTTATCTTCTGACGATACATATCCGAAC

>V300080312L3C004R0290294989

GTTGCTTTTTCCTTCCTTGGTAAGTTGACCCATCTAGTCTTCT

>V300080312L3C001R0480327081

TTTGTTGACCTGAGAGAGCAAATGCATCTCCAAGCATCGCTGG

>V300080312L3C004R0250636809

ATCACACCTGAATGGAGAACTTTTTGTAACCAAACACTACTTC

>V300080312L3C003R0270057723

GTAACCAAACACTACTTCACGCTCGCCAAATATAGGATATGTG

>V300080312L3C001R0141160926

CGACACTGCACCGGAATCTGCATCTGACGAAGAACATCCTGCA

>V300080312L3C006R0190732482

TGTTGCTGTATAACCTCGCTTTCTAAATATACTCATATTTCCT

>V300080312L3C004R0400069602

TGGGGGAGGGGGACATAATTGCATAAGTGGCTTCAATTTTGGA

>V300080312L3C004R0471152806

ATTCAATAACTGGTTCGGGTATCATGCACATTCGAAGGTCCAT

>V300080312L3C002R0670101850

GTTTTGTACTGTGCCATAATTTCTGTATGCTGTGAACTTCGTT

>V300080312L3C001R0510365192

TAAGACATCCGCCAGTTTTGCTATACGTCGACCGGCTGTTTCT

>V300080312L3C001R0450256581

CTTGATTAAGCAAGTTGCTCCATTCGCAGTACAGAAAAAAAAA

>V300080312L3C004R0070896679

ACAATGCTAATCCATCCTTCCAGCTATATGTGAAATCACGAAC

>V300080312L3C002R0371007801

GCTCGTACGGTGCTGTCTTGCGCTGGCACCACAGCAGGAGACC

>V300080312L3C005R0250149479

AGAGATGTGTCACCAATGATTTCCTGCATACCCCCGTGTTTTA

>V300080312L3C003R0490118018

AAGAAGCTTCGATGGAATCAGCTACGGCGGAAACCTCGCGGGA

>V300080312L3C006R0071328056

CGCAACTTTTTGTTCAGATCCATACAAACTTAATTAGTCAACC

>V300080312L3C002R0610520980

AAATATCACAAGCAAGGATCCAATACTTACAAGAGCTGGATAT

>V300080312L3C003R0251072256

TATAAATGCTCTGCAAATGTTCAAGAAGCATGCTAGTCCCAAG

>V300080312L3C003R0551321397

GGCAACTTTTCAGTTTTTAAAACACCCAATGGCAGAGGAATCA

>V300080312L3C005R0650560120

CAGACAAGGTACGCCATTGGAGTTTTACACGAAGGTAAAAGGA

>V300080312L3C002R0550021985

CAAAATACTCGTCTCTCCTGCCTTGACTCCCCCCGTTTCCCAT

>V300080312L3C003R0550798397

CGTCTCAAGGCACGCAAGGGCGACGGTGACTGGGTGCCCATCA

>V300080312L3C004R0610395530

AGTCAAGGATTGTAATCAGTAAGCCCGTAACGAAAGTTGCTCC

>V300080312L3C006R0440352274

CTTGTCTTTTCCAAGATGACGAAAAAATGAGGGTTGGAGCAGC

>V300080312L3C003R0350075355

CGACCAAGACCATGAGCCTGTAGAACCCTCCAGCAACAAGACG

>V300080312L3C003R0600530312

GATACGGCGGCGTTGGCAGACGATGGGATTGATCAATATGCCA

>V300080312L3C006R0550684827

ACAGATGATAATTGAGAACGGGAAGCACCCTTATTATTGTTGC

>V300080312L3C006R0500013633

TTAGTACAGGGAAAAGGGCCAAGGCAAAATGGTATATATAAAG

>V300080312L3C005R0510550189

AACAAATCTAAACTACGAATGATAGTCGGTCTCGATTCTCCTA

>V300080312L3C004R0680733702

TTTATGCTACCAGCTACCTGCACTTTTAGAACAGCGGAAATCC

>V300080312L3C001R0460324243

GGATAAGACTAGCGAGATCACCGATGATGGACAGAATGGCAAC

>V300080312L3C002R0441083930

CTTTTCGCTACATTGCGACAACAGCACCTGTCAGTAAAATATA

>V300080312L3C006R0260158263

CCTGAGTGGGACCTTGAGATGCGGCTCCCGCAGCAGTGGGGGA

>V300080312L3C005R0071120532

GGCGCTGATATCGCCGTCGTCAGATTCTGTGGTCGTCTTCGAT

>V300080312L3C005R0560449426

CGAGCGAGGAATACAAACAGCAACAGAAAGAGGGATCGAGTCG

>V300080312L3C005R0230901806

AGCACATTCACTTTCTCGTGCTCTCTGTAGAAATATTGTTGTC

>V300080312L3C004R0511308894

GGCGAATTAGATGTGCCACTTGCCTGTGCAACCAACGAAGCGA

>V300080312L3C005R0420963334

TTCTCGACGTGCCGCCAGATCGGTTTTATTGGCAACTACCACA

>V300080312L3C006R0260216047

ACTACCAACGAAGTCTTGCCAACGCCTAAAGCGACAATTGGAG

>V300080312L3C001R0480474479

CGCCGCATGATTTTACTGGTCTCGAGCGCGCGGGCTGACAAGG

>V300080312L3C005R0021217044

CTTGCTGAAATCCATCCTGCTGCTGCGGTGATGAACACAAAGG

>V300080312L3C003R0150843641

TCGCGTAGCTCGATTATCGCATCTTGCAAAATATCAAGTTGCT

>V300080312L3C005R0400834909

GTTCTTTTCACGACGAATCTTGGCCATGTTCTCCTCGTTTTGA

>V300080312L3C005R0581151992

TTCTCCTCGTTTTGACGCAATGTATCCTTGAGCTCCTCTACCA

>V300080312L3C006R0311045280

GAAAGAGAGAGAGGAAGGAAAGAGAGGGAAAAGGTTCCTGAAG

>V300080312L3C005R0530178558

TTCTCAATTTTACAAACCCCCCTTGAGATTATATCTTTTTGGG

>V300080312L3C005R0701240136

GAACTGTATTGGAAAGACTCATCTTCAGACGGTTAAACTGTAG

>V300080312L3C006R0290647739

TGCAGAGGCTCTTCTGATTGATGCTTCTGAAAGTCTGTTGCGC

>V300080312L3C004R0501310610

TACAAAAGGTACCTGGATTCCAGGTACCCGAACTTACCCCCAA

>V300080312L3C004R0640316353

GGTGCCACTTGTAACACCAAAAATGAAAACTCTACTCTCCATA

>V300080312L3C001R0060244702

TACTGATGTGTCGCGCAGATTCGTTTGGTCCTGACGTTTCTGA

>V300080312L3C002R0211170866

CGTTTACAAAACTCAGAAACATCGAGGTGGCATATCAGATTTC

>V300080312L3C006R0421189554

CAGAAACATCGAGGTGGCATATCAGATTTCCAGGATCTTCACT

>V300080312L3C004R0300694551

GGCGTGGATCGAAGCCGGTACCAAGGGCGCCGTCCTCCTCTTC

>V300080312L3C002R0691333902

TCGGTTTGGGTATGTGGCAGACTCTCTGTATGGTTGCACTAGG

>V300080312L3C001R0061236723

CCCTTACATACACGCACCAAAACCTATCCATCCAAGCACACGA

>V300080312L3C006R0630391059

AAATCAAAAAGAGATTAATAGATACCTATATTTGCAAGGAGCT

>V300080312L3C002R0150383822

CAGTTTGTGTTTTCAAATGTTTGGACAGAACTCTGTGTAGTTA

>V300080312L3C006R0030452717

GTTTTGTAAAATAAACTCGACTTGCGCCAGTAGCACCTACAAT

>V300080312L3C005R0510647271

TGGATCAATTGCAGCACTGTCTCTAAGCTCAGCAGATCCTGTA

>V300080312L3C005R0511346760

GCGGCTTTGCAGGCGTGGGCTCTAGAGGTGCTGGTATCGTAGT

>V300080312L3C002R0450984813

AACTGTGCAGCAAAGTTGCATGTCGTAACTCAGCGCTTAAGGA

>V300080312L3C002R0441077060

CACAAAGTAACACGAAGAATGTAAAATGTATTTTGACTTACCG

>V300080312L3C005R0491198386

AAGTGTCTTTCAAAAAGCTAATATAGTGATCCGTTACAGTACC

>V300080312L3C003R0650327438

CTTGCATCGCGTTAGCTCGATGAAATTTCTCTGCAGGCATTTT

>V300080312L3C003R0590030406

TTGCCAAAGGACTTGTTGCTGTAATCGTTCCAATTCATTTTGC

>V300080312L3C006R0380869259

TGGACATTTTTTAAACGTATTATTCTGTCCAATAAAAAGTAGC

>V300080312L3C004R0650751523

GGATCATTATGACTGTGATCCGTTTAGATAATCATGCTGCATA

>V300080312L3C002R0530390565

AAAACTGTTGCAGACGGTACTACATGCTGCATCATTCGAGTCG

>V300080312L3C003R0481218493

TACAGATGATGTTTTTGTCACGGTACTACGGGATCGACAGCCA

>V300080312L3C005R0540585462

TGGCTACATTTTCCTGTTGTCAAGTCTGAACATTGGCTTGCGG

>V300080312L3C004R0230415203

GGCTACATTTTCCTGTTGTCAAGTCTGAACATTGGCTTGCGGT

>V300080312L3C001R0160354676

GAGAGCTTGTTCTTGAGCCAAGACCCGGTATCCCTAATTTGGA

>V300080312L3C004R0680490871

CAGCAATTGACGATGCAGATATGGGCTGCCTTTTGTGTAAGGT

>V300080312L3C002R0251050884

ACGCCAGCCGCTTAACTCGTCGTCATCTCCGCGCTCTTCTCCA

>V300080312L3C001R0250876936

TCTTTGAGTTTTTTGATCTGGATTACAGAAGGCATTCTCTTTG

>V300080312L3C006R0480909516

GGTTGATAGGAATAATCTTGCCGAACGTTTGCATCTTTTGCTT

>V300080312L3C001R0350070660

AAATATGTGCCGTTTTCGTCTGAGGATATCGTAAAACGCCCGA

>V300080312L3C002R0191195560

TGCTAAAGCCTTGAAGCGGCAATGAATCGATTGTTATGGAAGC

>V300080312L3C004R0430911052

CAGAAACGTATCCAGAAAGATCGAATGCAGTGTTGAGCGGAAC

>V300080312L3C004R0631060686

GTAGTTGCCTGGCTGTTTGCTATACTCTACTGTAATTGCATCG

>V300080312L3C002R0040667517

GGTTGCTAACACTGTACCGAGTCCGGATCCTTTTGAATGAATT

>V300080312L3C001R0671135598

GCATGCATGCAATTACTGAGTTCATAGTTGTTGTTTTTCTTTT

>V300080312L3C002R0610767660

TACGAGCAATGCACATGAATCTGACGTTACCTTTTCCATAACT

>V300080312L3C004R0430818513

CCTATACTTGCTATGCTCGGTGACGTAAAAATATTTCTAAGGA

>V300080312L3C003R0370658634

CAATGTTACTAAACAGCTCTTCCTCAATTCGGCAACGATCGTC

>V300080312L3C005R0610602500

GGTGAAAGTGTAGCCGATAGCAGTAACATCAACAGAATTGGTG

>V300080312L3C005R0070676034

AGCCTGGTCACAGTGAGAAGGTAACCACTGCGTAGTGATGGTT

>V300080312L3C004R0070451907

AGTGATGGTTGCAAACTCTCCACTCTCGTTCGTACCCATCTTG

>V300080312L3C001R0141074813

GTGGCCATCGGGAGCGGCAGAGACACCGGTAGCAGTGGTCTGA

>V300080312L3C004R0450421018

GGAAGAATACAAAGACGACGAGACAAACAACGACAACGACAAT

>V300080312L3C003R0230219453

GCAAACCGACAATATAAATTGCAATCCGACAGTGCAGCTCCCC

>V300080312L3C005R0410706484

GGCGTACTTGATCGTCGCTGATGATGCGCGACGCGAAAGACTA

>V300080312L3C003R0140679227

CGATTGTTGCTTGCTTAGATCTCGTTGCGAAAACTACGCGACG

>V300080312L3C002R0210611137

AGCGGCAGTCGTCAAAATATTGGAAAAAAAAGACGATGAGATG

>V300080312L3C001R0630339737

GGGCGTGTGTCTTTTGTTGGCAAGGACGCCAAGTGAGAGAGAC

>V300080312L3C001R0490915330

AAGCTACAGCGAAGATGTCTATAAGATAGCGTTTCAACAGATA

>V300080312L3C004R0491085721

GTAACAATGTTGCATGTAAAGAAAAAGTTGAAAGGAAAAATTC

>V300080312L3C005R0310468513

AAACATGATGGAAACAAATAAAAAAGATGTGCGTATATTATAT

>V300080312L3C005R0680076446

TATTATATTGCATGTATCGTGCCATCTAGCACTCAAGGCAACC

>V300080312L3C005R0101364258

GATCCATGTTTTTGCTGGAGCATTTACCTTGGAATACCACATA

>V300080312L3C002R0390805396

GACTGTTTTTTTGCTAAGCGTTAACTAGATGCTCATTGCGGGG

>V300080312L3C005R0160020228

AACGTACAAGTAGCCTTCTACTGTCTTACCTCTCCTATATTTA

>V300080312L3C006R0220075231

CCGTAGTGGTCAACATCCCAGATTGTTATGCTCAATTTGAGCC

>V300080312L3C002R0510114562

TGGGGACGCTTCTTATAAAGATGTATCCGGCGATAACCATTTT

>V300080312L3C004R0420300764

ACATTATTGTGCATGAACGAATTCCGCAATTCATTCCATTCGA

>V300080312L3C003R0210102738

AGACTCGAGCGGCCGAGTGTCTGTGACGAGCGCAGGCGGCGGA

>V300080312L3C002R0420138493

AGAACCAACAATGTCAGGTTTGAGCAGTTGCAGGTGGCGCACT

>V300080312L3C005R0300072352

GAACAGGACGGTACTCTTGGAATTATTGATATTCTTGAGCGTC

>V300080312L3C005R0470309162

GTTATTATCTCTTTCAGGTGCACAATGGGACTATCCACCATGA

>V300080312L3C004R0150135076

GATAGTGTTACATATTGGGTTAAAGTTTGCATAGTTTTAGACG

>V300080312L3C006R0690060534

GATTACTCGATTAGGGTGTCACAAAGTTATCTATAGCATGTTT

>V300080312L3C004R0301260328

TCGCCCTTTTCAATCAACTCCAAGCGGTGCTTCTTTCCTTCGG

>V300080312L3C006R0470023643

TCAAGGCACACGATCCACGTGAGAGAAAAATTTATAACAAACA

>V300080312L3C001R0510880170

TAAATGTTGCTATGAACAGGGTAATTTAGTGGTTGCCAGGGTG

>V300080312L3C004R0640628824

ATCTTGCTGATAATCAAAGATGTACTTGACGGTAGCAGCTGCG

>V300080312L3C003R0350858795

GAGGGAATATTGAAAGAGAAAAGAAAAAAGAAGGGCACGAGGC

>V300080312L3C006R0360771985

ATGTTGCCGTAGTCCCGCGAAAATATGTGTGGGGAACCTGCTT

>V300080312L3C001R0300192757

GGCTAATGTTATTTCCCCACATTCCATTTCTCGATCAATGGAT

>V300080312L3C001R0150553248

CGGTCTTTGATGCTTGCTGCTGCTGCTGAAGCTGTTGTTCTCC

>V300080312L3C004R0380879908

CTCCTGGAGATGCTTGACACGGTCCTCAAGCAGCTTTGCAGCT

>V300080312L3C002R0251067794

TCGGTTCCTTTGGTGGTGGTGCGTCAGACATGATTAAGCGGGC

>V300080312L3C005R0060970400

CAAACGGGATAAAGTCGCTCCACATTTCACTGTACTTTTCCAG

>V300080312L3C003R0640783021

AAAGAAAAAAAAATGAAACGTTGGCCTTGCTTTTGCTGGATGG

>V300080312L3C002R0400815342

GCCGGGCACGGTCAAGTTTGACCATGTTTCTGGACAAGGATAT

>V300080312L3C004R0331158315

CGATGACTGATAGCAGTTCCTTGGTGCACAGCGGTGGAAGAGA

>V300080312L3C001R0170215499

GTTGCAAGTTCGTCAAGTGTGCTTTGGATCTGACTGGCGACAC

>V300080312L3C004R0051140962

ACTCTGTGTGAATTCATCCCGCCAAAGCTGGAAGGAACATGTC

>V300080312L3C002R0210579395

TATCCGCCTGGAAAATTCGCATACAGCAACGTTGTTGGAGCAG

>V300080312L3C004R0451074638

AACAATGAGCTAAAGCGCGTTGTTGTCAAAATGCTGACTTTTT

>V300080312L3C006R0021016743

GGGCCACATATAAAAAAGGGCGGAGCCAGTATGCACTTTATTC

>V300080312L3C006R0620067303

GTAAAGAATTTGTCAAGAACGATGAGGAAGAAGCAGAAATTGT

>V300080312L3C003R0551322151

CGCAGAAGATTGCAAATATCGTACCACGAAAGTGATCCAGAAG

>V300080312L3C005R0481286888

CGCAGTAACCGGCAGAAGAGGAAATACCCGAGGAGGACAGAAA

>V300080312L3C002R0020082400

GTGTATGCCTGCTGGCGTCCGACTACGAGACAAAATGCGATGA

>V300080312L3C003R0280571269

TTACTTCGATTCTGGCAAATAAGACCGCGCTCGGTAGCAAAGC

>V300080312L3C004R0370864202

TGATTGTCGATGGACGGCAGCGTAGGCAATGAATTGAAAGGAG

>V300080312L3C001R0370429570

GCGATCTGAGATTCAGGATCAGTGGAAGAACCAGTCAAGAATC

>V300080312L3C005R0160970478

CAAAAATGTCAAGTTGCATTCGACTTCGTGTCTAAACAATCGG

>V300080312L3C003R0600688282

ATTCGTTTAATATACAGTCGCCTGCTATCAGCATTGCATCGCA

>V300080312L3C001R0661396774

TCTTCATCCCATTCATACCTGCTTTGTCTCGATGAGCGAGGTG

>V300080312L3C006R0500493068

TGATAAGGAATTTCTGCAAGAGCGGATGAAACGCTAGTCGCAC

>V300080312L3C006R0500407299

TGCTCCGGGTTGACGCCATTCTCCAACAGTTCTTCTTTCAACT

>V300080312L3C003R0721007251

CACAATGCCGAGAGATAAGACGCAAGATAGGCGGTAAAAGAAC

>V300080312L3C002R0451354991

AAAGACAATGGCGGCAGAACTAGATAGACGGTGCATCAGGCAG

>V300080312L3C005R0311031778

TTCGGTAAGTTACATCAATAGCGGCGGCAACATCCAGCATGTG

>V300080312L3C001R0510897046

TAGGAAACTAAACGAGGAGCAAAACACGTGTCTAATGCACTAT

>V300080312L3C001R0520811338

CGTCACTCAAGCTTGACAGCAAGCATCGTCTCTGCCACTTCAG

>V300080312L3C001R0461321859

AACCCGAGGCAAAGCGGAGGAGAAGGTCCGCTAATGCCTTGCT

>V300080312L3C006R0390443797

TCCTCCTCCAGCGTTGCTGTTGCATAATTAATATATATACGCA

>V300080312L3C006R0350850793

TGCAAACAAACACGGTGGCATCGGCTGCTCCTCGTAAGCGACC

>V300080312L3C001R0511400652

CGCTATCGCCAATCTATCTTTTGCGCCATCCATTTATCATCCT

>V300080312L3C003R0340199145

TGGGCACCCATCGTCGGCTTCAGCGTGCAAAGCTTTGGCATGT

>V300080312L3C001R0280108708

CTTGATATCAACGGTGTTCGGAGCGGACGGTGCATTGAGTACC

>V300080312L3C002R0310513521

AAAACCAGCTTCCGGCGCTTTACAAGTTCAATAGCTACTGTGA

>V300080312L3C005R0670789479

GCGGCGGCCGCGGTGGAGGACGCGGCGGCCACCAAGGCAGTGG

>V300080312L3C005R0410792617

AACTCTTTTCAATGAAGAAGATAAAATGGAAATACATGCTGAA

>V300080312L3C002R0660645591

GCCATGATAGTATTTGTGTATTTCCACTTAAATCTACAAACCA

>V300080312L3C005R0250404386

GAACTACGGCAAGGTAATATTGGTATTGGAGTTCGCTTTCTTT

>V300080312L3C001R0261344299

CCCTATTATTGGTTAAAGACAAAATTAGGACAAGATCAATCCG

>V300080312L3C005R0460334607

CCCTGATGATATGTTATCAAGAAATAAGCCTTTTCCAAACAAA

>V300080312L3C004R0511103725

AAGGGCCTTTGCCTGGTAGACTATCCCGATTCATTCTTTTCTC

>V300080312L3C003R0090842720

CTGGGACGTACCAATTTATGAATCCAAGCTCTCGTCCATATGT

>V300080312L3C002R0380457815

CATTTCACATACATACACCACCAAAATTGGTTTGATGAGCCAT

>V300080312L3C002R0461096588

TGTTAGAGAGACATTTGAACAGAAATGAAGGAAAGATAGCGCA

>V300080312L3C004R0380742164

CTGTTAGGAGGAGGTATATGCCACAACGGAACAGATGTCGTCG

>V300080312L3C002R0700447957

AACCTCAAATTTTTTTGCGCGGCGCGTTCGGGTCGTAACTTTT

>V300080312L3C001R0350406554

ACATTCAAGACATGGCTCAAGCGGCACCAGCGAGGGGCGAGAC

>V300080312L3C006R0110098486

GTATCCAACCGAGGATGATAATGGCAATCCCTTGGTGACCGAG

>V300080312L3C002R0040297681

ATATCTATGAGAAATTCAACCGTCTTTTGCATGCAAACTATGG

>V300080312L3C005R0461134145

GTATGGTGTTTTAGAAATGCAAGACACAGTTTATGAGAACGAT

>V300080312L3C002R0621001240

GCCGTCAATGGAAGAAATAGCGGACGAGGCCATGTGGATCGCG

>V300080312L3C004R0450256784

GCCGAAAACATTTGTTGCGCGAGTTCATGTCAAAGTGCTTCGT

>V300080312L3C006R0360547315

TATCTTGCATCTGCTGCGCAATACATTTGTGGTCTGGGTGCAA

>V300080312L3C002R0010745875

TATTTTCATGAACTGCGCCAGCTTTATCCGGATACGAATTGAC

>V300080312L3C004R0091096332

CGACTTGTCGTGCCGGCCCTCCGATACAAAGCCTGACAGCGAT

>V300080312L3C004R0181257030

CGTCGTGACACAAACTCGATCGCAATTGACGTCACCCGCGTGG

>V300080312L3C003R0270692965

TCCTGAGTGTCAAAATTATACCTCCAACTCGTCGCAGACCAGC

>V300080312L3C006R0551280663

CACGGCCGCTCTGTCGAGAAGGAGCTCACTGAATTGGGTAACG

>V300080312L3C004R0140512467

ATTGTCTTTTCATCTAGAAGACGGATAACTTGCTGATCAAATC

>V300080312L3C003R0020108462

ATCATTATCATCCCACACCTGTGTGTACCTTTTTCGTTACCTG

>V300080312L3C005R0651212827

CCACGGAAATAGGGCCAACGTCTTGAGCGTAGCAGCTAGCACG

>V300080312L3C001R0120134141

CTGCCGCAACGGTATCAAGAATTTTACCTTTGACATTGGACGA

>V300080312L3C004R0700614135

GCCATGACATCAAACTCAAGCACTGCTCTTTCCCAGATCATTT

>V300080312L3C004R0050027993

AACTTTAACAAAATCGACACCAGCAATGGTTCGCCCTGGTACG

>V300080312L3C002R0280311545

CCTTGCAGCCCAATGCAACGGGGTTGCTTGCAAATCGCCTGCC

>V300080312L3C002R0280981419

CATTTTTGGAAAGCTGCTGCCAAAACTCTCAATCTTGGCATTT

>V300080312L3C005R0420444631

CCGTTTGCATAGCTACACTTCAATTATATACGTGTTTTCCACT

>V300080312L3C006R0190794869

CAGATATGTCTTTTGTTCCAAACCTGAAACAATGAACAAAAAT

>V300080312L3C003R0670261121

CGTTGAAGGATGATGCGCTGCAATAGCTGGATCGCCAAGAGCG

>V300080312L3C004R0201382301

GAAATTTAGAAAGCGGGACTGAGTATATAAAACTTACACACTA

>V300080312L3C003R0630237875

TCAGCGTCGCATCGAGATTTGCGTTTTCTGCAAAAGTCGCAGC

>V300080312L3C003R0520951170

TAAAGTTAAGGTTGTTGGTGTCCTTTCTAGTTTAAGCCATTCA

>V300080312L3C003R0270421471

GTTCTCTGTGCTTGGTGCAGACTCTCGAGATCCTTATGGTGCG

>V300080312L3C004R0550532994

TTGATGGTTCTTTTTGAGCGAATCCTGTGACATTCAGGTTATA

>V300080312L3C005R0310245835

TAAAAATGTATGTATTACAAAGCTAACTTGCCTCGATCTCCAC

>V300080312L3C001R0170377530

CTCCGTTAGATCGGCTTCCTTGGCCGTCAAGAGCTGTTCCTTT

>V300080312L3C006R0710018739

GCTGTTACACTAACGTTTAAATCCACCATTACTGTGTGACATC

>V300080312L3C002R0210026651

TCCTTCAGCTAGCTTCTTTTCCTTACCTCGTGTTTTTCTATCG

>V300080312L3C004R0650519107

AGTTCGTGTGGGAAATATATGGGCTTGATTGCTGGATGATCCT

>V300080312L3C001R0470080190

GATTTTCCGCGCAATTGAGAAAAAGAAAAAAGCATACTTTGTG

>V300080312L3C002R0680841739

TAAAATATACTGCCGACGAGTTTGTCCAACAGCGTTCTCTCTC

>V300080312L3C004R0621326637

ATAAGGACAGCCAGACGCAAAAGCAGGGCATTGTCGCTTGAGT

>V300080312L3C002R0420109164

AAGGCCGGGATGGGGGATTGGAGCAGCTACGGTGGTTGACATG

>V300080312L3C001R0710561912

GGAACAAAGAGAAAGGGAAACCAACCGAAGCTCCAAGTTTCCA

>V300080312L3C003R0351007585

AACATGTTCCCGCTTATTGCTAACTATTGTCTTCGCAATTCTC

>V300080312L3C005R0191020824

TGCCTGTGTATGCCAGCAGGTTGTGGTTCTCTACACCTTGGGC

>V300080312L3C005R0310484937

ATTGCTTGCGTATGAACGATAATGGATCAAAGCCTAAAAAACA

>V300080312L3C002R0060475525

ATACTTGCGTATAAAGAGTCCCCCAAGTCCTGCGATGGGATGC

>V300080312L3C001R0350824865

TATGCAGAAAAGGATATCGGTTCCATGTTCCTTTTACTGCTCC

>V300080312L3C004R0130128668

GCAAGAAAAGCGAGGAAAGGGATGTCCACCTTTTTAGAATAAA

>V300080312L3C006R0570615852

CTCGCAGATAGAGAGGAGAATGCTTGTATATATAATTCTTACC

>V300080312L3C006R0401156535

AAAATCTCTCGGTATCTGCTCGCAGATTTTCATTTGACATTGT

>V300080312L3C006R0490234644

TTTCAAGTAGTTGTAATAAATTATCTAGTGCTTCTACTAATTT

>V300080312L3C001R0701224131

AAATAGTGGCATTCAATTATCCGGATTAGGGCCATTGGTTCCT

>V300080312L3C001R0250042358

TATTCCGAGATTGCAGATTATAGACGAAAACCAAAACTTTACG

>V300080312L3C004R0660141669

AGGGCATCTGGATGTCCCGTGGACGGGGCATGCATGTGTTGGT

>V300080312L3C004R0211340620

ATATGGGAGCATCAAGTTGGTTTGTACCAGGGCGCAAACATGG

>V300080312L3C003R0150168182

CTTCGATTTCATGTACACCGGCCTTCCGCACAAAATTTTGCTG

>V300080312L3C001R0290315489

GACAAAAGGTTTTAGAAGCCAAAGCCAAGAGTAAGGATACGTT

>V300080312L3C005R0611062950

TGGCTTCTGGTTGTCATGATTGCTCTTGGCTGGAACGAGTTCA

>V300080312L3C001R0510695475

GGTTGGTGCTATTGAGTTGTTGAAGAGTTTTCCAGCTCAGCTC

>V300080312L3C002R0151371272

TCTTCGACTAAATGTCTCGCTAGCTTGCCCTTGAAGGAAGAAG

>V300080312L3C002R0251051952

CCAAAACGACAAATTGCAGCGACATTAATTTTTGGAAAAAGTG

>V300080312L3C001R0181065538

CCAGCCAACGTTATCTTTTTAAGTGCACGTTTCGCCCATTATT

>V300080312L3C001R0540508333

CATTGGACTCGTCGTTGCAACGGCCGAGCACCAGTTGAATATA

>V300080312L3C003R0130797599

TCCTTGTTGTCGACTCTCAGGAGCGTATCAACAAGATTTTGGA

>V300080312L3C006R0550664595

GATTTTCAACCGCATCTTGTCCAACAAGAAATACCTCTGTCGC

>V300080312L3C006R0620945153

CGTGGCAGACTGGACACGTGCATCCTGCAGGAAACGTCTGATG

>V300080312L3C003R0410479904

TGGCAGACTGGACACGTGCATCCTGCAGGAAACGTCTGATGGT

>V300080312L3C001R0440701676

GCTTGCTCAATGGAACGTGACTGAGCAACAGCAACAGCGTACG

>V300080312L3C001R0070057922

GATCTCCTGCACTTGTTCTCTGCGGAGCTGGTGTTCGCGTTGC

>V300080312L3C006R0191088030

CGATACAAAAGCTACGCAGTACAAGAATTGAAGACAACGTATC

>V300080312L3C001R0270275417

AACGCCCTTGACTTGGTTAACGGCACCTTCAGCAGTGTTCGTA

>V300080312L3C002R0640324530

TTCGCCGACCAAACGCTTGCGGAGACCAGCAGCACCTTCGACT

>V300080312L3C003R0180163830

CGACCAAACGCTTGCGGAGACCAGTGCTTCCGACGAGGGTACC

>V300080312L3C005R0040230430

GCTTCATAGATTCTCACGTAGTGGCCACTTAACGAGCCTGATT

>V300080312L3C002R0450283411

TGTTAAAGCTTAGTGCCTAATTTCCGAGGAAGTCTGGCTCGTT

>V300080312L3C001R0300746622

GCATGTTCAGCGTTTGCATCATTTGCTGCTCTGCGGAACATTT

>V300080312L3C006R0501337466

GATTGGGTATCGGCAAATTTAACAAATATTCGTACAGCTTGGT

>V300080312L3C002R0651187826

AATATTCGTACAGCTTGGTCATCCGGTACTTGGCCGTTCGGTA

>V300080312L3C006R0630922124

CGAGGTCCATCAGCCAATGAATAAGGTGAACGCGATCTGGACC

>V300080312L3C002R0480533846

ACAGCATTGGTGGTAGCACCGGAAGGCGCTGGTAACGACGCAT

>V300080312L3C001R0071388681

GAGTTCTTCGACTCTTCAGTCTTTCCAGTCTTTGGATTAGGCA

>V300080312L3C004R0301175866

AGAAGTGAAGAAAGCCACTGCGGTTGCTGCCAAAAAATTGGTA

>V300080312L3C004R0670647978

TATTTAGATTCCAGCATAGTCCCGTTTTACATGAATACTGACC

>V300080312L3C006R0490546122

ACTCTTGAAAAATGGCAGACTTTGCAGCCATCGCGCTCTCGAT

>V300080312L3C005R0161346467

GGCTCGGCAAAAGGTTCGATACTACGTTGCTATTTCAGTCTAT

>V300080312L3C003R0400504079

TATTAGCTCTCTTAGCATCGCGACGAGACGAAAATGACAATAA

>V300080312L3C001R0260880265

AGATATCTCTCAGCAACGAACCCAGCATCGAACTATTCAAGAA

>V300080312L3C005R0251398279

TTTCTATTCATGCATTTGTAATCTTAATGTTATATCCAGACGG

>V300080312L3C006R0340895421

GGAGGCGTGAATACTCTTGGAGGCGTTGTAATATTGGTGTCTT

>V300080312L3C002R0140029043

TCCTTTTCTGCATCCATCTCCTCAATTACTTGCTCCAGCTGCA

>V300080312L3C001R0620500975

TGGAGCGTCAGTGCTTCTATCTGGGACTCAAGTTGCCGCGTGC

>V300080312L3C001R0640486920

CTTACTGTGCTTTGCGTTGTCTGGTTGAGAGGCTGCTGTGATG

>V300080312L3C001R0390904184

TGATACGTTTACGGGACGCGTATGCCAACCGTAGCTACTTCAG

>V300080312L3C002R0170941337

ACACCCGACACAGAGAAGAATATTTGCGATTCTATAGCTACAT

>V300080312L3C006R0260274104

TGTCTGGGGCCAGGATACAAAACGGCATGCCACACTCATATCT

>V300080312L3C004R0720543854

GCTCATCAATAGCCGTCTTTGACATTAGCAAACCAGCTGACCC

>V300080312L3C004R0560170485

GTTGGCGATCTTCGCAAGTTCGACGACATTTACAATGCTCTGG

>V300080312L3C005R0341079743

AACGGCACACCCGTTCCATTCTGGGATTTTGCTGCCAAAGTTT

>V300080312L3C002R0370859833

TTGATTTTACAGTATTACAACTCCCATAAGCAGAAATAATAGA

>V300080312L3C003R0431379970

CCACTTTGCGTGCTCCTTGGCAACGCCCTAGATAAATATCACA

>V300080312L3C006R0550273100

TAGTATCTGTCTCCTCCGACAGCATCAAAGATAATGTCCACCG

>V300080312L3C004R0130622348

CACTCCTACAGCCTTGACTTGAACCAGGACATCTTTTGGGCTT

>V300080312L3C003R0280840898

TTTATGTGATATATCAGATATTCTACTTCCTCTGCAATGTATG

>V300080312L3C005R0670975657

GCGCTCTTCTTGATCGCCGTTTTCACTGTTTCTCGCTACGGCA

>V300080312L3C002R0700419759

ATCCCATTTCTACAGAGTTACGTAAATACGGGGGATTTTACCA

>V300080312L3C001R0271326721

CTTGTAGCGGTTCCATGGCGCTTGTGGTTTACAGGCGATGTCA

>V300080312L3C003R0591071174

TATGGGTCGCGCAGATCTTGTGCCTTTCCTTGGCACAACATTC

>V300080312L3C001R0380708474

TATGAGAATGTAGAAGGTGACCCAGAAACCGGCACGGAAGGCT

>V300080312L3C001R0711342088

GACCCAGAAACCGGCACGGAAGGCTCAATTACATCAGAGGTCG

>V300080312L3C004R0541150547

TACCTCAAAGTATGGTCCACCATCGTCACCGATCAGTGTCCAC

>V300080312L3C001R0330673666

TGCATACCCGTCTCAAAATCGACCTAGCGCATTGTCTTCAAAT

>V300080312L3C003R0171231955

CCCGTCTCAAAATCGACCTAGCGCATTGTCTTCAAATTTGAGA

>V300080312L3C003R0221149718

CGTTTGTTTCTGCTTTGCTGGCCGGTGCGAAGCTTGGCTTCTT

>V300080312L3C005R0320193962

GACGTGGATGAGTGCCCAGACAGAAGCTGAAGAAGCGCTTTCA

>V300080312L3C002R0151353088

AGTGCAGTTGCTCCAAACAATGCCAACCGGCTCAATCAGTAAA

>V300080312L3C006R0640496305

CGACAATTCCGCTTTCATGTCCTTTGATTTTAACTCCATCTTT

>V300080312L3C001R0401008188

AGTCGGAAATCTTGATTCAATTTGCTCGATGCCATTCGCTTTC

>V300080312L3C004R0280344334

GTAACCATCAGAGGCTGTAAAAACATGCTTCCGGCCTGGGTCG

>V300080312L3C002R0310947229

AATATTCTTCGATTTCTGACATGCTGAAGTCCTCCAGCTTCAA

>V300080312L3C003R0250222430

TAAGAAGCGGTATTTCGTCAAGTCGAAAATTTCTTCAAATGGA

>V300080312L3C006R0600741872

GGTAATGAAGCGCCAGTGGAAATTGGGTCTTGGAGTTAAAGCA

>V300080312L3C006R0450498840

GATATAGTTTCAAGAAGCTCATAGTCTTCATCAAAGTAAATCA

>V300080312L3C003R0280030657

TTTTACTTACCGAATCATCTAAAGAGAAGCAGTCTCAATATTA

>V300080312L3C006R0291041186

GGACCAAAATGATTAAATGTTGTGCGAACGCATCCATATATAA

>V300080312L3C005R0650422124

GGCTAATCTAGTTTGCGTTTTGGAGCTGCCCATACGAGTCACA

>V300080312L3C004R0700668391

ACTACATGCTTTATTCTTCCAGGACACTTAAAACTGTTATTTC

>V300080312L3C002R0260339258

GAGGTGAGAGAAAGAGAACGCCCGCGTATTGTTTAGTTCGTCT

>V300080312L3C003R0550407830

ACGGAAAACCGTTGCAAGGTTTGACTGGAGGCAGTACGTACAT

>V300080312L3C005R0380608143

CGATACTTTACAATTTCAAAAGCTGATTTGGTCGTACCAAATC

>V300080312L3C005R0590953177

TGAAAGAAAATCAGACCTGTCATGCTTAAAGTACCGTTAACAC

>V300080312L3C001R0210018711

AAGCATTTTGGCAAGCGTACTCGAATTTTTTGGCTTCCAGATT

>V300080312L3C005R0080309647

ATGGGTAGGTTAAAAGAAAATACTGTTTTCCAATGGCTACATT

>V300080312L3C001R0520533319

GTTAGCGGGGAATGATTATCCAAGATGAGTTCTACTCATTGTC

>V300080312L3C001R0290595848

CAATTCGATGTCTATTTGCAGAAAGTAGAATTCGCATGGGATA

>V300080312L3C006R0080676843

TATTTGCAGAAAGTAGAATTCGCATGGGATATCTTGAGCGACC

>V300080312L3C005R0380449130

AAAGTTTCCTTCAAGAATCAGACGATTGAACTAGCGAATTGTA

>V300080312L3C004R0201145547

AATGACACTTCCGGAAACGGGCGAGCGTATCTATCGCCTTCCC

>V300080312L3C005R0091230609

GAGAAGACAAAATACTCATCGCGTCGCTACATCCTTTATAGTC

>V300080312L3C002R0390160622

CTACCGAGTATGCTGTTTCTCGCAATTACCTGGAGCTGCTGGC

>V300080312L3C006R0550656403

ACATATTGCTTGTGCTTGGGATTGATCGTTCACAAATTTTGTA

>V300080312L3C005R0690569457

ATACTATCGAAACCATGTATAACGTTTCTATCCGTGGGTAGCT

>V300080312L3C002R0340342140

TTACACTTGCATGTGCTCTAACATCGCTTTTCTCTGGCCAATG

>V300080312L3C006R0600404219

TTTTTCGATCCATTTGTATGTACCAGCGGTGCCAAGAAGTATG

>V300080312L3C005R0400711832

TGTAAATACGCTCAGCAAAGTCATCTTCGTCGTCCGAGTCACT

>V300080312L3C006R0531028151

TATTATCGATAATTCATATGCCTCTTTGAGTGCACGGTCTTCA

>V300080312L3C002R0521033618

CCAGGGACCGAGATGATACAGAATATTATTCGTCACATCAAAG

>V300080312L3C001R0400558028

ATTGAACTTACAACACACTGTATTGAACATCTGTCTTGCCGTG

>V300080312L3C002R0150571105

AGTAAGTTTAGCCTGAACAAAGAAAAGGGGAACACCAAATCAG

>V300080312L3C003R0651390934

GGACAGCAATAACATTCTCATCTATGGCTTTTTGGTACAGCTC

>V300080312L3C005R0380307489

AGAGACTGAACGGCTGTGGATCGCCGGTTGCCAAAGGTGGAAG

>V300080312L3C003R0400347868

CGCCCAGACTCATGACTGCCTTCCACAATTTCTCGATTGGCAG

>V300080312L3C003R0440253363

AATCTGTGGTAGAAAATTATCATTATTAAATGGAAACTCATCA

>V300080312L3C002R0601317159

CGTTCCACACTTATCTCCTCGAGACCTTGAGCTTAATCGTTTA

>V300080312L3C004R0490412994

GAATTGTGGGAAATATGCGCCAGGGAGCCGCTTTATGTATTCA

>V300080312L3C006R0690825959

GCATTGCTGGTGATGTTTTGTCGATAGCAATGTAGTTTGAGAT

>V300080312L3C001R0210605480

GCGCATGAGAAGCGCCTACTACGCTTTGTTGCAATGATATATA

>V300080312L3C001R0710178631

CGAATCGAAAAGAACCGAACTTGGATGGATGTATACGATATCC

>V300080312L3C004R0430611729

GCCTTCGGACGGATCTAATCTTATTGCAATGGAATCACAGTAG

>V300080312L3C004R0390776973

TGCAATGGAATCACAGTAGCCAGTCAGCAATGCCCGCCTCAAA

>V300080312L3C006R0691265550

ATGAATGCATTTACCATCGTCTCCAGCGACTTCTGACTTCTTG

>V300080312L3C002R0120997018

AGGATGAATTTCATGCAGAGAGACAAAATCTGCAAAAGCCACT

>V300080312L3C003R0410046669

TCGCAGTGATATTCAGAGATTACACTAGAAATCTGCCTTTCGG

>V300080312L3C004R0590891740

GGTTCTGCACTCATTAATGACTTGACAGTGATCATCAAAAGTG

>V300080312L3C002R0480009291

TGTGGTGAGTTTAGACGTAGTATGTAGCGCACACATCACCAGG

>V300080312L3C003R0391272175

CGTTGGCCAGAAACAATATGTTCTTGAAACGATTAGTTCTCAT

>V300080312L3C006R0541154456

TTAAGGAACGATAATATTGGGTTCGCGATTGGGAACAAACAGA

>V300080312L3C006R0700591627

AACGATTAGCATGTGAGACAAAAACTGAATGAAAGGCTTCAGC

>V300080312L3C002R0071305727

CCTTCGGTGGATTCGGAACGACCAACACGAGTCAGCCGCAACA

>V300080312L3C002R0141007209

GGTAGCAACCAGACTTCTTCTTCGTCCCTTGGGTTTGGAGGGT

>V300080312L3C001R0221235427

ACCTCCCGTGGCTTCTATTTGGGACATTGGCGTGAACCCACGG

>V300080312L3C003R0070611043

GGCTGCCCCGTGCGCAAAGTGGAATTGTTCATATCTCTTCGCA

>V300080312L3C004R0140201785

ACGGATCATTACTCTGTCAACGGCCGGCCGGCGACTGCATCTC

>V300080312L3C005R0670432831

ATGGCGGCATCAGTGGGTTCTTGGCAGGTTACTTTACCACCCC

>V300080312L3C006R0161153070

GCGAAAGGAATGCTGGAACGAGCTATGTACGGAGCACCGAAAT

>V300080312L3C002R0400284874

CTACCGGCTAGAGTCGTTACTGTCAAATATATTAGGAGATGCA

>V300080312L3C004R0301258298

CCTTCTCTTTCTGCATCCTATATCTCGCAAAATTATGATGCGA

>V300080312L3C006R0081198511

AGACGTCTTTACCGCAACAGATCCATTATTGAAAGACGTTGGC

>V300080312L3C001R0351035998

ATACAATTTGACGACAACAAGATAATCAGTGGTAGTCGTGACC

>V300080312L3C004R0540973422

CCGTCAAACTCGTTCAAAGCCGGATAAATCATGCTGTACAAGA

>V300080312L3C002R0440278457

TGATGTGTGCGCCCTGATAGAATGGACAGCAGAATTAGAAAAT

>V300080312L3C004R0540943727

ATTCACTAGAACTAAAGTAAAAAACTGACGTTTCTGGCAGCTT

>V300080312L3C004R0681222050

AAAGCAGCACTTAAAATAGGAATCGATAAAAGTAAAATGATTT

>V300080312L3C005R0180182396

TGATTTATCATAATGTATGTAACCAGTTGTTTTAAACTGGTGG

>V300080312L3C003R0280139686

CAATGGATTATTGAATAGCATCATCGATTGCGAACATCATAGG

>V300080312L3C004R0550636434

CTGAACAATTGTAATGATAGCAGTATACACATCTGACCCGCTT

>V300080312L3C003R0520559219

AAGGCAAGCAATAATCTGTATATGGTTCGTCCTTGTCAGAATA

>V300080312L3C005R0050726699

ACATAGACACGCACACTGAAACATTGATTCTCCCAAAAAATGG

>V300080312L3C003R0200662380

CAAAAACGAAAAAATACAAGCAATACAAACATTGTGGAAGATA

>V300080312L3C001R0131290947

AAAGGTTTAAATTTGTCTTAATCGTTGTGCAATTTGATGCTCC

>V300080312L3C003R0061227451

CGCTGGTCTATAGGAACATCCTTGCCATCTATCCTTCTTTCCA

>V300080312L3C006R0440833212

TAGTGCTATTGCAAATATATGCTTACAGAAAGCAATGCTTAGA

>V300080312L3C006R0081336095

AATCCAAAATGTAAATTTATCACTGCATACCAGGAGCGAAGGT

>V300080312L3C002R0650546785

TACATACATGGCTTACCGATATTTACACCGGTATATGCTAGGT

>V300080312L3C003R0100595782

CCATCGTCGAAGATTATGCAATTCACATCGCCTTGGTAATCCT

>V300080312L3C001R0060411017

GACCATATTTCACAGAGAACGTCCGGATTCGTCGCCAGAGACT

>V300080312L3C004R0370005937

CACGTATAACTAGGCCAGAAATCTTTTCGAAATATCTAGGTGA

>V300080312L3C003R0410836208

CGCAGAAAATATAAGTCTCAGTAACAAGCTTGCAGAATTCTTC

>V300080312L3C006R0630664564

CGGCATCAGTTGCGGCAACGTCGCCTGTGCCACCGAAACCGCA

>V300080312L3C005R0550957342

TCTCTATCGTCGACTCAAATCCAACCGCATCCTTCCAGCAGCA

>V300080312L3C005R0341022376

CATATTTCTCTCTGATGTCCGTTATCAGATACGTACACGCATG

>V300080312L3C004R0280154033

TATCGTCCTTTCCCAATCTTGTATTTATATCACCTGTTATCAA

>V300080312L3C004R0070175074

CAATCTTGTATTTATATCACCTGTTATCAAGTGCAGAAAATCG

>V300080312L3C006R0271345784

GCTTCCTGTCTGAGGTTGTTTGTCACACTCTCTTGGTCCTATC

>V300080312L3C005R0251183865

TGTTTGTCACACTCTCTTGGTCCTATCAGATGGACAGCGTCAG

>V300080312L3C004R0711010457

AGAAAAAGTTCAGGTACAACGTACTACATGCGGTCTCCCCTGC

>V300080312L3C006R0460942000

AAAAATAAAGAGTTGTGGGAAGGGAACAGAGAAAGACGCGAGA

>V300080312L3C005R0071063755

GAAGCAGACAAGACGCCCTTTGAGAACTGTTCGGCCTGCGACA

>V300080312L3C002R0670279860

GCAGCCTCGGTGCCACCTTCGTAACGGTAGTAGCCTTCGCGAA

>V300080312L3C006R0401151881

CATGTACACACAACGTATATTCTATCATTCTATGTACTTTTTA

>V300080312L3C006R0361233703

TGTTGACAATGAATGATTCCTTGTGAACATACCGGAATGATTC

>V300080312L3C004R0640040291

TAAAGAAACGTGCATTGCTCGGGTTGTAATGGTTTTTGTGAAA

>V300080312L3C006R0680931932

TAGGTGTATAGCTGCAGGAATGAAAAAGGGGTTGGGTTAGAGG

>V300080312L3C002R0700934261

AGTTGCTGTTTTCAGCTGCCGATTTTAACCGTTTCTTTTCTTC

>V300080312L3C002R0431106961

TCATTGTTGGAGAAAACTGATGAAAGCCCAAAGTCCGTCCCCA

>V300080312L3C002R0600099623

CTCGCATGAAATTTGCTGCTCTCGAGAGCTTGTCTCGTCTATA

>V300080312L3C001R0030986158

AAATGTGCAAATGGTAGTCCTGACAATCGCTTTAGATCACTCG

>V300080312L3C004R0361264386

GTGTCTCGACTTGGCGATAGCAGCTGGTAAGCAAGCAAAAATG

>V300080312L3C003R0210989224

CAAAATGGAGGTCTTTGCGACGACTCAAAACGATGCTGGATAT

>V300080312L3C002R0091253369

GACCTCCACGATGGTGATCTTTTGGTCGCGGCATAGCTGAATG

>V300080312L3C002R0671029098

GCAATCGACATCTCGACTGTATCACCACCACGATGCCATTGAC

>V300080312L3C003R0710825673

CACGATGCCATTGACGAGTTTTTCACTAACAGCAACATTGAAG

>V300080312L3C004R0140539990

GCAGAAGTTCTCCAGGTGCTTGATCAAGAAACTAGCACGCTAT

>V300080312L3C005R0040727039

GCGGTCGGTGTGCCACAGCAAACATTGCATACAGTTGATCGAT

>V300080312L3C005R0320760367

AATGAAAACAAACGCTAGTCTTTGTAAACAATCAACAAATTTT

>V300080312L3C006R0420975077

TCTCTTGGGTCTACCCAGGTACCGTATATGTCTACAAATAGCA

>V300080312L3C002R0030789277

TATCATAAATTTCATGATTCAGCATAAATAATAGCCTTCTACA

>V300080312L3C003R0570026792

CGGAAGAAATGAAGAGAAATTCACTGCAGCGTGCTGCATCATT

>V300080312L3C006R0140113599

ACTTTTTCCGTTTCTGCCATCCTTATACTGAGCAGCGCAGTTC

>V300080312L3C006R0190819698

TGCAAAAGAGAAAAGCCAAAGATGCCGTTGCTAAAGGATACGA

>V300080312L3C004R0031299135

GCCGTTGCTAAAAGATACGATCGACTCGCGACTTTTCCGCATC

>V300080312L3C002R0720863877

TTATCGTCAATGATAATATTTACATACTGCCAGGCATTCCTCG

>V300080312L3C006R0431370499

TGTCAGCGTGACAGGAAAAAACTACGCTGCAGTGAAAGCGGTT

>V300080312L3C006R0470457382

CCATTTTTTTTTTTTGCCAATAAATTGGTGGTGCAAATAAAAC

>V300080312L3C002R0420789485

TCCTCCAAGCCCTTGGATGAAAGTGTCCGTGGCAAGATCTCCA

>V300080312L3C006R0090644606

GCAAAGAGGTAAATATAAATATTCATGATTGAAAGGGGAAATG

>V300080312L3C004R0610359798

ATCGTGCGCAAGCTCTACAACGACCAGCCCTCTGTTGATGAAC

>V300080312L3C001R0400242340

CGCGATCGCTTCTTTACAGCAATGCGCTTCTGTGACGAAAACA

>V300080312L3C001R0220195820

CGGATTTTGATGGGTATTTTTTGCCCTGGCCCCAATTCGACGT

>V300080312L3C001R0720912891

CCTTGATTCTGGTTTGTTGACATCTTCTGATCCCAAGAGGTTC

>V300080312L3C006R0450856594

TCACTAAATGTACTCATCAATACGAGGAACTCGACTGTAAGAG

>V300080312L3C003R0580204419

GTAAGAGTTCAAATTTTCAGGACCAGCTTAAGCTGAATGCCCC

>V300080312L3C002R0470193074

AGTGCACCTTTTCGTGTCAAAGCAATCGTACCATGCCGAAGAG

>V300080312L3C003R0310862115

GACTGATGTGTGGCAGGAATGGGATGTTGGACTCACGCAAGGT

>V300080312L3C002R0290743284

CCATTTCACTGTATCCTTTGGTAGCCCTCCTCTCAATACCTCT

>V300080312L3C001R0671083425

AAGGACGCGTAATTCGATTAATGGACGCTTCGTTCACTTATAG

>V300080312L3C004R0020643949

GATATCTTGCACCCTGTAATATTGCTAGTCATCTCGTCCGAGG

>V300080312L3C004R0240465312

ATAATAAGCCCGTAGCGAAAGTTGCTCCACAGCGGAGCCTAGT

>V300080312L3C004R0391007753

AAGCACGCATTTAGGAAGACCAGAAAGAAATGCATAGAGACCG

>V300080312L3C002R0560074355

GACAATGCGTCGTATCAGGAACAGCATTCATATTGCGAGCAGC

>V300080312L3C004R0100934034

GTGGCCAAGGAGACGCACCGCTTCCATCCACTCACACCTTCTT

>V300080312L3C002R0440697760

ACAAAGCCAACCTTAGCCGATGTGGACGAGGCAGCAGGAGATA

>V300080312L3C006R0230635994

CCAACCTTGGCCGATGTGGACGAGGCAGCAGGAGATACCGGTC

>V300080312L3C005R0430917287

GGATGCTGTCTGACTGCCACTGCTGACCGTTTTAGGCGACTGA

>V300080312L3C006R0610930830

ATCAACGATTATCAACTCGGATCCACACGTTTACCTCTGCCGG

>V300080312L3C001R0600481597

GCAAGCATACGGGAATAGAACTTTTGGAAGATATCCTTGTCAT

>V300080312L3C001R0110470352

GGAGTTGATCCACATATGCACGTGGAATTTTTGGAGGGCTGTT

>V300080312L3C002R0351113190

TGCATTACCATCAAAACGCTCTTCTTGCAGCATGTTGGGAAAC

>V300080312L3C003R0630070100

ACGCAGATACTCGATCGCCCGGCGACACTAATCGGTTCAGATA

>V300080312L3C006R0720674143

CCCGGTTCCCGTACTTCTTCAAGCTTGCGAGTCAAGACAGTTT

>V300080312L3C005R0490632398

TTACCGGGTACAAAAGCTTGTATCACTATTCTCTGGACTCGGC

>V300080312L3C004R0611223830

TCTTATTCAGCATTCTTTGTACTGCTGCTTAGTTTCAACACTT

>V300080312L3C003R0051092068

GCTAATGTTCCAATGTCATCTATTTTCCGTGCGCGGCATACCA

>V300080312L3C006R0420947294

TATATTTCCCTCCAGAATACCGTGCATATACAGTTTGCCACTA

>V300080312L3C005R0570771609

TGATAATAACGACGCTGTCGTTGTAGCCGTGGGCGTGGAAGCA

>V300080312L3C001R0480203043

GGGCGTGGAAGCAGCAGCAGCGGTAGCTCTGGCCGTATCAGTT

>V300080312L3C005R0410110486

AATTATCATCCACGTTCAATTATTCGTCGGTCAGCAGATATTG

>V300080312L3C004R0690568580

GAAGTGTAACCACTCTGGGGCAATGCAGAAGAAGCAAAGTTGG

>V300080312L3C006R0621177444

AACATGTAGCCCATCGGACCTGTAAAGTCCATACCCACAGTGA

>V300080312L3C005R0281269699

TGTTGGCCAGGCGTGCCGGGTGCTGTGAAGCCGTTCATGAGCT

>V300080312L3C006R0710473044

CAACCAACCCCTTCCCAATTTTCCACCATTGCCAAATATCTTT

>V300080312L3C003R0291304925

AGTTGTCTGTGCCCGTGTTTGTGTTTTGGGCACTTTGACTACT

>V300080312L3C006R0260193549

AAAGAATCTTGCGAATCGCAAGAGCCGTTGTTCTCGCGTTCGC

>V300080312L3C002R0511375520

CATACAAACGGCATTTCGCTACGGAGGACATTTTCGCCAAACG

>V300080312L3C003R0080608724

TACTTTTGGGAAAAAGTGTACTTCAGAATGCAACTCTGGAATT

>V300080312L3C001R0250187595

ACCGGTTGATCTTAGCATACTCGAGAGTAATCCCCTTGAGATG

>V300080312L3C003R0720064859

ACCAACTATATCCGTACGTAAACTTATTTTTCATAGAAACAGG

>V300080312L3C004R0600961670

CAAGACCTTGACGACCTTGCAAGGCCTTCCCAAAGAATACGAC

>V300080312L3C006R0091193284

AATATATATTCCCCTCTCTCTCTCTTCCCCCAACCTTTACACA

>V300080312L3C002R0400334035

CTCTGGTTTGCTTGAATAGCAGAAAGATGTATCTGTGCTCTCC

>V300080312L3C005R0270822642

TGTCAGTCAGGGGTGGGGATGTAACAATCTATCTATCTGTCCC

>V300080312L3C001R0120151836

CGCAGCTGCGTCGTCTTCTGGCAATAGGAAAGAGACTTTAGGT

>V300080312L3C003R0560419521

GATTCATGTTATTTGCGTTAAATGGGGCATCTATCAAATCTCT

>V300080312L3C005R0340941088

TCTTCTTCGTGTTCTTTTGGTTTTGTTCGTCGCTTTGGCTCCT

>V300080312L3C004R0071265624

GTTTCCCTTTTACATCCTTGGCCGCTGCTGCTTCTTCCTCTGC

>V300080312L3C003R0451044254

GTACTGCAGGTCGCCGTCATATCCTTTGTAAAAGTACAGGGCC

>V300080312L3C006R0071294973

AGGGTAAAGAAACCGAAGCTCAACGCGCTCCTCCGAGAGGTCC

>V300080312L3C002R0100192000

TTGTTATAACACAAAAAAGCGCGCGTTTTTTTTCCTTTGCTTT

>V300080312L3C004R0590178301

AAGCAACATGGTTCAGGCTTTACGCGAAAGCTTTGAGGGAAGA

>V300080312L3C004R0330828567

AACATCTCTCTCTCTCGTCTCCTTGAGTTTAGGCTGTGCATAG

>V300080312L3C003R0010212706

CGCTCGACCATTTCAAAAATGTTGTCAAGGAAGAATTTGTGAT

>V300080312L3C006R0440770489

CTGCAAGCAATAGGTTATTCATCCTTAGTTGGTCTTCTACGCA

>V300080312L3C001R0691320020

CTGTGTTGGTTTCAAACGAAAAGGGTGTGCGTACCTGTATAAC

>V300080312L3C005R0590414081

GGGATATTTTATAGCATTAAAAAGGATGTATATACATGTTCTT

>V300080312L3C004R0570334282

TGACGCCAGAGTACAAGCCAGCAAGACCTTCGTTCTTGAGGAT

>V300080312L3C006R0430835128

ATTTATCCCTTTGCCATTATACGCTTCCTTCTCGATGAAAAGA

>V300080312L3C002R0511251037

GGAGAATTGGGATATTTTCCAGCTTCATTATCAAGATAATGCT

>V300080312L3C003R0600093868

CCGAATCCGCTATCTACAGTGATCCAAAGGACTTGATCAACGA

>V300080312L3C005R0230706124

CCGAGACTATCGTTGCAGTAGCAGAGAACTGGGCGTACCATCC

>V300080312L3C004R0431263820

GTCGCAATTTACCGACGTTGAAGGCGAAATGTTCAATCTGTTC

>V300080312L3C005R0580278530

CTCGCTTTCGTTGCCGCTGCAGTCCAGTCTGCACAGACGCAAC

>V300080312L3C003R0291023601

GATAGATGTGAACGATTTGTAGACTAGGAACTTTACAGGTTTA

>V300080312L3C002R0450008052

TATTCAATTCTCAATCTGGCAATAATCTAATGATATAATTGAT

>V300080312L3C003R0340281782

ATAACGAAATCGATATCAGCAGCATCAGCAAACAAAGGAAATG

>V300080312L3C002R0220657249

TAACACGAAACGCTCGCGTGATAGTCCGCAAGTTATCGATGGC

>V300080312L3C004R0340442304

TGAAGGGCTATCTCAACAACCCAGAAGCCAACGCCGAGACATT

>V300080312L3C006R0140623466

CCAAAATTGATTCGCGGAATATCACGACGGCCGATACCACAGA

>V300080312L3C003R0521259359

CACTTCGAAAATGGAAAATGGAACCCGGGGAGCAAAAGGAAGC

>V300080312L3C001R0120609679

CAAACACGCCGTCGTCAAATTCTCAACAGCCTGTGATTAGCTG

>V300080312L3C002R0441044463

TTACATGGTACAAGTCGTTTTCGATGGCATATATGGTAAGTCA

>V300080312L3C006R0451108207

GTCGCACTCTAGCTGAAATTGGACGATCCAAAATCTTCCCATT

>V300080312L3C004R0100411260

AAGACTGCAGATACAAAGCAGGTCGAACTGTACTTTGCAGGTG

>V300080312L3C001R0300586495

GCGACCCCACCGTCGTTTCTGTTGCCGATGGATCCAAAGTCAT

>V300080312L3C001R0310764507

TATGTTGAAGAATCCGGTGAGTTTCCTTGCTGCCAAATTTCGC

>V300080312L3C002R0180257060

GGTTTAGATGAAGATGCACATGCTGCCAGCGCTGTTACGATGG

>V300080312L3C006R0280819245

ATCACTCTTATTCTTTATCGCAGTGACAACAACAAGGTGAACC

>V300080312L3C006R0220831936

GCGCTTCCACAGCAACCGTCAAGCCCAACGCTGCTTCTGCTGA

>V300080312L3C002R0130574131

TTCATCAACCTTCGTTACGTTTACTTCCATGTTAAAAAAAGGA

>V300080312L3C004R0650607730

GGTTGAACGTTCTTTTGCAATAAACTTGTGTTTATCTTCCTGC

>V300080312L3C001R0370146211

AATTGAATTTCTGGAGAGATACACTGAGGTCTAAATGTTCTTC

>V300080312L3C005R0150402405

AGCAGCAGGTCGCGTCGTGGATGCGAGGAGCTGCGAAACAACC

>V300080312L3C004R0321195911

TCGTCACCACCACTGCCACCAAGCCCACCAGGCCAAGAATCGA

>V300080312L3C005R0090262555

ATGTTTGCGTTAATCCTTTTGCACTGGATCATATTGTCCATTG

>V300080312L3C005R0240327570

CAACGTCTGCTACACGTATGTGTGTAGATACTGCAGTGAGAGT

>V300080312L3C006R0700760539

GGATGTCAGAGGATGACATGGCAACCGCTAAGGAGCCATGATT

>V300080312L3C002R0700539268

TAAAACATTAGCCGCAGCGCGTATCCTAAGACTCGTCATGATT

>V300080312L3C002R0660568302

GGCAACAAGCACGGAGCATACGATATAGATACCGGCACAGTGC

>V300080312L3C005R0710890164

TTTCGTTCTAGACGCATTTAATTGGATTTTTGCGTTGGAGATA

>V300080312L3C003R0011310211

CTCACGTTCTTTACGTTTTTGGTACTCTTTCTGCTGTGCCCCA

>V300080312L3C004R0281117402

GGTTGCTACTTGTTTGCCGGCCGCTTTGCTCGGTGCAACGATG

>V300080312L3C005R0211010512

GGCGTATCACCACTGGAGAGAACTGGGTGAGAGAATTTGCAAT

>V300080312L3C001R0650083192

AGCTAATGAACCTTTGTCTTTCTTTTTTTCTTCTTCTCCCAGA

>V300080312L3C001R0450975239

CGCAACCTGGAGAACAGAGCCCTGCTAACAACTCTATCTCGAG

>V300080312L3C004R0401233443

AGCAGATATTGGAGTACATTAGCTCCATCCAGGTAGAGATGGC

>V300080312L3C004R0210843713

CGGAAACTTTTCTGCTACACCAATCACCACTAGTGTAAAGCAA

>V300080312L3C004R0170399871

GCAAAAGTGAAGCATGCAATGTATGACGTACCAAGAGGTAGAG

>V300080312L3C002R0021002381

GGCATGTGGGATAGGCCTTGCTAAATACAATTTTACATAAATG

>V300080312L3C002R0670009071

CCGAGGATCTGCATACGGTTGCAAAGTACTTTGAAAATCAAGG

>V300080312L3C005R0270744454

GTCATGGCTTGAACGATCAGGCACGTGTGCTGTGGGATTGTTG

>V300080312L3C005R0490575421

GCGGATCACAACTTTAAGGGTAAATACGAGGAAGTAGTAAACG

>V300080312L3C006R0510904216

GTCTATATGGTGATCCTCGAAAAGGGAGCGCAGCAATTCCGTA

>V300080312L3C001R0240333697

TGGGGTACGTAAAGAAGCCATAGATCGACGTGCGTGTCCTTTA

>V300080312L3C004R0350521963

ACAAGGCTTGCAGTGACTGGGCCAGGAAGAATGGCCGCACGTT

>V300080312L3C004R0520616553

CAGCAAACCCGGAAGTATGCATTATGCTTTTTCTGTCTGCACC

>V300080312L3C006R0060902468

ATACTTATCTTATCATTGCATGGCGTCAATCCAAAGAGTTATT

>V300080312L3C002R0010752301

ACCTTGAGACGCGCGCGCCTGTCGATCTAGTCAAGGAATATTA

>V300080312L3C001R0510174501

CCAAGGGCGGATACATTTAGCTGCAAATATGGCATGCGGGGCG

>V300080312L3C004R0290846766

ATCCACGGGGCGAACGGATACTTGATTGACCAGTTTATCAACA

>V300080312L3C002R0150900747

CCAGCAACAAGCGCACCGACGAGTAGTCGGATCGATTGAAAAC

>V300080312L3C006R0560539406

ATCGTTGCTGGTTTGAAACGGGACCTGCCATCGTCCTTCCTCC

>V300080312L3C006R0400539967

TGCTGGCTATGTAACGCTAAATAATTGCACGAAACAGCTTAAG

>V300080312L3C004R0450512121

GGCAAGGAAGCCCTTGTGATGGTGCGGATCTATCATATCTTCA

>V300080312L3C004R0691015787

TTGTACATTATCATGGAGGGCGGACGAGGCCGCATTTGTCGCG

>V300080312L3C003R0450130077

CTGTTGTGCAGAGTGATCATTGTCAGAATCTCCATAGATACCC

>V300080312L3C004R0720822116

CCCTAGTGCGTGACAACGTCCCTCCGCGCACGTTTTTTGCAGC

>V300080312L3C005R0220141076

CCGCGCACGTTTTTTGCAGCAGTGCCAGCATTGTCCTCTATTA

>V300080312L3C001R0340601396

CGCTTCCCACGAAACACCATTGATAGATGACTTGACAGTCATT

>V300080312L3C001R0430611902

AATAAGTGATGGGAGAAGTTCCGGTATCATAAGTTTGAACTTC

>V300080312L3C004R0170170358

TTCGTTTACAGTGATGACGAGCTTCTCCAACGGACTTCGATAT

>V300080312L3C006R0560450569

GTGGTACACACGACCCCAGCAGTGCCTTTTCCTTCTTGTTCTT

>V300080312L3C004R0650359505

CTCTTCTTGTTTTCTCGCCCGAATGTCGGCTACTCGCAAGGTG

>V300080312L3C002R0660121469

CCCAACATTCAGCAAGAGGCACTCGTTCAGGAACGTTATCGAG

>V300080312L3C001R0480559233

CAAGCGAACCTTATCCGCACTTTACTCGCTGCTAGTCAAATCG

>V300080312L3C005R0701072455

ATTGCTTTGTTGATATCACATTTTGATGTCCTCGGCACTCGAT

>V300080312L3C004R0670113381

TCAAGCCAGATAGTAAGTCCAAGTTCAAGTCTGTCCTGTTCTG

>V300080312L3C003R0490176175

GCAGCTCTCAACTCTCGGCATCGATTTCTTGAAAAAGTGTTTC

>V300080312L3C003R0100472926

CCACAAGCTCAGGCCGACGGTTGTAATGTAAAAGATTTCTAGT

>V300080312L3C006R0210770881

GACTAATGAACCAGTGCTCTTCGCTTTCCTTGTCCCACACCAG

>V300080312L3C006R0090503300

TACAAAGAGTGCTGTCTGAGACAGTTTGCTACCGGAAACCACG

>V300080312L3C002R0560443730

TGTGAAATGTCGGCAGAGTTAAAGAAGGTGTTTTTGCAGAAAT

>V300080312L3C003R0681189941

TGTAATTTGTATTTTTGATCGCCGATGCCAGATGCGATAACTT

>V300080312L3C002R0300293190

GGAGGTGTTCGATTGTGACAATAATTGATAGAGGAGTGGCTAG

>V300080312L3C003R0540928181

CGACCTTTTTCTGCAAATCATTGAGCCACTGTTTCTTGCGCTG

>V300080312L3C004R0040002691

CAAGCTGGGTACACTCGCTGTGCTGCTCAGCGACGACGATCTC

>V300080312L3C003R0050002139

TGCCTACACCGTCCATGAGAGGGGGAGGTCTCTGAGCAGCAGA

>V300080312L3C002R0280307571

TGCGAAAAAGCATAAAAACACGAGGCTGTTGTTAAGTAGAATT

>V300080312L3C001R0570676235

GGTGTGCTCTGTGATAACAATACATTTGAATGATCCCATCACA

>V300080312L3C001R0140481743

GAGATCGTTTAGGCCGATACTTGGTGATTCAACAAGACTCGTC

>V300080312L3C003R0561382528

TCGCATGAAAGAAACGGGTATCTAGAAAGAAGGACACGGATTA

>V300080312L3C006R0570511205

TAATTGTAGCTCTGTGATCATATCACATACTTGAATCGTCGAC

>V300080312L3C005R0251363843

CAACGTTGCACGCAGGTACCATCCGGAGAAGCTACATCGACAA

>V300080312L3C002R0290817247

TTCCGCTTAGATATGTAACATCTGGACCGACGTGACGCAGAGC

>V300080312L3C002R0290891858

AAATAACCTACCCAATTGACCAGTTGAAGCTTCCAGTAATAAC

>V300080312L3C002R0610237968

ATGCCGCTGGCATGAAAGAACTGTACTTGCAAGATGTCACGTA

>V300080312L3C003R0300143094

AAAACTTAGAATTTTTGACTACGGGATTCTCAGGGCAGAGACA

>V300080312L3C004R0680049699

TCAAAAGAAATATGCATACGCGGCCATCAGCGTAGTATCCGAC

>V300080312L3C006R0651106622

GCACTGTAGATGGACAACCACCTCCAAGAGATCCTTTAGATTA

>V300080312L3C006R0710991037

AATATGTCGGATACAGCACAACCAGCCAGCACTAGCTATTATA

>V300080312L3C006R0521172359

AAAATTGATATTTGCGGTATATGACCCATCGAATAACATGAAC

>V300080312L3C002R0050529317

GAACTTTGTTGTACAATACATGACCGGTGAGATGCGATCAGAG

>V300080312L3C003R0490131874

AGCTCCTGAAGAGGAGAAGGTTGTGACGACCGAGCCTTTTACT

>V300080312L3C002R0361263311

GCAATGAACTAGACTTAACCTTGTAACTGTGATAGGTCACTAC

>V300080312L3C005R0210980810

TAACGCTGTATATATCCGTTCTCTGGCGATGACACAGTTTCTA

>V300080312L3C003R0371205626

CACTCAAGTTGCAGCAAACACCACACGTATATCGGACATCCTT

>V300080312L3C005R0390549685

AGGATGAGCCCCCAGGCAGGTCAAGGTTGGCCCATCGACAGGC

>V300080312L3C006R0720826408

GACAGAGAGCGATGCGGACACCAGAGAATAGCCAGCACGTCTG

>V300080312L3C004R0210459590

GAAGGGCGGTTCCGTACCCTACGCCAGCAGTCCAAACCCAGCA

>V300080312L3C005R0290321926

CTGGCTGCACACTGGAGGAAGTGCCTGGCCTTTCACTGCTGTC

>V300080312L3C001R0320477609

AACATCAAATCAACCCACCACTGCGAATGTGGGACCCGAAAGG

>V300080312L3C005R0231288384

TCGGAATCGTGTAGCTGCAATCCGTTCGTAATCGTAAAAATCC

>V300080312L3C004R0270019743

TGATGACGGTCGTAAGATGCTTACTGGTACGAACTTTTGTAAC

>V300080312L3C003R0460293394

AGTATAATATTTGAAGTTGTTCACGGGGTAAATGAGCGCAGGA

>V300080312L3C001R0130124819

CTGAAAGATATCTATATACAAGCTTTTTATCTACTTTCTCATG

>V300080312L3C004R0231299903

AATATCGTCAATGACAATCAACTTGTTTCCGCACTTTTCATCT

>V300080312L3C001R0190035693

TTTCATCTTCAATGCGAGGTTCGCTGGACTGGCCATCGACTTG

>V300080312L3C003R0330021197

CCAGCAAGGTCGATTTGGACGACATACGTCACTGCGATGCCTT

>V300080312L3C003R0680390079

TAATTGGATTGATGTACTTTGAGGGTGCATAATGTGAAGTTGT

>V300080312L3C001R0680754704

CAAAACTGTGTACGAATGGATCTAGCAATTGCGTAGCTCAGGA

>V300080312L3C002R0270947038

CCACCAGATCCTTGCGGTCGCAACACCGACGCCTTTGTAAAGG

>V300080312L3C005R0540138752

TACCAAATACTTTGCAAAATAGGCTCAGAGTATTGAACTTTTC

>V300080312L3C001R0511347942

TGTCCAATTCTCGCTGTCTTTCAAGTAGCCGTAGCAAATACAC

>V300080312L3C001R0300640752

ATTTTGCATGAATTGCTTGTAAATAAATTACCGTGCTTCTCAA

>V300080312L3C003R0431184421

TCTCTATCTTTTGTAAAACTCATCATGTAAACGATGAAATGTA

>V300080312L3C001R0310879063

CATGTAAACGATGAAATGTATGGAGTTAGCAATACCTATCAGC

>V300080312L3C006R0280187537

ACGACGAATACTGAAAATGGAGATCAAGGCGATCAAGAAGAGG

>V300080312L3C005R0260659319

TAGGTTGCTTTGACTCCAGGTGTACAAAGAGTTTCCACTTGCC

>V300080312L3C005R0360676579

CCCCTCGTCATGTACGGTGACGACCAGCAGCAGGATCATTATG

>V300080312L3C001R0690866576

TGCAACCTCCATTGAGGATTACACTCACAAACCTTACCTTACT

>V300080312L3C002R0090175759

CAAGTATACACAGAAAGACGAGCGAGTGCTCTCAAACTTTTTG

>V300080312L3C001R0471305584

CGGAGCAAGTCACTAGACAGGCAACGCTTCGACGTTGATCTTG

>V300080312L3C002R0340131808

AATGCTAATTTTAGCCTGTATCATGGACGTCTGGAGCGCGACG

>V300080312L3C006R0370699806

CAACTTTGTATTGCGTTACACGCATACCTCGACGACTGGTACT

>V300080312L3C004R0610766917

GCATACCTCGACGACTGGTACTTACTTGGGTATCAATGGTACA

>V300080312L3C004R0180716976

TATCAATGGTACAACTGCACAGCAACCATCATCATCTCAAGCA

>V300080312L3C004R0210488872

ATTATTACAATATATATCAAAGAACGTTTATGTTTTTATTTTA

>V300080312L3C003R0260977924

ACTTTGTTAGCGAGACTGTCCACGTATTTACGCGCCAACCCGG

>V300080312L3C003R0680020112

TATTTACGCGCCAACCCGGAATACTTGCAAAGGTTTTGCTCCA

>V300080312L3C004R0550583482

AAGGCCATAATGATGACTAGGATTCCCTCGTTGTAAAATAGTT

>V300080312L3C003R0350389898

CCTCCTCTGCAGCCACTGGAAAATGGGATCAACGCGAACGAGA

>V300080312L3C006R0171117931

GGAAGAAATGTAAAATGTATATGATGCATGCATGCCATGTCTA

>V300080312L3C003R0550013463

CTTCTTCTTCTCTTCAATCTCTTGAAGCGTTTCGGTACCCCAC

>V300080312L3C005R0481194244

ACGCGTTCATGCCTCCAAGTACCTGGTTTTTAACACATTTAGA

>V300080312L3C001R0580088145

TAACACATTTAGAAAATATGATATGCATGGAAAAGTCAAACAT

>V300080312L3C004R0700342192

ATCACTGCTGGGCCGTAGATGGTGATAGGGAATGTACGTGTCC

>V300080312L3C002R0491292018

AAGTTCTCAACAATCACGATGGTAACGAAAACTGAAAAGCCAA

>V300080312L3C003R0050104322

GCAAAGGCCTATTCCATTTCTTTTCTCATCCTATTAAGCTCTT

>V300080312L3C002R0701322577

ATGGCAGCTTACTATTCAATCCCCAGAATGGCGTCTGTCGCTT

>V300080312L3C005R0571054241

CATGCGCTGATAGACCGCCAGCAATGTACAGTTTGTCGTTAAA

>V300080312L3C002R0371007105

AGGTTGGTTACAGATATCAAGGGGAATACATGTAAATATATCC

>V300080312L3C006R0241021269

GCATGATGAAAATAGTGCGGTGCCGACATTGGCTGCCAAATTC

>V300080312L3C001R0150114211

TGTCCAATTTCTCAGCTTCTAAATTGTTTAAACATCAGCTGCC

>V300080312L3C006R0080692172

TAATCCACATGGTCCTTTCCGCATTATCGGTGATCGATTTCCA

>V300080312L3C002R0140077952

ATTACCAGGAGCTCTTTATGGATGCCGTTCGCCAAACGGTTCA

>V300080312L3C004R0240396289

CTTCAATTACCAGATTGCTTTGCAGGCTATCCATGCATTGCCG

>V300080312L3C003R0701039762

AATGAATAATCCGCCTCGCCTCGGCGCCACATCCGATGTCCTG

>V300080312L3C003R0280694665

TGGAGAATTCTACGCAAGATGTTTTGCAAGATATTGAAAACCA

>V300080312L3C001R0070928991

CAACCAGCAGCGCCGTCGCCGGAGTCACAGCATCAGACGTGGA

>V300080312L3C005R0600763529

TTTTACTATTTCTTCGAGATTTTATCCACGACAGATTGCGTGA

>V300080312L3C003R0040060471

TCTACGATCAGAGCTGCAGGCTGAAGGCGCCGACTCGAGTTTT

>V300080312L3C005R0571106137

AGATTCGCCTCTGTACACGATCCTCAAAGCAGCGTACCTCTTC

>V300080312L3C001R0120675846

TGAAAACAAAGGAGCCGATGGCCGGTTCCGCGTCGTAGGCAGG

>V300080312L3C006R0710623161

CCTATAGACGAAACAGCTGATGGAAGCCCGTTAAAGAAGACAA

>V300080312L3C004R0650520254

AGCAAAGAAATACATTTCAGTACAACAGCACGAAATTATCATT

>V300080312L3C005R0411180445

GAATACCTCACAGTGACTGCCTGTAGACGTAACATGACGGGCG

>V300080312L3C004R0630761306

TACAAGTGCAGAAAGAAATCGCGAAACGGAACACTGTAAGCAC

>V300080312L3C006R0620185276

AGCGGTGGTGCCATGCCTGGCGGCGCTGCTACTGCTGGTGGAG

>V300080312L3C003R0380782476

TAAACCAGCTTATGCCATTTCTATTTAGCGACGTTGTTGTAAA

>V300080312L3C004R0071025449

GATATGTGCGATCGGCGTCGTATCCGCTGCGAGTAATTCATTT

>V300080312L3C005R0131075133

AGACGAGTTTTGAAACATGTACGTACGACGTCGCGCTTGTCTT

>V300080312L3C006R0050856134

GCGCTTGTCTTTTGACGATTTGCCCATGCTAAGCGGATCGTTG

>V300080312L3C004R0670544186

GGCGAGTGGGTTGCATGAAACGTTAAGGCGGGCGGATGTCGTA

>V300080312L3C005R0240799188

GCATACAAAAATCCGGCGTGTGTACACTCTTTCTTCTTTCTTC

>V300080312L3C006R0150978183

AAAAGATCCCAACGCGTCCTGATGGAACCCGTGCTCTCGACCC

>V300080312L3C002R0140190444

AAGTATCTCTAGGCACTTGTACATATATACTCGCAAGGTTTTG

>V300080312L3C006R0250488025

GGTCCAACAGCAGGCTTTGCCGGCGCAGTGGATTTGCCAAGCG

>V300080312L3C003R0280595633

ATCCAACACCTTGGTACTTGTTGCGGTTCTGTCTAGCCTTCTT

>V300080312L3C002R0190480047

AATAATACAGCAGCAGCATTTGTCCCGACTTGCAGAAACTACT

>V300080312L3C006R0281143861

CTGCCGAGGACCTGAAAAAGTTGGCTGTGCTTAAGCTGAACGG

>V300080312L3C001R0091327120

CTGATCCTATGGATTGATCCACGACACGAGACCCGACTTTTGG

>V300080312L3C003R0681224240

GGTTCGGGATACTCTAGACGCTGCACAGACTCGAGACATTGCC

>V300080312L3C001R0470541279

CTTGCGGTAAAAATGCAGCGTGACCCGGTTGAGCGTTGCAAAC

>V300080312L3C001R0451143242

TGCAAACAGGAGCTGGAGCGTGATAAATACAAACACCACCGTG

>V300080312L3C001R0690401320

ATTTTCGCAATTGATCTTGGGATGGATTAGGATTGTATTCTTC

>V300080312L3C002R0291072851

GTTGCCATTTCTGACGAACGCTCACAATGACTTTTCAGTCAAC

>V300080312L3C006R0060244567

CTGAACGATGCATATATCGTTGTCGTCGCTTTCGACAGAAGGT

>V300080312L3C002R0681396942

GGTTGCGAATCCGGCATTCCGTCGATCTATGCCAGTGGAAGTC

>V300080312L3C005R0690361579

GAATTGAAGGGCTACAAGCCTACTCCCGTAAGTTATCTGATAT

>V300080312L3C005R0360198910

TCTATGATCGCAACTACAATGTCATTGATGTCAAAGAGAATGC

>V300080312L3C002R0531011384

GAGAGAACCGACACTTGAAAGTCTCCTTGAGCGTTGGCGGATG

>V300080312L3C006R0170195845

AAATCATACGTCGACCTTGTTCGTGACCTTAGAGAAACGCTGG

>V300080312L3C005R0441168036

CAAGTTATGGCATCTTACTCATACGACGCCGATCTGCGCGAGT

>V300080312L3C004R0380199560

GCTGTCACTCAGGTCGGGTGGATCAGAAGGTTGCAAGAGATCT

>V300080312L3C001R0601156536

CCTCCACAGCCGGCGAGGAATTACTTTGTGCATCACGTCGTCC

>V300080312L3C001R0010952196

CGCGCAGCTCAAGAGTTTGGAGGACGAAGACGATGACTTGAAG

>V300080312L3C006R0680372778

TGAAAACGTCGTCGATTCAGCATCGGTGGTCGTTGTTGACGTA

>V300080312L3C005R0681070651

GGAGGATGTAGTCGATGATGCTTCTTTCTCGGTCGTGGTCGTA

>V300080312L3C001R0330099503

GGCATCTCTTTCGTCTGCTAATTCTACGAATCCAAACGGACCT

>V300080312L3C004R0501240268

CCGATATATAGCTTGACACCCATTCTGAAAGAGAAGATGTAAA

>V300080312L3C006R0261127144

CCAAGTCCAGTAGAGCTTTTGACTAATCTCAAAAGATATCAAA

>V300080312L3C004R0160662616

GCCGCCGCGAAAAAACCACTGCAAAGGAAAAGCAGCAGCCTGT

>V300080312L3C002R0190842857

GTTGAGCTGCTGCTGCATGCTATCTTTAAAGAACGTGATCTGG

>V300080312L3C004R0511004375

ATACAAATATACAATTTGAACAATGGCAACAACAAAATCTTGG

>V300080312L3C001R0500808512

CAGATTAAGTGCCCTAAATGCCTTATTGGTATGCCGGCTTACA

>V300080312L3C006R0150216806

GAACTTGACGACAGGTTCGAAAAAGGAGGTTCCTTTCTTTGCT

>V300080312L3C001R0061064536

CATATCTTTTATTCACGCTCGCGATAACTCCCGCTCCTTTTTT

>V300080312L3C005R0041124517

ACTGGTTAATTATTTTGCTTGAGTCTTCGCGACGATGATGAAT

>V300080312L3C006R0280684381

TCACACTTTCCTCTGGTTTCTTTCCGTTCTTTTCCCTTTGCAC

>V300080312L3C004R0570030334

CACTCCGGGGCTTGGAACGGTATCAGCTGCTCGCAAAGCTAGG

>V300080312L3C002R0111188812

GAGTAAGTACTAGTCACAGGCTCTTGTGTCATCGACGCACGCA

>V300080312L3C001R0690622132

TAGCATTATTTTCTTATATTAGAACTCTGCGAAGGCGGCGAGC

>V300080312L3C004R0360274837

CAGAGAACTTGTTGTTCGAGCCTATACCATTCATGAAACGCGT

>V300080312L3C006R0551131128

AACTCGTGCCGCCACCGTCGCAAGAAGATACAAACAAGCTCTT

>V300080312L3C005R0611380130

TCAGCCCGCTGGATCGGGATCCTCGAGAATAAGAAATTGACTC

>V300080312L3C002R0100344139

CAGCTGGCGGATTGGTTGAATTGGATGTGGATCCCAATGAACC

>V300080312L3C006R0150536402

AATGCAATTGTGCTTGAGCTTGAGGAGTGAACAATCCGCGACC

>V300080312L3C005R0640687152

ACAGCTGCAGACTCGTGGGATCGGGTGAGTTCAACAGGAGTAG

>V300080312L3C002R0181001750

AGAACCATGGCAGTTTCCTCAAGCGACCAGTCAATATCCGAGG

>V300080312L3C004R0240905017

CGCAGGTTTAAACAGGTTGCAGACGAAGATTTTGAAAAATTTC

>V300080312L3C006R0150115517

TCGAGATCGGATACAAACTCACGCTGCGGGATGATTCCAAAAC

>V300080312L3C001R0360973970

GATTCCAAAACATCAGCAACTGTACCTTATATGCAATGGCATA

>V300080312L3C005R0061265763

CTTTGCGTTCGCGAGTAGCCTCTCGCCGCTCGAGCTTCTTTTT

>V300080312L3C002R0601207758

GGGGGAGGATTATGCTCGAACTAAAGTACCCAGTGGAAAACTG

>V300080312L3C004R0450700754

GAAATACCAGACTTAGATTTCACTAACAACTGCGTTTTGTTTG

>V300080312L3C003R0440348386

GGGTCACAGTAGCTAGGTCTCACAACCGCAATGGTTCTAACCT

>V300080312L3C002R0600767948

CTTGCATCGATCTGATCATCAACGCCTGAATAAATACCCGTCG

>V300080312L3C003R0021124810

AATCCTCTGTATGTGAAGAGATTAAGCGTCTGCGACAGAAACA

>V300080312L3C003R0020874109

CGATTGAAAACGATGGCCTTGCACAGGAAAGGCAGCAGAGCAA

>V300080312L3C002R0010358669

GAAGCCTTTGAAACTCAAAAGGATACCTTTTCTGTCCCGGATT

>V300080312L3C003R0190749751

ACAAGCGCGTAGAGGCAATTGGAAGATACGCAGTTGCTGCAGT

>V300080312L3C003R0700625584

GGAAACAACATCGTTGTTTGAAGACGTTCTTGAACCTTTTGCA

>V300080312L3C004R0340783247

AAAAAGGAGGGATACAGATTTTATCTGTTGAACTGCCGGGAAC

>V300080312L3C002R0340422084

AATATTTTCAAGAAACTAAAGCAAACTTTTAGATCTTTTCTGT

>V300080312L3C006R0330369396

CCTCCAACGTAGCCTTCAGGAATATGATCCGATTACGAACGTA

>V300080312L3C004R0440827962

CGCGCCTCTCCTTCGTCTTCTCTTCCGCTACTACTGTGATGCA

>V300080312L3C003R0100236376

GATTTACGTAACATCGCTACCCTTTCTTGCTCTTCTCGTAAGA

>V300080312L3C002R0570924667

GATTTCTTATTGATGACGAATTTTCAAAACGTTGGGTTAGGAC

>V300080312L3C005R0390360895

GGTAGATATACGAAAATTTCACAGTTGCAGCTGTGCGCGATCG

>V300080312L3C001R0010984612

AGTCTGACACTCGCGTCGATTTTCGCGACCTAGTGCGCGATTT

>V300080312L3C006R0331113835

GCCCGACAATGCAGAGCCGCTGAGGCCGACTGTTAACCGTCGC

>V300080312L3C002R0290472007

CAAACTCGCGCCAAATCACACTCACAGTAAGCAACAATGCAGA

>V300080312L3C001R0090905714

AGATGCTTAACATGTGCAATGTAGATATCCAAGTGGAAGCGCT

>V300080312L3C006R0540048315

TCTAGTTTTGATGAATACTGCGTACTTCGCTGAGACTCCTTCC

>V300080312L3C001R0061166706

AGGGAAGGTGCAAGATAAACATGGACTTTTATGTTTCACCCGG

>V300080312L3C002R0420028193

GTCACCCCGCCTTTAGAAGAACAACAGGATATAGAATCACACA

>V300080312L3C004R0701055930

AGGAGGAAGGGGAATTTAGGGGAGATGATCGTCGTCTAATCAT

>V300080312L3C003R0130082523

CTTCCTGCCACATGGCAAAGCGTGCTTCTCGTTCTTTAACATC

>V300080312L3C006R0130457801

CCGTGTCTTCCACTAATGCATAGCTGTTTGTGTTAGCGTCTCT

>V300080312L3C004R0550401121

CCTCTGCTCGCAAACTCGTCAATTTCGCTAGGTTTCTTGATCA

>V300080312L3C004R0500499098

GGTTGTTTCATAAAACTGATTAGGCTGCAAATGGCAAAGACTG

>V300080312L3C004R0500915086

TAAGAGCAAAATGCGAGGTTGAGAAGATAAGATGGATCGCTTA

>V300080312L3C005R0350936401

CACATCGCAGTGTCAAACGTTAGTGGGAAACTGCTAGAAACAT

>V300080312L3C003R0390550375

GTAAGCAGATAAGAACGCATATGGAATCAGCATGACTTTGTTC

>V300080312L3C002R0570974566

GTATAGGATTCTTACTCTGATTATCCTCTTGTGTGATTACTGT

>V300080312L3C003R0650388966

AGCACGTCAGAAGCGATCCGGAGACCCACATAAGAAGGAGGAA

>V300080312L3C005R0460660807

GATAGGGTTGATAGGTACGATCATGCGACGATTATCAACGACT

>V300080312L3C002R0420599223

GTACTGGTGCTTTTTCCCGAAGGATCCAAGCTTTAAATGTGCG

>V300080312L3C003R0331091146

GCAAGGCTTGACAGCGGCGTACTATTCCTTAACGGTGAGTTCT

>V300080312L3C003R0080052661

TATTCCTTAACGGTGAGTTCTTCAGTTGTTTGCTGGTGGTTTG

>V300080312L3C004R0170628305

TATCCTCTCCAAGATCAAATGGTTCGTCGTCTTCAGCTGCTTG

>V300080312L3C002R0040472574

TCAATATTATGTACATCCTGCTGCTGTGGGGTCTGATGCTGCC

>V300080312L3C002R0200687180

CGCATCTCGTCTTTCCTGATCAGCGTTCATTGCGTCGATAAAA

>V300080312L3C001R0321090501

GTAAATACAAACGGATATTCACATAATTCGCAATATCTATGCA

>V300080312L3C005R0230846315

GATTTCGAGCATATGCGCAAACCCCGATTTTTGAGATGCAAGT

>V300080312L3C003R0390261025

TCGAGCCACGCATTGCATGTGAGAGCACTTTGGTGGCTGCTGT

>V300080312L3C001R0270277214

TGAGCGAGAGAGCATTGTTCTCCTCCTCCCCTGCAGGCCGTTC

>V300080312L3C003R0050397690

AATCCATCAGAGCCTAGTAGGAGCCCCTTTTGGTGTCAAGGCC

>V300080312L3C002R0231241836

CAAGGAAGTTATTTACAATAAGCATCTAGCATCATCGATATAC

>V300080312L3C003R0551248467

CCTAGTAGCGGAATTCGCCAAGGCAAAGGAAGATATTAATGCA

>V300080312L3C006R0211321139

ATTCGCCAAGGCAAAGGAAGAAATTAATGCAAAGCTCTCCTTG

>V300080312L3C002R0241291080

CGCGGCTTTCCCGTACGAACGGCGAGGCAAATGTTCTGTAATA

>V300080312L3C002R0250213968

CAAAGGTTTTGTTTGTTAAACGGGCTATATAGCGGCACGGCTT

>V300080312L3C006R0250276822

TCACAACATCTACAAGAGACGTTTCAAAGAACCTACTAACCTC

>V300080312L3C003R0310692218

ACGAACTTGCCTGCAAAGAGCATTGGCCCCATTTGAACTTGGT

>V300080312L3C005R0170919520

CGAACTGACCAGCAGTCGCTGGAACGATTGCGCAAATTGCGGT

>V300080312L3C002R0710382496

GTCGAACGGCGGCGGCCAGCCCAACCGCAACGGCGGACTATCG

>V300080312L3C006R0561358007

CCAGCCCAACCGCAACGGTGGACTATCGTCTTTGCCTATCAAG

>V300080312L3C002R0571139571

GCTTCTTATCTTCCCCCCTTCTCCTCGTATCTCTGTACTATAC

>V300080312L3C005R0641406552

TTTTCCGTTTCGCTCCTGTTAATGTTGGAGTGCTTTTACTGCC

>V300080312L3C001R0170001295

CAGTAAACAACGAGAAAAAGAGAGATAGAGAGCAATATAGAAA

>V300080312L3C005R0090320915

ATCTTGATATATGTTCCATCAATGCCCTGCCCAATTCATTGTT

>V300080312L3C003R0540592052

GCAGATATCCGACCGCAGGCAAAAGCAAGGAAAAAAGTCCAGG

>V300080312L3C005R0611027254

AGTTCGGTTACTTGGAGTGAATATGTGAGCGGCGGGGGTGCGA

>V300080312L3C004R0590487218

CGTGCCTGCTAGAACGTGTTTTAAAGTGGTCCAATCGTTAAGG

>V300080312L3C002R0170273680

CTGCACAGTTGAATATGCATACAATTACCCTCAAGCATTGCTC

>V300080312L3C004R0321001416

AGTTATTGAGATGCCTTTGACTTGTTGAGATTTGCTTCTGCCC

>V300080312L3C003R0570341530

GTTCAAGTGTCGACGACCGACCTTCCAACGCTTCAAGTTACCG

>V300080312L3C006R0150296496

AATAGACAAATGACGCAGTCTCTGGTAGATTGCAAAAGCGATC

>V300080312L3C002R0660617753

GGATAATTCGACGGCTCAAGACCGTCGCAACGAGACTCATGAA

>V300080312L3C001R0721093565

AACATCCGCGGGGACAGTAACGTACTCGCCGACCAAAGCAAAT

>V300080312L3C004R0680381798

CATGTTAAATTATGTTTTTCGCCAAGCATACGAGTTTCACACC

>V300080312L3C004R0720462914

TGGTGCTGCGGAGCAGGTATGTTGTGACAATGAAAAGTGTGGG

>V300080312L3C001R0700788326

CCCGATAAAAGGTTGTCGGCATGTTCCCTTTTTTCTAATCAGC

>V300080312L3C003R0461269250

TTAGGCAGGTCTCGCATCATGGCTTCTTCTTTGGATGTAAACT

>V300080312L3C005R0380587955

GATGTGCCAATGTTGATATCTGGTAACGTCTGTGGAAGTTCCC

>V300080312L3C006R0181309105

CAAACGATTCATTCACGTCAGCACCGACGAAGTGTACGGTGAA

>V300080312L3C004R0010187750

GGTGTCCTCGCATGGGATTCGAAGAAGGATTAAGAAAGACAAG

>V300080312L3C002R0590815141

CTTGTTAGAAGGAGTTTTGCTGCTGATCCGTAATGCTTCCTTG

>V300080312L3C003R0380124976

TTGAATATTTCGCATTAAGTTCTGCTGTCTTCTCCTCGCCTCG

>V300080312L3C002R0630258317

CCAGAACTTTCTCTTCAATCGCATTCTATATAACAATTAATTG

>V300080312L3C001R0700177304

CGTCAATGGCGTCGATGCGCTCTTCCTGGCTCGTTTGACCGTC

>V300080312L3C004R0580097783

TTTCGCTGCATAGGACTCATTTTGACGTAAAGATTGATCTCTT

>V300080312L3C002R0451336091

TGCTGTCGCGCTACATTGAAATCAAGGGAGTTTCCCAGCAAGC

>V300080312L3C001R0150882717

GCTATATTAGCTGGATCAAGTCCTTCGCATCACGGGTTTCTTG

>V300080312L3C006R0630888107

ATCACGGGTTTCTTGCCTTTCCAAAGACCCTTCAGGACAAAAG

>V300080312L3C005R0480714476

ACCACAAATCCAACAAGTCGTTTGATATATTCCTAAAGGATAC

>V300080312L3C003R0311161246

ACGATACAAAGCAGCGCAGTATTAGGCAGCAACAGCAGCAGCA

>V300080312L3C004R0230312327

TTGAAGAAACTGAGCTGGAGCGGTATACACAAAGAATTTCGAC

>V300080312L3C006R0711293975

TTTACTTATCAGCTTACATTGGTAAGTATATTGATTCCGTTGC

>V300080312L3C006R0070544171

ACATCAGATCACGATATCTACTCTTATCGGTAGACTTACACCT

>V300080312L3C004R0620973245

CCGTCAGGGGAATAACTCCCTACTACTCTGTCAAGAACATTTG

>V300080312L3C004R0090251253

GACAAGGATACCTGCAGATGAAGGAACAAACGAACCACAGCTT

>V300080312L3C002R0711011930

ATTTGATTAATAGCTTCTCAATATTAGATCTACCATGTGCTGG

>V300080312L3C006R0420404317

GCACCGCGATGCATGCTTTGCCCTGACTGCATGGTATCTTGTT

>V300080312L3C005R0660696647

AATTGATGATGGAATCATACCACTTGAGGCTCCTGAGGAGATA

>V300080312L3C006R0700660717

TGCAGCCAGCCGATGACGAAGACGATATGCTGGTACCCGAAGG

>V300080312L3C004R0590552623

CGCCCGCCCGTCCGCCAATCGCCAGAGCTAATAAATTAGCGGA

>V300080312L3C004R0081053120

AGACAACGCAAGATACAACAAACGCGTACGGTCTATGGCAGAC

>V300080312L3C005R0140816740

GTAGCGGCAGTGCTGAATGAGCTCCAAATACGAGAATACATGC

>V300080312L3C001R0240110614

CGAGTCAACGGACGGCGGCGGCAGTGGCGGTTGAGACATGGAT

>V300080312L3C006R0530002191

GCCTTTATACTTGCGGATTCGCTTCTCAAGCATGTGGTTGCGT

>V300080312L3C001R0360121463

GTCGCTGCTTCTCTGCTTCGTGATCCATTTCGCGCATCTTTAC

>V300080312L3C003R0440268398

CTTCGTGATCCATTTCGCGCATCTTTACAGTGAGCATTTCTTC

>V300080312L3C001R0690917672

GTCAAGTCCCGACTGATGACGCGCTCGTAAGGCGACGTCTCGA

>V300080312L3C003R0520460973

TAATTTCTCGGGTGACGAGGGCACCTTGCGACGCTCGGCCAGC

>V300080312L3C004R0310902970

AGGGAGGGAGCAAAGAAGAGTAGTAAAAAATCAGGTACGAACT

>V300080312L3C005R0181291904

AGCTAGAGAGATATGAAGATATAGAGAACAAATGGAAAGAGTC

>V300080312L3C006R0050382453

GATATAGAGAACAAATGGAAAGAGTCATGTGTAAAAAATTGCG

>V300080312L3C003R0570591620

CGTGTGTGTGTGCTTTTCGTGTGTGCTATGTTGACTAGTAGGT

>V300080312L3C002R0200987030

CATCAGTATGAATTTAAGTCGGAATCGTCCTTCCGTGCGCACG

>V300080312L3C005R0520004004

TAACATCTTGGAAACACGAGCAGATTGACAAGGATCTTTGCCG

>V300080312L3C004R0600233388

TGCATATAATGCTACTGATAGATGGTGGCTGGTAGACCATCCT

>V300080312L3C001R0590267541

CTATCCAGTTTGATGGCCGATGGACTATCAGTCATTATCAGTT

>V300080312L3C002R0450509892

TGGTGCCAACATCGTCGCGGTTTCGCACGTTGACAGAGTTGGT

>V300080312L3C001R0230246921

ACTTTTCCAAAGCAGCATAGTCTTGCTGTGATACAACGCTGGG

>V300080312L3C003R0411292749

CCAGAATACTTTCTTGCCCTCGGGGTTTTCAAAGTCAAGCAAC

>V300080312L3C003R0660525784

ACCTTTTACGAATGCATCGTACTCAGCCTTGAGCCGCTCAAAC

>V300080312L3C005R0121336319

CGAATGCATCGTATTCAGCCTTGAGCCGCTCAAACATTTGAAT

>V300080312L3C005R0720262179

GTAGTCACATCTCCATTACACACTTACATCAAGATCGGACACC

>V300080312L3C005R0361357915

CGGGAGTTCCACTGCAAAATCGTCTGGTAATACGTAAAACTCG

>V300080312L3C004R0561200219

CGATGAACACTTTAAAGCAAAAATGGGAAAACTTCTTACCAAG

>V300080312L3C005R0710068572

TACGTCTAAAATCGGTTGCTGGGAACACATTGCTGGGCATCTC

>V300080312L3C005R0420730821

GCATAAACTTGGAAAGGCAGTCAGGGCTGTGCGAGACGCTGCT

>V300080312L3C003R0280072958

CAAACAGAATAAAAATGGTCCCTCATTTTTTGCATGCGATGAG

>V300080312L3C004R0231396837

AGCAACTATTTACTGTTGTGGTCTAAAAATACGCACTAAGATT

>V300080312L3C004R0531325201

TTTTAGAGAGTTCTTTTGGGAACGCCGCGAGAATAAGTCGCAC

>V300080312L3C002R0610579704

TTGCAACAGAGATGATACGACATTTACGCTGCCTTTGGTGGAA

>V300080312L3C005R0261076223

GAAGAGCGGACCTTCTTGTATGATATCGAGAGTCTCGATAACG

>V300080312L3C003R0381225349

GATATCGAGAGTCTCGATAACGTTTGGATGATGTAGCGTGGAC

>V300080312L3C002R0441024146

CCTGGACCCGTTTTGTGTGAGAACAATGGAGTAAACAAATAAA

>V300080312L3C004R0161204408

TGTTCGCCCAGTCGCATCAGTTCAAGATGTGACCGTAAAGCTC

>V300080312L3C006R0511002553

AGACACAGATAAAGATGTATGATGTCTCTAGCATTCCCACCGG

>V300080312L3C005R0490965166

AACATCAATTCGGAGCGTTCTAATGTACAAAGTCCTCTTTCAA

>V300080312L3C003R0610128564

AGCATGCAATCACTTGCTAACACGTATGCATAGGCGCCACAGG

>V300080312L3C002R0190147911

GTGAGACAACGAATTATCATATTAGAACGCCAATAAGAACTCT

>V300080312L3C002R0350976706

CGCGTAAGTGCCTTTGTGTGAGCATGGCAAACGTTCGAATCGC

>V300080312L3C002R0260566468

ACTTACTGCAATGCTATACGATATTTTGGACCCAATTTTTCTA

>V300080312L3C003R0680165823

TAGCATTTATTGAGCATCAATCCTACTACCGATGTTATCCAAC

>V300080312L3C006R0251280256

ACAATACCGGCATTACCAGTCTACATTGTCTATTTTTTGTTCC

>V300080312L3C001R0370203435

CAGCGCTGGAAAGCACGTCCCTCGCACTGTCTTTGTCGATTTG

>V300080312L3C005R0010178865

GACATTGAGCGCCCTACTTATGCCAACTTGAACCGCTTGATTG

>V300080312L3C003R0280396165

AGAATCCAAGTACGCGTAAAATTATTGCTCTCACGCTTTTCTG

>V300080312L3C002R0190127424

GCTTTTCTTCGGATACACCGTCATGCTTTGCAAGTGCTTCGAG

>V300080312L3C001R0280014092

GCCTCCTTGACCTCTTTATCGCCTATGCTGGCTGATAAGCATC

>V300080312L3C005R0560643891

CTGTGTTCTTTGGCGCGATACGGTTCCAGACATCTGTTGTTGT

>V300080312L3C004R0400410091

CGTGCCTTATAAGGTACCATAGGAGGCTGATGTAGGAAATCGA

>V300080312L3C003R0500221817

GGTACCATAGGAGGCTGATGTAGGAAATCGACGCGTTGCTCTC

>V300080312L3C004R0331099427

ATTTTAAAAACGCATCGACGATCGACTGACTTCTCGGTCGCTC

>V300080312L3C005R0141188675

GGGACGTTTGAGAGCTGTTCGATAGCGAGCAGAGCCTATCATG

>V300080312L3C004R0390906497

TCTAATAATCCTTTCAGGAGCTGCTCACACATAGAACTCCTCA

>V300080312L3C005R0070614856

GGAACGGATAAGATAATTATTTGTTGTATTCTGATACATGCAT

>V300080312L3C004R0600324560

CGTTCCGTAAATTCGCATAGCATGGTCTGCATTCTCGGCTTCA

>V300080312L3C002R0340477014

TTGATGGTCGCCAAAAGCATACGTTTCCACTAGATCATTCTCC

>V300080312L3C006R0460125699

TATTGCTGTTAGCTATTTGCTTGTTCCTGACGATCCGATGGTA

>V300080312L3C006R0410749955

GACTATCGAAAGTGCTTGTAGAACGCGCTTCTGTCTTTCTATT

>V300080312L3C003R0580760457

CTATCGAAAGTGCTTGTAGAACGCGCTTCTGTCTATCTATTCC

>V300080312L3C001R0681312861

GTAATCGTCGTAGAGAAAGGTATCAAAATGAGAAGTCTTGACG

>V300080312L3C003R0320260029

TATGAAGCACACTCGTCTCTGTAGAATCCGCATCTCGGGAATG
[truncated: 1,940,474 more chars]
